# Cannabis- and Substance-Related Carcinogenesis in Europe: A Lagged Causal Inferential Panel Regression Study

**DOI:** 10.3390/jox13030024

**Published:** 2023-07-18

**Authors:** Albert Stuart Reece, Kellie Bennett, Gary Kenneth Hulse

**Affiliations:** 1 Division of Psychiatry, University of Western Australia, Crawley, WA 6009, Australia; 2 School of Medical and Health Sciences, Edith Cowan University, Joondalup, WA 6027, Australia; 3 Faculty of Health Sciences, Curtin University, 208 Kent St., Bentley, Perth, WA 6102, Australia

**Keywords:** cannabis, cancer, epidemiology, causal inference, genotoxicity, epigenotoxicity, carcinogenesis

## Abstract

Recent European data facilitate an epidemiological investigation of the controversial cannabis–cancer relationship. Of particular concern were prior findings associating high-dose cannabis use with reproductive problems and potential genetic impacts. Cancer incidence data age-standardised to the world population was obtained from the European Cancer Information System 2000–2020 and many European national cancer registries. Drug use data were obtained from the European Monitoring Centre for Drugs and Drug Addiction. Alcohol and tobacco consumption was sourced from the WHO. Median household income was taken from the World bank. Cancer rates in high-cannabis-use countries were significantly higher than elsewhere (β-estimate = 0.4165, *p* = 3.54 × 10^−115^). Eighteen of forty-one cancers (42,675 individual rates) were significantly associated with cannabis exposure at bivariate analysis. Twenty-five cancers were linked in inverse-probability-weighted multivariate models. Temporal lagging in panel models intensified these effects. In multivariable models, cannabis was a more powerful correlate of cancer incidence than tobacco or alcohol. Reproductive toxicity was evidenced by the involvement of testis, ovary, prostate and breast cancers and because some of the myeloid and lymphoid leukaemias implicated occur in childhood, indicating inherited intergenerational genotoxicity. Cannabis is a more important carcinogen than tobacco and alcohol and fulfills epidemiological qualitative and quantitative criteria for causality for 25/41 cancers. Reproductive and transgenerational effects are prominent. These findings confirm the clinical and epidemiological salience of cannabis as a major multigenerational community carcinogen.

## 1. Background

Whilst the link between tobacco and alcohol and several cancer types is now well-accepted, the relationship between cannabis and cancer remains unresolved [1,2]. As commercial considerations continue to advance, cannabis liberalization and increase dosages and availability internationally the impetus for the provision of useable public health information on this association becomes correspondingly increasingly important [3].

The cancer for which the strongest evidence exists is testicular cancer [1,2] where the link has been replicated in four major longitudinal studies [4,5,6,7] and the association is widely recognised to be powerful and causal [1,8,9,10,11,12,13,14,15,16]. One meta-analysis found the relative rate of testicular cancer to be elevated 2.59-fold (95% C.I. 1.60–4.19) after cannabis exposure [10], and dose response effects have been described for frequency of use [5,7], total dose exposure [4], long-term use [7] and age of first onset [5]. The issue of testicular cancer is important as the average incubation period of the preclinical oncogenic phase in this disorder is about 34 years, which is greatly accelerated by cannabis exposure to about 14 years [17]. Moreover, as this tumourigenesis occurs in the male germ cell epithelium, the clear possibility exists for the transgenerational transmission of genetic or epigenomic damage to following generations. Moreover, it is not inconceivable that the pro-oncogenic effect seen in the testis may also be occurring in other tissue beds.

Cannabis has also been shown to be an important factor leading to hepatic cirrhosis [18,19,20] and is recognised as being involved in hepatocarcinogenesis, particularly in the context of cocarcinogens such as chronic hepatitis B and C infection [18]. Cannabis has also been shown to be powerfully pro-oncogenic by multiple cellular, vascular and immune mechanisms in the liver and that its pro-oncogenic effects occur three orders of magnitude (over 1000-fold) lower than those of its anti-oncogenic effects [21]. Epidemiologically this is important as the rise in hepatocarcinogenesis in many places is widely attributed to altered ethnic patterns and viral infection rates [22] with the impact of increasing cannabinoid exposure typically overlooked.

Cannabis exposure has also been linked with cancers of the brain [23], head and neck [24,25], larynx, lung [26,27,28], urothelium [29,30,31], prostate [32] and cervix [32]. However, these reports are not without controversy. For example, amongst tumours of the upper aerodigestive tract, both positive [24,26] and negative [33,34] reports exist and the issue has been considered to be undecided [1,2]. Cannabis has also been linked with childhood cancers after parental exposure in acute non-lymphoblastic leukaemia [35,36], neuroblastoma [37] and rhabdomyosarcoma [34,38], thereby documenting a clinically significant intergenerational transmission of genotoxicity [39,40].

Important additions to this classical literature have occurred more recently with the demonstration in the North American context that the rising community cannabis consumption is linked with the major tumour of childhood acute lymphoid leukaemia, that increased cannabis use is a major driver of the 50% rise in total paediatric cancers [41], and that community cannabis exposure has been linked with cancers of the breast, pancreas, liver, thyroid and acute myeloid leukaemia. Liver cancer incidence has been noted to be rising in many places [22] and pancreatic cancer mortality has also been noted to be rising in recent years [42,43].

In addition to carcinogenesis, congenital anomalies (birth defects) form another major metric of genotoxicity observed clinically [44]. It is therefore highly relevant that tripling levels of community cannabis exposure have been linked with a tripling of total birth defect rates in Canada’s northern provinces, and increased levels of cannabis exposure have been linked with higher rates of dozens of congenital anomalies in Hawaii, Colorado, Australia and the USA [44,45,46,47,48,49], affecting most major organ systems (cardiovascular, gastrointestinal, genitourinary, respiratory, neurological and body wall), including limbs and chromosomal anomalies, trisomies and monosomy [44,45,46,47,48,49]. Much data have come to light recently as a result of large studies of national and transnational datasets on this subject [44,46,50,51,52,53,54,55,56,57,58,59,60,61,62,63,64,65,66].

In a similar way, a series of recent studies has linked cannabis with accelerated aging at the organismal [67], cardiovascular [68], epigenomic [69], metabolic [70,71,72,73,74,75,76,77,78,79,80,81,82] and immunomic [83,84,85,86,87,88,89,90,91] levels and in regard to the heightened incidence of morbidity [67] and mortality [92,93,94,95,96,97,98,99,100,101,102,103], which accompanies aging syndromes, all of which amplify our understanding of the severe clinical impact and long-term magnitude of cannabinoid-related genotoxicity [104,105,106,107,108,109].

The European context provides an ideal situation to investigate the cannabis–cancer link further, given the availability of data across all relevant fields. The subject of European community exposure to cannabis has been relatively confusing and complex, but with the recent publication of a major public health resource, country-wide trends in cannabis exposure have been greatly clarified [110,111].

One of the most exciting fields of modern enquiry in the basic sciences relate to the increasingly powerful insights being gained into the regulation of genomic expression and its modulation within the cell nucleus [112,113,114,115,116,117,118,119,120,121,122,123,124,125,126,127,128]. Whereas many studies in recent decades have focussed on genomic or transcriptomic or epigenomic levels of monitoring gene activity, some of the deepest insights and many of the leading current papers are actually focussing on the cooperation and coordination between these levels and others to coordinate and control gene expression within the 3D space of the nuclear architecture [129]. Gene expression has been shown to be controlled by the looping of chromatin through cohesin rings with boundaries formed usually by CTCF, which control the access of enhancers to promoters, acting both in *cis* and in *trans*. Recent epigenomic studies have shown that cannabis disrupts this machinery at many levels, including the synthesis of histone proteins [130,131,132,133,134], interference with the basic epigenomic machinery for histone and DNA methylation and demethylation and DNA acetylation, active disruption of energy-dependent modification of nucleosome positioning through SMARCA2/4, the disruption of both energy generation in the cell, the actin and microtubular cytoskeleton and its dependent epigenome, as well as the disruption of both CTCF and the cohesin ring motors [135,136,137,138]. All of these changes can be expected to be pro-oncogenic [138,139,140,141,142,143,144,145,146]. In particular, cannabis has been shown to widely disrupt the epigenome in many respects and cause widespread genomic demethylation, which is a change very characteristic of epigenomic aging [105,108,147,148,149,150,151,152]. Indeed, one very insightful longitudinal study recently demonstrated 810 cancer-related hits in its spectrum of differentially methylated genes [137]. Hence, major new advances in the cannabinoid epigenomics [135,153,154,155,156,157,158,159,160,161] have ushered in a whole new paradigmatic advance in our understanding of the widespread perturbation of normal nuclear physiology by the cannabinoids apparently acting as a class-wide effect [108,109,132,136,162,163,164,165,166].

The present study was based on the hypothesis that the oncogenic effects of cannabinoids identified in vitro would extend beyond testicular cancer to an undetermined number of other tumour types. This paper therefore sought to study in overview the association between community cannabinoid exposure and clinical cancer incidence across Europe for forty different cancer types in recent decades in bivariate and multivariate frameworks, to determine effect sizes and public health impacts, to ascertain the potentially causal effect of these associations by the quantitative techniques of formal causal inference and to compare population health findings in Europe with those from North America [167,168,169]. Given the high density of cannabinoid receptors in the reproductive tracts, the myriad important functions of cannabinoids in these tissues in both sexes and the importance of potentially inheritable genotoxic effects [170,171] and the above-cited findings relating to reproductive tumourigenicity were a particular focus of interest. Based on the findings of a similar analysis of trends in the USA, we hypothesised that cannabis would be positively associated with eight cancers—breast, liver, thyroid, pancreas, oropharynx, kidney, melanoma and acute myeloid leukemia—amongst others [53].

Therefore, the four basic questions investigated in the present study were:

(1) Is there evidence for a link between cannabinoid exposure and patterns of cancer incidence in Europe?
(2) How do these findings compare with similar data from elsewhere?
(3) How do the putative carcinogenic effects of cannabis compare to those of the known carcinogens, tobacco and alcohol?
(4) Was there evidence of inheritable tumourigenicity or cancerogenicity?

The basic hypotheses, investigative questions and analytical procedures were determined prior to commencing the analysis.

## 2. Methods

### 2.1. Data: Cancer—Annual Country Rates

Cancer data were taken from the Cancer in Five Continents (CI5) dataset publicly available from the International Association for Research on Cancer (IARC) and the European Cancer Information Systems (ECIS) website [172,173]. Data for 26 cancers were provided directly from ECIS and the included age-standardised rates (ASRs) calculated for the world-standardised population for 1973 (ASRw) [174,175,176]. The cancers for which ASRw rates were provided directly from ECIS were: all cancers (excluding non-melanoma skin cancer), anus, bladder, brain and central nervous system, female breast, cervix and corpus uteri, colorectum, gall bladder, Hodgkin lymphoma, kidney, larynx, liver, lung, melanoma of skin, multiple myeloma, non-Hodgkin’s lymphoma, oesophagus, vulva and vagina combined, ovary, pancreas, penis, prostate, stomach, testis and thyroid.

The ECIS data collection essentially terminated in 2012, albeit it is understood that the archivists there have recently issued a further call for data from member registries. In order to update the centrally available data resource, we also contacted the national cancer registries for each of the European nations as described in the Results Section and accessed specific data downloads from them and also their publicly available online data materials.

It is appropriate to offer some explanatory comments on terminology. Many registries provide data on “all cancers”. This was studied as a listed type of cancer and appears on many of the tumour lists in this report. Along with “all cancers” many registries list a group called all cancers (ACs), but not non-melanoma skin cancer (ACnNMSC), a grouping which omits small and superficial cutaneous malignancies which are not usually considered as constituting a clinical cancer syndrome per se. This second group is referred to specifically where that is the group being referenced by the cancer registry concerned. That is, we were faithful in this report to continue the nomination supplied in the data provided to us from the registries.

A second point of confusion relates to the designation of “oropharyngeal cancers”, both local carcinoma of the oropharynx (International Classification of Diseases version 10, ICD10) Code C10 and, in some cases, all of the tumours of the head and neck (ICD10 C00-C14). It appears that both groups have been designated as oropharyngeal tumours by different registries. Where we were provided information as to which of these two groups were indicated, we have been faithful to preserve this in the present analysis. We refer to local tumours of the oropharynx itself as “oropharynx” in this report, whilst any reference to the broader category of head and neck cancers, we denote as “Oropharynx_Broad”, which is a reference to the broader sense in which this term is used.

### 2.2. Substances—Annual Country Estimates

Tobacco and alcohol consumption was downloaded from the Global Health Observatory of the World Health Organisation [177]. The tobacco metric was the percentage of the population exposure to tobacco. The metric of alcohol consumption was the number of litres of pure alcohol consumed annually per capita (over 15 years of age). Data for exposure to various addictive drugs were taken from the European Monitoring Centre for Drugs and Drug Addiction (EMCDDA) website [178]. This data extraction was facilitated by a recent report, which presented a thorough exploration and extraction of the EMCDDA data on cannabis and other substances of concern [111]. Cannabis use metrics, which were available, included last year’s and last month’s cannabis exposure. Near daily/daily use data were also available. Data on the Δ9-tetrahydrocannabinol (THC) concentration of cannabis herb and resin were also available. EMCDDA past-year-use data were accessed for amphetamine and cocaine exposure.

### 2.3. Household Income

Median household income data were sourced from the World Bank [179].

### 2.4. Data Analysis

Data were processed in R-Studio version 12.4.1717 (2021), which was based on R version 4.1.1 (2021) [180]. The analysis was conducted in February 2023. Data were manipulated using dplyr from the tidyverse [181] and graphs were drawn in ggplot2 [181], also from tidyverse. Graphs are presented in ordered metrics. Graphs were arranged using R packages ggpubr, cowplot and patchwork [182,183,184]. Maps were drawn with sf (simple features [185]) and rnaturalearth [186] and coloured with palettes from viridis, viridis light and RColorBrewer [187,188]. Colorplaner was used to generate the bivariate fill palettes for bivariate maps [189].

Data were log-transformed as guided by the Shapiro test. On occasion, *p*-value adjustment for multiple testing was conducted using the false discovery rate (FDR denoted as P-FDR) adjustment of Benjamini and Hochberg [190] or by the method of Holm [191]. The Holm method, which is the more stringent of the two methods, was preferred throughout and is listed in many tables. Correlation matrices were compiled in the R package WGCNA which tolerates missing data [192,193]. Correlograms were generated with Corrplot [194].

For the categorical analysis, substance exposure cohorts were broken into higher compared to lower exposure groups. Indices for prevalence ratio (PR), attributable fraction in the exposed (AFE) and the population attributable risk (PAR) were calculated in a modified version of the R package epiR customised specially to handle the very large integers involved in the multidecadal European population by Professor Mark Stevenson (version 2.0.57) [195]. The R package collapse was used to access the ‘not match’ function for data manipulation and subgrouping [196].

Multivariable modelling was employed to compare the adjusted effects of the different covariates. Mixed-effects models were performed using the nlme R package with the cancer registry as the random effect [197]. Panel regression was performed using the pooled approach across space and time simultaneously (“twoways” method) using the plm package from R [198]. This technique was particularly useful for generating time-lagged models. Model prediction was performed using the predict function from the stats library which ships with Base R [180]. Due to the constraints imposed on regression techniques by missing data, the number of cancers studied by multivariable techniques was less than that analysed in bivariate techniques as described in Results. A panel of 36 cancers was studied with one analytical pass using purr–broom–predict workflows from tidyverse, broom and R-base [181,199,200]. The model Akaike information criterion (AIC) was used to measure the goodness of fit to predicted data. Models were compared using ANOVA tests in package stats. Data were listed as mean ± standard error of the mean (S.E.M.). *p* < 0.05 was considered statistically significant.

### 2.5. Missing Data: Interpolation

Linear interpolation was used on the substance use and income datasets. Another alternative to the significant missing data problem was multiple imputation; however, multiple imputation methods were not available at the time of writing for mixed-effects or panel model analysis.

### 2.6. Causal Inference

The formal methods of causal inference were utilised as follows for positive associations following standard public health practice [201]. All panel models were inverse-probability-weighted. Inverse-probability weighting has the effect of transforming an observational study into a pseudo-randomised controlled study by evening out exposures across study groups. It was performed in this analysis using the ipw R package [202]. Secondly, minimum E-values (expected values) were widely employed. The E-value estimates whether findings are robust to potential confounding. It measures the bidirectional cross-correlation required of some hypothetical unknown confounder variable with both the exposure of interest and the outcome of concern to explain an apparently causal effect [203,204,205,206,207]. Its 95% lower confidence bound is given by the minimum E-value (mEV). mEVs in excess of 1.25 are quoted in the literature as potentially indicating causal processes and hence were used as criteria in the current analysis. The mEV for the tobacco—lung cancer relationship is nine, which is described as being very high [208]. E-values were calculated for this study using the EValue R package [209].

### 2.7. Ethics

Ethical permission for this study was granted from the University of Western Australia Human Research Ethics Committee on 24 September 2021 with HREC Number 2019/RA/4/20/4724.

## 3. Results

The outline for the presentation of the Results Section is as follows:

3.1 Data
3.2 Bivariate Analysis
  3.2.1 Continuous
    Graphical
    Tabular analysis
    Bivariate conclusions
    Correlation analysis
    Mapping review
  3.2.2 Categorical
    Tabular analysis
    Graphical analysis
3.3 Multivariable panel regression analysis
  3.3.1 Additive
    Mixed-effects model
    Panel model—additive
  3.3.2 Interactive panel modelling
    No temporal lags (unlagged)
    Two-year temporal lags
    Four-year temporal lags
    Six-year temporal lags
  3.3.3 Multivariable conclusions

### 3.1. Data

Sources of data on organ-specific rates of cancer standardised to the world population of 1976 were obtained from the European Cancer Information System (ECIS) dataset and from the various national cancer registries and their online datasets, as listed in Supplementary Table S1. Raw data sources and processed input files and files used for the various analyses are also provided in the online Mendeley data repositories as mentioned in the Methods Section. A total of 47,920 age-adjusted rates were obtained from 24 nations and 130 regions. Other details relating to drug exposure, cancer type and income are shown in Supplementary Table S2. This table lists the sociodemographic and drug exposure datasets for both the bivariate and multivariable studies. It also provides the International Classification of Diseases Version 10 (ICD 10) Codes of interest for each cancer of concern.

Supplementary Figure S1 shows the rates of different groups of cancer across time. Most are shown to be stationary, some are rising and a few are falling. It is important to note in reading this figure that the ordinate axis is a log scale and so changes are arguably more marked than they appear. Imputed rates of substance exposure by country are shown in Supplementary Figure S2 with the data jittered to assist with illustration. The overall rates of tobacco use appear to be falling whilst the rates of other substances are variable. Interpolated rates for the various cannabis metrics are shown in Supplementary Figure S3. The four rates which were available were: (1) last month’s cannabis use (shown as LM. Cannabis in some figures), (2) daily or (3) near-daily cannabis use and (4) the THC content of cannabis herb and resin. In general, most metrics of cannabis use rose across this period with the notable exceptions of Poland, Denmark and Hungary, where rates of last month’s use declined, and Bulgaria, Luxembourg and Romania, where the THC content of cannabis also declined.

### 3.2. Bivariate Analysis

#### 3.2.1. Continuous Analysis

##### Graphical Analysis

Supplementary Figure S4 shows the time trend of 41 different cancers ordered by the declining slope of their regression curves with one panel for each cancer type. The rates of some cancers are rising, many are stationary, whilst a few decline. The ordinate scale is again logarithmic. In this figure, the notation “All Cancers nNMSC” (ACnNMSC) refers to all cancers with the exception of non-melanoma skin cancer.

**Figure 1:**
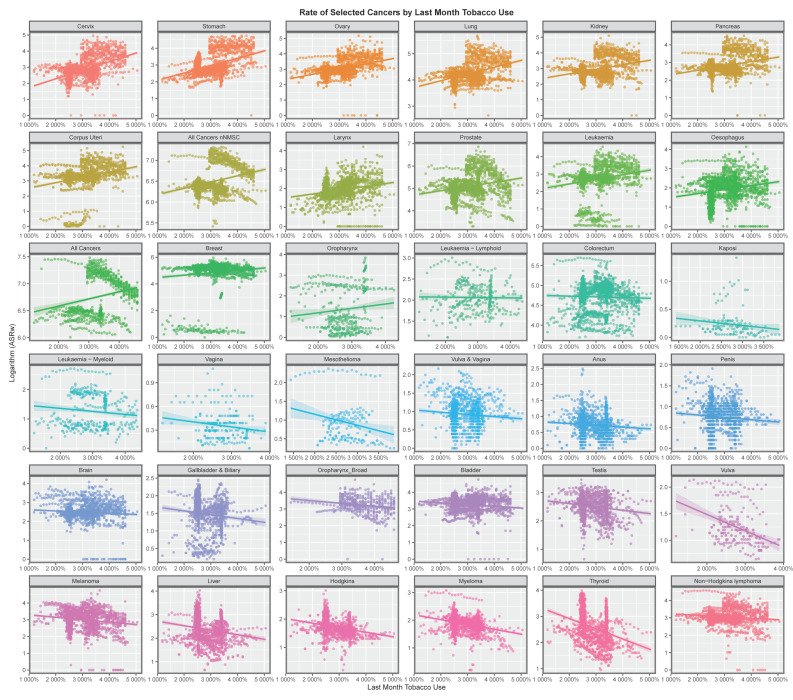
Rates of selected cancers by tobacco exposure.

Figure 1 shows the relationship of many cancers to tobacco exposure. The figure clearly shows that fifteen cancers are identified as being linked with tobacco exposure, all of which have previously been identified in epidemiological studies [210]. All cancers, ACnNMSC, lung cancer, larynx, cervical, oesophageal and cervical cancer are shown to be tobacco-related. This finding confirms this methodology as a way to look at cancer incidence from the real-world epidemiological data when controlled studies of risk exposure would not be possible.

**Figure 2:**
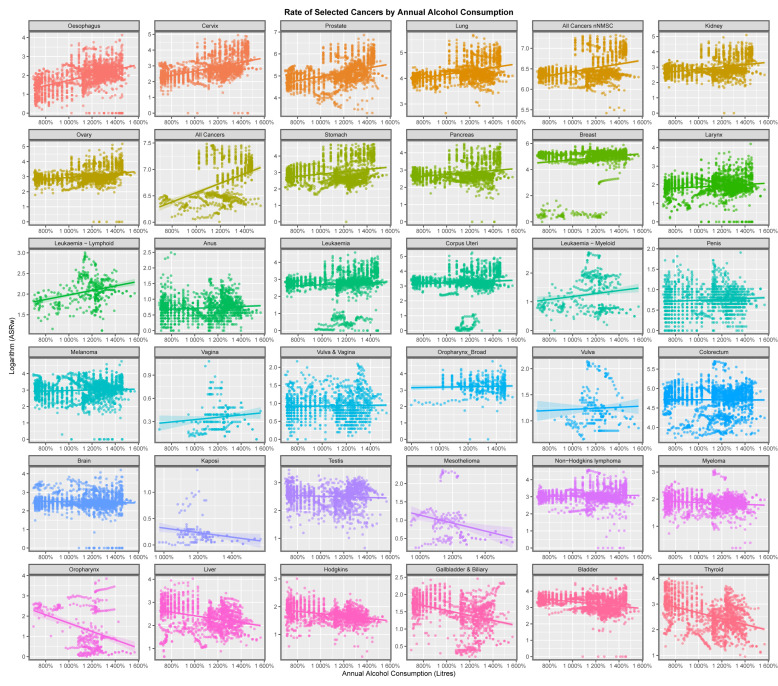
Rates of selected cancers by alcohol exposure.

Figure 2 plays a similar role for alcohol exposure. Again, the cancers seen to be rising here are well-known to be alcohol-related, a finding which further confirms the methodology. Again, all cancers, ACnNMSC, breast cancer, oesophageal cancer and chronic lymphoid leukaemia (CLL) appear to be alcohol-related.

**Figure 3:**
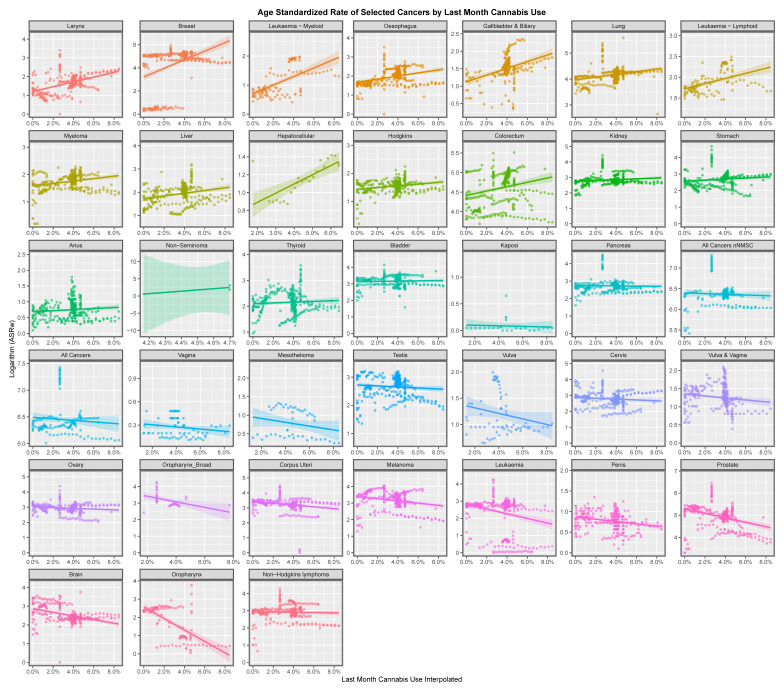
Rates of selected cancers by last month’s cannabis exposure.

**Figure 4:**
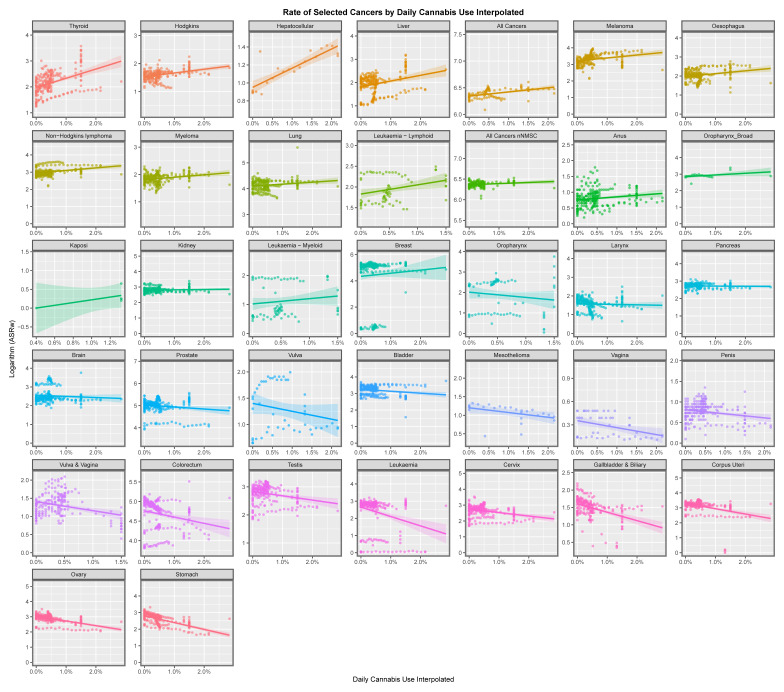
Rates of selected cancers by daily cannabis use interpolated.

Figure 3 plays a similar role for last month’s cannabis exposure. Cancers including hepatocellular cancer, laryngeal cancer, lung and breast cancer appear to be related to last month’s cannabis exposure. Figure 4 performs this role for daily cannabis exposure. Again, hepatocellular cancer and also thyroid, liver, non-Hodgkin’s lymphoma and breast cancer, amongst others, appear to be related to this exposure.

**Figure 5:**
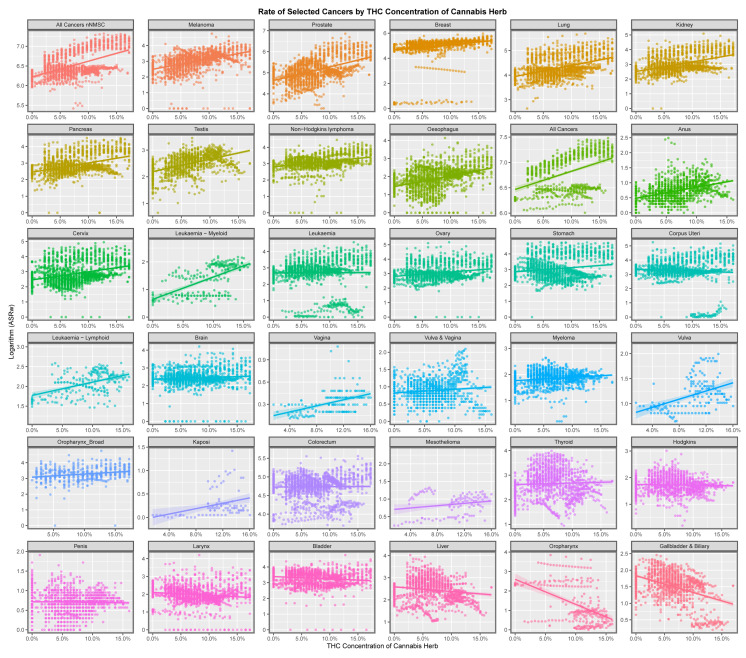
Rates of selected cancers by THC concentration of cannabis herb.

Figure 5 shows the cancer rates against the THC concentration of cannabis herb. Both all cancers and ACnNMSC are rising strongly against this metric, along with lung, kidney, pancreas, testis, cervical, oesophageal, lymphoid leukaemia, anal, vulva and Kaposi tumours.

**Figure 6:**
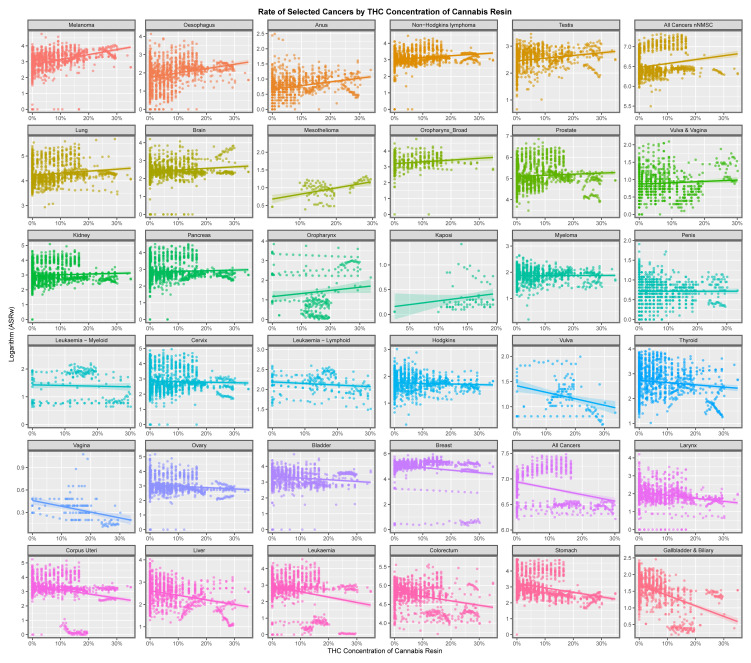
Rates of selected cancers by THC concentration of cannabis resin.

When rates of cannabis resin THC concentration is considered as the denominator, cancer rates appear to be less strongly associated with this metric (Figure 6).

When amphetamine exposure is considered, the strongest association appears to be with female genital tract cancers (Supplementary Figure S5). When cocaine is considered, several cancers appear to be associated with rising cocaine use (Supplementary Figure S6).

##### Tabular Analysis

The slopes of these regression trends and their statistical significance may be formally considered. A table of sequential model results from the purr–broom workflow in R is shown in Table 1 for tobacco exposure. These results confirm that 17 cancers are significantly related to tobacco exposure, confirming the appearances shown in Figure 1. This number drops to 14 after adjustment for multiple testing. In all cases, the minimum E-values are elevated above unity (1). Both all cancers and ACnNMSC have E-value estimates exceeding 1.25, which is considered to be the threshold for causal association [204]. When these data are studied by mixed-effects regression, only seven tumours are found to be significantly tobacco-related (Supplementary Table S3). Importantly, the E-value estimate for the tobacco–lung cancer relationship is noted near the top of this Table as 1.34, and its lower bound is 1.32, both of which exceed the threshold for causality.

When alcohol is considered in a mixed-effects model, 13 cancers are seen to be significantly related to alcohol exposure (Supplementary Table S4). When alcohol is considered in a series of linear regression models, 19 cancers are seen to be alcohol-related (Table 2). As shown, this number drops to 17 after adjustment for multiple testing.

When the various metrics of cannabis exposure were considered by mixed effects regression last month cannabis use was related to 10 cancers (Supplementary Table S5), daily cannabis use was related to 11 cancers (Supplementary Table S6), the THC content of cannabis herb was related to 21 cancers (Table 3), and the THC content of cannabis resin was related to 20 cancers (Table 4). These numbers drop to 6, 11, 21 and 13 after adjustment for multiple testing. From observations in the above paragraph it is ap-parent that linear regression detects more statistically significant signals than mixed effects regression. When the associations of cannabis herb THC concentration are studied by linear regression 31 positive cancers are significantly related, which drops to 29 cancers after multiple testing correction (Supplementary Table S7).

When amphetamine is studied by mixed-effects regression, it is noted to be related to only three cancers and this result is not affected by multiple testing adjustment (Supplementary Table S8). When the associations of cocaine are studied, it is apparently related to 18 cancers and this result also does not change after multiple testing adjustment (Supplementary Table S9).

We were also interested to observe if the interaction between cannabis herb THC concentration and daily cannabis use was also associated with tumour incidence. This interaction was similarly studied in nested mixed-effects models, and as shown in Supplementary Table S10, this was associated with 13 cancers, which reduced to 9 after multiple testing adjustment. Similarly, when the interaction between cannabis resin and daily cannabis use was studied, it was significant in ten cancers, declining to nine after multiple testing adjustment (Supplementary Table S11).

##### Bivariate Conclusions

Naturally, it was of interest to see how these different tumours performed across the various markers of cannabis use. Table 5 sets out the cancers significantly associated with the various indices of cannabis exposure for the four main bivariate mixed-effects models. As shown in the table, eight cancers appeared in all four mixed-effects models, and ten cancers appeared in three of them, making a total of eighteen cancers potentially implicated with cannabis exposure when similar analytical techniques to those for tobacco and alcohol were applied. Supplementary Table S12 performs a similar function by taking the level of significance as the multiple adjustment level of Holm. In this table, only oesophageal cancer is significant across all models and five other cancers appear in three of the four main models.

##### Correlation Analysis

It is of interest to consider the correlation between the different covariates and the most common cancers. As the data had some missing values, the correlation matrix was calculated in the R package WGCNA. The correlograms shown in Supplementary Figure S7 were drawn in the R package corrplot, which also indicates the various Pearson correlation coefficients. The significance levels of these correlation coefficients are shown quantitatively in Supplementary Figure S8 and semi-quantitatively in Supplementary Figure S9. Supplementary Table S13 sets out the correlation coefficients themselves; their *p* values are shown in Supplementary Table S14 and the numbers of observations upon which they are based are shown in Supplementary Table S15. Interestingly, the strongest correlation of interest shown is between daily cannabis use and cocaine of R = 0.7795, *p* = 4.40 × 10^−50^ and is based on 249 observations. The correlation between last month’s cannabis use and lung cancer is 0.44, which is associated with a *p* value of 7.61 × 10^−18^ and is based on 345 observations.

##### Mapping Analysis

**Figure 7:**
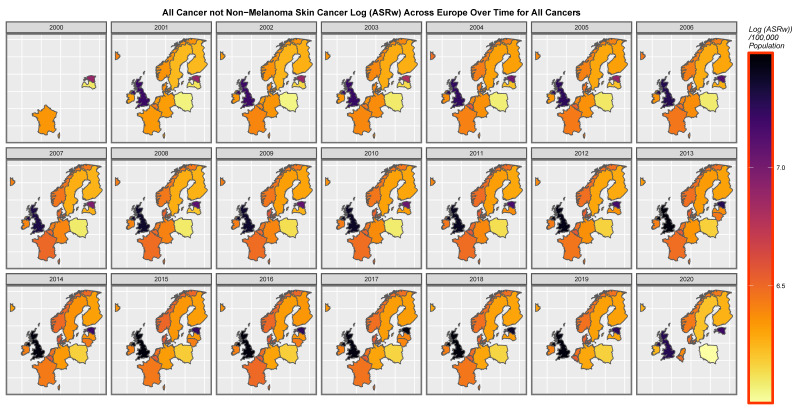
Rates of all cancers but not non-melanoma skin cancer across Europe 2000–2020.

**Figure 8:**
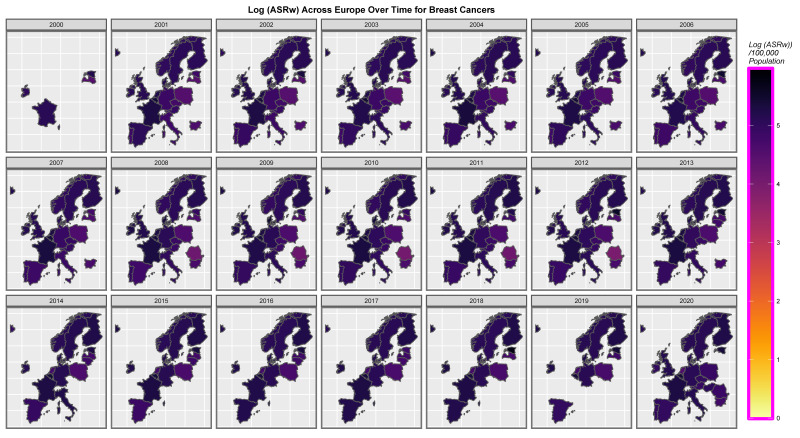
Rates of breast cancer across Europe 2000–2020.

It is of interest to consider the distribution of cancer across space and time. Supplementary Figure S10 sets this out for all cancers. High levels are noted across time in both the United Kingdom and Estonia. Figure 7 presents a similar series of maps for ACnNMSC and observes a similar pattern. Rates in the low countries such as Norway and Denmark are intermediate between those of the nations where cancer is more common and lower incidence countries such as Poland and Lithuania. Figure 8 shows a similar plot of breast cancer across the continent. Rates appear to be uniformly elevated across both time and space.

Supplementary Figure S11 presents a map graph of the THC concentration of cannabis herb. High levels are noted in Spain, Netherlands and Estonia, with intermediate levels in France and Germany.

It is possible to consider the cooccurrence of two different covariates across time and space. Supplementary Figure S12 sets this out for the rates of all cancers and cannabis herb THC concentration. In this map, the green areas denote zones in which both covariates are low whilst the pink and purple areas indicate zones where both covariates are elevated. On this map, Estonia stands out prominently as being a country with high levels of both total cancer and THC content of cannabis herb. Data for many other nations are absent.

**Figure 9:**
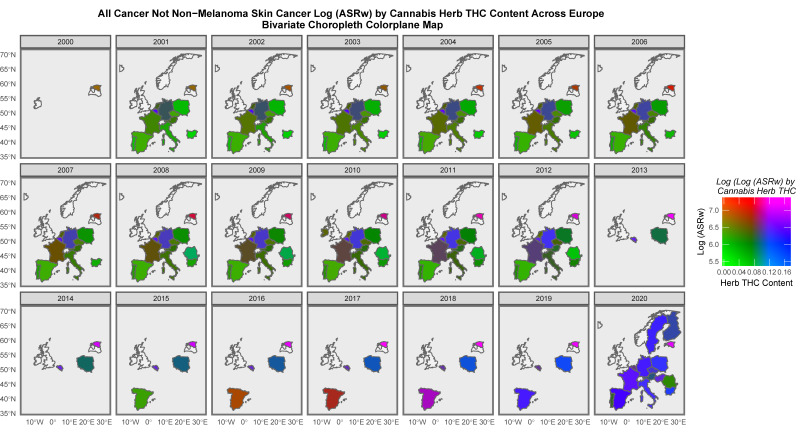
Bivariate map of all cancers but not non-melanoma skin cancer by cannabis herb THC concentration. Please see text for details.

When ACnNMSC is studied in a similar manner, the appearances shown in Figure 9 are seen. Estonia is still high, but here, France, Czechia and Hungary are noted to be shaded in purple.

**Figure 10:**
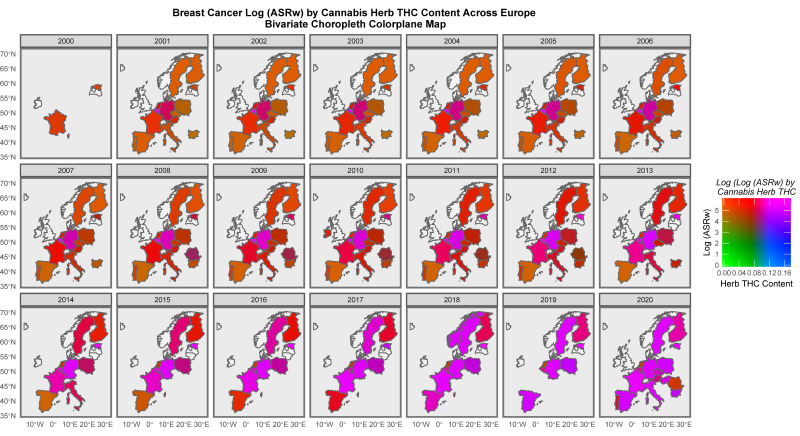
Bivariate map of breast cancer by cannabis herb THC concentration.

When breast cancer is analysed in a similar manner, most of the European continent is noted to be highlighted in pink where data are available (Figure 10).

**Figure 11:**
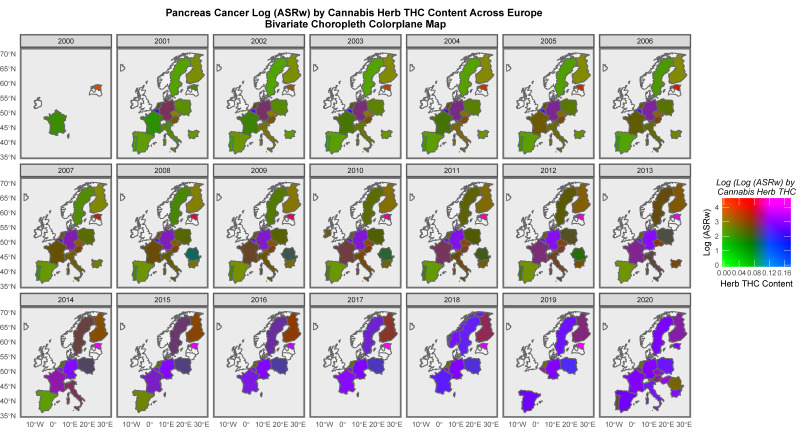
Bivariate map of pancreatic cancer by cannabis herb THC concentration. Please see text for details.

When liver cancer is studied, Spain, France and Italy are shaded in purple (Supplementary Figure S13). When the rates of pancreatic cancer are examined, many nations are noted to be shaded in purple (Figure 11). Considering prostate cancer, France, Czechia, Estonia and Finland are seen to be highlighted in purple or pink (Supplementary Figure S14). When colorectal cancer is considered, Spain, France, Czechia, Estonia and Hungary are all highlighted (Supplementary Figure S15). France, Hungary, Germany, Estonia and the Netherlands are highlighted when lung cancer is considered (Supplementary Figure S16). Most of Europe is shaded in purple in Supplementary Figure S17 when non-Hodgkin’s lymphoma is similarly considered. Lymphoid leukaemia (chronic lymphatic leukaemia) is highlighted in Estonia, Germany, France, Czech Republic, and Hungary (Supplementary Figure S18). Vulval carcinoma is corelated with cannabis herb THC concentration in Germany, the Netherlands and Czechia as shown in Supplementary Figure S19.

#### 3.2.2. Categorical Analysis

##### Tabular Analysis

As shown in Supplementary Table S16, the countries involved in this study may be divided into nations with higher compared to lower levels of tobacco use. The top ten nations for average tobacco use across this period are Bulgaria, Austria, Latvia, Estonia, Lithuania, France, Spain, Czech Republic, Sweden and Hungary. These nations therefore may be grouped as relatively high-tobacco-using countries compared with cancer rates in the other countries. The age-standardised rates in each country were multiplied by the population of that country for each of the years concerned to generate estimates of the numbers of the cases of each cancer in the higher and lower tobacco-using groups, respectively, as shown in Supplementary Table S17. This Table also shows applicable *p*-values and E-values. The *p*-values shown are often very low (in R a *p*-value < 10^−307^ is considered to be zero) and the E-values range up to 6.67 for oropharyngeal cancer. Both the E-value and its lower bound for the lung cancer–tobacco relationship by this method are 1.24. From these data, the relative risk incidences, attributable fractions in the exposed (AFE) and population-attributable risks (PAR) (also known as the attributable fraction in the population) may be calculated for higher levels of tobacco exposure, as shown in Table 6. The Table is headed by oropharyngeal cancer with an RR of 3.63 (95% C.I. 3.60–3.65), AFE of 72.43% (72.24%–72.61%) and PAR of 50.43% (50.19%–50.66%).

A similar exercise may be performed for alcohol. In this case, the top ten nations for alcohol consumption across this period, defined by their mean annual alcohol consumption based on study data, summarised in Supplementary Table S18 and used to derive the cases in the highly exposed and less-exposed groups, are shown in Supplementary Table S19. Once again, broadly defined upper airways carcinoma (also called head and neck cancer) leads in this table with a *p*-value of zero and an E-value estimate of 4.20. Once again, elevated relative risk ratios, AFEs and PARs can be calculated (Supplementary Table S20). The high results, which are again seen (RR = 2.38 (2.35–2.41), AFE = 58.05% (57.48–58.60%) and PAR (56.61% (56.04–57.16%)), are consistent with the known causal role of both tobacco and alcohol in upper aerodigestive tract cancerogenesis.

Supplementary Table S21 sets out the rate of cannabis use across the nations in this study by the four main metrics of cannabis use. Using such information in addition to that of major epidemiological reports recently produced on the subject, it was possible to denote Belgium, Netherlands, France, Germany, Ireland, Italy, Estonia, Norway, Portugal and Spain as high-risk nations and the others as lower risk countries.

With the nations grouped in this way, it was again possible to calculate numbers in the more highly exposed countries (Supplementary Table S22) and their applicable RR, AFE and PAR ratios (Table 7). These tables are led by Kaposi sarcoma and liver and thyroid cancers with *p*-values, E-value estimates, RRs, AFEs and PARs of: Kaposi sarcoma *p* = 1.86 × 10^−170^, E.est. = 3.58, RR = 2.08 (1.98–2.19), AFE = 51.95% (49.34–25.42%) and PAR = 25.73% (23.76–27.65%); liver cancer *p* = zero, E.est. = 2.92, RR = 1.76 (1.76–1.77), AFE = 43.27% (43.04–43.50%) and PAR = 40.77% (40.55–40.99%); thyroid cancer *p* = zero, E.est. = 2.77, RR = 1.69 (1.68–1.70), AFE = 40.90% (40.69–41.11%) and PAR = 38.50% (38.30–38.71%).

##### Graphical Analysis

**Figure 12:**
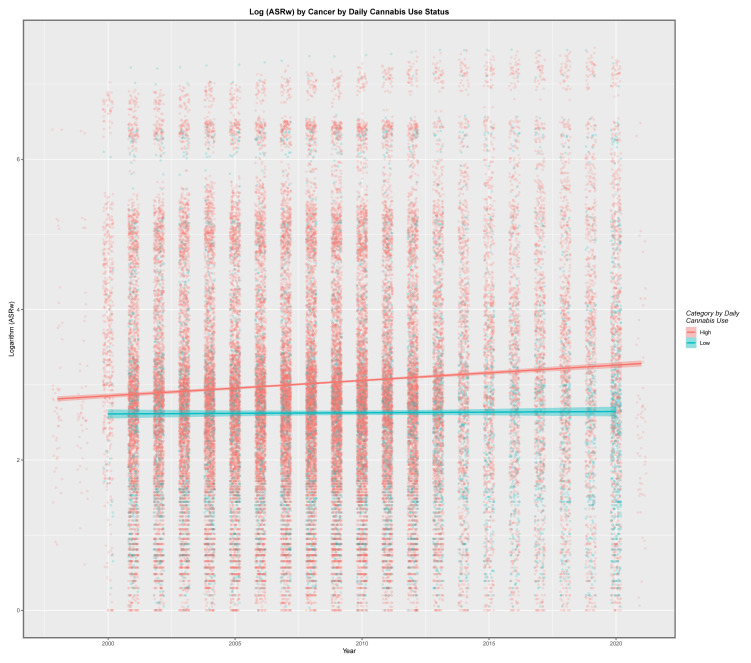
Logarithm ASRw rates in high-cannabis-using countries compared to low-cannabis-using countries. Please see text for details.

Moreover, the cancer rates across all tumour types in high-cannabis-using countries may be contrasted with those in nations with lower rates as shown in Figure 12, where the log age-standardised cancer rates across all tumours appear to be markedly higher than those in lower-cannabis-use countries. When these data were considered by linear regression in an additive model with time, the higher status group demonstrated significantly elevated cancer rates compared to those with lower use rates (β-estimate = 0.4161, t = 22.9, *p* = 3.54 × 10^−115^; model Adj. R. Squ. = 0.0125; F = 319 on df = 2, 50,175, *p* < 2.2 × 10^−16^). When an interactive linear model was again considered, the result was also highly significant both for the high-status group (β-estimate = −37.4445, t = −5.60, *p* = 2.18 × 10^−8^; model Adj. R. Squ. = 0.0131; F = 224 on df = 3, 501,754 *p* < 2.2 × 10^−16^) and for the time: status interaction (β-estimate = 0.01884, t = 5.66, *p* = 1.52 × 10^−8^). When the data were considered by mixed-effects regression in an additive model with time, with region as a random effect, the effect of exposure group designation was again highly significant (β-estimate = 0.1541, t = 5.55, *p* = 2.74 × 10^−8^; model AIC = 176,315, BIC = 176,360, log.Lik ratio = 88,152.7).

These data may also be considered in a tumour-specific manner as shown in Supplementary Figure S20. Interestingly, the regression lines for common tumours such as all cancers, ACnNMSC, breast cancer, colorectal cancer, laryngeal cancer and thyroid cancer are significantly above those for the lower-cannabis-using countries. The pattern for other tumours is the inverse of this. Aggregated across time, the boxplots generated in Supplementary Figure S21 may be shown. These graphs are read by noting where the notches on the boxes do not overlap between groups. The extent of the failure of overlap is a measure of the statistical significance of between group differences. The cancers are panelled alphabetically, which makes finding a tumour of interest straightforward. It is immediately apparent that the notches for all cancers, ACnNMSC, breast, thyroid, lymphoid leukaemia, liver cancer and many others are widely separated. Supplementary Figure S22 illustrates the same data, but this time ordered in descending order of the ratio of the cancer rates in the high- to the low-cannabis-using countries.

### 3.3. Multivariable Regression Analysis

#### 3.3.1. Additive

##### Mixed-Effects Model

A multivariable mixed-effects model was next considered, which examines the relative contribution of the various covariates to the tumour-specific cancer rates. The model was an additive mixed-effects model with terms for tobacco, alcohol, last month’s cannabis use, median household income and the mean THC concentration of cannabis herb and resin. The random effect was assigned to country and region. The full output from the model is shown in Supplementary Table S23. The Table is headed by ACnNMSC and liver cancer with *p*-values of 1.52 × 10^−11^ and 3.50 × 10^−17^ and E-values of 6.30 × 10^126^ and 7.94 × 10^73^, respectively. Terms from this model, which are positive and significant, are extracted as Supplementary Table S24. Correction for multiple testing has also been included in the tabulation of results. Fifty-four terms were extracted in this way. These terms may be summarised as shown in Supplementary Table S25, which shows the number of cancers implicated, and the sum, mean and median of the (negative) *p*-value exponents and, similarly, the sum, mean and median of the minimum E-value exponents.

The main findings from this table are illustrated graphically in Supplementary Figure S23, which shows the number of cancers, the sum of the (negative) exponents of the *p*-values and the total and mean minimum E-values in panels A–D, respectively. It is clear from this figure that the concentration of cannabis herb and last month’s cannabis occupy the highest position on all four graphs. It is noted that the ordinate scale in the lower two panels is a logarithmic scale, which amplifies the differences shown.

##### Panel Model—Additive

**Figure 13:**
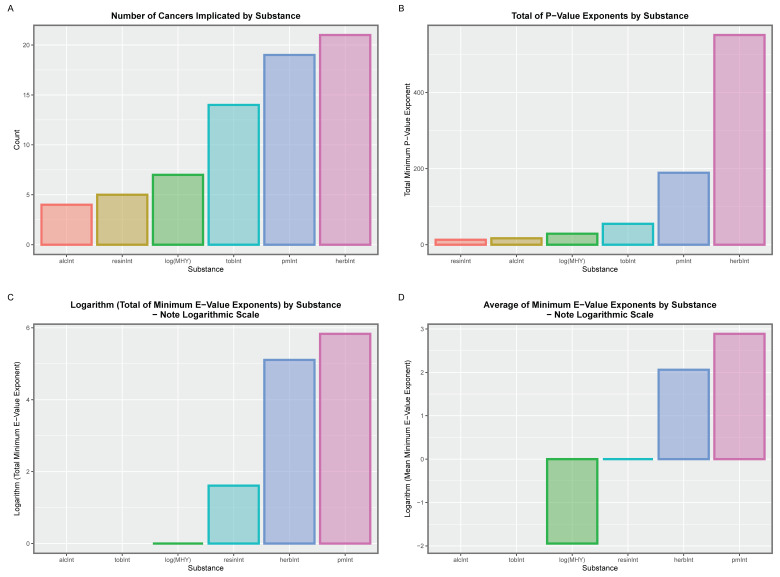
Graphical summary of additive panel model. (**A**) number of cancers implicated by substance, (**B**) Totals of (negative) *p*-value exponents by substance, (**C**) Logarithm (total of minimum E-Value Exponents) by substance—note logarithmic scale and (**D**) average of minimum E-value exponents by substance—note logarithmic scale.

This same model was studied by panel regression techniques as panel techniques can be used to study temporal lagging, which are not available with mixed-effects models. For this reason, a similar model was studied by panel techniques. The output from this model is shown in Supplementary Table S27. From this exercise, 70 positive and significant terms were extracted and are shown in Table 8. These are summarised in Table 9 and illustrated graphically in Figure 13. Once again, the indices of cannabis use appear on the right-hand side of these graphs for numbers of tumours implicated and the cumulative indices of *p*- and E- values.

#### 3.3.2. Interactive Panel Modelling

##### No Temporal Lags (Unlagged)

**Figure 14:**
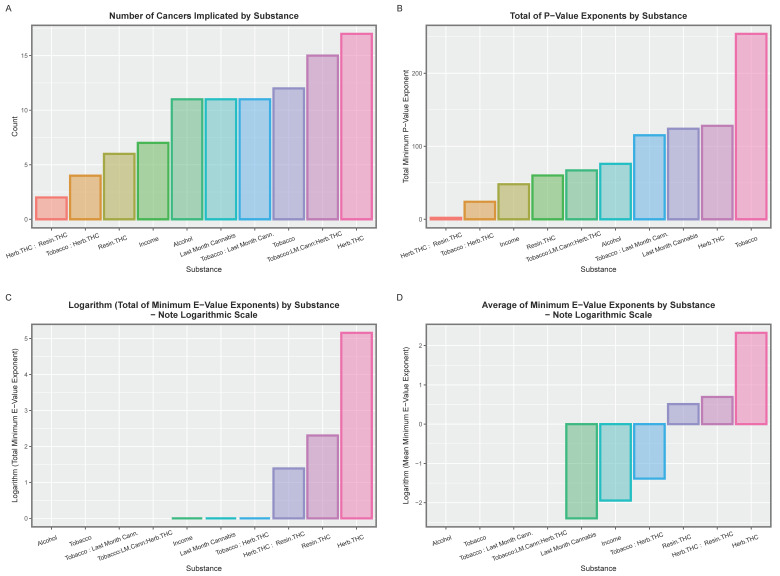
Graphical summary of interactive panel model. (**A**) number of cancers implicated by substance, (**B**) Totals of (negative) *p*-value exponents by substance, (**C**) Logarithm (total of minimum E-Value Exponents) by substance—note logarithmic scale and (**D**) average of minimum E-value exponents by substance—note logarithmic scale.

A three-way interaction term was introduced between tobacco use, last month’s cannabis use and the THC concentration of cannabis herb into the above additive model. The output from this model is shown as Supplementary Table S27. Significant terms are extracted (Table 10) and summarised in tabular (Table 11) and graphical (Figure 14) formats. Table 10 is ordered by descending minimum E-value. It is clear from this table that cannabis terms dominate the top of this table and tobacco terms are near the bottom. These findings are reflected in the tabular and graphical summaries provided (Table 11 and Figure 14), which again show that the effect of terms, including cannabis, are much more potent than the known carcinogens tobacco and alcohol.

##### Two-Year Temporal Lags

This modelling procedure was repeated at two years of temporal lags. Model output appears as Supplementary Table S28 and the reduced tabulation consisting of significant positive terms appears as Supplementary Table S29. The terms of Supplementary Table S29 are then summarised in Supplementary Table S30 and displayed graphically in Supplementary Figure S24. It is again noted that the cannabis terms preponderate over tobacco, alcohol and income terms in all four panels.

##### Four-Year Temporal Lags

The above-described interactive panel model was run at four years of temporal lag. Full model outputs are shown in Supplementary Table S31, the reduced model with positive significant terms is shown in Supplementary Table S32 and the summary of this model appears in Supplementary Table S33 and Supplementary Figure S25. From Supplementary Figure S25, it is clear that the sum of the negative *p*-value exponents is greater for tobacco than for the other covariates. However, for the other three metrics, it is clear that the impact of the measures of cannabis predominate.

##### Six-Year Temporal Lags

A similar exercise was conducted at six years of temporal lags. Interactive panel model output appears as Supplementary Table S34, positive and significant terms are shown in Supplementary Table S35 and these are summarised by term in Supplementary Table S36 and Supplementary Figure S26. Cannabis-related terms again predominate in all four panels. For both the numbers of cancers implicated and the total of the negative *p*-value exponents, tobacco comes in second place for terms related to cannabis exposure.

#### 3.3.3. Multivariable Conclusions

The above results demonstrate that in these fixed-effects and panel multivariable regression models, the impact of cannabis is greater than that of the other covariates. A major remaining issue is how each of the different cancers assessed performed across the various models. This issue is addressed in Table 12, which sets out the six different multivariable models and considers only those cancers which were shown to be significant after adjustment for multiple testing (by the Holm’s method).

As shown in this Table, 17 groups of cancers were related to the metrics of cannabis exposure on all multivariable models used, including: all cancers, ACnNMSC and cancers of the: bladder, breast, colorectum, Hodgkin’s disease, kidney, larynx, melanoma, myeloma, non-Hodgkin’s lymphoma, oesophagus, ovary, pancreas, prostate, stomach and thyroid. In five of the six models, cancers of the brain, cervix uteri, liver, lung and testis also tested positive to stringent multiple testing adjustment. Oropharyngeal cancer should probably also be included in this group, as it was included in four models when strictly defined and in four models when broadly defined as cancer of the upper aerodigestive tract.

Notable amongst this list was several cancers of the reproductive tract, including the germinal cells of the testis and ovary and also the prostate and breast.

## 4. Discussion

### 4.1. Main Results and Interpretation

The main results of this study reveal that cannabis is indeed related to the incidence of many cancers in both bivariate- and multivariable-adjusted models and strongly confirm results, which have been previously described elsewhere, particularly in the USA [17,18,44,167,168,169,211,212,213,214,215].

In most comparisons with tobacco and alcohol, cannabis was a much more potent carcinogen, particularly when metrics relating to E-values were considered. Moreover, the unequivocal involvement of tumours of the reproductive tract, including the testis, ovary, breast and prostate, along with various leukaemias—some of which occur in childhood—all point to clinically significant heritable genotoxicity impacting subsequent generations.

Therefore, the answer to the four questions posed in the Introduction were all affirmative. Cannabis was confirmed to be an important human carcinogen, and results similar to those reported elsewhere in terms of the tumours implicated were identified; cannabis has again been shown to be a more potent carcinogen than tobacco or alcohol and evidence of reproductive and inheritable genotoxicity and carcinogenicity has again been confirmed.

This paper presents strong evidence that cannabis exposure is linked to the incidence of many cancers in Europe using bivariate analysis and that these changes were actually increased by multivariate adjustment. The slope of many cancer incidence–substance exposure trend lines at bivariate analysis is obviously more strongly positive for metrics of cannabis exposure than with tobacco and alcohol (Figure 1, Figure 2, Figure 3, Figure 4, Figure 5 and Figure 6) and these appearances are confirmed by quantitative bivariate and multivariate analyses (Table 1, Table 2, Table 3, Table 4, Table 6, Table 7, Table 8, Table 9, Table 10, Table 11 and Table 12 and Supplementary Tables S33–S36) and mathematical collations of these data (Table 9 and Table 11, Supplementary Tables S33 and S36). Findings are robust to different regression algorithms used, with similar results being obtained from both mixed-effects and panel regression models (Supplementary Table S24 and Table 8).

### 4.2. Cannabis-Linked Cancers

The question of which cancers should be considered to be linked with cannabis exposure emerges from this study. This issue should be considered in relation to tumours considered to be tobacco- and alcohol-related, where the significant slope of the linear regression trend line and its associated metrics (RR, AFE and PAR at categorical analysis) seems to be the major issue deciding the matter. The 22 tumours identified at multivariable regression should be included in this list of malignant disorders. All of the tumours identified on bivariate regression were also identified on multivariable panel regression. Whilst oropharyngeal cancer (defined both locally to the oropharynx and more broadly across the upper aerodigestive tract) was noted to be associated in four of the six multivariable models, it was also noted in all of the linear models and in two of the linear models (for cannabis herb and resin THC concentration) after adjustment for multiple testing (Table 5 and Supplementary Table S12).

Given that both acute myeloid and lymphoid leukaemias have been previously identified as being cannabis-associated [35,36,212,216], an interesting question relates to the findings of this study in relation to this group of diseases. Lymphoid leukaemia was identified in two bivariate models and three multivariable models. Myeloid leukaemia was identified in three linear models and two multivariable models. For lymphoid leukaemia, its minimal E-values were 4.42 × 10^14^ and 51.55 at linear regression (Supplementary Table S5 and Table 3) and 2.67 × 10^−22^, 2.67 × 10^22^ and 7.90 × 10^4^, respectively, on multivariable testing (Supplementary Tables S24 and S28 and Table 8). Based on these results and the above-cited salience of the bivariate relationships in epidemiological studies, these leukaemias should be included in the list of cannabis-related cancers. Unfortunately, detailed information on acute lymphoid leukaemia and acute myeloid leukaemia was not available to the present researchers and this study must await a subsequent investigation.

Brain cancer was identified on two linear models and four multivariable models. Its minimal E-values on bivariate testing were 19.93 and 5.20 (Table 3 and Table 4) and on multivariable testing were 9.00, 2.09, 1.62, 2.18 and 276.21 (Table 8, Table 10 and Table 12 and Supplementary Table S28). Irrespective of the pathways of neurocarcinogenesis, this fits with modern studies, which clearly demonstrate that an altered brain neurotransmission is a direct stimulant to the growth of both primary [217,218] and secondary [219] intracerebral tumours. Given the well-known high density of intracerebral cannabinoid receptors and the established neuroactivity of a diverse range of phytocannabinoids, this would fit with the cannabinoid-modulated neurotransmission potentiation of a pathway to neuroglial tumourigenesis. Indeed, brain cancer has previously been linked with cannabis exposure [23,220].

Thus, the final list of the 25 cancer types related to cannabis in this study is: all cancers, ACnNMSC, bladder, brain, breast, cervix, colorectum, Hodgkin’s, kidney, larynx, myeloid and lymphoid leukaemias, liver, lung, melanoma, myeloma, non-Hodgkin’s lymphoma, oesophagus, oropharyngeal tumours both broadly and narrowly defined, ovary, pancreas, prostate, stomach, testis and thyroid cancer.

### 4.3. Specific Cancers

Some of the bivariate space–time relationships described graphically in maps with the THC concentrations of cannabis herb for cancers, such as all cancers, excluding non-melanoma skin cancer (ACnNMSC), and cancers of the breast, liver, pancreas, lung and non-Hodgkin’s lymphoma (Figure 12, Figure 13 and Figure 14), are particularly striking. Indeed, in the case of breast cancer, the map series shows that the whole of Europe was transformed from red-brown (low cannabis and moderate-to-high incidences of breast cancer) to the whole continent being shaded in pink to purple (both cannabis and breast cancer high, respectively) as both breast cancer (Supplementary Figure S1) and cannabis exposure (Supplementary Figure S1 and Figure 11) rates rose across the decades of this study (Figure 13). Far from this being a mere circumstantial association, the existence of a host of cellular, biological and epigenomic mechanisms (mentioned in both the Introduction and below)—together with the mathematical and statistical methodologies employed throughout this study using the classical tools of causal inference, as well as a strong corroboration from parallel findings in the USA [44,167,168,169,211]—clearly point to the causal nature of this relationship. Given that breast cancer is the most common form of cancer in many nations, this is a signal finding indeed and supports the highly salient remarks above in relation to the rates of all cancers and ACnNMSC. Importantly, this European trend linking the rise in breast cancer to increasing cannabis use has also been recently confirmed in the USA in a space–time and formal causal inferential paradigm [211].

The observations relating to liver cancer (Figure 14) are also highly salient, given that the incidence of this tumour is growing quickly around the world [18,215,221,222,223,224]. Whilst viral causes in the context of international migration trends are usually invoked by way of explanation for this trend, the current data suggest that cannabis may well be a significant albeit usually overlooked environmental risk factor in this aetiological complex [222,223] as has been previously observed [18,21,215,225].

Similarly, a recent study covering 65% of the USA from Cedar Mt. Sinai Cancer Centre found that the incidence of pancreatic cancer is rising across the USA [226]. The rise is most marked among young women, particularly amongst African Americans. Whereas the annual adjusted incidence rate of pancreatic cancer in men younger than 55 years is increasing by 0.62% annually, it is rising almost four times as fast in young women in this age bracket at 2.36%. In patients from 15 to 34 years of age, the disease was increasing exponentially in both sexes. In females, the rate of annual average percentage (AAPC) rise was a remarkable 6.45% (5.36–7.55%) and 2.97% (1.69–4.27%) in males. The use of cannabis by young females is also rising rapidly, including during pregnancy. Based upon these and similar results, it would appear that cannabis use is an important community cancerogenic risk factor, which has likely been overlooked in public health discussions to the time of writing [227,228].

It is also of interest that cannabis has recently been shown to drive the 50% increase in total paediatric cancer in the USA [41] and the doubling in the commonest cancer of childhood acute lymphoid leukaemia in USA [212]. Cannabis was also shown to be the primary driver of the doubling of testicular cancer rates in young men since 1975 [17,214].

Multivariable models lagged to two, four and six years were also studied. In general, the effect of temporal lagging was to increase the effects described in non-lagged models.

### 4.4. Reproductive Cancers

The present results implicate testicular and prostate cancers in males, and breast, ovarian, uterine and cervix cancers in females. Indeed, the involvement of a number of cancers of the reproductive tract in both sexes is noteworthy and of great concern for the multigenerational passage of genotoxic and/or epigenotoxic damage. The concept of heritable mutagenesis is also supported by the identification of leukaemias of various types in this analysis. Both myeloid and lymphoid leukaemias can occur in childhood, and indeed, acute lymphoid leukaemia is the most common form of cancer for children under five years of age, accounting for around 25% of the tumours in toddlers under five.

### 4.5. Cannabis Herb THC Concentration

It was somewhat surprising to us that the main cannabis-related covariate, which was most strongly related to malignant outcomes, was the THC concentration of cannabis herb, as shown in many tables. However, as cannabis herb is likely much more widely available and used than other cannabis products, it does make sense in a real-world application that this would be a primarily important metric of cannabis exposure. It has been suggested that it is the convergence of cannabis use prevalence, the intensity of use and cannabis herb and resin concentration, which is of the greatest concern [110], along with this important exploration of the most incisive cannabis-related metric, merits further investigation by subsequent research.

### 4.6. Comparison with USA Data

The other major dataset which is available for a similar comparison is the USA dataset, which also contains information on tumour type and drug-use exposures. Published reports, which performed similar analyses on the USA data to those described here, have only recently begun to appear in the medical literature of the USA data. One recent paper did find that breast, liver, thyroid and pancreatic cancer and acute myeloid leukaemia were elevated in the USA in relation to cannabis exposure. Another paper found acute lymphoid leukaemia to be elevated in association with community cannabis exposure [212]. Since this is the most common form of early childhood cancer, it is perhaps to be expected that cannabis was found to be a driver of rising paediatric cancer rates across the USA [212]. Importantly, breast, thyroid, liver and pancreatic cancers were all positively identified in the present study in the categorical analysis of cannabis herb (Table 7).

Hence, there is good agreement between these two major datasets, which together are understood to comprise the majority of the extant publicly available data in the world at this time. This further supports our confidence in the present study conclusions.

The present results are also supported by recent analyses of the USA data [167,168,169]. Importantly, breast cancer was recently also linked with cannabis exposure in the USA.

### 4.7. Causality

There are many medical questions which would be difficult, expensive or time-consuming to investigate by formal randomised controlled trials, which are not always timely, possible or even ethical. Since many such contexts exist in medicine, it is often important to maximise the opportunity presented by real-world pseudo-experimental situations, which present themselves [3] and which can meaningfully inform policy-makers before multiple-outcome randomised clinical trials can be organised [229].

Two of the major limitations which commonly plague observational studies are the issue of non-comparability between groups and the related issue of unmeasured uncontrolled confounding covariates. It has been well-shown that inverse-probability weighting (IPW), when applied to an observational study, can transform its findings from a merely situational and local account by simply applying to that dataset into a pseudo-randomised study from which causal inferences can be meaningfully drawn [230,231]. IPW has been extensively applied to all multivariable panel models in the present analysis in order to avail ourselves of its profound advantages.

Similarly, given an apparently or potentially causal association, it is possible to quantify the degree of association required between *both* an exposure of interest and an outcome of concern in order to obviate an apparently causal relationship. This value is known as the E-value or expected value and has been computed in several of our tables. Similarly, its 95% lower bound (the minimum E-value, mEV) can also be calculated and sets a lower bound on this confidence interval. The many highly elevated mEV’s shown in our tables provide a strong reassurance in these respects.

In this context, it is worth reviewing briefly the way these data intersect with the qualitative causal criteria proposed in 1965 by Hill, which arose from the decade-long debate on the nature of the tobacco–lung cancer relationship [232]. The present results demonstrate a strong strength of association, consistency amongst studies, specificity (the group of cannabis-related cancers is not identical to the group of tobacco- or alcohol-related cancers), temporality, coherence with known data from elsewhere and in the laboratory, biological plausibility, a dose–response biological gradient, analogy with similar situations elsewhere and experimental confirmation.

For these reasons, our results may properly be considered to fulfil both quantitative and qualitative epidemiological criteria for causality. We feel that these provocative results strongly indicate on-going research to further explore mechanistic links experimentally in the identified tumours.

### 4.8. Specific Cannabinoids

Our present understanding is that data relating to community exposure to various individual cannabinoids is not generally available in Europe. Hence, we are not able to comment from this dataset on the relative genotoxicity of the many diverse cannabinoid compounds. It is clearly seen in the nature of the above results that THC itself is generally and broadly implicated in all results for cannabis herb and resin. However, this finding by no means exonerates other cannabinoids for which no readily accessible metric exists.

It is, however, pertinent in this respect that in the USA, THC, cannabinol, cannabigerol and cannabidiol have all been implicated in carcinogenic environmental exposure by recent epidemiological studies [44,48,212]. Since it is the central cannabinoid nucleus, known as olevitol, which is a dihydroxylated benzene ring on the C-ring of cannabinoids, which has been implicated in genotoxic cellular actions [132,233,234,235,236], it seems likely that the carcinogenic effect is actually a class effect shared across many or most cannabinoids. This is certainly the conclusion to which published and unpublished epidemiological data clearly points.

### 4.9. Mechanisms

Since the time of Bradford Hill and the tobacco–lung cancer debates of the 1950s, the centrality of biologically plausible mechanistic pathways to establish a conceptual mechanistic link between an exposure of concern and a pathology of interest has been central to any discussion of potentially causal mechanisms. A wide variety of cannabinoids have been implicated in multiple cellular and molecular pathogenic mechanisms. The issue is complex and has been reviewed in detail elsewhere [63,65,154,155,157,237,238,239,240,241,242].

In summary, it may be considered that: cannabis smoke includes all of the tars and other carcinogens found in tobacco smoke [33,34,243,244,245]; cannabis has well-described effects dramatically increasing the mitotic and meiotic division error rates in both sperm and oocytes [236,246,247]; cannabis, Δ9-tetrahydrocannabinol (THC), cannabidiol, cannabinol and cannabichromene have classically described major toxic effects on chromosomes [241,246,248] (including single- and double-stranded chromosomal breaks [241,246,248,249,250,251] and chromosomal ring and chain formation [241,246]) with chromosomal shattering “chromothripsis” [65], which is a major engine driving the genetic chaos of cancer [65,252,253,254,255,256,257,258,259,260,261,262,263,264], with the oxidation of DNA nucleosides and thus, direct genotoxicity and mutagenicity [248], with major changes of DNA methylation [137,153,154,155,156,157,158], which can be passed to subsequent generations [137,157] and which have also been identified in human sperm [137,157]; with a reduction in the gross levels of histone synthesis, which is a pro-oncogenic change that necessarily opens up chromatin for dysregulated transcription [265,266,267]; and several cannabinoids have well-established multiple adverse consequences on mitochondrial metabolism [75,82,268,269] (including a reduced synthesis of the F1-ATPase [270]), which have direct (via epigenomic substrate supply) and indirect (via mitonuclear shuttles and metabolic crosstalk pathways) genomic and epigenomic impacts [164,271].

Cannabinoids also reduce tubulin synthesis [270]. Long polymers of tubulin form the microtubules of the mitotic spindle, along which chromosomes slide during the chromosomal separation of anaphase, and their disruption directly causes chromosomes to become dislocated, therefore leading to micronucleus formation [65,247,256,272]. Microtubules also form the spine of the sperm tail, and disruptions of the post-translational modifications of this sophisticated “tubulin code” have been linked with highly aberrant sperm motility as sperms swim around in circles and are not able to move progressively towards a normal fertilisation target [273].

### 4.10. Carcinoma of the Testis

Since testicular cancer, including cancer of the testis, was strongly identified in the present data, and since testicular cancer is the best validated of all cannabis-related cancers [4,5,6,7,10], some detailed consideration of the genetic, epigenetic and chromosomal malignant biogenesis of this tumour is both of interest and of relevance.

### 4.11. Structural Observations

A total of 42% of genomes were involved.

It has been shown that testicular cancer predictably displays an isochromosome 12 together with gains of chromosomes 7, 8, 12, 21 and X along with losses of chromosomes 1, 11, 13,18 and Y [274]. Human genome project studies show that chromosomes 1, 7, 8, 11, 12, 13, 18, 21, X and Y have lengths of 246, 158, 146, 134, 132, 113, 76, 46, 153 and 50 megabases, respectively, implying a total chromosomal length of 1,254 megabases or 42% of the human genome of 3000 megabases [275].

### 4.12. Mechanistic Observations

A 6.5-fold acceleration of the incidence–oncogenic induction period.

The increased frequency of testicular cancer following cannabis exposure documented in metanalysis at 2.6-fold was noted above [10]. As described above, testicular cancer is noted to generally develop over 33 years and is based on an the activation of genotoxic insults by the hormonal surge of puberty [8,9,11,14,274]. However, if one accepts a median age of cannabis exposure of 20 years, then it follows that testicular cancer develops following cannabis exposure over only 13 years, which represents a 2.5-fold acceleration in the pre-oncogenic induction time from 33 to only 13 years. The 2.6-fold increase in incidence and the 2.5-fold acceleration of oncogenic incubation suggest a (2.5 × 2.6=) 6.5-fold elevation of an incidence–induction period metric.

### 4.13. Major Errors of Mitosis and Meiosis

Moreover, massive genomic hyperploidy and chromosomal over-replication has also been demonstrated, following cannabis exposure of mammalian lymphocytes and oocytes [247,272]. This fits with the one or two rounds of genomic doubling required in testicular carcinogenesis [274].

Trisomies (of chromosomes 13, 18 and 21) and monosomies (of chromosome X, Turners syndrome) are also well-documented following cannabis exposure [44,45,46,49,63], making chromosomal mis-segregation a major feature of cannabis-related genotoxicity. This evidence is corroborated by the well-known positive status of cannabis in the micronucleus assay [276] and the documentation of lagging chromosomes [241,246,250,251,277,278].

One simplistic mechanism which may account for this is interference with tubulin synthesis and acetylation, which has been documented to occur from cannabinoid exposure [270] and which structurally disrupts the microtubules of the mitotic spindle, which they comprise [65]. Cannabis disrupts tubulin synthesis both directly and epigenomically [161,275].

These concepts are elegantly illustrated in the cited references.

It is highly pertinent to note that chromosomal positioning on the mitotic spindle is controlled by the kinetochore, which is a large 90-protein complex in mammals that binds the centromeric chromatin of each chromosome to 25–30 microtubules of the mitotic spindle [279]. Cannabis has been shown to broadly disrupt 14 of these key centrosomal proteins along with many key kinetochore proteins by heritable epigenetic mechanisms. Cannabis also disrupts the molecular kinesin and dynein–dynactin motors, which move chromosomes to the positive and negative ends of the microtubule, respectively.

Furthermore, specialised histones occur in centromeric chromatin, including H3 variant CENP-A [280], which carry key epigenomic post-translational modifications (PTMs). Primarily, among them, is the addition of a small ubiquitin-like modifier (SUMO) of proteins, a process known as sumoylation. This is a key PTM, which controls the addition of a complex set of further PTMs (methylation, acetylation, phosphorylation, ubiquitination, etc.), which then act combinatorially to control kinetochore function [281]. Histone sumoylation therefore acts as a key functional switch which controls the kinetochore function and the release of the spindle-associated checkpoint (SAC), which allows the chromosomal separation of anaphase to commence [279]. It may also be that a complex code of PTMs underlies the apparent ability of cells to identify each chromosome, as indicated by the relatively invariant nature of the chromosomes, which are predictably lost or duplicated in testicular carcinoma [274].

It is therefore of great interest to learn that this histone sumoylation switch is powerfully controlled by Δ9THC [282]. The application of Δ9THC in dividing cells causes major disruptions of the kinetochore signalling to the spindle assembly checkpoint (SAC) controller and leads to chromosomal mis-segregation errors. THC also directly affects Mdm2 (murine double minute) and SUMO-1 protein and acts to directly activate P53, the classical “guardian of the genome”. P53 in cannabis-exposed dividing cells can thus be expected to be activated both directly via cannabinoids and indirectly by sensing DNA breaks and damage. Oocyte mitotic errors are also a major feature of aging in human oocytes [283].

That is to say that a major epigenomic mechanism acts to regulate centromeric chromatin through the vital stages of attachment to the mitotic spindle and chromosomal segregation, and the epigenomic code controlling this sophisticated machinery is grossly disrupted by cannabinoid application. Since this mechanism likely controls both chromosomal counting and chromosomal segregation, it becomes apparent that kinetochore disruption plays a central role in both the induction of hyperploidy and chromosomal mis-segregation errors and all of their downstream sequelae.

Moreover, the multi-hit nature of carcinogenesis is often described [284,285,286,287,288]. As cannabinoids can deliver both double- and single-stranded DNA breaks and cause kinetochore disruption (hyperploidy and chromosomal mis-segregation), it becomes apparent that cannabinoids are capable of delivering the multi-point genomic hit in themselves. This also explains the dramatic effect on cell karyotype from minimal cannabis exposure (only a few puffs) in classical cell morphology studies [241,247,249,250,251,272,278,289].

The concept of presumptive pericentromeric chromatin dysregulation also explains the usual presence of an isochromosome 12, as the dysregulated pericentromeric epigenome presumably directs the aberrant scission of the chromosome at the centromere to form the isochromosome. The presence of KRAS, KIT and NRAS on this chromosome then confers a growth advantage on the mutant clone and malignant tumourigenesis is the end result of this process continued over time.

Combined with their important cannabinoid-related effects noted on the DNA methylome described above, these observations altogether begin to address the extraordinary issue of the dramatic acceleration both in incidence and oncogenic induction rate—which together is 6.5-fold, as described above.

### 4.14. Scope of Chromosomal Involvement

It is of interest to consider the extent of the chromosomal landscape deranged by cannabis. As noted above, the involvement of testicular cancer in cannabis carcinogenesis directly implicates 42% of the human genome in direct genomic disruption.

Other reports have implicated acute lymphoid leukaemia in the spectrum of cannabis carcinogenesis [212]. Chromosomal translocations between (at least) chromosomes, 4, 9, 10 and 11 are all well-described in that disorder [212,290]. Based on chromosomal lengths quoted in the human genome project, this totals (191 + 136 + 135 + 134 + 49=) 645 megabases of all 3000 megabases, or 21.5% in the human genome.

Reports from the congenital anomaly literature describe the implication of cannabis exposure with various trisomies/monosomies affecting chromosomes 13, 18, 21 and X and this sums up to (113 + 76 + 46 + 153=) 388 megabases or 12.9% of the human genome [44,45,46,49,50,51,53,57,59,63].

If one adds the chromosomesdamaged in acute lymphoid leukaemia, testis cancer and trisomies/monosomies all together, one reaches the impressive result of 1765 megabases, or 59% of the human genome being directly affected by cannabis genotoxicity. Much of this damage in terms of DNA breaks, pericentromeric chromatin dysfunction, and putative breakage–fusion–bridge cycles is epigenetically mediated.

### 4.15. Epigenomic Effects

A profoundly important epigenome-wide association study was recently published, looking at cannabis dependence and withdrawal with an 11-week period of documented abstinence in between the two sampling times [137]. The online dataset accompanying this paper contains many gene annotations for positive hits identified in the differentially methylated DNA CpG screen in this work, including 487 hits for the term “cancer”, 112 hits for “tumor”, 126 hits for “carcinoma”, 28 hits for “neoplasm”, 8 hits for “neoplasia”, 32 hits for “leukemia” and 17 hits for “lymphoma”, totalling 810 hits for malignancy in all. This makes cancerogenicity one of the major findings of this study and is clearly of direct and major importance to the present review of pathophysiological mechanisms. These important themes are too large to be considered in depth here and are considered in more depth in related papers [65,105,107,108,109,136,137,238,291].

### 4.16. Comparison to Tobacco and Alcohol

The effects of cannabis herb THC concentration were greater than those of tobacco and alcohol via bivariate analysis (Table 1 and Table 2 and Figure 1, Figure 2 and Figure 4) in an additive panel model (Table 4 and Figure 6), an interactive panel model (Supplementary Table S22 and Supplementary Figure S16), an interactive panel model at two lags (Table 8 and Figure 12), an interactive panel model at four lags (Supplementary Table S26 and Supplementary Figure S16) and in an interactive panel model at six lags (Supplementary Table S28 and Supplementary Figure S20). Overall, in many cases, the effects of cannabis metrics greatly exceeded those of tobacco and alcohol, as is well-demonstrated by the illustrations included in this report.

### 4.17. Cocaine

The finding that cocaine exposure was significantly associated with 18 cancers (Tabular Analysis) was noteworthy. However, it was also observed that there was a very strong association between cocaine and cannabis use with Pearson’s R = 0.78 corresponding to a significance level of 4.4 × 10^−55^ (Correlation Analysis). Moreover, in multiple regression studies, the effect of cocaine was very often obviated by the cannabinoid covariates. It therefore appears from these studies that cannabis alone or possibly cannabis–cocaine co-exposure accounts for much of the cocaine signal. A further dissection of the relative carcinogenicity of these two agents must await further research.

### 4.18. Generalizability

From a data analytical point of view, the European datasets are fabulously rich, and even more so by comparison with other datasets available internationally, which are relatively much more lean. A total of 170 cancer types are available in the detailed cancer statistics from ECIS. Moreover, cannabis use is measured by several metrics. For these reasons, we feel that these very impressive European datasets set a new benchmark in data exploration in this area. Several features in the present analysis point to a very high-level of significance, including vanishingly low *p*-values, minimum E-values ranging to infinity and relatively large sets of data. Moreover, all multivariable models utilised inverse-probability weighting, which, as noted, transforms models from merely observational context to a pseudo-randomised context where causal inferences may properly be drawn. A clear concordance between the European and American datasets is described in Section 4.6. Together, these factors of large and rich datasets, high-level significance and positive results on causal inferential analysis for many cancers suggest that, indeed, such results are likely to be widely generalizable internationally wherever reliable data exist on the relevant covariates.

### 4.19. Strengths and Limitations

This study has a number of strengths and limitations. Study strengths include the availability of continent-wide data or cancers and for drug and substance exposures alike. We were also fortunate to obtain access to a very long time series of cancer case rates covering 21 years. We were also able to access current data on cancer rates in Europe for many national registries. We were also able to access newly re-presented datasets on European cannabis exposure by many metrics as described recently in the study by Manthey and colleagues of the EMCDDA database [111]. Analytical strengths included the use of the quantitative techniques of causal analysis, particularly inverse-probability weighing and E-values to move beyond simply an observational ecological study and begin to address important causal questions in a pseudo-randomised framework. Utilising panel regression for multivariable adjustment carried several advantages—including that time and place can be accounted for intrinsic to the model structure without having to be specified in the model formula—so that their model standard deviation could be used to calculate E-values, they could be inverse-probability-weighted, they could be temporally lagged, and so that comparable models could be run across all tumour types virtually simultaneously in a purr–map workflow. The ability to display trends for all 40 cancers in one figure or table was also a notable strength.

Limitations of our study arise from several considerations. In common with most epidemiological studies, detailed information on personal cannabis exposure was not available to the present investigators. An interpolation of time series data was performed for the drug exposures to complete missing datasets in the manner described. Other methods of accounting for missing data, including multiple imputation, exist but are not suitable for the kinds of advanced analyses which were required in this study. For these reasons, it is important that future efforts work towards completing these gaps in the substance exposure data. We readily accept that in a very rich dataset, there are many ways to analyse such detailed data resources. Data also lend themselves to formal geospatial analyses where spatial networks are formally considered. This is a large project which will have to await subsequent dedicated analyses. In accordance with standard public health practice, we also examined only the positive signals in our data. Investigating negative signals is a project which must await a future opportunity. Finally, it is also of interest to study rarer cancers in greater depth. Such a project must await a future opportunity to accrue the requisite data for these malignancies, which may potentially offer key insights into cannabinoid-related oncogenic mechanisms.

## 5. Conclusions

In summary, this study demonstrates that cannabis exposure is linked across both time and space with the incidence of 25 of 41 cancers in Europe and thus confirms findings on other continents [17,41,44,167,168,169,211,212]. On epidemiological grounds, cannabis appears to be a more potent carcinogen than tobacco or alcohol in most tabulations, and based on E-value criteria, is a more potent carcinogen that tobacco and alcohol combined. It is important to note that the use of adjustment for multiple testing throughout these studies, the use of inverse-probability weighting in multivariable regressions and the use of E-values in bivariate and multivariable regressions move the present consideration merely from an extended report of various associations to a detailed investigation of causal relationships. All four questions considered in the Introductory Section have been answered in the affirmative in relation to the carcinogenic potential of cannabis at the level of population health, in concordance with the results of similar studies in North America, increased carcinogenic effects compared to known carcinogens tobacco and alcohol (often combined) and its implication in inheritable tumorigenesis and toxicity to multiple reproductive organs on several grounds. Together with recent findings demonstrating that cannabis exposure has driven a doubling of the US testicular cancer rate as well as rising US breast cancer rates, has increased the paediatric cancer rate in the USA by 50% in the last fifty years and also appears in the context of other mutagenic exposures to be driving current impressive and very concerning trends in pancreatic and liver cancer, the conclusion that the tumourigenic potential of cannabinoids has been seriously underestimated by the medical, scientific, professional and lay communities alike becomes inescapable. The present results strongly reinforce all of these worrying findings. It would appear that based on results such as those in the present study and of comparable similar studies in North America, a plethora of carcinogenic mechanisms outlined by the basic sciences particularly recent impressive epigenomic studies, the seriously concerning issue of transgenerational mutagenicity and malignant teratogenicity, the known exponential dose–response relationships and the implication of multiple cannabinoids, that communities need to severely restrict the exposure of their citizenry to environmental carcinogens such as cannabinoids not only in the interests of public health and safety, but also in order to protect the genomic, epigenomic and neurodevelopmental potential of several generations to come.

## Figures and Tables

**Table 1:** Regression modelling results, including slopes, significance levels and E-values for tobacco–cancer relationships.

Cancer	β-Estimate	Std Error	*p*-Value	P. Adj. Holm	E-Value Estimate	95% Lower Bound of E-Value
Non-Seminoma	0.2348	0.0233	5.36 × 10^−12^	1.34 × 10^−10^	2.10	1.92
Cervix	0.0533	0.0022	1.01 × 10^−112^	4.15 × 10^−111^	1.40	1.37
Lung	0.0259	0.0013	3.51 × 10^−75^	1.33 × 10^−73^	1.34	1.32
Stomach	0.0437	0.0022	8.16 × 10^−77^	3.26 × 10^−75^	1.34	1.32
Ovary	0.0339	0.0017	3.42 × 10^−76^	1.33 × 10^−74^	1.34	1.32
Kidney	0.0288	0.0020	3.90 × 10^−45^	1.44 × 10^−43^	1.28	1.26
All Cancers nNMSC	0.0150	0.0012	1.67 × 10^−33^	5.50 × 10^−32^	1.27	1.24
Pancreas	0.0247	0.0019	1.19 × 10^−38^	4.16 × 10^−37^	1.27	1.24
Corpus Uteri	0.0350	0.0028	1.34 × 10^−34^	4.56 × 10^−33^	1.25	1.23
Larynx	0.0202	0.0020	2.26 × 10^−23^	6.78 × 10^−22^	1.23	1.20
Prostate	0.0188	0.0019	4.13 × 10^−22^	1.20 × 10^−20^	1.22	1.20
Leukaemia	0.0264	0.0030	9.97 × 10^−18^	2.79 × 10^−16^	1.21	1.18
All Cancers	0.0132	0.0019	1.95 × 10^−11^	4.69 × 10^−10^	1.22	1.18
Oesophagus	0.0205	0.0024	1.24 × 10^−16^	3.34 × 10^−15^	1.20	1.17
Seminoma	0.0128	0.0050	0.0160	0.1437	1.38	1.15
Breast	0.0178	0.0039	4.39 × 10^−06^	7.90 × 10^−5^	1.14	1.10
Oropharynx	0.0213	0.0096	0.0280	0.1894	1.16	1.05
Leukaemia—Lymphoid	−0.0008	0.0028	0.7663	1.0000	1.05	-
Colorectum	−0.0018	0.0013	0.1638	0.6553	1.07	-
Brain	−0.0063	0.0018	5.34 × 10^−4^	0.0080	1.12	-
Vulva and Vagina	−0.0059	0.0019	0.0021	0.0256	1.14	-
Anus	−0.0055	0.0016	8.48 × 10^−4^	0.0115	1.14	-
Leukaemia—Myeloid	−0.0103	0.0046	0.0269	0.1894	1.14	-
Penis	−0.0056	0.0017	8.22 × 10^−4^	0.0115	1.15	-
Non-Hodgkin’s Lymphoma	−0.0083	0.0017	1.05 × 10^−6^	1.99 × 10^−5^	1.15	-
Bladder	−0.0104	0.0018	7.02 × 10^−9^	1.40 × 10^−7^	1.16	-
Gallbladder and Biliary	−0.0108	0.0027	7.95 × 10^−5^	0.0013	1.17	-
Ovarian Dysgerminoma	−0.0014	0.0030	0.6599	1.0000	1.17	-
Melanoma	−0.0145	0.0022	3.22 × 10^−11^	7.40 × 10^−10^	1.18	-
Medulloblastoma	−0.0023	0.0054	0.6807	1.0000	1.18	-
Testis	−0.0117	0.0018	1.98 × 10^−10^	4.35 × 10^−9^	1.21	-
Kaposi	−0.0081	0.0044	0.0643	0.3213	1.21	-
Vagina	−0.0064	0.0025	0.0124	0.1243	1.22	-
Liver	−0.0192	0.0027	3.63 × 10^−12^	9.43 × 10^−11^	1.22	-
Oropharynx_Broad	−0.0172	0.0038	8.05 × 10^−6^	1.37E-04	1.22	-
Hepatocellular	−0.0098	0.0042	0.0237	0.1894	1.24	-
Mesothelioma	−0.0291	0.0097	0.0032	0.0351	1.28	-
Hodgkin’s	−0.0160	0.0014	2.24 × 10^−28^	6.93 × 10^−27^	1.29	-
Myeloma	−0.0175	0.0015	1.06 × 10^−31^	3.40 × 10^−30^	1.30	-
Thyroid	−0.0390	0.0028	2.04 × 10^−40^	7.36 × 10^−39^	1.33	-
Vulva	−0.0309	0.0047	3.98 × 10^−10^	8.37 × 10^−9^	1.39	-

Table key: β-Estimate—estimate of the regression coefficient; Std. Error—standard error of the regression coefficient; *p*-value—significance level; P. Adj. Holm—*p*-value adjusted for multiple testing by the method of Holm; E-value—expected value required of some unknown confounder covariate with both the exposure and the outcome to explain the observed effect; lower bound of the E-value—the 95% lower bound of the confidence interval of the E-value.

**Table 2:** Regression modelling results, including slopes, significance levels and E-values for alcohol–cancer relationships.

Cancer	β-Estimate	Std. Error	*p*-Value	P. Adj. Holm	E-Value Estimate	Lower Bound E-Value
Oesophagus	0.1388	0.0059	5.92 × 10^−108^	2.43 × 10^−106^	1.80	1.75
All Cancers	0.0888	0.0069	3.72 × 10^−34^	1.12 × 10^−32^	1.85	1.74
Cervix	0.1395	0.0060	1.39 × 10^−105^	5.56 × 10^−104^	1.79	1.74
Prostate	0.0950	0.0048	1.12 × 10^−78^	4.36 × 10^−77^	1.70	1.64
Lung	0.0663	0.0036	3.94 × 10^−69^	1.50 × 10^−67^	1.66	1.60
All Cancers nNMSC	0.0456	0.0032	4.20 × 10^−44^	1.47 × 10^−42^	1.58	1.52
Kidney	0.0750	0.0054	3.29 × 10^−42^	1.12 × 10^−40^	1.54	1.48
Leukaemia—Lymphoid	0.0547	0.0091	4.19 × 10^−09^	9.63 × 10^−08^	1.61	1.46
Ovary	0.0652	0.0049	6.49 × 10^−38^	2.08 × 10^−36^	1.52	1.46
Stomach	0.0711	0.0064	1.65 × 10^−27^	4.62 × 10^−26^	1.45	1.40
Pancreas	0.0547	0.0051	2.34 × 10^−26^	6.31 × 10^−25^	1.45	1.39
Breast	0.0771	0.0104	1.82 × 10^−13^	4.72 × 10^−12^	1.34	1.28
Larynx	0.0332	0.0054	1.05 × 10^−9^	2.52 × 10^−8^	1.31	1.24
Leukaemia—Myeloid	0.0517	0.0163	0.0017	0.0284	1.38	1.21
Anus	0.0143	0.0038	0.0002	0.0036	1.25	1.16
Leukaemia	0.0305	0.0083	0.0003	0.0051	1.23	1.15
Corpus Uteri	0.0290	0.0080	0.0003	0.0061	1.22	1.14
Penis	0.0109	0.0038	0.0036	0.0542	1.21	1.11
Melanoma	0.0138	0.0059	0.0200	0.2398	1.17	1.06
Vulva and Vagina	0.0048	0.0044	0.2774	1.0000	1.12	1.00
Non-Hodgkin’s Lymphoma	0.0067	0.0046	0.1458	1.0000	1.13	1.00
Vulva	0.0104	0.0190	0.5831	1.0000	1.19	1.00
Oropharynx_Broad	0.0152	0.0166	0.3621	1.0000	1.20	1.00
Vagina	0.0151	0.0097	0.1221	1.0000	1.37	1.00
Colorectum	−0.0009	0.0036	0.7948	1.0000	1.05	-
Brain	−0.0113	0.0049	0.0216	0.2398	1.17	-
Non-Seminoma	−0.0316	0.4242	0.9411	1.0000	1.17	-
Testis	−0.0106	0.0043	0.0130	0.1825	1.19	-
Myeloma	−0.0172	0.0035	9.02 × 10^−7^	1.98 × 10^−5^	1.29	-
Hepatocellular	−0.0232	0.0170	0.1779	1.0000	1.41	-
Liver	−0.0749	0.0061	5.30 × 10^−33^	1.54 × 10^−31^	1.54	-
Bladder	−0.0678	0.0046	5.27 × 10^−47^	1.90 × 10^−45^	1.56	-
Hodgkin’s	−0.0425	0.0032	8.56 × 10^−38^	2.65 × 10^−36^	1.56	-
Gallbladder and Biliary	−0.0721	0.0053	8.46 × 10^−39^	2.79 × 10^−37^	1.60	-
Ovarian Dysgerminoma	−0.0102	0.0143	0.4840	1.0000	1.64	-
Kaposi	−0.0430	0.0179	0.0174	0.2260	1.65	-
Mesothelioma	−0.1087	0.0353	0.0024	0.0382	1.69	-
Oropharynx	−0.2054	0.0290	6.49 × 10^−12^	1.62 × 10^−10^	1.70	-
Thyroid	−0.1136	0.0063	3.01 × 10^−66^	1.11 × 10^−64^	1.72	-
Medulloblastoma	−0.0374	0.0272	0.1908	1.0000	2.43	-
Seminoma	−0.0882	0.0250	0.0011	0.0203	3.01	-

Table key: β-Estimate—estimate of the regression coefficient; Std. Error—standard error of the regression coefficient; *p*-value—significance level; P. Adj. Holm—*p*-value adjusted for multiple testing by the method of Holm; E-value—expected value required of some unknown confounder covariate with both the exposure and the outcome to explain the observed effect; lower bound of the E-value—the 95% lower bound of the confidence interval of the E-value.

**Table 3:** Regression modelling results, including slopes, significance levels and E-values for cannabis herb THC concentration–cancer relationship slopes by mixed-effects regression.

Cancer	β-Estimate	Std. Error	*p*-Value	P. Adj. Holm	E-Value Estimate	E-Value Lower Bound
All Cancers nNMSC	2.6457	0.0778	4.69 × 10^−180^	1.41 × 10^−178^	4.88 × 10^13^	8.28 × 10^12^
All Cancers	2.8076	0.1071	6.70 × 10^−102^	1.94 × 10^−100^	3.15 × 10^10^	5.46 × 10^9^
Prostate	5.2624	0.2489	1.55 × 10^−86^	4.35 × 10^−85^	4.59 × 10^7^	9.56 × 10^6^
Breast	2.4686	0.1369	1.36 × 10^−65^	3.67 × 10^−64^	3.51 × 10^6^	7.37 × 10^5^
Melanoma	5.9745	0.3598	8.89 × 10^−57^	2.31 × 10^−55^	5.59 × 10^5^	1.28 × 10^5^
Kidney	3.5398	0.2357	1.64 × 10^−47^	4.11 × 10^−46^	3.32 × 10^5^	6.95 × 10^4^
Colorectum	2.5415	0.1742	4.61 × 10^−45^	1.11 × 10^−43^	2.03 × 10^5^	4.33 × 10^4^
Pancreas	3.6625	0.2554	1.04 × 10^−43^	2.40 × 10^−42^	1.67 × 10^5^	3.55 × 10^4^
Testis	4.5855	0.3263	2.84 × 10^−41^	6.25 × 10^−40^	5.74 × 10^7^	5.26 × 10^6^
Thyroid	4.9267	0.3666	3.26 × 10^−38^	6.85 × 10^−37^	8.56 × 10^7^	6.63 × 10^6^
Non-Hodgkin’s Lymphoma	3.7844	0.2854	7.36 × 10^−38^	1.47 × 10^−36^	6.82 × 10^4^	1.46 × 10^4^
Lung	2.0357	0.1687	5.35 × 10^−32^	1.02 × 10^−30^	3.40 × 10^4^	7.01 × 10^3^
Anus	3.6672	0.3097	1.61 × 10^−30^	2.89 × 10^−29^	2.77 × 10^6^	2.68 × 10^5^
Oesophagus	3.5244	0.3847	1.71 × 10^−19^	2.90 × 10^−18^	2.58 × 10^3^	557.87
Leukaemia—Myeloid	4.2458	0.6638	8.92 × 10^−10^	1.43 × 10^−8^	2.22 × 10^5^	6.35 × 10^3^
Oropharynx_Broad	2.5577	0.4523	2.82 × 10^−8^	4.23 × 10^−7^	5.05 × 10^2^	74.07
Leukaemia—Lymphoid	1.6165	0.4299	2.16 × 10^−4^	0.0026	1784.06	51.56
Brain	1.1480	0.3174	3.09 × 10^−4^	0.0034	19.93	5.26
Myeloma	0.8656	0.2619	9.80 × 10^−4^	0.0098	127.86	10.41
Corpus Uteri	1.2936	0.4601	0.0050	0.0400	16.44	3.24
Liver	0.7809	0.3581	0.0294	0.2059	37.76	2.05
Hodgkin’s	0.4718	0.3032	0.1200	0.5999	12.08	1.00
Cervix	0.1742	0.3411	0.6097	1.0000	2.38	1.00
Bladder	0.0250	0.2849	0.9302	1.0000	1.35	1.00
Kaposi	0.0346	0.4840	0.9432	1.0000	2.09	1.00
Ovary	−0.0127	0.3205	0.9684	1.0000	1.21	-
Oropharynx	−2.7034	1.4854	0.0699	0.4192	54.33	-
Gallbladder and Biliary	−1.1202	0.3420	0.0011	0.0098	487.89	-
Larynx	−1.3647	0.3578	1.42 × 10^−4^	0.0018	27.57	-
Stomach	−0.8809	0.2164	4.92 × 10^−5^	6.89 × 10^−4^	53.25	-

Table key: β-Estimate—estimate of the regression coefficient; Std. Error—standard error of the regression coefficient; *p*-value—significance level; P. Adj. Holm—*p*-value adjusted for multiple testing by the method of Holm; E-value—expected value required of some unknown confounder covariate with both the exposure and the outcome to explain the observed effect; lower bound of the E-value—the 95% lower bound of the confidence interval of the E-value.

**Table 4:** Regression modelling results, including slopes, significance levels and E-values for THC concentration of cannabis resin–cancer relationship slopes by mixed-effects regression.

Cancer	β-Estimate	Std. Error	*p*-Value	P. Adj. Holm	E-Value Estimate	E-Value Lower Bound
All Cancers nNMSC	1.1789	0.0622	2.82 × 10^−70^	8.46 × 10^−69^	2.22 × 10^5^	6.70 × 10^4^
Melanoma	2.8370	0.2030	1.36 × 10^−41^	3.93 × 10^−40^	795.41	343.77
All Cancers	1.0317	0.0758	7.34 × 10^−37^	2.05 × 10^−35^	2065.96	761.63
Pancreas	1.5037	0.1432	6.91 × 10^−25^	1.80 × 10^−23^	185.22	79.36
Breast	0.7634	0.0813	2.34 × 10^−20^	5.86 × 10^−19^	142.23	58.19
Anus	1.1325	0.1299	1.12 × 10^−17^	2.69 × 10^−16^	159.29	59.29
Non-Hodgkin’s Lymphoma	1.4279	0.1679	4.80 × 10^−17^	1.10 × 10^−15^	101.29	40.73
Lung	0.7312	0.0921	4.22 × 10^−15^	9.29 × 10^−14^	71.04	29.12
Kidney	0.9545	0.1363	3.85 × 10^−12^	8.08 × 10^−11^	41.16	17.33
Oesophagus	1.2442	0.2143	7.86 × 10^−9^	1.42 × 10^−7^	23.50	9.87
Testis	0.8143	0.1502	7.38 × 10^−8^	1.26 × 10^−6^	33.71	11.76
Oropharynx	2.3818	0.5030	3.70 × 10^−6^	5.92 × 10^−5^	69.39	15.59
Oropharynx_Broad	1.1861	0.2844	3.67 × 10^−5^	4.78 × 10^−4^	24.73	7.15
Bladder	0.5357	0.1597	8.18-04	0.0098	7.87	3.04
Hodgkin’s	0.3397	0.1236	0.0061	0.0608	6.91	2.28
Brain	0.5346	0.1948	0.0061	0.0608	5.20	2.05
Thyroid	0.3513	0.1603	0.0287	0.2006	5.58	1.51
Prostate	0.3118	0.1532	0.0420	0.2521	4.40	1.23
Kaposi	0.1992	0.5144	0.6998	1.0000	9.45	1.00
Myeloma	0.0387	0.1039	0.7096	1.0000	1.70	1.00
Colorectum	0.0315	0.0960	0.7429	1.0000	1.58	1.00
Corpus Uteri	−0.3324	0.2662	0.2119	0.8477	2.82	-
Leukaemia—Myeloid	−0.4144	0.2523	0.1020	0.5100	5.73	-
Liver	−0.3425	0.1377	0.0130	0.1044	7.02	-
Leukaemia—Lymphoid	−0.4516	0.1521	0.0033	0.0366	15.46	-
Cervix	−0.8035	0.1902	2.54 × 10^−5^	3.56 × 10^−4^	12.79	-
Ovary	−0.8408	0.1827	4.57 × 10^−6^	6.86 × 10^−5^	14.58	-
Gallbladder and Biliary	−0.8315	0.1407	4.78 × 10^−9^	9.08 × 10^−8^	116.77	-
Larynx	−1.2162	0.2026	2.47 × 10^−9^	4.94 × 10^−8^	21.12	-
Stomach	−1.3226	0.1114	4.89 × 10^−31^	1.32 × 10^−29^	352.51	-

Table key: β-Estimate—estimate of the regression coefficient; Std. Error—standard error of the regression coefficient; *p*-value—significance level; P. Adj. Holm—*p*-value adjusted for multiple testing by the method of Holm; E-value—expected value required of some unknown confounder covariate with both the exposure and the outcome to explain the observed effect; lower bound of the E-value—the 95% lower bound of the confidence interval of the E-value.

**Table 5:** Collated results of bivariate regressions for cannabis metrics by model type.

Herb. THC	Resin. THC	Daily Interpolated	Last Month’s Cannabis
All Cancers	All Cancers	All Cancers	
All Cancers nNMSC	All Cancers nNMSC	All Cancers nNMSC	All Cancers nNMSC
Anus	Anus	Anus	Anus
	Bladder	Bladder	Bladder
Brain	Brain		
Breast	Breast	Breast	Breast
			Cervix
Colorectum	Colorectum		Colorectum
Corpus Uteri			
			Gallbladder and Biliary
	Hodgkin’s	Hodgkin’s	Hodgkin’s
	Kaposi	Kaposi	Kaposi
Kidney	Kidney	Kidney	Kidney
		Larynx	Larynx
Leukaemia			
Leukaemia—Lymphoid			Leukaemia—Lymphoid
Leukaemia—Myeloid		Leukaemia—Myeloid	Leukaemia—Myeloid
Liver		Liver	Liver
Lung	Lung	Lung	Lung
Melanoma	Melanoma	Melanoma	
	Mesothelioma		
Myeloma		Myeloma	Myeloma
Non-Hodgkin’s Lymphoma	Non-Hodgkin’s Lymphoma	Non-Hodgkin’s Lymphoma	Non-Hodgkin’s Lymphoma
Oesophagus	Oesophagus	Oesophagus	Oesophagus
	Oropharynx	Oropharynx	
Oropharynx_Broad	Oropharynx_Broad	Oropharynx_Broad	Oropharynx_Broad
Pancreas	Pancreas	Pancreas	
Prostate	Prostate		
			Stomach
Testis	Testis		
Thyroid	Thyroid	Thyroid	Thyroid
	Vulva and Vagina	Vulva and Vagina	Vulva and Vagina

Key—Each column in this table relates to a different independent regression covariate.

**Table 6:** Relative risks, attributable fractions in the exposed and population-attributable risks for high- vs. low-tobacco-exposure nations, respectively.

Cancer	*p*-Value	RR (C.I.)	AFE (C.I.)	PAF (C.I.)
Oropharynx	0.0000	3.627 (3.6028, 3.6514)	0.7243 (0.7224, 0.7261)	0.5043 (0.5019, 0.5066)
Cervix	0.0000	1.9962 (1.9932, 1.9992)	0.499 (0.4983, 0.4998)	0.2763 (0.2757, 0.2769)
Stomach	0.0000	1.8241 (1.8216, 1.8266)	0.4518 (0.451, 0.4525)	0.2468 (0.2463, 0.2474)
Kidney	0.0000	1.7574 (1.7549, 1.7599)	0.431 (0.4302, 0.4318)	0.2315 (0.2309, 0.2321)
Prostate	0.0000	1.6336 (1.6328, 1.6344)	0.3879 (0.3876, 0.3882)	0.1964 (0.1962, 0.1966)
Pancreas	0.0000	1.601 (1.5985, 1.6035)	0.3754 (0.3744, 0.3764)	0.1929 (0.1923, 0.1935)
Corpus Uteri	0.0000	1.5789 (1.577, 1.5808)	0.3666 (0.3659, 0.3674)	0.1758 (0.1753, 0.1763)
Leukaemia	0.0000	1.5676 (1.565, 1.5701)	0.3621 (0.361, 0.3631)	0.1615 (0.1609, 0.1621)
All Cancers	0.0000	1.5195 (1.5186, 1.5203)	0.3419 (0.3415, 0.3423)	0.2234 (0.2231, 0.2237)
Ovary	0.0000	1.4957 (1.4936, 1.4978)	0.3314 (0.3305, 0.3323)	0.1596 (0.1591, 0.1602)
Larynx	0.0000	1.453 (1.4495, 1.4565)	0.3118 (0.3101, 0.3134)	0.1549 (0.1539, 0.1559)
Lung	0.0000	1.442 (1.4409, 1.4431)	0.3065 (0.306, 0.3071)	0.1479 (0.1476, 0.1482)
Oesophagus	0.0000	1.3267 (1.3236, 1.3298)	0.2462 (0.2445, 0.248)	0.115 (0.114, 0.1159)
All Cancers nNMSC	0.0000	1.3089 (1.3086, 1.3093)	0.236 (0.2358, 0.2362)	0.0995 (0.0994, 0.0996)
Oropharynx_Broad	0.0000	1.2962 (1.2905, 1.302)	0.2285 (0.2251, 0.232)	0.1432 (0.1408, 0.1456)
Brain	0.0000	1.0472 (1.0452, 1.0492)	0.0451 (0.0432, 0.0469)	0.0178 (0.017, 0.0185)
Colorectum	0.0000	1.0408 (1.0402, 1.0415)	0.0392 (0.0386, 0.0398)	0.0155 (0.0152, 0.0157)
Breast	0.0000	1.0281 (1.0275, 1.0286)	0.0273 (0.0268, 0.0278)	0.011 (0.0107, 0.0112)
Liver	2.25 × 10^−34^	1.0122 (1.0102, 1.0141)	0.012 (0.0101, 0.0139)	0.0049 (0.0041, 0.0056)
Bladder	5.05 × 10^−45^	1.0089 (1.0076, 1.0101)	0.0088 (0.0076, 0.01)	0.0035 (0.003, 0.004)
Hepatocellular	1.75 × 10^−2^	1.0937 (1.0063, 1.1888)	0.0857 (0.0062, 0.1588)	0.0081 (0.0003, 0.0159)
Penis	7.09 × 10^−13^	0.9807 (0.9754, 0.986)	−0.0197 (−0.0252, −0.0142)	−0.0068 (−0.0087, −0.0049)
Melanoma	0.0000	0.9646 (0.9632, 0.966)	−0.0367 (−0.0382, −0.0352)	−0.0139 (−0.0144, −0.0133)
Gallbladder and Biliary	1.33 × 10^−284^	0.939 (0.9358, 0.9422)	−0.065 (−0.0686, −0.0613)	−0.0216 (−0.0227, −0.0204)
Leukaemia—Lymphoid	4.55 × 10^−161^	0.9207 (0.9152, 0.9262)	−0.0861 (−0.0927, −0.0796)	−0.045 (−0.0482, −0.0417)
Hodgkin’s	0.0000	0.9043 (0.9017, 0.9069)	−0.1059 (−0.109, −0.1027)	−0.0383 (−0.0393, −0.0372)
Anus	0.0000	0.8729 (0.8682, 0.8776)	−0.1456 (−0.1518, −0.1395)	−0.0522 (−0.0542, −0.0501)
Thyroid	0.0000	0.8523 (0.8508, 0.8538)	−0.1733 (−0.1754, −0.1712)	−0.0617 (−0.0624, −0.061)
Myeloma	0.0000	0.8404 (0.8381, 0.8427)	−0.1899 (−0.1931, −0.1867)	−0.0669 (−0.0679, −0.0659)
Testis	0.0000	0.8054 (0.8038, 0.807)	−0.2416 (−0.2441, −0.2392)	−0.0796 (−0.0803, −0.0789)
Non-Hodgkin’s Lymphoma	0.0000	0.8028 (0.8015, 0.8041)	−0.2456 (−0.2477, −0.2436)	−0.0724 (−0.0729, −0.0719)
Vulva and Vagina	0.0000	0.7813 (0.7773, 0.7853)	−0.28 (−0.2866, −0.2734)	−0.084 (−0.0857, −0.0823)
Vagina	1.84 × 10^−85^	0.7031 (0.6786, 0.7285)	−0.4223 (−0.4737, −0.3727)	−0.065 (−0.0708, −0.0592)
Leukaemia—Myeloid	0.0000	0.6386 (0.6329, 0.6443)	−0.566 (−0.5801, −0.5521)	−0.2466 (−0.2514, −0.2417)
Vulva	0.0000	0.591 (0.5808, 0.6015)	−0.6919 (−0.7217, −0.6626)	−0.0926 (−0.0951, −0.09)

Table key: R.R.—Relative risk; AFE—attributable fraction in the exposed; PAR—population-attributable risk; C.I.—confidence interval; *p*-value—significance level.

**Table 7:** Relative risks, attributable fractions in the exposed and population-attributable risks for high- vs. low-cannabis-exposure nations, respectively.

Cancer	*p*-Value	RR (C.I.)	AFE (C.I.)	PAF (C.I.)
Kaposi	1.86 × 10^−170^	2.081 (1.9739, 2.1939)	0.5195 (0.4934, 0.5442)	0.2573 (0.2376, 0.2765)
Liver	0.0000	1.7627 (1.7556, 1.7698)	0.4327 (0.4304, 0.435)	0.4077 (0.4055, 0.4099)
Thyroid	0.0000	1.6921 (1.6861, 1.6981)	0.409 (0.4069, 0.4111)	0.385 (0.383, 0.3871)
Stomach	0.0000	1.6847 (1.68, 1.6893)	0.4064 (0.4048, 0.408)	0.3827 (0.3811, 0.3843)
Oropharynx_Broad	0.0000	1.6204 (1.6104, 1.6304)	0.3829 (0.3791, 0.3866)	0.3306 (0.3271, 0.3342)
Larynx	0.0000	1.5906 (1.5822, 1.5991)	0.3713 (0.368, 0.3746)	0.3524 (0.3492, 0.3557)
Breast	0.0000	1.4899 (1.4882, 1.4915)	0.3288 (0.3281, 0.3296)	0.3095 (0.3088, 0.3102)
All Cancers	0.0000	1.4057 (1.4047, 1.4067)	0.2886 (0.2881, 0.2891)	0.2411 (0.2406, 0.2415)
Hodgkin’s	0.0000	1.2985 (1.2918, 1.3054)	0.2299 (0.2259, 0.2339)	0.213 (0.2092, 0.2168)
Bladder	0.0000	1.2896 (1.2866, 1.2925)	0.2246 (0.2228, 0.2263)	0.2077 (0.206, 0.2094)
Kidney	0.0000	1.287 (1.2836, 1.2903)	0.223 (0.221, 0.225)	0.2062 (0.2043, 0.2081)
Pancreas	0.0000	1.2859 (1.2822, 1.2895)	0.2223 (0.2201, 0.2245)	0.2056 (0.2035, 0.2077)
Prostate	0.0000	1.274 (1.2728, 1.2751)	0.2151 (0.2144, 0.2158)	0.1984 (0.1978, 0.1991)
Lung	0.0000	1.2704 (1.2685, 1.2724)	0.2129 (0.2116, 0.2141)	0.1992 (0.198, 0.2004)
Leukaemia	0.0000	1.2471 (1.2436, 1.2507)	0.1981 (0.1959, 0.2004)	0.1819 (0.1798, 0.1841)
Colorectum	0.0000	1.2186 (1.2173, 1.22)	0.1794 (0.1785, 0.1803)	0.1646 (0.1638, 0.1655)
All Cancers nNMSC	0.0000	1.2121 (1.2115, 1.2127)	0.175 (0.1746, 0.1754)	0.1607 (0.1603, 0.1611)
Gallbladder and Biliary	1.91 × 10^−252^	1.109 (1.1023, 1.1156)	0.0982 (0.0928, 0.1036)	0.0905 (0.0855, 0.0955)
Myeloma	0.0000	1.1072 (1.1022, 1.1123)	0.0968 (0.0927, 0.101)	0.0884 (0.0846, 0.0922)
Leukaemia—Myeloid	6.17 × 10^−64^	1.0916 (1.0806, 1.1028)	0.084 (0.0746, 0.0933)	0.0655 (0.0581, 0.073)
Leukaemia—Lymphoid	9.73 × 10^−101^	1.0805 (1.0728, 1.0883)	0.0745 (0.0679, 0.0811)	0.0582 (0.0529, 0.0634)
Corpus Uteri	0.0000	1.0657 (1.0638, 1.0676)	0.0616 (0.06, 0.0633)	0.0543 (0.0528, 0.0557)
Cervix	0.0000	1.056 (1.0534, 1.0586)	0.053 (0.0507, 0.0554)	0.0481 (0.046, 0.0503)
Testis	1.76 × 10^−119^	1.0386 (1.0353, 1.042)	0.0372 (0.0341, 0.0403)	0.0337 (0.0309, 0.0365)
Ovary	2.24 × 10^−92^	1.0245 (1.0222, 1.0269)	0.024 (0.0217, 0.0262)	0.0217 (0.0196, 0.0238)
Anus	1.47 × 10^−8^	1.0254 (1.0164, 1.0346)	0.0248 (0.0161, 0.0334)	0.0225 (0.0146, 0.0303)
Non-Hodgkin’s Lymphoma	1.19 × 10^−70^	0.9781 (0.9757, 0.9805)	−0.0224 (−0.0249, −0.0199)	−0.02 (−0.0223, −0.0178)
Melanoma	6.32 × 10^−297^	0.9571 (0.9549, 0.9594)	−0.0448 (−0.0472, −0.0424)	−0.0403 (−0.0425, −0.0381)
Hepatocellular	0.0175	0.9143 (0.8412, 0.9938)	−0.0937 (−0.1888, −0.0063)	−0.0848 (−0.1699, −0.006)
Oesophagus	0.0000	0.8962 (0.8929, 0.8996)	−0.1158 (−0.12, −0.1116)	−0.1037 (−0.1074, −0.0999)
Brain	0.0000	0.7878 (0.7855, 0.79)	−0.2694 (−0.273, −0.2658)	−0.2371 (−0.2402, −0.234)
Penis	0.0000	0.7663 (0.7604, 0.7722)	−0.305 (−0.3152, −0.2949)	−0.2659 (−0.2745, −0.2574)
Vulva	0.0000	0.7187 (0.7103, 0.7271)	−0.3915 (−0.4078, −0.3753)	−0.2291 (−0.2375, −0.2207)
Vagina	2.72 × 10^−162^	0.7082 (0.6907, 0.7262)	−0.412 (−0.4478, −0.377)	−0.2403 (−0.2585, −0.2223)
Vulva and Vagina	0.0000	0.6967 (0.6918, 0.7017)	−0.4353 (−0.4456, −0.4251)	−0.3788 (−0.3874, −0.3703)
Oropharynx	0.0000	0.6292 (0.6245, 0.6339)	−0.5894 (−0.6012, −0.5776)	−0.3675 (−0.3739, −0.3612)

Table key: R.R.—Relative risk; AFE—attributable fraction in the exposed; PAR—population-attributable risk; C.I.—confidence interval; *p*-value—significance level.

**Table 8:** Positive and significant terms from the additive panel model.

Cancer	Term	β-Estimate	Std. Error	*p*-Value	Adj. P. FDR	Adj. P. Holm	E-Value Estimate	E-Value 95% Lower Bound
All Cancers nNMSC	LM.Cannabis	28.793	2.305	7.96 × 10^−26^	5.58 × 10^−25^	4.86 × 10^−24^	3.50 × 10^60^	1.29 × 10^51^
Myeloma	LM.Cannabis	18.834	2.110	1.64 × 10^−16^	7.15 × 10^−16^	8.99 × 10^−15^	1.79 × 10^43^	6.91 × 10^33^
Lung	LM.Cannabis	27.039	2.293	1.32 × 10^−25^	8.38 × 10^−25^	7.90 × 10^−24^	7.26 × 10^39^	2.00 × 10^33^
Kidney	LM.Cannabis	34.251	2.951	4.67 × 10^−25^	2.73 × 10^−24^	2.76 × 10^−23^	1.70 × 10^39^	4.68 × 10^32^
Pancreas	LM.Cannabis	29.502	2.956	7.13 × 10^−20^	3.57 × 10^−19^	4.07 × 10^−18^	5.97 × 10^33^	1.65 × 10^27^
Leukaemia—Lymphoid	LM.Cannabis	9.998	2.584	3.66 × 10^−4^	5.56 × 10^−4^	9.14 × 10^−3^	1.09 × 10^45^	2.64 × 10^22^
All Cancers nNMSC	THC.Herb	10.642	0.521	1.15 × 10^−47^	1.34 × 10^−46^	7.48 × 10^−46^	3.69 × 10^22^	2.73 × 10^20^
Non-Hodgkin’s Lymphoma	LM.Cannabis	26.819	3.429	1.75 × 10^−13^	6.45 × 10^−13^	9.10 × 10^−12^	3.42 × 10^26^	9.39 × 10^19^
Colorectum	LM.Cannabis	18.142	2.303	1.13 × 10^−13^	4.38 × 10^−13^	5.97 × 10^−12^	5.83 × 10^25^	2.76 × 10^19^
Prostate	LM.Cannabis	22.525	3.112	6.20 × 10^−12^	1.97 × 10^−11^	3.04 × 10^−10^	3.77 × 10^24^	1.04 × 10^18^
All Cancers	LM.Cannabis	17.153	4.312	1.51 × 10^−4^	2.40 × 10^−4^	4.07 × 10^−3^	4.43 × 10^34^	5.71 × 10^17^
Pancreas	THC.Herb	15.302	0.584	1.27 × 10^−72^	8.88 × 10^−71^	8.88 × 10^−71^	4.61 × 10^17^	2.33 × 10^16^
Hodgkin’s	LM.Cannabis	13.231	2.507	2.91 × 10^−7^	6.56 × 10^−7^	1.16 × 10^−5^	3.05 × 10^25^	1.41 × 10^16^
Stomach	LM.Cannabis	20.239	3.032	1.67 × 10^−10^	4.88 × 10^−10^	7.87 × 10^−9^	4.86 × 10^22^	1.34 × 10^16^
Stomach	THC.Herb	15.302	0.599	1.20 × 10^−70^	4.21 × 10^−69^	8.29 × 10^−69^	1.68 × 10^17^	8.51 × 10^15^
Prostate	THC.Herb	15.119	0.616	2.58 × 10^−67^	6.01 × 10^−66^	1.75 × 10^−65^	3.94 × 10^16^	1.98 × 10^15^
Breast	LM.Cannabis	13.958	2.225	1.66 × 10^−9^	4.29 × 10^−9^	7.28 × 10^−8^	2.18 × 10^21^	5.99 × 10^14^
Kidney	THC.Herb	13.038	0.583	8.04 × 10^−61^	1.41 × 10^−59^	5.38 × 10^−59^	1.32 × 10^15^	6.66 × 10^13^
Lung	THC.Herb	9.751	0.453	8.43 × 10^−58^	1.18 × 10^−56^	5.56 × 10^−56^	3.69 × 10^14^	1.86 × 10^13^
All Cancers	THC.Herb	9.102	1.851	4.52 × 10^−6^	8.78 × 10^−6^	1.58 × 10^−4^	3.37 × 10^18^	1.89 × 10^11^
Breast	THC.Herb	7.398	0.440	2.23 × 10^−42^	2.23 × 10^−41^	1.43 × 10^−40^	2.83 × 10^11^	1.43 × 10^10^
Melanoma	LM.Cannabis	19.128	4.101	5.13 × 10^−6^	9.71 × 10^−6^	1.74 × 10^−4^	8.80 × 10^15^	2.42 × 10^9^
Non-Hodgkin’s Lymphoma	THC.Herb	10.009	0.679	2.68 × 10^−35^	2.35 × 10^−34^	1.69 × 10^−33^	1.24 × 10^10^	6.20 × 10^8^
Oropharynx	THC.Herb	17.962	5.844	3.42 × 10^−3^	4.35 × 10^−3^	5.47 × 10^−2^	4.75 × 10^19^	2.22 × 10^7^
Corpus Uteri	THC.Herb	10.573	0.871	7.86 × 10^−27^	6.12 × 10^−26^	4.88 × 10^−25^	2.06 × 10^8^	1.06 × 10^7^
Cervix	THC.Herb	7.048	0.692	1.79 × 10^−20^	9.64 × 10^−20^	1.04 × 10^−18^	1.15 × 10^7^	5.77 × 10^5^
Oropharynx	THC.Resin	7.056	1.081	3.30 × 10^−8^	7.96 × 10^−8^	1.38 × 10^−6^	8.17 × 10^7^	4.28 × 10^5^
Colorectum	THC.Herb	4.636	0.467	1.03 × 10^−19^	4.79 × 10^−19^	5.75 × 10^−18^	6.43 × 10^6^	3.36 × 10^5^
Myeloma	THC.Herb	4.293	1.067	7.80 × 10^−5^	1.33 × 10^−4^	2.34 × 10^−3^	1.24 × 10^10^	2.16 × 10^5^
Bladder	LM.Cannabis	12.723	3.734	7.68 × 10^−4^	1.12 × 10^−3^	1.77 × 10^−2^	5.35 × 10^11^	1.47 × 10^5^
Larynx	LM.Cannabis	21.062	6.232	8.46 × 10^−4^	1.21 × 10^−3^	1.86 × 10^−2^	4.31 × 10^11^	1.19 × 10^5^
Oesophagus	LM.Cannabis	21.862	6.703	1.27 × 10^−3^	1.74 × 10^−3^	2.53 × 10^−2^	1.73 × 10^11^	4.78 × 10^4^
Ovary	THC.Herb	6.596	0.867	6.26 × 10^−13^	2.09 × 10^−12^	3.13 × 10^−11^	2.24 × 10^5^	1.13 × 10^4^
Larynx	THC.Herb	8.671	1.231	1.95 × 10^−11^	5.93 × 10^−11^	9.35 × 10^−10^	9.27 × 10^4^	4.69 × 10^3^
Liver	THC.Herb	6.224	1.927	1.44 × 10^−3^	1.94 × 10^−3^	2.74 × 10^−2^	1.64 × 10^8^	2.64 × 10^3^
Melanoma	THC.Herb	5.221	0.810	6.37 × 10^−10^	1.72 × 10^−9^	2.87 × 10^−8^	3.72 × 10^4^	1.88 × 10^3^
Oropharynx	Income	2.004	0.345	4.46 × 10^−7^	9.75 × 10^−7^	1.74 × 10^−5^	2.90 × 10^2^	53.68
Bladder	THC.Herb	2.976	0.738	7.35 × 10^−5^	1.29 × 10^−4^	2.28 × 10^−3^	9.41 × 102	47.12
Liver	LM.Cannabis	8.543	3.824	2.66 × 10^−2^	2.86 × 10^−2^	0.1792	1.45 × 10^11^	44.49
Oesophagus	THC.Herb	5.217	1.324	1.07 × 10^−4^	1.74 × 10^−4^	0.0031	814.30	40.70
Testis	Income	0.568	0.090	1.72 × 10^−9^	4.30 × 10^−9^	7.40 × 10^−8^	18.99	9.07
Brain	THC.Herb	3.806	1.278	3.20 × 10^−3^	4.14 × 10^−3^	0.0543	188.66	9.00
Gallbladder and Biliary	Income	0.600	0.123	2.23 × 10^−6^	4.59 × 10^−6^	8.24 × 10^−5^	23.49	8.33
Thyroid	THC.Resin	1.567	0.533	3.62 × 10^−3^	4.52 × 10^−3^	0.0547	61.00	5.75
Anus	Income	0.347	0.069	1.09 × 10^−6^	2.30 × 10^−6^	4.12 × 10^−5^	11.84	5.52
Myeloma	THC.Resin	0.632	0.222	4.79 × 10^−3^	5.68 × 10^−3^	0.0575	54.80	5.11
Gallbladder and Biliary	Alcohol	0.296	0.037	1.09 × 10^−13^	4.38 × 10^−13^	5.87 × 10^−12^	6.27	4.48
Ovary	LM.Cannabis	9.010	4.381	4.08 × 10^−2^	4.14 × 10^−2^	0.1792	1.58 × 10^7^	3.76
Myeloma	Income	0.272	0.069	1.06 × 10^−4^	1.74 × 10^−4^	0.0031	7.82	3.53
Oropharynx	Tobacco	0.298	0.037	1.99 × 10^−10^	5.58 × 10^−10^	9.17 × 10^−9^	3.61	2.89
All Cancers nNMSC	THC.Resin	0.643	0.290	2.78 × 10^−2^	2.95 × 10^−2^	0.1792	43.75	2.24
Leukaemia—Myeloid	Alcohol	0.164	0.062	1.15 × 10^−2^	1.32 × 10^−2^	0.1155	5.24	1.96
Testis	Alcohol	0.126	0.035	3.89 × 10^−4^	5.80 × 10^−4^	0.0093	2.70	1.83
Hodgkin’s	THC.Resin	0.564	0.263	3.29 × 10^−2^	3.38 × 10^−2^	0.1792	23.15	1.80
Prostate	Tobacco	0.089	0.011	2.47 × 10^−13^	8.66 × 10^−13^	1.26 × 10^−11^	1.80	1.64
Corpus Uteri	Tobacco	0.085	0.016	1.05 × 10^−7^	2.45 × 10^−7^	4.31 × 10^−6^	1.59	1.43
Breast	Income	0.145	0.060	0.0172	0.0192	0.1379	2.69	1.42
Myeloma	Tobacco	0.029	0.006	2.86 × 10^−6^	5.72 × 10^−6^	1.03 × 10^−4^	1.60	1.42
Anus	Alcohol	0.066	0.027	0.0140	0.0158	0.1263	2.18	1.36
Breast	Tobacco	0.037	0.008	1.21 × 10^−5^	2.24 × 10^−5^	4.01 × 10^−4^	1.53	1.36
Hodgkin’s	Income	0.176	0.082	0.0325	0.0338	0.1792	3.75	1.35
Kidney	Tobacco	0.045	0.011	4.32 × 10^−5^	7.75 × 10^−5^	0.0014	1.50	1.32
Lung	Tobacco	0.032	0.008	0.0002	0.0003	0.0046	1.47	1.29
Hodgkin’s	Tobacco	0.023	0.007	0.0011	0.0016	0.0237	1.45	1.25
Non-Hodgkin’s Lymphoma	Tobacco	0.041	0.013	0.0016	0.0022	0.0294	1.42	1.23
Pancreas	Tobacco	0.031	0.011	0.0039	0.0048	0.0551	1.39	1.19
Colorectum	Tobacco	0.025	0.009	0.0041	0.0050	0.0551	1.39	1.19
All Cancers	Tobacco	0.039	0.017	0.0256	0.0280	0.1792	1.68	1.19
Stomach	Tobacco	0.030	0.011	0.0077	0.0089	0.0842	1.37	1.17
Ovary	Tobacco	0.033	0.016	0.0418	0.0418	0.1792	1.31	1.05

Table key: β-Estimate—estimate of the regression coefficient; Std. Error—standard error of the regression coefficient; *p*-value—significance level; P. Adj. Holm—*p*-value adjusted for multiple testing by the method of Holm; Adj. P. FDR—*p*-value adjusted for multiple testing by the false discovery rate method of Benjamini and Hochberg; E-value—expected value required of some unknown confounder covariate with both the exposure and the outcome to explain the observed effect; lower bound of the E-value—the 95% lower bound of the confidence interval of the E-value.

**Table 9:** Summary table for positive significant terms in additive panel model.

Term	Count	Negative Total of *p*-Value Exponents	Mean of the Negative *p*-Value Exponents	Median of the Negative *p*-Value Exponents	Total of the Lower E-Value Exponents	Mean of the Lower E-Value Exponents	Median of the Lower E-Value Exponents
Last Month’s Cannabis	19	189	9.95	8	341	17.95	17
Herb. THC	21	551	26.24	18	165	7.86	7
Resin. THC	5	13	2.6	2	5	1.00	0
Income	7	29	4.14	5	1	0.14	0
Alcohol	4	17	4.25	2	0	0	0
Tobacco	14	55	3.93	2.5	0	0	0

Table key: Term—Relates to the number of models which include the cited independent covariate as significant. The other columns in this table relate to the described parameters (see text).

**Table 10:** Significant positive terms from interactive panel regression.

Cancer	Term	β-Estimate	Std. Error	*p*-Value	Adj. P. FDR	Adj. P. Holm	E-Value Estimate	E-Value 95% Lower Bound
Colorectum	Herb. THC	55.387	5.081	1.17 × 10^−26^	1.13 × 10^−25^	3.14 × 10^−24^	8.51 × 10^30^	2.72 × 10^25^
Breast	Herb. THC	37.005	3.771	4.69 × 10^−22^	3.59 × 10^−21^	1.22 × 10^−19^	7.65 × 10^27^	2.43 × 10^22^
Gallbladder and Biliary	Herb. THC	20.744	2.892	1.41 × 10^−12^	6.28 × 10^−12^	3.28 × 10^−10^	1.84 × 10^26^	1.53 × 10^19^
Oropharynx_Broad	Herb. THC	23.856	4.274	4.15 × 10^8^	1.24 × 10^−7^	8.26 × 10^−6^	1.31 × 10^26^	1.17 × 10^17^
All Cancers	Herb. THC	12.034	2.019	4.45 × 10^9^	1.46 × 10^−8^	9.26 × 10^−7^	1.41 × 10^23^	4.48 × 10^15^
Thyroid	Herb. THC	20.386	3.101	7.61 × 10^−11^	2.80 × 10^−10^	1.67 × 10^−8^	2.72 × 10^21^	1.40 × 10^15^
Anus	Herb. THC	13.789	2.229	8.74 × 10^−10^	3.03 × 10^−9^	1.86 × 10^−7^	1.74 × 10^20^	8.63 × 10^13^
Testis	Herb. THC	33.843	6.241	7.22 × 10^8^	2.03 × 10^−7^	1.39 × 10^−5^	5.43 × 10^17^	2.80 × 10^11^
Stomach	Herb. THC	24.735	4.252	7.31 × 10^9^	2.28 × 10^−8^	1.49 × 10^−6^	4.63 × 10^16^	1.46 × 10^11^
Oropharynx	Resin. THC	7.349	0.625	4.34 × 10^−22^	3.40 × 10^−21^	1.13 × 10^−19^	1.92 × 10^11^	2.86 × 10^9^
Corpus Uteri	Herb. THC	25.436	5.293	1.70 × 10^6^	4.16 × 10^−6^	3.01 × 10^−4^	6.37 × 10^13^	2.03 × 10^8^
Prostate	Herb. THC	24.791	5.498	7.03 × 10^6^	1.65 × 10^−5^	1.21 × 10^−3^	9.35 × 10^12^	2.98 × 10^7^
Oesophagus	Herb. THC	13.982	3.228	1.58 × 10^5^	3.58 × 10^−5^	2.65 × 10^−3^	3.05 × 10^12^	9.59 × 10^6^
Leukaemia—Lymphoid	LM. Cannabis: Herb. THC	7.397	3.030	1.57 × 10^2^	2.59 × 10^−2^	1.0000	2.60 × 10^23^	7.91 × 10^4^
Melanoma	Herb. THC	10.965	3.151	5.17 × 10^4^	1.03 × 10^−3^	7.76 × 10^−2^	1.26 × 10^10^	3.90 × 10^4^
Cervix	Herb. THC	13.081	5.359	1.48 × 10^2^	2.46 × 10^−2^	1.00	1.47 × 10^7^	45.71
Oesophagus	Resin. THC	1.763	0.155	1.11 × 10^−28^	1.27 × 10^−27^	3.04 × 10^−26^	68.18	36.84
All Cancers nNMSC	Tobacco: Herb. THC	0.527	0.059	9.85 × 10^−19^	5.87 × 10^−18^	2.45 × 10^−16^	48.59	23.92
Oropharynx	Income	1.024	0.191	3.81 × 10^−7^	1.01 × 10^−6^	7.12 × 10^5^	67.24	18.20
Stomach	LM. Cannabis	1.283	0.096	3.16 × 10^−38^	6.28 × 10^−37^	8.98 × 10^−36^	13.60	10.06
Kidney	Herb. THC	6.010	2.758	2.95 × 10^−2^	4.60 × 10^−2^	1.00	2.69 × 10^6^	7.94
Colorectum	LM. Cannabis	1.323	0.115	2.41 × 10^−29^	3.27 × 10^−28^	6.69 × 10^−27^	10.26	7.56
Myeloma	Resin. THC	0.526	0.090	6.60 × 10^−9^	2.12 × 10^−8^	1.36 × 10^−6^	14.34	7.06
Ovary	Herb. THC	9.381	4.345	3.10 × 10^−2^	4.76 × 10^−2^	1.00	2.37 × 10^6^	6.91
Larynx	Resin. THC	0.796	0.145	4.56 × 10^−8^	1.33 × 10^−7^	8.99 × 10^−6^	10.56	5.48
Larynx	LM. Cannabis	0.612	0.068	8.34 × 10^−19^	5.08 × 10^−18^	2.09 × 10^−16^	6.93	5.05
Leukaemia—Lymphoid	Alcohol	0.164	0.021	4.81 × 10^−13^	2.24 × 10^−12^	1.13 × 10^−10^	5.96	4.29
Breast	LM. Cannabis	0.628	0.086	3.61 × 10^−13^	1.71 × 10^−12^	8.51 × 10^−11^	5.32	3.83
Thyroid	LM. Cannabis	0.433	0.065	4.51 × 10^−11^	1.75 × 10^−10^	1.00 × 10^−8^	5.07	3.57
Pancreas	Herb. THC	5.546	2.709	4.09 × 10^−2^	6.06 × 10^−2^	1.00	1.14 × 10^6^	3.00
Hodgkin’s	LM. Cannabis	0.253	0.044	1.54 × 10^−8^	4.64 × 10^−8^	3.08 × 10^−6^	4.28	2.98
Gallbladder and Biliary	Tobacco: LM. Cannabis: Herb. THC	0.266	0.034	7.85 × 10^−15^	4.10 × 10^−14^	1.90 × 10^−12^	3.72	2.95
Leukaemia—Myeloid	LM. Cannabis: Herb. THC	10.783	5.449	4.96 × 10^−2^	7.24 × 10^−2^	1.00	1.08 × 10^19^	2.67
Oropharynx	Tobacco: Herb. THC	0.509	0.187	7.31 × 10^−3^	1.27 × 10^−2^	9.36 × 10^−1^	11.00	2.66
Leukaemia—Myeloid	Alcohol	0.195	0.037	6.00 × 10^−7^	1.53 × 10^−6^	1.09 × 10^−4^	3.78	2.63
Corpus Uteri	LM. Cannabis	0.611	0.120	3.94 × 10^−7^	1.04 × 10^−6^	7.34 × 10^−5^	3.64	2.55
Gallbladder and Biliary	LM. Cannabis	0.252	0.055	5.50 × 10^−6^	1.31 × 10^−5^	9.57 × 104	3.55	2.40
Colorectum	Tobacco: LM. Cannabis: Herb. THC	0.453	0.069	5.71 × 10^−11^	2.18 × 10^−10^	1.26 × 10^−8^	2.96	2.37
Myeloma	Income	0.137	0.023	6.68 × 10^−9^	2.12 × 10^−8^	1.37 × 10^−6^	2.77	2.19
Testis	Income	0.440	0.076	7.35 × 10^−9^	2.28 × 10^−8^	1.49 × 10^−6^	2.76	2.18
Prostate	LM. Cannabis	0.512	0.125	4.20 × 10^−5^	9.14 × 10^−5^	6.81 × 10^−3^	3.05	2.08
Prostate	Income	0.372	0.054	9.01 × 10^−12^	3.63 × 10^−11^	2.03 × 10^−9^	2.47	2.08
Stomach	Tobacco: LM. Cannabis: Herb. THC	0.317	0.058	4.27 × 10^−8^	1.26 × 10^−7^	8.46 × 10^−6^	2.63	2.07
Thyroid	Tobacco: LM. Cannabis: Herb. THC	0.202	0.038	9.80 × 10^−8^	2.73 × 10^−7^	1.88 × 10^−5^	2.62	2.06
All Cancers	Alcohol	0.092	0.013	5.34 × 10^−13^	2.45 × 10^−12^	1.25 × 10^−10^	2.36	2.03
Oropharynx	Tobacco	0.148	0.033	1.44 × 10^−5^	3.27 × 10^−5^	2.42 × 10^−3^	2.71	2.00
Breast	Tobacco: LM. Cannabis: Herb. THC	0.267	0.051	1.98 × 10^−7^	5.47 × 10^−7^	3.78 × 10^−5^	2.54	2.00
Anus	Tobacco: LM. Cannabis: Herb. THC	0.138	0.027	4.91 × 10^−7^	1.26 × 10^−6^	8.99 × 10^−5^	2.55	1.98
Breast	Income	0.216	0.037	8.22 × 10^−9^	2.53 × 10^−8^	1.66 × 10^−6^	2.25	1.87
Non-Hodgkin’s Lymphoma	Tobacco: Herb. THC	0.314	0.103	2.26 × 10^−3^	4.19 × 10^−3^	3.12 × 10^−1^	3.15	1.82
Lung	Tobacco: Herb. THC	0.186	0.061	2.51 × 10^−3^	4.62 × 10^−3^	3.44 × 10^−1^	3.11	1.80
Brain	Income	0.136	0.027	7.04 × 10^−7^	1.76 × 10^−6^	1.27 × 10^−4^	2.09	1.72
Gallbladder and Biliary	Tobacco	0.072	0.005	1.16 × 10^−41^	2.89 × 10^−40^	3.34 × 10^−39^	1.76	1.68
Larynx	Alcohol	0.097	0.010	8.57 × 10^−22^	6.38 × 10^−21^	2.22 × 10^−19^	1.76	1.64
Colorectum	Resin. THC	0.604	0.242	1.28 × 10^−2^	2.14 × 10^−2^	1.00	3.74	1.64
Prostate	Tobacco: LM. Cannabis: Herb. THC	0.253	0.074	6.94 × 10^−4^	1.37 × 10^−3^	1.03 × 10^−1^	2.03	1.53
Kidney	Tobacco: LM. Cannabis: Herb. THC	0.127	0.037	7.28 × 10^−4^	1.43 × 10^−3^	1.07 × 10^−1^	2.03	1.52
Colorectum	Tobacco	0.104	0.007	1.16 × 10^−-46^	4.95 × 10^−45^	3.39 × 10^−44^	1.54	1.49
Breast	Tobacco	0.074	0.005	5.07 × 10^−43^	1.51 × 10^−41^	1.47 × 10^−40^	1.53	1.47
Oesophagus	Tobacco: LM. Cannabis: Herb. THC	0.136	0.044	1.86 × 10^−3^	3.48 × 10^−3^	2.60 × 10^−1^	1.96	1.45
Stomach	Tobacco	0.076	0.006	3.92 × 10^−37^	6.88 × 10^−36^	1.11 × 10^−34^	1.50	1.44
Colorectum	Alcohol	0.103	0.017	7.58 × 10^−10^	2.66 × 10^−9^	1.62 × 10^−7^	1.54	1.41
Pancreas	Tobacco: LM. Cannabis: Herb. THC	0.108	0.037	3.44 × 10^−3^	6.22 × 10^−3^	4.62 × 10^−1^	1.91	1.40
Ovary	LM. Cannabis	0.249	0.099	1.16 × 10^−2^	1.97 × 10^−2^	1.00	2.26	1.40
Corpus Uteri	Tobacco	0.077	0.007	1.75 × 10^−25^	1.58 × 10^−24^	4.66 × 10^−23^	1.43	1.37
Oropharynx	Tobacco: LM. Cannabis	0.060	0.020	2.75 × 10^−3^	5.02 × 10^−3^	3.74 × 10^−1^	1.76	1.37
All Cancers nNMSC	Tobacco: LM. Cannabis	0.014	0.001	1.10 × 10^−28^	1.27 × 10^−27^	3.01 × 10^−26^	1.41	1.36
Ovary	Tobacco	0.056	0.006	3.11 × 10^−20^	2.21 × 10^−19^	8.00 × 10^−18^	1.39	1.34
Prostate	Tobacco	0.068	0.008	7.41 × 10^−19^	4.60 × 10^−18^	1.86 × 10^−16^	1.38	1.33
Corpus Uteri	Tobacco: LM. Cannabis: Herb. THC	0.188	0.072	0.0085	0.0147	1.0000	1.83	1.32
Hodgkin’s	Tobacco: LM. Cannabis: Herb. THC	0.067	0.026	0.0091	0.0157	1.0000	1.84	1.31
Bladder	Resin. THC	0.266	0.125	0.0330	0.0505	1.0000	3.30	1.30
Testis	Tobacco	0.063	0.009	2.70 × 10^−11^	1.06 × 10^−10^	6.02 × 10^−9^	1.37	1.29
Prostate	Alcohol	0.075	0.018	3.02 × 10^−5^	6.71 × 10^−5^	0.0050	1.41	1.27
Oesophagus	Alcohol	0.041	0.011	1.26 × 10^−4^	2.63 × 10^−4^	0.0198	1.39	1.25
Stomach	Alcohol	0.053	0.014	1.41 × 10^−4^	2.92 × 10^−4^	0.0219	1.39	1.25
Oropharynx_Broad	Tobacco	0.037	0.012	0.0017	0.0033	0.2432	1.42	1.23
Larynx	Tobacco	0.024	0.004	9.70 × 10^−9^	2.95 × 10^−8^	1.95 × 10^−6^	1.29	1.22
Melanoma	Tobacco: LM. Cannabis	0.020	0.002	4.91 × 10^−18^	2.87 × 10^−17^	1.22 × 10^−15^	1.25	1.22
Liver	Tobacco: LM. Cannabis	0.018	0.002	3.69 × 10^−16^	2.03 × 10^−15^	9.03 × 10^−14^	1.25	1.21
Cervix	Alcohol	0.059	0.018	0.0008	0.0016	0.1181	1.36	1.21
Breast	Alcohol	0.041	0.012	0.0011	0.0020	0.1510	1.35	1.20
Lung	Tobacco: LM. Cannabis	0.011	0.001	5.55 × 10^−15^	2.95 × 10^−14^	1.35 × 10^−12^	1.23	1.20
Melanoma	Income	0.073	0.031	0.0183	0.0297	1.0000	1.60	1.19
Bladder	LM. Cannabis	0.126	0.059	0.0332	0.0505	1.0000	2.08	1.19
Pancreas	Tobacco: LM. Cannabis	0.014	0.002	1.62 × 10^−12^	7.01 × 10^−12^	3.73 × 10^−10^	1.22	1.18
Melanoma	Tobacco: LM. Cannabis: Herb. THC	0.095	0.043	0.0266	0.0420	1.0000	1.73	1.18
Oesophagus	Tobacco: LM. Cannabis	0.015	0.002	7.62 × 10^−11^	2.80 × 10^−10^	1.67 × 10^−8^	1.21	1.17
Kidney	Tobacco: LM. Cannabis	0.013	0.002	2.33 × 10^−10^	8.48 × 10^−10^	5.07 × 10^−8^	1.21	1.17
Brain	Tobacco: LM. Cannabis: Herb. THC	0.081	0.038	0.0308	0.0476	1.0000	1.71	1.15
Thyroid	Alcohol	0.027	0.011	0.0156	0.0258	1.0000	1.33	1.12
Anus	Tobacco: LM. Cannabis	0.006	0.001	6.92 × 10^−5^	0.0001	0.0110	1.16	1.11
Non-Hodgkin’s Lymphoma	Tobacco: LM. Cannabis	0.009	0.002	6.70 × 10^−5^	0.0001	0.0107	1.16	1.11
Testis	Tobacco: LM. Cannabis: Herb. THC	0.154	0.076	0.0422	0.0619	1.0000	1.69	1.09
All Cancers	Tobacco: LM. Cannabis	0.013	0.006	0.0307	0.0476	1.0000	1.31	1.08
Cervix	Tobacco	0.017	0.007	0.0192	0.0310	1.0000	1.17	1.06

Table key: β-Estimate—estimate of the regression coefficient; Std. Error—standard error of the regression coefficient; *p*-value—significance level; P. Adj. Holm—*p*-value adjusted for multiple testing by the method of Holm; Adj. P. FDR—*p*-value adjusted for multiple testing by the false discovery rate method of Benjamini and Hochberg; E-value—expected value required of some unknown confounder covariate with both the exposure and the outcome to explain the observed effect; lower bound of the E-value—the 95% lower bound of the confidence interval of the E-value.

**Table 11:** Summary of significant positive terms from interactive panel regression.

Term	Count	Negative Total of *p*-Value Exponents	Mean of the Negative *p*-Value Exponents	Median of the Negative *p*-Value Exponents	Total of the Lower E-Value Exponents	Mean of the Lower E-Value Exponents	Median of the Lower E-Value Exponents
Herb. THC	17	128	7.53	7	174	10.24	11
Resin. THC	6	60	10.00	7.5	10	1.67	0
Herb. THC: Resin. THC	2	2	1.00	1	4	2.00	2
Income	7	48	6.86	8	1	0.14	0
Last Month’s Cannabis	11	124	11.27	7	1	0.09	0
Tobacco: Herb. THC	4	24	6.00	2	1	0.25	0
Alcohol	11	76	6.91	4	0	0	0
Tobacco	12	254	21.17	18.5	0	0	0
Tobacco: Last Month’s Cann.	11	115	10.45	10	0	0	0
Tobacco: LM. Cann: Herb. THC	15	67	4.47	3	0	0	0

Table key: Term—relates to the number of models which include the cited independent covariate as significant. The other columns in this table relate to the described parameters.

**Table 12:** Summary of cancers found to be significant in multivariable models.

No.	Mixed_Effects	Panel_Additive	Panel_Interactive	Panel_2_Lags	Panel_4_Lags	Panel_6_Lags	All Models	5/6 Models
1	All Cancers	All Cancers	All Cancers	All Cancers	All Cancers	All Cancers	1	
2	All Cancers nNMSC	All Cancers nNMSC	All Cancers nNMSC	All Cancers nNMSC	All Cancers nNMSC	All Cancers nNMSC	1	
3			Anus	Anus	Anus	Anus		
4	Bladder	Bladder	Bladder	Bladder	Bladder	Bladder	1	
5		Brain	Brain	Brain	Brain	Brain		1
6	Breast	Breast	Breast	Breast	Breast	Breast	1	
7	Cervix	Cervix	Cervix		Cervix	Cervix		1
8	Colorectum	Colorectum	Colorectum	Colorectum	Colorectum	Colorectum	1	
9		Corpus Uteri	Corpus Uteri	Corpus Uteri	Corpus Uteri			
10			Gallbladder and Biliary	Gallbladder and Biliary	Gallbladder and Biliary	Gallbladder and Biliary		
11	Hodgkin’s	Hodgkin’s	Hodgkin’s	Hodgkin’s	Hodgkin’s	Hodgkin’s	1	
12	Kidney	Kidney	Kidney	Kidney	Kidney	Kidney	1	
13	Larynx	Larynx	Larynx	Larynx	Larynx	Larynx	1	
14	Leukaemia—Lymphoid	Leukaemia—Lymphoid	Leukaemia—Lymphoid					
15	Leukaemia—Myeloid		Leukaemia—Myeloid					
16	Liver	Liver	Liver	Liver		Liver		1
17		Lung	Lung	Lung	Lung	Lung		1
18	Melanoma	Melanoma	Melanoma	Melanoma	Melanoma	Melanoma	1	
19	Myeloma	Myeloma	Myeloma	Myeloma	Myeloma	Myeloma	1	
20	Non-Hodgkin’s Lymphoma	Non-Hodgkin’s Lymphoma	Non-Hodgkin’s Lymphoma	Non-Hodgkin’s Lymphoma	Non-Hodgkin’s Lymphoma	Non-Hodgkin’s Lymphoma	1	
21	Oesophagus	Oesophagus	Oesophagus	Oesophagus	Oesophagus	Oesophagus	1	
22		Oropharynx	Oropharynx	Oropharynx		Oropharynx		1
23			Oropharynx_Broad	Oropharynx_Broad	Oropharynx_Broad	Oropharynx_Broad		
24	Ovary	Ovary	Ovary	Ovary	Ovary	Ovary	1	
25	Pancreas	Pancreas	Pancreas	Pancreas	Pancreas	Pancreas	1	
26	Prostate	Prostate	Prostate	Prostate	Prostate	Prostate	1	
27	Stomach	Stomach	Stomach	Stomach	Stomach	Stomach	1	
28	Testis		Testis	Testis	Testis	Testis		1
29	Thyroid	Thyroid	Thyroid	Thyroid	Thyroid	Thyroid	1	
	**Totals**						**17**	**6**

Table key: The columns relate to the various model types listed.

## Data Availability

All data generated or analysed during this study are included in this published article and its supplementary information files. Data along with the relevant R codes have been made publicly available on the Mendeley Database Repository and can be accessed from this URL: https://data.mendeley.com//datasets/hh4456yxp4/1 (accessed on 11 December 2022). This dataset along with the relevant R code has been made publicly available and can be accessed via the Mendeley data repository via the following URLs: Raw source data: https:/data.mendeley.com/datasets/pyn8fy4gtz (accessed on 15 February 2023); Processed input data: https:/data.mendeley.com/datasets/dxc3br6863 (accessed on 15 February 2023); Major data files for main analyses: https:/data.mendeley.com/datasets/x96g53z4kk (accessed on 15 February 2023); R code for processing: https:/data.mendeley.com/datasets/v4xfnxwv3z (accessed on 15 February 2023); Background analysis: https:/data.mendeley.com/datasets/hh4456yxp4.7 (accessed on 15 February 2023).

## Supplementary Materials

The following supporting information can be downloaded at: https://www.mdpi.com/article/10.3390/jox13030024/s1. Supplementary tables: Table S1: Cancer Registry Data Sources; Table S2: Sociodemographic, Drug Exposure and Income Data for Bivariate & Multivariable Studies; Table S3: Results of Bivariate Mixed Effects Regression Models for Tobacco-Cancer; Table S4: Results of Bivariate Mixed Effects Regression Models for Alcohol -Cancer; Table S5: Results of Bivariate Linear Regression Models for Last Month Cannabis Use Content-Cancer; Table S6: Results of Bivariate Mixed Effects Regression Models for Daily Cannabis use-Cancer; Table S7: Results of Bivariate Linear Regression Models for Cannabis Herb THC Content-Cancer; Table S8: Results of Bivariate Mixed Effects Regression Models for Amphetamine use-Cancer; Table S9: Results of Bivariate Mixed Effects Regression Models for Cocaine use-Cancer; Table S10: Results of Bivariate Linear Regression Models for Cannabis Herb: Daily Use Interaction THC Content-Cancer; Table S11: Results of Bivariate Linear Regression Models for Cannabis Resin: Daily Use Interaction THC Content-Cancer; Table S12: Significant Results in Different Bivariate Models After Adjustment for Multiple Testing; Table S13: Correlation Matrix for Common Cancer—Substance Abuse Associations—Pearson Coefficients; Table S14: Correlation Matrix for Common Cancer—Substance Abuse Associations—Significance Levels; Table S15: Correlation Matrix for Common Cancer—Substance Abuse Associations—Numbers of Observations; Table S16: Ranked List of Selected Nations for Tobacco Use; Table S17: Categorical Analysis of High and Low Tobacco Using Nations—Numbers of Cases; Table S18: Ranked List of Selected Nations for Alcohol Use; Table S19: Categorical Analysis of High and Low Alcohol Using Nations—Numbers of Cases; Table S20: Categorical Analysis of High and Low Alcohol Using Nations—P, RR, AFE, PAR; Table S21: Ranked List of Selected Nations for Cannabis Use; Table S22: Categorical Analysis of High and Low Daily Cannabis Using Nations—Numbers of Cases; Table S23: Full Output from Additive Mixed Effects Model; Table S24: Positive and Significant Terms from Additive Mixed Effects Model; Table S25: Summary Table from Positive Significant Terms from Additive Mixed Effects Model; Table S26: Full Output from Additive Panel Model; Table S27: Full Output from Interactive Panel Model; Table S28: Full Output from Interactive Panel Model at Two Lags; Table S29: Positive and Significant Terms from Interactive Panel Model at Two Lags; Table S30: Summary Table from Positive Significant Terms from Interactive Panel Model Two Lags; Table S31: Full Output from Interactive Panel Model at Four Lags; Table S32: Positive and Significant Terms from Interactive Panel Model at Four Lags; Table S33: Summary Table from Positive Significant Terms from Interactive Panel Model Four Lags; Table S34: Full Output from Interactive Panel Model at Six Lags; Table S35: Positive and Significant Terms from Interactive Panel Model at Six Lags; Table S36: Summary Table from Positive Significant Terms from Interactive Panel Model Six Lags. Supplementary Figures: Figure S1: Cancer trends across time for (A) common, (B) intermediate, (C) rarer and (D) rare cancer types. Figure S2: Trends in substance use across time interpolated for (A) last month tobacco use, (B) annual alcohol consumption, (C) last year amphetamine use and (D) last year cocaine use. Figure S3: Trends across time for selected cannabis metrics. (A) Last month cannabis use, (B) daily cannabis use, (C) THC concentration of cannabis herb and (D) THC concentration of cannabis resin. Figure S4: Trends across time for selected cancer types. The log of the age standardized rate standardized to the world population 1976 is plotted on the ordinate axis. Figure S5: The log ASRw of selected cancers against last year amphetamine use. Figure S6: The log ASRw of selected cancers against last year cocaine amphetamine use.Figure S7: Correlogram as Correlation Matrix for Bivariate Relationships. Figure S8: Significance of Correlation Matrix for Bivariate Relationships. Figure S9: Semiquantitative significance testing results from the correlogram shown in Figure S7 in the main text. Figure S10: Time trends of the Log ASRw across Europe 2000–2020. Figure S11: Log of All cancer Rate across Europe over time. Figure S12: Bivariate map of All cancer rates by Log of Cannabis Herb THC Concentration Across Europe over time. Please refer to the colorplane for the interpretation of the various colour shadings. Green is where both covariates are low.Pink and purple indicates where they are both high etc. Figure S13: Bivariate map of Liver cancer rate by Log of Cannabis Herb THC Concentration Across Europe over time. Figure S14: Bivariate map of Prostate cancer rate by Log of Cannabis Herb THC Concentration Across Europe over time Figure S15: The bivariate relationship between cannabis herb THC content and colorectal cancers. Figure S16: The bivariate relationship between cannabis herb THC content and lung cancers. Figure S17: The bivariate relationship between cannabis herb THC content and rate of Non-Hodgkins Lymphoma. Figure S18: The bivariate relationship between cannabis herb THC content and lymphoid leukaemias. Figure S19: The bivariate relationship between cannabis herb THC content and vulva cancer. Figure S20: Log Rate of Selected Cancers over time by high and low cannabis use nations. (See text for details.) Figure S21: Serial Boxplots Log Rate of Selected Cancers aggregated across time by high and low cannabis use nations. (See text for details.) Figure S22: Paneled boxplot of the aggregated rates of various cancers ordered by the ratio between the rates in the high cannabis use group and that in the low cannabis use group. Figure S23: Summary plots of the additive mixed effects regression. (A) number of cancers implicated by substance, (B) the total value of the negative exponents of the *p*-values, (C) the sum of the exponents of the minimum *p*-values and (D) the average of the exponents of the minimum *p*-values. Figure S24: Graphical summary of interactive panel model at two years of lag. Figure S25: Graphical summary of interactive panel model at four years of lag. Figure S26: Graphical summary of interactive panel model at six years of lag.

## Abbreviations

Acronym	Meaning
AFE	Attributable Fraction in the Exposed
AIC	Akaike Information Criterion
ALL	Acute Lymphoid Leukaemia
AME	Average Marginal Effect
ASRe	Age Standardised Rate of cancer—European 2013 Population
ASRw	Age Standardised Rate of cancer—World Population, 1973
CI5	Cancer in Five Continents Report of IARC by the WHO
ECIS	European Cancer Information System
EMCDDA	European Monitoring Centre for Drugs and Drug Addiction
E-value	Expected Value
IARC	International Association Against Cancer
IPW	Inverse-Probability Weighting
mEV	Minimum E-value
PAF	Population Attributable Fraction
P-FDR	*p*-value Corrected for False Discovery Rate
PR	Prevalence Ratio
THC	Δ9-Tetrahydrocannabinol
