# Supplementary material for: Cannabis- and Substance-Related Carcinogenesis in Europe: A Lagged Causal Inferential Panel Regression Study
Source: J Xenobiot. 2023 Jul 18;13(3):323–85. doi: 10.3390/jox13030024 (PMC10366890; doi:10.3390/jox13030024)
Supplement: Supplementary file 1 [file jox-13-00024-s001.zip › jox-2350308-supplementary.pdf]

SUPPLEMENTARY TABLES

Table of Contents

Supplementary Table Number	Contents
S1	Cancer Registry Data Sources
S2	Sociodemographic, Drug Exposure and Income Data for Bivariate & Multivariable Studies
S3	Results of Bivariate Mixed Effects Regression Models for Tobacco-Cancer
S4	Results of Bivariate Mixed Effects Regression Models for Alcohol -Cancer
S5	Results of Bivariate Linear Regression Models for Last Month Cannabis Use Content-Cancer
S6	Results of Bivariate Mixed Effects Regression Models for Daily Cannabis use-Cancer
S7	Results of Bivariate Linear Regression Models for Cannabis Herb THC Content-Cancer
S8	Results of Bivariate Mixed Effects Regression Models for Amphetamine use-Cancer
S9	Results of Bivariate Mixed Effects Regression Models for Cocaine use-Cancer
S10	Results of Bivariate Linear Regression Models for Cannabis Herb: Daily Use Interaction THC Content-Cancer
S11	Results of Bivariate Linear Regression Models for Cannabis Resin: Daily Use Interaction THC Content-Cancer
S12	Significant Results in Different Bivariate Models After Adjustment for Multiple Testing
S13	Correlation Matrix for Common Cancer - Substance Abuse Associations - Pearson Coefficients
S14	Correlation Matrix for Common Cancer - Substance Abuse Associations - Significance Levels
S15	Correlation Matrix for Common Cancer - Substance Abuse Associations - Numbers of Observations
S16	Ranked List of Selected Nations for Tobacco Use
S17	Categorical Analysis of High and Low Tobacco Using Nations - Numbers of Cases
S18	Ranked List of Selected Nations for Alcohol Use
S19	Categorical Analysis of High and Low Alcohol Using Nations - Numbers of Cases
S20	Categorical Analysis of High and Low Alcohol Using Nations - P, RR, AFE, PAR
S21	Ranked List of Selected Nations for Cannabis Use
S22	Categorical Analysis of High and Low Daily Cannabis Using Nations - Numbers of Cases
S23	Full Output from Additive Mixed Effects Model
S24	Positive and Significant Terms from Additive Mixed Effects Model
S25	Summary Table from Positive Significant Terms from Additive Mixed Effects Model
S26	Full Output from Additive Panel Model
S27	Full Output from Interactive Panel Model
S28	Full Output from Interactive Panel Model at Two Lags
S29	Positive and Significant Terms from Interactive Panel Model at Two Lags

S30	Summary Table from Positive Significant Terms from Interactive Panel Model Two Lags
S31	Full Output from Interactive Panel Model at Four Lags
S32	Positive and Significant Terms from Interactive Panel Model at Four Lags
S33	Summary Table from Positive Significant Terms from Interactive Panel Model Four Lags
S34	Full Output from Interactive Panel Model at Six Lags
S35	Positive and Significant Terms from Interactive Panel Model at Six Lags
S36	Summary Table from Positive Significant Terms from Interactive Panel Model Six Lags

Supplementary Table S1.: Cancer and Data Sources

Country	Data Sources
Austria	Austrian National Cancer Registry
Austria	https://www.statistik.at/en/databases/statcube/statcube-statistical-database/login
Belgium	https://kankerregister.org/Annual%20Tables
Belgium	Belgian Cancer Registry. https://kankerregister.org/
Belgium	Health For All Database. WHO EURO. https://gateway.euro.who.int/en/datasets/european-health-for-all-database/
Belgium	Cancer burden in Belgium 2004-2017, Belgian Cancer Registry, Brussels 2020. https://kankerregister.org/media/docs/CancerBurdenfeb2020reduced.pdf
Estonia	https://statistika.tai.ee/pxweb/en/Andmebaas/Andmebaas__02Haigestumus__04PahaloomulisedKasvajad/?tablelist=true
Estonia	https://www.ceicdata.com/en/indicator/estonia/annual-household-income-per-capita#:~:text=Estonia%20Household%20Income%20per%20Capita%201%20Estonia%20Annual,an%20averaged%20value%20of%208%2C185.545%20USD.%20More%20items
Estonia	https://statistika.tai.ee/pxweb/en/Andmebaas/Andmebaas__02Haigestumus__04PahaloomulisedKasvajad/?tablelist=true
Europe	EPIC Centres
Europe	https://epic.iarc.fr/centers/iarc.php
Europe	European Cancer Information System
Europe	https://ecis.jrc.ec.europa.eu/
Europe	European Network of Cancer Registries
Europe	https://www.encr.eu/
France	https://www.registre-cancers-44-85.fr/donnees-cancers/
France	https://www.registre-cancers-44-85.fr/donnees-cancers/chiffres-nationaux-et-internationaux/
France	https://www.registre-cancers-44-85.fr/donnees-cancers/rapports-dincidence/
France	https://www.registre-cancers-44-85.fr/wp-content/uploads/2022/02/2021RA-epicv1618.pdf
France	https://www.registre-cancers-44-85.fr/wp-content/uploads/2022/02/2021RA-epicla1618.pdf
Germany	https://www.krebsdaten.de/Krebs/DE/Datenbankabfrage/datenbankabfrage_stufe1_node.html
Germany	https://www.krebsdaten.de/Krebs/SiteGlobals/Forms/Datenbankabfrage/EN/datenbankabfrage_stufe2_form.html
Hungary	https://stat.nrr.hu/
Ireland	Irish National Cancer Registry
Latvia	National Cancer Institute of Latvia - https://www.nvi.lt/cancer-registry/
Lithuania	https://www.nvi.lt/naujausi-duomenys/

Nertherlands	https://iknl.nl/en
Nordcan	https://nordcan.iarc.fr/en/dataviz/trends?cancers=520&sexes=1_2&populations=246_208_578_752_352&mode=population&multiple_populations=1&multiple_cancers=0&key=asr
Nordcan	Finland, Denmark, Norway, Sweden, Iceland
Poland	https://onkologia.org.pl/pl/raporty
Portugal	https://ron.min-saude.pt/pt/PesquisaSearch.aspx?q=contact
Spain	https://seom.org/images/Las_cifras_del_Cancer_en_Espana_2023.pdf
Spain	Sociedad Española de Oncologia Médica
Spain	https://www.encr.eu/
Spain	REDECAN: Red española de registros de cáncer. Estimaciones de la inciencia del cáncer en españa, 2020.
Spain	https://redecan.org/storage/documents/c1e2997b-c0f2-4cdc-bb4a-b09662b6e009.pdf
Spain	ESTIMACIONES DE LA INCIDENCIA DEL CÁNCER EN ESPAÑA, 2019
Spain	https://redecan.org/storage/documents/a1d352cb-5f15-49e9-99ef-07725d639aee.pdf
Spain	https://redecan.org/en
Spain	Registo Oncoloica Nacional, RON
Spain	https://redecan.org/en/online-statistics
Spain	https://www.isciii.es/QuienesSomos/CentrosPropios/CNE/EpidemiologiaCancerAmbiental/Paginas/Publicaciones.aspx
Spain	https://www.europeancancer.org/
Spain	https://redecan.org/en/links
Spain	https://www.jto.org/article/S1556-0864(20)30812-1/fulltext
Spain	https://www.sanidad.gob.es/en/estadEstudios/portada/home.htm
Spain	Also wrote to all 18 Spanish registries individually
Spain	https://redecan.org/en/cancer-registries
Spain	https://redecan.org/production/current/public/es/registros-de-cancer
Spain	Las_cifras_del_Cancer_en_Espana_2023.pdf (seom.org)
Spain	https://seom.org/images/Las_cifras_del_Cancer_en_Espana_2023.pdf
Spain	https://www.populationof.net/spain/
Spain	https://www.multpl.com/spain-population/table/by-year
Switzerland	https://www.nacr.ch/en/statistics-atlas/national-statistics-on-cancer-incidence/
Switzerland	https://view.officeapps.live.com/op/view.aspx?src=https%3A%2F%2Fwww.nacr.ch%2Fassets%2Ffiles%2Fuploads%2F5ye-inc-ch-sl-sa-198084-201519-website-en.xlsx&wdOrigin=BROWSELINK
Switzerland	https://www.pxweb.bfs.admin.ch/pxweb/en/px-x-1403030300_162/px-x-1403030300_162/px-x-1403030300_162.px/table/tableViewLayout2/
UK	https://www.cancerdata.nhs.uk/incidence_and_mortality
UK	https://digital.nhs.uk/data-and-information/publications/statistical/cancer-registration-statistics/england-2020

Alcohol	https://www.who.int/data/gho/data/indicators/indicator-details/GHO/total-(recorded-unrecorded)-alcohol-per-capita-(15-)-consumption
Tobacco	https://www.who.int/data/gho/data/indicators/indicator-details/GHO/gho-tobacco-control-monitor-current-tobaccouse-tobaccosmoking-cigarrettesmoking-agestd-tobagestdcurr

Supplementary Table S2.: Sociodemographic, Income and Drug Use Data
for whole Study Group for Both
Bivariate and Multivariate Studies

Measure	Bivariate	Multivariate
Sample Size	47,920	42,675
Country (%)		
Austria	354 (0.7)	314 (0.7)
Belgium	2910 (6.1)	2451 (5.7)
Bulgaria	380 (0.8)	337 (0.8)
Czechia	380 (0.8)	337 (0.8)
Denmark	600 (1.3)	520 (1.2)
Estonia	7560 (15.8)	7182 (16.8)
Finland	630 (1.3)	546 (1.3)
France	6134 (12.8)	5550 (13.0)
Germany	3704 (7.7)	3266 (7.7)
Hungary	30 (0.1)	26 (0.1)
Iceland	1006 (2.1)	880 (2.1)
Ireland	1757 (3.7)	1462 (3.4)
Italy	11086 (23.1)	9852 (23.1)
Latvia	600 (1.3)	560 (1.3)
Lithuania	130 (0.3)	120 (0.3)
Luxembourg	30 (0.1)	
Netherlands	980 (2.0)	811 (1.9)
Norway	980 (2.0)	857 (2.0)
Poland	1036 (2.2)	893 (2.1)
Portugal	1002 (2.1)	890 (2.1)
Romania	273 (0.6)	242 (0.6)
Spain	3624 (7.6)	3206 (7.5)
Sweden	630 (1.3)	546 (1.3)
United Kingdom	2104 (4.4)	1827 (4.3)
Cancer (%) (ICD10 Codes)		
All Cancers (C00-C99)	757 (1.6)	756 (1.8)
All Cancers nNMSC, (C00-C99 Excluding C44)	1524 (3.2)	1522 (3.6)
Anus (C21)	1429 (3.0)	1427 (3.3)
Bladder (C67)	1817 (3.8)	1814 (4.3)
Brain (C71)	1791 (3.7)	1788 (4.2)
Breast (C50)	1878 (3.9)	1757 (4.1)
Cervix (C53)	1791 (3.7)	1788 (4.2)
Colorectum (C18-C20)	1812 (3.8)	1808 (4.2)
Corpus Uteri (C54)	1904 (4.0)	1901 (4.5)
Gallbladder & Biliary (C23)	1216 (2.5)	1213 (2.8)
Hepatocellular, (C22; ICDO 3.2: 9170/3 - 8715/3)	62 (0.1)	

Hodgkins (C81)	1458 (3.0)	1455 (3.4)
Kaposi (C46)	151 (0.3)	151 (0.4)
Kidney (C64)	1817 (3.8)	1814 (4.3)
Larynx (C32)	1797 (3.8)	1794 (4.2)
Leukaemia (C91-C95)	1692 (3.5)	
Leukaemia - Lymphoid (C91)	373 (0.8)	373 (0.9)
Leukaemia - Myeloid (92)	394 (0.8)	394 (0.9)
Liver & Intrahepatic ducts (C22)	1420 (3.0)	1417 (3.3)
Lung (C34)	1774 (3.7)	1771 (4.1)
Medulloblastoma (C716, CIDO 9470/3 - 9472/3 & 9474/3 - 9477/3)	16 (0.0)	
Melanoma (C43)	1798 (3.8)	1795 (4.2)
Mesothelioma (C45)	172 (0.4)	
Myeloma (C90)	1438 (3.0)	1435 (3.4)
Non-Hodgkins lymphoma (C82-C86)	1735 (3.6)	1732 (4.1)
Non-Seminoma (C62, ICDO 3.2 9065/3)	38 (0.1)	
Oesophagus (C15)	1817 (3.8)	1814 (4.3)
Oropharynx (C10)	400 (0.8)	399 (0.9)
Oropharynx_Broad (C00-C14)	485 (1.0)	504 (1.2)
Ovarian Dysgerminoma (C56, ICDO 3.2 9060/3)	17 (0.0)	
Ovary (C56)	1791 (3.7)	1788 (4.2)
Pancreas (C25)	1817 (3.8)	1814 (4.3)
Penis (C60)	1346 (2.8)	
Prostate (C61)	1794 (3.7)	1791 (4.2)
Seminoma (C62, ICDO 3.2 9061/3 - 9063/3)	39 (0.1)	
Stomach (C16)	1817 (3.8)	1814 (4.3)
Testis (C62)	1413 (2.9)	1410 (3.3)
Thyroid (C73)	1439 (3.0)	1436 (3.4)
Vagina (C52)	229 (0.5)	
Vulva (C51)	234 (0.5)	
Vulva & Vagina (C51-C52)	1228 (2.6)	
ASRw (median [IQR])	7.90 [3.50, 19.34]	8.60 [4.30, 23.44]
Group, N (%)		
High	39737 (82.9%)	35527 (83.3%)
Low	8183 (17.1%)	7148 (16.7%)
Substances		
Tobacco (% Using) (mean (SD))	29.34 (6.52)	29.54 (6.55)
Tobacco Interpolated (mean (SD))	30.37 (6.05)	30.50 (6.08)
Alcohol Consumption (L/Yr) (mean (SD))	11.14 (2.46)	11.17 (2.47)
Alcohol Interpolated (mean (SD))	11.27 (2.28)	11.29 (2.29)
Last Month Cannabis Use (mean (SD))	0.04 (0.01)	0.04 (0.01)
Last Month Cannabis Interpolated (mean (SD))	0.03 (0.02)	0.03 (0.02)
Daily Cannabis Use (mean (SD))	0.01 (0.01)	0.01 (0.01)
Daily Cannabis Use Interpolated (mean (SD))	0.00 (0.01)	0.00 (0.00)
THC Content Cannabis Resin (mean (SD))	0.11 (0.07)	0.11 (0.07)

Cannabis Resin Interpolated (mean (SD))	0.06 (0.08)	0.06 (0.08)
THC Content Cannabis Herb (mean (SD))	0.10 (0.04)	0.10 (0.04)
Cannabis Herb Interpolated (mean (SD))	0.07 (0.04)	0.07 (0.04)
Amphetamine Use (Last Year %) (median [IQR])	0.40 [0.20, 0.70]	0.40 [0.20, 0.70]
Amphetamine Interpolated (mean (SD))	0.46 (0.52)	0.45 (0.51)
Cocaine Use (Last Year %) (median [IQR])	1.10 [0.80, 1.90]	1.00 [0.60, 1.90]
Cocaine Use Interpolated (median [IQR])	1.10 [0.80, 2.20]	1.10 [0.80, 2.20]
Median Household Income (median [IQR])	50502.00 [28365.00, 50502.00]	28560.67 [18506.70, 34834.70]

Supplementary Table S3.: Regression Modelling Results Including Slopes and Significance Levels and E-Values for Tobacco-Cancer Relationships

- Significant Positive Slopes at Mixed Effects Regression

**Cancer**	**β-Estimate**	**Std. Error**	**t-statistic**	**P-Value**	**Sigma**	**logLik**	**AIC**	**E-Value Estimate**	**95% Lower Bound of E-Value**
Stomach	0.018	0.002	11.510	1.45E-29	0.262	-392.259	792.519	1.33	1.29
Non-Seminoma	0.235	0.023	10.055	5.36E-12	0.664	-41.483	90.967	2.10	1.92
Larynx	0.020	0.002	8.201	4.71E-16	0.446	-1199.941	2407.881	1.25	1.21
Ovary	0.016	0.002	7.643	3.57E-14	0.370	-897.453	1802.907	1.24	1.20
Cervix	0.011	0.002	5.005	6.19E-07	0.377	-995.219	1998.438	1.20	1.15
Seminoma	0.013	0.005	2.525	0.0160	0.145	13.694	-19.387	1.38	1.15
Corpus Uteri	0.007	0.003	2.114	0.0347	0.587	-1810.787	3629.574	1.11	1.03
Oropharynx	0.013	0.008	1.593	0.1119	0.900	-536.104	1080.208	1.13	1.00
Breast	0.003	0.004	0.735	0.4626	0.901	-2509.267	5026.534	1.06	1.00
Leukaemia - Lymphoid	-0.001	0.003	-0.196	0.8443	0.308	-101.932	211.863	1.04	-
Gallbladder & Biliary	-0.001	0.003	-0.281	0.7786	0.237	-157.881	323.763	1.06	-
Medulloblastoma	-0.002	0.005	-0.420	0.6807	0.085	10.587	-13.174	1.18	-
Ovarian Dysgerminoma	-0.001	0.003	-0.449	0.6599	0.057	17.448	-26.896	1.17	-
Leukaemia - Myeloid	-0.006	0.004	-1.466	0.1434	0.497	-296.978	601.957	1.12	-
Hepatocellular	-0.010	0.004	-2.321	0.0237	0.236	-4.536	17.071	1.24	-
Vagina	-0.006	0.002	-2.502	0.0131	0.174	62.399	-116.797	1.22	-

Mesothelioma	-0.030	0.010	-3.121	0.0021	0.535	-143.442	294.883	1.29	-
Kaposi	-0.006	0.002	-3.319	0.0011	0.097	118.990	-229.981	1.31	-
Brain	-0.009	0.002	-4.103	4.28E-05	0.437	-1115.508	2239.017	1.15	-
Lung	-0.005	0.001	-4.154	3.44E-05	0.188	210.962	-413.924	1.18	-
Penis	-0.010	0.002	-4.676	3.25E-06	0.281	-281.959	571.918	1.21	-
Leukaemia	-0.017	0.003	-4.856	1.32E-06	0.543	-1528.963	3065.927	1.20	-
Hodgkins	-0.009	0.002	-5.580	2.91E-08	0.228	-42.273	92.547	1.23	-
Liver	-0.011	0.002	-5.588	2.79E-08	0.270	-355.957	719.914	1.24	-
Bladder	-0.011	0.002	-5.685	1.54E-08	0.338	-775.358	1558.716	1.21	-
Vulva & Vagina	-0.012	0.002	-6.312	3.96E-10	0.231	-79.738	167.476	1.27	-
Vulva	-0.031	0.005	-6.537	4.16E-10	0.345	-88.818	185.637	1.39	-
Anus	-0.012	0.002	-6.681	3.49E-11	0.255	-205.795	419.590	1.26	-
Thyroid	-0.017	0.002	-7.759	1.70E-14	0.295	-485.156	978.312	1.29	-
Oesophagus	-0.020	0.003	-7.763	1.43E-14	0.447	-1313.209	2634.417	1.25	-
Myeloma	-0.013	0.002	-7.790	1.35E-14	0.238	-106.338	220.677	1.28	-
Oropharynx_Broad	-0.031	0.004	-8.596	1.28E-16	0.414	-285.579	579.158	1.34	-
Pancreas	-0.015	0.002	-8.766	4.44E-18	0.296	-582.477	1172.954	1.27	-
Colorectum	-0.013	0.001	-9.212	9.29E-20	0.252	-212.852	433.705	1.27	-
Kidney	-0.017	0.002	-10.458	7.60E-25	0.271	-450.969	909.938	1.31	-

Testis	-0.021	0.002	-10.994	6.03E-27	0.266	-281.370	570.741	1.36	-
Non-Hodgkins lymphoma	-0.030	0.002	-14.664	8.68E-46	0.347	-779.942	1567.883	1.38	-
All Cancers	-0.020	0.001	-16.352	1.86E-51	0.186	138.489	-268.979	1.44	-
Prostate	-0.031	0.002	-17.437	1.15E-62	0.297	-606.050	1220.101	1.43	-
Melanoma	-0.043	0.002	-17.672	3.41E-64	0.420	-1168.005	2344.011	1.43	-
All Cancers nNMSC	-0.022	0.001	-28.345	6.07E-140	0.106	956.888	-1905.777	1.71	-

Supplementary Table S4.: Regression Modelling Results Including Slopes and Significance Levels and E-Values for Alcohol-Cancer Relationships

- Significant Positive Slopes at Mixed Effects Regression

Cancer	β-Estimate	Std.Error	P-Value	P.Adj.Holm	E-Value Estimate	E-Value Lower Bound
Stomach	0.063	0.005	1.99E-32	8.15E-31	1.80	1.70
Larynx	0.060	0.007	6.93E-16	2.70E-14	1.51	1.42
Oesophagus	0.066	0.008	1.97E-15	7.50E-14	1.54	1.45
Liver	0.045	0.006	1.94E-12	7.19E-11	1.61	1.48
Myeloma	0.034	0.005	5.05E-11	1.77E-09	1.54	1.42
Cervix	0.047	0.007	1.73E-10	5.89E-09	1.49	1.38
Prostate	0.040	0.006	2.86E-10	9.43E-09	1.48	1.37
Breast	0.067	0.011	7.40E-10	2.30E-08	1.35	1.27
Ovary	0.039	0.007	3.10E-09	9.31E-08	1.44	1.33
Lung	0.020	0.004	9.49E-08	2.75E-06	1.44	1.32
Leukaemia - Lymphoid	0.045	0.009	3.64E-07	9.82E-06	1.56	1.40
Colorectum	0.013	0.004	3.56E-03	7.48E-02	1.27	1.14
Vagina	0.020	0.010	4.20E-02	7.99E-01	1.45	1.07
Leukaemia - Myeloid	0.024	0.014	9.50E-02	1.00E+00	1.26	1.00
All Cancers nNMSC	0.005	0.004	1.31E-01	1.00E+00	1.23	1.00
All Cancers	0.005	0.005	3.32E-01	1.00E+00	1.17	1.00
Kidney	0.005	0.006	3.35E-01	1.00E+00	1.15	1.00
Penis	0.003	0.005	5.61E-01	1.00E+00	1.11	1.00
Vulva	0.010	0.019	5.83E-01	1.00E+00	1.19	1.00
Corpus Uteri	0.002	0.010	8.46E-01	1.00E+00	1.06	1.00
Pancreas	0.001	0.006	8.57E-01	1.00E+00	1.06	1.00
Non-Hodgkins lymphoma	0.000	0.007	9.88E-01	1.00E+00	1.02	-
Non-Seminoma	-0.032	0.424	9.41E-01	1.00E+00	1.17	-
Anus	-0.002	0.005	7.04E-01	1.00E+00	1.09	-
Kaposi	-0.004	0.008	6.48E-01	1.00E+00	1.22	-
Ovarian Dysgerminoma	-0.010	0.014	4.84E-01	1.00E+00	1.64	-
Oropharynx_Broad	-0.018	0.016	2.50E-01	1.00E+00	1.24	-
Vulva & Vagina	-0.008	0.006	1.84E-01	1.00E+00	1.22	-
Hepatocellular	-0.023	0.017	1.78E-01	1.00E+00	1.41	-
Medulloblastoma	-0.037	0.027	1.91E-01	1.00E+00	2.43	-
Leukaemia	-0.015	0.011	1.64E-01	1.00E+00	1.18	-
Brain	-0.016	0.006	5.43E-03	1.09E-01	1.22	-
Mesothelioma	-0.114	0.035	1.34E-03	2.94E-02	1.72	-
Seminoma	-0.088	0.025	1.13E-03	2.59E-02	3.01	-
Hodgkins	-0.017	0.005	1.92E-04	4.60E-03	1.35	-
Melanoma	-0.036	0.008	1.11E-05	2.78E-04	1.36	-
Gallbladder & Biliary	-0.036	0.007	2.01E-06	5.22E-05	1.56	-
Thyroid	-0.036	0.007	1.68E-07	4.69E-06	1.47	-
Testis	-0.037	0.006	7.00E-10	2.24E-08	1.51	-
Oropharynx	-0.179	0.025	2.90E-12	1.04E-10	1.72	-

Bladder	-0.056	0.006	4.89E-20	1.96E-18	1.60	-

Supplementary Table S5.: Regression Modelling Results Including Slopes and Significance Levels and E-Values for Alcohol-Cancer Relationships

- Significant Positive Slopes at Mixed Effects Regression

Cancer	ß-Estimate	Std.Error	P_Value	P.Adj.Holm	E-Value Estimate	E-Value Lower Bound
Larynx	13.9624	1.0975	8.82E-30	2.47E-28	5.37E+20	3.88E+17
Oesophagus	7.4666	0.9646	1.64E-13	4.10E-12	5.43E+12	3.92E+09
Leukaemia - Myeloid	14.1710	1.8814	1.75E-11	4.02E-10	4.42E+14	8.32E+10
Lung	5.7026	0.7822	2.99E-12	7.18E-11	1.34E+12	8.98E+08
Myeloma	3.6037	0.6921	3.63E-07	6.54E-06	4.78E+08	3.41E+05
Leukaemia - Lymphoid	5.4028	1.1279	5.50E-06	9.34E-05	2.82E+09	5.18E+05
Gallbladder & Biliary	4.5657	1.6295	5.57E-03	8.91E-02	1.63E+11	3.93E+03
Colorectum	2.7213	1.0322	8.82E-03	1.32E-01	2.37E+04	2.20E+01
Kidney	1.1616	0.5289	2.89E-02	3.75E-01	7.36E+03	4.35E+00
Stomach	2.0769	1.0365	4.60E-02	5.52E-01	3.43E+03	1.67E+00
Hodgkins	1.1497	0.6256	6.70E-02	7.37E-01	1.64E+03	1.00E+00
Oropharynx_Broad	1.2403	1.4225	3.89E-01	1.00E+00	1.38E+05	1.00E+00
Liver	0.7396	1.0515	4.82E-01	1.00E+00	2.75E+01	1.00E+00
All Cancers nNMSC	0.2159	0.5022	6.68E-01	1.00E+00	8.35E+01	1.00E+00
Anus	0.1794	0.8052	8.24E-01	1.00E+00	3.99E+00	1.00E+00
Non-Hodgkins lymphoma	-0.7235	1.1659	5.35E-01	1.00E+00	1.96E+01	-
Thyroid	-0.8420	1.1835	4.77E-01	1.00E+00	2.72E+01	-
Cervix	-1.5008	1.2630	2.36E-01	1.00E+00	1.59E+02	-
Kaposi	-0.3104	0.2339	1.99E-01	1.00E+00	4.67E+05	-
Bladder	-1.3475	0.8408	1.10E-01	9.91E-01	7.05E+02	-
Pancreas	-1.1066	0.6108	7.11E-02	7.37E-01	1.74E+03	-
All Cancers	-1.1866	0.5229	2.50E-02	3.50E-01	1.79E+04	-
Testis	-5.5780	1.0226	1.06E-07	2.01E-06	1.14E+09	-
Melanoma	-8.0840	1.4581	6.63E-08	1.33E-06	1.04E+09	-
Corpus Uteri	-7.6375	1.3293	2.30E-08	4.84E-07	3.43E+09	-
Ovary	-5.3495	0.8948	6.65E-09	1.46E-07	7.65E+09	-
Oropharynx	-30.6373	3.1850	1.85E-16	4.81E-15	1.22E+18	-
Breast	-5.9859	0.5782	1.46E-21	3.95E-20	1.24E+17	-
Brain	-13.5135	0.9459	2.26E-35	6.57E-34	2.52E+23	-
Prostate	-17.2115	1.0063	9.55E-46	2.87E-44	1.19E+28	-

Supplementary Table S6.: Regression Modelling Results Including Slopes and Significance Levels and E-Values for Last Month Cannabis-Cancer Relationships
- Significant Positive Slopes at Mixed Effects Regression

Cancer	ß-Estimate	Std.Error	P_Value	P.Adj.Holm	E-Value Estimate	E-Value Lower Bound
Larynx	13.9624	1.0975	8.82E-30	2.47E-28	5.37E+20	3.88E+17
Oesophagus	7.4666	0.9646	1.64E-13	4.10E-12	5.43E+12	3.92E+09
Leukaemia - Myeloid	14.1710	1.8814	1.75E-11	4.02E-10	4.42E+14	8.32E+10
Lung	5.7026	0.7822	2.99E-12	7.18E-11	1.34E+12	8.98E+08
Myeloma	3.6037	0.6921	3.63E-07	6.54E-06	4.78E+08	3.41E+05
Leukaemia - Lymphoid	5.4028	1.1279	5.50E-06	9.34E-05	2.82E+09	5.18E+05
Gallbladder & Biliary	4.5657	1.6295	5.57E-03	8.91E-02	1.63E+11	3.93E+03
Colorectum	2.7213	1.0322	8.82E-03	1.32E-01	2.37E+04	2.20E+01
Kidney	1.1616	0.5289	2.89E-02	3.75E-01	7.36E+03	4.35E+00
Stomach	2.0769	1.0365	4.60E-02	5.52E-01	3.43E+03	1.67E+00
Hodgkins	1.1497	0.6256	6.70E-02	7.37E-01	1.64E+03	1.00E+00
Oropharynx_Broad	1.2403	1.4225	3.89E-01	1.00E+00	1.38E+05	1.00E+00
Liver	0.7396	1.0515	4.82E-01	1.00E+00	2.75E+01	1.00E+00
All Cancers nNMSC	0.2159	0.5022	6.68E-01	1.00E+00	8.35E+01	1.00E+00
Anus	0.1794	0.8052	8.24E-01	1.00E+00	3.99E+00	1.00E+00
Non-Hodgkins lymphoma	-0.7235	1.1659	5.35E-01	1.00E+00	1.96E+01	-
Thyroid	-0.8420	1.1835	4.77E-01	1.00E+00	2.72E+01	-
Cervix	-1.5008	1.2630	2.36E-01	1.00E+00	1.59E+02	-
Kaposi	-0.3104	0.2339	1.99E-01	1.00E+00	4.67E+05	-
Bladder	-1.3475	0.8408	1.10E-01	9.91E-01	7.05E+02	-
Pancreas	-1.1066	0.6108	7.11E-02	7.37E-01	1.74E+03	-
All Cancers	-1.1866	0.5229	2.50E-02	3.50E-01	1.79E+04	-
Testis	-5.5780	1.0226	1.06E-07	2.01E-06	1.14E+09	-
Melanoma	-8.0840	1.4581	6.63E-08	1.33E-06	1.04E+09	-
Corpus Uteri	-7.6375	1.3293	2.30E-08	4.84E-07	3.43E+09	-
Ovary	-5.3495	0.8948	6.65E-09	1.46E-07	7.65E+09	-
Oropharynx	-30.6373	3.1850	1.85E-16	4.81E-15	1.22E+18	-
Breast	-5.9859	0.5782	1.46E-21	3.95E-20	1.24E+17	-
Brain	-13.5135	0.9459	2.26E-35	6.57E-34	2.52E+23	-
Prostate	-17.2115	1.0063	9.55E-46	2.87E-44	1.19E+28	-

Supplementary Table S7.: Regression Modelling Results Including Slopes and Significance Levels and E-Values for THC concentration of Cannabis Herb-Cancer Relationships

- Significant Positive Slopes at Linear Regression

Cancer	ß-Estimate	Std.Error	P_Value	P.Adj.Holm	E-Value Estimate	E-Value Lower Bound
Seminoma	6.0276	0.0678	3.97E-24	9.14E-23	Inf	Inf
All Cancers nNMSC	3.9813	0.1587	1.19E-114	4.39E-113	2.98E+06	9.84E+05
Prostate	6.4439	0.2773	8.42E-103	3.03E-101	5.85E+05	2.03E+05
Kidney	6.5740	0.3027	1.07E-91	3.73E-90	2.45E+05	8.52E+04
Melanoma	6.5178	0.3088	3.42E-87	1.16E-85	1.82E+05	6.32E+04
Lung	4.4753	0.2126	9.36E-87	3.09E-85	1.79E+05	6.19E+04
Pancreas	5.6047	0.2852	4.47E-77	1.43E-75	8.03E+04	2.80E+04
Testis	4.6650	0.2423	5.39E-72	1.67E-70	1.44E+06	3.66E+05
Oesophagus	5.9347	0.3633	1.49E-55	4.48E-54	1.34E+04	4.68E+03
Anus	3.6486	0.2289	5.30E-52	1.54E-50	1.42E+05	3.61E+04
Gallbladder & Biliary	-5.0564	0.3274	1.14E-48	3.20E-47	1.82E+05	-
Non-Hodgkins lymphoma	3.4585	0.2494	3.14E-41	8.48E-40	3.90E+03	1.34E+03
Cervix	5.3564	0.3994	7.07E-39	1.84E-37	2.86E+03	9.90E+02
All Cancers	3.5844	0.3048	5.17E-29	1.29E-27	3.64E+04	7.12E+03
Leukaemia - Myeloid	8.0929	0.7028	1.33E-24	3.19E-23	5.89E+07	3.17E+06
Hepatocellular	-14.9401	1.4736	2.51E-09	3.76E-08	2.51E+73	-
Ovary	3.0285	0.3050	1.44E-22	3.16E-21	4.33E+02	1.50E+02
Oropharynx	-12.8760	1.3853	4.71E-18	9.42E-17	9.45E+05	-
Breast	4.1776	0.4567	1.73E-19	3.63E-18	2.59E+02	9.13E+01
Leukaemia - Lymphoid	3.3598	0.4196	5.55E-14	1.06E-12	3.17E+05	1.70E+04
Stomach	2.6638	0.3901	1.21E-11	2.19E-10	7.91E+01	2.72E+01
Vagina	2.0475	0.3207	1.72E-09	2.93E-08	1.26E+06	2.10E+04

Myeloma	1.2672	0.2103	2.23E-09	3.57E-08	1.31E+02	3.33E+01
Liver	-2.1072	0.3926	9.59E-08	1.34E-06	8.73E+01	-
Bladder	-1.4861	0.2809	1.39E-07	1.80E-06	3.42E+01	-
Larynx	-1.6197	0.3108	2.13E-07	2.56E-06	3.27E+01	-
Oropharynx_Broad	2.3735	0.4968	2.42E-06	2.66E-05	2.23E+02	3.19E+01
Brain	0.9826	0.2818	5.01E-04	5.01E-03	1.27E+01	4.01E+00
Kaposi	2.8692	0.8767	1.51E-03	1.36E-02	2.96E+04	9.47E+01
Corpus Uteri	-1.2291	0.4552	7.01E-03	5.61E-02	7.64E+00	-
Mesothelioma	1.6450	0.7649	3.37E-02	2.36E-01	3.00E+02	2.52E+00
Thyroid	0.6214	0.4363	1.55E-01	9.28E-01	4.82E+00	1.00E+00
Hodgkins	-0.3068	0.2307	1.84E-01	9.28E-01	4.40E+00	-
Penis	-0.1852	0.2347	4.30E-01	1.00E+00	2.92E+00	-
Colorectum	0.0966	0.1847	6.01E-01	1.00E+00	1.98E+00	1.00E+00
Non-Seminoma	-2.4302	5.5344	6.65E-01	1.00E+00	4.53E+01	-
Leukaemia	0.1491	0.4996	7.65E-01	1.00E+00	1.63E+00	1.00E+00

Supplementary Table S8.: Regression Modelling Results Including Slopes and Significance Levels and E-Values for Amphetamine Use Cannabis-Cancer Relationships

- Significant Positive Slopes at Mixed Effects Regression

Cancer	ß-Estimate	Std.Error	P_Value	P.Adj.Holm	E-Value Estimate	E-Value Lower Bound
Gallbladder & Biliary	0.3018	0.0392	6.34E-14	1.52E-12	5.78E+00	4.16E+00
Leukaemia - Myeloid	0.2624	0.0389	1.37E-10	2.75E-09	3.39E+00	2.64E+00
Bladder	0.0903	0.0249	3.01E-04	3.62E-03	2.11E+00	1.60E+00
Larynx	0.0145	0.0255	5.70E-01	1.00E+00	1.28E+00	1.00E+00
Stomach	-0.0071	0.0195	7.16E-01	1.00E+00	1.22E+00	-
Corpus Uteri	-0.0318	0.0249	2.01E-01	6.02E-01	1.50E+00	-
Pancreas	-0.0241	0.0161	1.35E-01	5.39E-01	1.54E+00	-
Cervix	-0.0451	0.0272	9.80E-02	4.90E-01	1.60E+00	-
Thyroid	-0.0607	0.0329	6.56E-02	3.93E-01	1.64E+00	-
Oropharynx_Broad	-0.0756	0.0288	1.05E-02	7.32E-02	4.12E+00	-
Leukaemia - Lymphoid	-0.0727	0.0250	4.00E-03	3.20E-02	2.03E+00	-
Ovary	-0.0595	0.0194	2.22E-03	2.00E-02	1.96E+00	-
Anus	-0.0735	0.0214	6.16E-04	6.16E-03	2.05E+00	-
Oropharynx	-0.4242	0.1182	4.17E-04	4.58E-03	2.21E+00	-
Hodgkins	-0.0851	0.0188	6.69E-06	9.36E-05	2.33E+00	-
Kaposi	-0.0356	0.0076	1.79E-05	2.33E-04	2.98E+00	-
Kidney	-0.0687	0.0144	2.05E-06	3.08E-05	2.45E+00	-
Liver	-0.1392	0.0287	1.54E-06	2.46E-05	2.47E+00	-
Testis	-0.1376	0.0248	4.14E-08	7.04E-07	2.71E+00	-
Lung	-0.1042	0.0183	1.87E-08	3.37E-07	2.68E+00	-
Brain	-0.1413	0.0240	6.03E-09	1.15E-07	2.64E+00	-
Melanoma	-0.2016	0.0288	6.16E-12	1.29E-10	3.15E+00	-
Oesophagus	-0.1690	0.0239	3.97E-12	8.73E-11	3.19E+00	-

Myeloma	-0.1327	0.0175	9.64E-14	2.22E-12	3.36E+00	-
Non-Hodgkins lymphoma	-0.1776	0.0208	9.38E-17	2.34E-15	3.88E+00	-
Colorectum	-0.1956	0.0205	2.41E-20	6.28E-19	4.10E+00	-
All Cancers nNMSC	-0.1148	0.0111	9.13E-23	2.47E-21	8.80E+00	-
All Cancers	-0.1113	0.0098	1.07E-23	2.99E-22	5.66E+00	-
Breast	-0.1556	0.0127	2.30E-31	6.67E-30	5.86E+00	-
Prostate	-0.3794	0.0242	2.14E-47	6.43E-46	7.90E+00	-

Supplementary Table S9.: Regression Modelling Results Including Slopes and Significance Levels and E-Values for Cocaine Use Cannabis-Cancer Relationships

- Significant Positive Slopes at Mixed Effects Regression

-

Cancer	ß-Estimate	Std.Error	P_Value	P.Adj.Holm	E-Value Estimate	E-Value Lower Bound
Melanoma	0.3026	0.0252	3.28E-31	9.52E-30	4.76E+00	3.96E+00
All Cancers nNMSC	0.0767	0.0071	1.06E-25	2.98E-24	5.91E+00	4.66E+00
Thyroid	0.2568	0.0256	9.71E-23	2.62E-21	3.84E+00	3.19E+00
Hodgkins	0.1651	0.0170	1.81E-21	4.70E-20	3.26E+00	2.76E+00
Liver	0.1904	0.0211	8.78E-19	2.11E-17	3.48E+00	2.88E+00
Non-Hodgkins lymphoma	0.1604	0.0186	2.49E-17	5.72E-16	3.54E+00	2.90E+00
Anus	0.1760	0.0205	2.89E-17	6.35E-16	3.28E+00	2.72E+00
Testis	0.1757	0.0222	6.57E-15	1.31E-13	2.99E+00	2.48E+00
Myeloma	0.1173	0.0158	2.09E-13	3.96E-12	2.86E+00	2.37E+00
Bladder	0.1284	0.0184	4.74E-12	8.54E-11	2.85E+00	2.33E+00
Colorectum	0.1003	0.0149	3.01E-11	5.12E-10	2.54E+00	2.11E+00
Pancreas	0.0884	0.0145	1.33E-09	2.13E-08	2.73E+00	2.19E+00
Oropharynx_Broad	0.2256	0.0370	2.26E-08	2.94E-07	4.64E+00	3.23E+00
All Cancers	0.0850	0.0142	7.83E-09	1.17E-07	3.55E+00	2.64E+00
Breast	0.0517	0.0112	4.00E-06	4.40E-05	2.28E+00	1.79E+00
Lung	0.0667	0.0147	6.21E-06	6.21E-05	2.25E+00	1.77E+00
Kidney	0.0566	0.0137	3.72E-05	3.35E-04	2.18E+00	1.69E+00
Gallbladder & Biliary	0.0851	0.0219	1.10E-04	8.81E-04	2.16E+00	1.65E+00
Kaposi	0.0192	0.0111	8.93E-02	5.36E-01	1.96E+00	1.00E+00
Brain	0.0271	0.0168	1.08E-01	5.40E-01	1.43E+00	1.00E+00
Oropharynx	0.2084	0.1487	1.63E-01	5.75E-01	1.66E+00	1.00E+00
Leukaemia - Myeloid	-0.0172	0.0479	7.20E-01	8.52E-01	1.24E+00	-
Oesophagus	-0.0176	0.0222	4.26E-01	8.52E-01	1.34E+00	-

Prostate	-0.0311	0.0213	1.44E-01	5.75E-01	1.50E+00	-
Corpus Uteri	-0.0524	0.0192	6.40E-03	4.48E-02	1.79E+00	-
Leukaemia - Lymphoid	-0.1601	0.0289	8.13E-08	9.76E-07	3.23E+00	-
Larynx	-0.1211	0.0214	1.86E-08	2.61E-07	2.47E+00	-
Cervix	-0.1994	0.0239	2.16E-16	4.53E-15	3.24E+00	-
Ovary	-0.1556	0.0167	6.59E-20	1.65E-18	3.44E+00	-
Stomach	-0.2627	0.0175	1.25E-46	3.74E-45	6.47E+00	-

Supplementary Table S10.: Regression Modelling Results Including Slopes and Significance Levels and E-Values for Cannabis Herb THC : Daily Cannabis Use Interaction Cannabis-Cancer Relationships

- Significant Positive Slopes at Mixed Effects Regression

Cancer	ß-Estimate	Std.Error	P_Value	P.Adj.Holm	E-Value Estimate	E-Value Lower Bound
Anus	412.7268	37.2436	4.32E-22	1.30E-20	Inf	Inf
Thyroid	526.6490	61.0909	3.27E-15	9.47E-14	Inf	Inf
Liver	426.3964	59.0183	1.86E-11	4.84E-10	Inf	Inf
Melanoma	309.2841	48.0528	1.06E-09	2.66E-08	Inf	Inf
Non-Hodgkins lymphoma	199.1670	34.2164	2.70E-08	6.47E-07	Inf	Inf
Myeloma	134.4610	26.5136	9.72E-07	2.14E-05	Inf	Inf
Oesophagus	182.1030	46.9719	1.48E-04	2.96E-03	Inf	Inf
All Cancers	85.8550	22.6303	3.35E-04	6.04E-03	Inf	Inf
Hodgkins	110.2881	29.6441	2.58E-04	4.91E-03	Inf	4.85E+134
Breast	100.7892	29.4649	7.75E-04	1.24E-02	Inf	9.95E+119
Leukaemia - Lymphoid	305.3433	91.4881	1.75E-03	2.63E-02	Inf	Inf
Lung	113.2827	36.1718	2.03E-03	2.84E-02	Inf	3.70E+99
Testis	155.4019	52.8126	3.69E-03	4.80E-02	Inf	3.02E+89
Kidney	57.6144	23.8980	1.69E-02	2.03E-01	Inf	2.30E+40
All Cancers nNMSC	32.1477	15.9036	4.56E-02	4.56E-01	Inf	2.13E+09
Leukaemia - Myeloid	349.5884	174.0222	5.09E-02	4.56E-01	Inf	1.57E+06
Pancreas	47.9978	24.3399	5.01E-02	4.56E-01	Inf	3.81E+01
Kaposi	302.5919	213.0733	2.51E-01	1.00E+00	Inf	1.00E+00
Prostate	39.8643	54.1304	4.62E-01	1.00E+00	1.15E+55	1.00E+00
Oropharynx	-115.0968	241.5488	6.36E-01	1.00E+00	5.69E+55	-
Larynx	-29.0589	47.9380	5.45E-01	1.00E+00	8.33E+45	-
Cervix	-58.6212	54.1668	2.81E-01	1.00E+00	1.51E+89	-

Colorectum	-82.0065	57.2284	1.54E-01	9.22E-01	8.79E+115	-
Brain	-79.3731	46.6710	9.07E-02	6.35E-01	1.91E+127	-
Oropharynx_Broad	-60.6127	27.7363	3.51E-02	3.86E-01	Inf	-
Corpus Uteri	-310.2456	85.6156	3.77E-04	6.40E-03	Inf	-
Bladder	-218.4021	45.9669	4.10E-06	8.62E-05	Inf	-
Gallbladder & Biliary	-223.5176	38.2502	2.92E-08	6.71E-07	Inf	-
Ovary	-302.7960	40.8986	5.09E-12	1.37E-10	Inf	-
Stomach	-362.4852	43.7580	2.59E-14	7.24E-13	Inf	-

Supplementary Table S11.: Regression Modelling Results Including Slopes and Significance Levels and E-Values for

- Cannabis Resin THC : Daily Cannabis Use Interaction Cannabis-Cancer Relationships
- Significant Positive Slopes at Mixed Effects Regression

Cancer	ß-Estimate	Std.Error	P_Value	P.Adj.Holm	E-Value Estimate	E-Value Lower Bound
Melanoma	123.8679	15.5947	3.30E-13	8.90E-12	Inf	Inf
Oesophagus	89.5415	13.6501	7.10E-10	1.78E-08	Inf	Inf
Liver	108.9451	25.6312	3.86E-05	7.33E-04	Inf	1.30E+94
Hodgkins	48.8718	11.9709	6.71E-05	1.21E-03	8.39E+129	6.59E+67
All Cancers	24.2464	6.2438	3.50E-04	5.60E-03	3.18E+153	2.15E+76
Bladder	63.6961	19.2668	1.17E-03	1.64E-02	3.08E+115	2.17E+47
Oropharynx	570.5800	173.0068	2.58E-03	2.65E-02	Inf	1.96E+130
All Cancers nNMSC	23.1056	7.2198	1.79E-03	2.15E-02	Inf	1.35E+86
Myeloma	28.7010	9.3067	2.41E-03	2.65E-02	5.52E+107	3.94E+39
Lung	24.0030	10.5493	2.42E-02	1.94E-01	2.22E+78	1.79E+11
Anus	21.5360	16.3543	1.90E-01	1.00E+00	7.88E+44	1.00E+00
Non-Hodgkins lymphoma	8.6548	8.9775	3.37E-01	1.00E+00	1.18E+34	1.00E+00
Thyroid	24.6482	27.3899	3.70E-01	1.00E+00	6.66E+31	1.00E+00
Oropharynx_Broad	-3.0714	6.4918	6.39E-01	1.00E+00	1.34E+19	-
Pancreas	-5.4923	8.9133	5.39E-01	1.00E+00	7.53E+20	-
Brain	-10.0795	14.1286	4.77E-01	1.00E+00	4.68E+21	-
Kidney	-9.4814	9.8204	3.36E-01	1.00E+00	5.44E+33	-
Breast	-32.1319	10.9960	3.99E-03	3.59E-02	4.28E+98	-
Gallbladder & Biliary	-41.8260	12.5018	1.03E-03	1.55E-02	1.83E+120	-
Leukaemia - Lymphoid	-145.5173	40.7427	1.62E-03	2.10E-02	Inf	-
Leukaemia - Myeloid	-424.0653	95.1826	1.81E-04	3.08E-03	Inf	-
Larynx	-69.6941	14.6597	4.41E-06	8.83E-05	Inf	-
Testis	-90.9015	18.9708	3.80E-06	7.99E-05	2.98E+148	-

Corpus Uteri	-168.5215	32.3178	5.59E-07	1.23E-05	Inf	-
Prostate	-100.5291	19.0616	4.36E-07	1.00E-05	Inf	-
Cervix	-119.8792	20.0130	1.38E-08	3.31E-07	Inf	-
Colorectum	-121.2823	15.7807	1.48E-12	3.85E-11	Inf	-
Ovary	-157.9161	13.5064	4.11E-23	1.15E-21	Inf	-
Stomach	-229.6094	13.8821	1.83E-36	5.31E-35	Inf	-

Supplementary Table S12.: Collated Results of Bivariate Regressions
For Cannabis Metrics by Model Type
Constructed with Significance Levels (P-Values) Adjusted for Multiple Testing

Last Month	Daily	Herb	Resin
	All Cancers	All Cancers	All Cancers
		All Cancers nNMSC	All Cancers nNMSC
	Anus	Anus	Anus
			Bladder
		Brain	
		Breast	Breast
		Colorectum	
		Corpus Uteri	
	Hodgkins		
		Kidney	Kidney
Larynx			
	Liver		
Leukaemia - Lymphoid		Leukaemia - Lymphoid	
Leukaemia - Myeloid		Leukaemia - Myeloid	
Lung	Lung	Lung	Lung
	Melanoma	Melanoma	Melanoma
Myeloma	Myeloma	Myeloma	
	Non-Hodgkins lymphoma	Non-Hodgkins lymphoma	Non-Hodgkins lymphoma
Oesophagus	Oesophagus	Oesophagus	Oesophagus
			Oropharynx
		Oropharynx_Broad	Oropharynx_Broad
		Pancreas	Pancreas
		Prostate	

		Testis	Testis
	Thyroid	Thyroid	

Supplementary Table S13: Correlation Matrix for Common Cancers
With Selected Substances – Pearson Correlation Coefficients

All Cancers nNMSC	Breast	Colorectum	Lung	Non-Hodgkins lymphoma	Prostate	Tobacco	Alcohol	Last_Month_Cann	Daily_Cannabis	Herb_THC	Resin_THC	Amphetamines	Cocaine
1	0.6151	0.2717	0.7955	0.6784	0.7808	0.2665	0.3290	-0.0725	0.2030	0.4928	0.2267	-0.0724	0.1550
0.6151	1	0.1515	0.3799	0.4535	0.5125	-0.0857	0.1049	-0.0246	0.1206	0.5164	0.3911	-0.0912	0.0687
0.2717	0.1515	1	0.1132	0.1943	0.1615	-0.0518	0.0187	0.2520	-0.3660	0.0170	-0.1862	-0.0516	0.2456
0.7955	0.3799	0.1132	1	0.4543	0.4919	0.3712	0.4025	0.4409	0.0686	0.4485	0.2139	0.1519	-0.1549
0.6784	0.4535	0.1943	0.4543	1	0.4003	-0.0742	0.0385	-0.1226	0.3533	0.3340	0.2397	-0.0844	0.3519
0.7808	0.5125	0.1615	0.4919	0.4003	1	0.3358	0.4780	-0.3917	0.0231	0.4754	0.1893	-0.4060	-0.3247
0.2665	-0.0857	-0.0518	0.3712	-0.0742	0.3358	1	0.6836	0.2197	-0.5526	-0.0721	-0.3106	-0.5273	-0.7240
0.3290	0.1049	0.0187	0.4025	0.0385	0.4780	0.6836	1	0.3587	-0.5916	0.2561	-0.1407	-0.0774	-0.7920
-0.0725	-0.0246	0.2520	0.4409	-0.1226	-0.3917	0.2197	0.3587	1	0.1014	-0.2927	-0.2926	0.2801	0.0697
0.2030	0.1206	-0.3660	0.0686	0.3533	0.0231	-0.5526	-0.5916	0.1014	1	0.1154	0.6512	0.2028	0.7795
0.4928	0.5164	0.0170	0.4485	0.3340	0.4754	-0.0721	0.2561	-0.2927	0.1154	1	0.7347	0.4083	-0.1574
0.2267	0.3911	-0.1862	0.2139	0.2397	0.1893	-0.3106	-0.1407	-0.2926	0.6512	0.7347	1	0.2118	-0.0674

-0.0724	-0.0912	-0.0516	0.1519	-0.0844	-0.4060	-0.5273	-0.0774	0.2801	0.2028	0.4083	0.2118	1	0.2415
0.1550	0.0687	0.2456	-0.1549	0.3519	-0.3247	-0.7240	-0.7920	0.0697	0.7795	-0.1574	-0.0674	0.2415	1

Supplementary Table S14: Correlation Matrix for Common Cancers
With Selected Substances – Significance Levels (P-Values) of Correlation Coefficients

Parametert	All Cancers nNMSC	Breast	Colorectum	Lung	Non-Hodgkins lymphoma	Prostate	Tobacco	Alcohol	Last_Month_Cann	Daily_Cannabis	Herb_THC	Resin_THC	Amphetamines	Cocaine
**All Cancers nNMSC**	0	0.0000	0.0000	0.0000	0.0000	5.22e-313	0.0000	0.0000	0.2738	0.0100	0.0000	0.0000	0.0890	0.0000
**Breast**	0.0000	0	0.0000	0.0000	0.0000	0.0000	0.0003	0.0000	0.6494	0.0574	0.0000	0.0000	0.0142	0.0198
**Colorectum**	0.0000	0.0000	0	0.0000	0.0000	0.0000	0.0293	0.4311	0.0000	0.0000	0.5044	0.0000	0.1618	0.0000
**Lung**	0.0000	0.0000	0.0000	0	0.0000	0.0000	0.0000	0.0000	0.0000	0.2807	0.0000	0.0000	0.0000	0.0000
**Non-Hodgkins lymphoma**	0.0000	0.0000	0.0000	0.0000	0	0.0000	0.0021	0.1113	0.0233	0.0000	0.0000	0.0000	0.0280	0.0000
**Prostate**	5.26E-313	0.0000	0.0000	0.0000	0.0000	0	0.0000	0.0000	0.0000	0.7163	0.0000	0.0000	0.0000	0.0000
**Tobacco**	0.0000	0.0003	0.0293	0.0000	0.0021	0.0000	0	0.0000	0.0000	0.0000	0.0045	0.0000	0.0000	0.0000
**Alcohol**	0.0000	0.0000	0.4311	0.0000	0.1113	0.0000	0.0000	0	0.0000	0.0000	0.0000	0.0000	0.0357	0.0000
**Last_Month_Cann**	0.2738	0.6494	0.0000	0.0000	0.0233	0.0000	0.0000	0.0000	0	0.1106	0.0000	0.0000	0.0000	0.2106
**Daily_Cannabis**	0.0100	0.0574	0.0000	0.2807	0.0000	0.7163	0.0000	0.0000	0.1106	0	0.0923	0.0000	0.0013	0.0000
**Herb_THC**	0.0000	0.0000	0.5044	0.0000	0.0000	0.0000	0.0045	0.0000	0.0000	0.0923	0	0.0000	0.0000	0.0000
**Resin_THC**	0.0000	0.0000	0.0000	0.0000	0.0000	0.0000	0.0000	0.0000	0.0000	0.0000	0.0000	0	0.0000	0.0330

**Amphetamines**	0.0890	0.0142	0.1618	0.0000	0.0280	0.0000	0.0000	0.0357	0.0000	0.0013	0.0000	0.0000	0	0.0000
**Cocaine**	0.0000	0.0198	0.0000	0.0000	0.0000	0.0000	0.0000	0.0000	0.2106	0.0000	0.0000	0.0330	0.0000	0

Supplementary Table S15: Correlation Matrix for Common Cancers
With Selected Substances – Numbers of Observations Pertaining to Each Cell

Parameter	All Cancers nNMSC	Breast	Colorectum	Lung	Non-Hodgkins lymphoma	Prostate	Tobacco	Alcohol	Last_Month_Cann	Daily_Cannabis	Herb_THC	Resin_THC	Amphetamines	Cocaine
**All Cancers nNMSC**	1523	1503	1523	1503	1520	1523	1522	1522	230	160	1375	1325	553	965
**Breast**	1503	1738	1738	1738	1693	1738	1737	1737	345	249	1540	1484	723	1149
**Colorectum**	1523	1738	1771	1751	1713	1771	1770	1770	345	249	1553	1497	736	1162
**Lung**	1503	1738	1751	1751	1693	1751	1750	1750	345	249	1553	1497	736	1162
**Non-Hodgkins lymphoma**	1520	1693	1713	1693	1713	1713	1712	1712	342	246	1495	1439	678	1104
**Prostate**	1523	1738	1771	1751	1713	1771	1770	1770	345	249	1553	1497	736	1162
**Tobacco**	1522	1737	1770	1750	1712	1770	1770	1770	345	249	1553	1497	736	1162
**Alcohol**	1522	1737	1770	1750	1712	1770	1770	1770	345	249	1553	1497	736	1162
**Last_Month_Cann**	230	345	345	345	342	345	345	345	345	249	309	247	297	324
**Daily_Cannabis**	160	249	249	249	246	249	249	249	249	249	214	192	248	249
**Herb_THC**	1375	1540	1553	1553	1495	1553	1553	1553	309	214	1553	1477	651	1057

**Resin_THC**	1325	1484	1497	149<br>7	1439	1497	1497	1497	247	192	1477	1497	603	1001
**Amphetamine<br>s**	553	723	736	736	678	736	736	736	297	248	651	603	736	736
**Cocaine**	965	1149	1162	116<br>2	1104	1162	1162	1162	324	249	1057	1001	736	1162

Supplementary Table S16.: Ranking of Countries by Mean Tobacco Usage

**Rank**	**Country**	**Mean Tobacco Consumption Annually**
1	Bulgaria	43.78
2	Austria	40.78
3	Latvia	40.37
4	Estonia	37.45
5	Lithuania	34.04
6	France	33.93
7	Spain	33.24
8	Czechia	32.74
9	Sweden	31.93
10	Hungary	31.79
11	Poland	31.43
12	Romania	31.12
13	Germany	30.19
14	Norway	28.42
15	Netherlands	27.96
16	Ireland	27.70
17	Belgium	27.42
18	Finland	27.38
19	United Kingdom	27.32
20	Denmark	25.59
21	Portugal	25.37
22	Italy	25.00
23	Iceland	20.04
24	Luxembourg	-

Supplementary Table S17.: Numbers of Cumulative Exposed and Non-Exposed People in High and Low Tobacco using Countries Respectively and Applicable E-Values

- See also Table 6

Cancer	Mean ASRw High Nations	Mean ASRw Low Nations	Case Numbers in High Countries	Case Numbers in Low Countries	No.'s Non-Cases in High Countries	No.'s Non-Cases in Low Countries	E-Value Estimate	95% Lower Bound of E-Value
Oropharynx	7.674	2.116	282280	123174	3.68E+09	5.82E+09	6.71	6.67
Cervix	14.291	7.159	3851494	3105343	2.69E+10	4.34E+10	3.41	3.40
Stomach	16.058	8.803	4598574	3818352	2.86E+10	4.34E+10	3.05	3.05
Kidney	14.244	8.105	4079247	3515636	2.86E+10	4.34E+10	2.91	2.91
Prostate	114.961	70.372	31303652	30524234	2.72E+10	4.33E+10	2.65	2.65
Pancreas	11.701	7.308	3350836	3170031	2.86E+10	4.34E+10	2.58	2.58
Corpus Uteri	19.558	12.387	5325659	5780667	2.72E+10	4.67E+10	2.53	2.53
Leukaemia	11.408	7.277	2622253	3256530	2.30E+10	4.47E+10	2.51	2.51
All Cancers	522.451	343.838	33237845	17622587	6.33E+09	5.11E+09	2.41	2.41
Ovary	14.008	9.366	3775222	4062447	2.69E+10	4.34E+10	2.36	2.35
Larynx	4.654	3.203	1332667	1348837	2.86E+10	4.21E+10	2.26	2.26
Lung	44.077	30.566	12002011	12872940	2.72E+10	4.21E+10	2.24	2.24
Oesophagus	4.594	3.463	1315587	1501983	2.86E+10	4.34E+10	1.98	1.98
All Cancers nNMSC	386.008	294.903	84876190	116392051	2.19E+10	3.94E+10	1.94	1.94
Oropharynx_Broad	14.240	10.986	519477	309620	3.65E+09	2.82E+09	1.92	1.90
Brain	6.596	6.299	1777663	2732158	2.69E+10	4.34E+10	1.27	1.26
Colorectum	60.428	58.058	16523277	25360673	2.73E+10	4.37E+10	1.25	1.24
Breast	79.880	77.700	22111875	32974891	2.77E+10	4.24E+10	1.20	1.20
Liver	6.064	5.991	1705853	2505349	2.81E+10	4.18E+10	1.12	1.11
Bladder	14.602	14.473	4181692	6277827	2.86E+10	4.34E+10	1.10	1.10
Hepatocellular	1.440	1.316	611	5848	4.25E+07	4.44E+08	1.41	1.09

Penis	0.864	0.881	201803	381933	2.34E+10	4.34E+10	1.16	-
Melanoma	10.883	11.282	2970124	4893660	2.73E+10	4.34E+10	1.23	-
Gallbladder & Biliary	2.191	2.334	489894	986629	2.24E+10	4.23E+10	1.33	-
Leukaemia - Lymphoid	3.872	4.205	223530	204699	5.77E+09	4.87E+09	1.39	-
Hodgkins	2.595	2.869	729933	1289326	2.81E+10	4.49E+10	1.45	-
Anus	0.739	0.846	204788	367078	2.77E+10	4.34E+10	1.55	-
Thyroid	6.715	7.878	1888902	3417217	2.81E+10	4.34E+10	1.62	-
Myeloma	2.896	3.446	812819	1494844	2.81E+10	4.34E+10	1.67	-
Testis	5.433	6.745	1436624	2925877	2.64E+10	4.34E+10	1.79	-
Non-Hodgkins lymphoma	8.885	11.068	2007179	4800778	2.26E+10	4.34E+10	1.80	-
Vulva & Vagina	0.944	1.209	208408	486139	2.21E+10	4.02E+10	1.88	-
Vagina	0.268	0.381	3604	19828	1.34E+09	5.20E+09	2.20	-
Leukaemia - Myeloid	1.455	2.279	85599	110922	5.88E+09	4.87E+09	2.51	-
Vulva	1.070	1.810	14542	94156	1.36E+09	5.20E+09	2.77	-

Supplementary Table S18: Ranking of Nations for

Annual Average Alcohol Consumption

Rank	Country	Annual Mean Alcohol Consumption (Litres)
1	Czechia	14.47
2	Lithuania	14.46
3	Romania	13.54
4	Ireland	13.39
5	Germany	13.36
6	Estonia	13.16
7	Portugal	13.07
8	France	12.92
9	Austria	12.55
10	United Kingdom	11.81
11	Belgium	11.71
12	Finland	11.70
13	Spain	11.62
14	Bulgaria	11.47
15	Denmark	11.22
16	Poland	11.03
17	Hungary	10.64
18	Latvia	10.47
19	Netherlands	10.30
20	Sweden	9.03
21	Italy	8.17
22	Iceland	8.04
23	Norway	7.35
24	Luxembourg	-

Supplementary Table S19.: Numbers of Cumulative Exposed and Non-Exposed People in High and Low Alcohol Consuming Countries Respectively and Applicable E-Values

- See also Supplementary Table S20

Cancer	Mean ASRw High Nations	Mean ASRw Low Nations	Case Numbers in High Countries	Case Numbers in Low Countries	No.'s Non-Cases in High Countries	No.'s Non-Cases in Low Countries	E-Value Estimate	95% Lower Bound of E-Value
Oropharynx_Broad	14.442	6.059	880554	22375	6.10E+09	3.69E+08	4.20	4.14
Cervix	15.026	6.592	4638488	2600841	3.09E+10	3.95E+10	3.99	3.98
Oesophagus	5.455	2.652	1775988	1046404	3.26E+10	3.95E+10	3.53	3.52
Kidney	14.955	7.525	4868863	2969084	3.26E+10	3.95E+10	3.39	3.38
Stomach	16.438	8.561	5351526	3377806	3.26E+10	3.95E+10	3.25	3.24
Prostate	120.720	66.030	37265161	26237487	3.08E+10	3.97E+10	3.06	3.06
All Cancers	534.641	301.414	48319880	7382700	8.99E+09	2.44E+09	2.95	2.94
Pancreas	12.210	6.893	3975104	2719639	3.26E+10	3.95E+10	2.94	2.93
Ovary	14.787	8.714	4564704	3438419	3.09E+10	3.95E+10	2.78	2.78
Leukaemia	11.807	7.010	3344005	2762772	2.83E+10	3.94E+10	2.76	2.75
Lung	45.844	29.535	13573877	11735845	2.96E+10	3.97E+10	2.48	2.48
Corpus Uteri	19.282	12.524	6438382	5073017	3.34E+10	4.05E+10	2.45	2.45
Anus	1.006	0.690	321632	269829	3.20E+10	3.91E+10	2.28	2.26
All Cancers nNMSC	405.170	280.903	97380651	105118389	2.39E+10	3.73E+10	2.24	2.24
Vulva	2.019	1.437	91229	29330	4.52E+09	2.04E+09	2.16	2.12
Oropharynx	3.803	2.875	252846	81979	6.65E+09	2.85E+09	1.98	1.95
Melanoma	12.604	9.652	3933886	3808515	3.12E+10	3.95E+10	1.94	1.93
Breast	87.807	71.223	26228450	28666703	2.98E+10	4.02E+10	1.77	1.77
Vagina	0.415	0.328	18695	6703	4.50E+09	2.04E+09	1.84	1.76
Testis	7.036	5.829	2136444	2299738	3.04E+10	3.95E+10	1.71	1.70

Leukaemia - Lymphoid	4.500	3.774	384464	79188	8.54E+09	2.10E+09	1.67	1.65
Leukaemia - Myeloid	2.179	1.826	187619	39103	8.61E+09	2.14E+09	1.67	1.64
Vulva & Vagina	1.228	1.061	299806	401826	2.44E+10	3.79E+10	1.58	1.57
Non-Hodgkins lymphoma	11.169	9.895	2960744	3904069	2.65E+10	3.95E+10	1.51	1.51
Larynx	4.118	3.762	1288744	1484425	3.13E+10	3.95E+10	1.42	1.41
Colorectum	61.624	56.994	19190460	22731571	3.11E+10	3.99E+10	1.38	1.38
Myeloma	3.397	3.145	1086387	1240974	3.20E+10	3.95E+10	1.37	1.37
Penis	0.902	0.861	249423	336389	2.77E+10	3.91E+10	1.27	1.25
Brain	6.568	6.331	2027388	2497820	3.09E+10	3.95E+10	1.23	1.23
Hodgkins	2.572	2.883	864543	1137703	3.36E+10	3.95E+10	1.49	NA
Hepatocellular	1.207	1.432	1130	5633	9.36E+07	3.93E+08	1.66	NA
Liver	5.317	6.416	1621229	2531429	3.05E+10	3.95E+10	1.71	NA
Thyroid	6.492	7.988	2080789	3151859	3.20E+10	3.95E+10	1.76	NA
Gallbladder & Biliary	1.969	2.466	521955	940325	2.65E+10	3.81E+10	1.81	NA
Bladder	12.554	16.449	4087242	6490250	3.26E+10	3.95E+10	1.95	NA

Supplementary Table S20.: Relative Risks, Attributable Fractions in the Exposed and Population Attributable Risks For High and Low Alcohol Exposure Nations Respectively

Cancer	P_Value	RR_C.I.	AFE_C.I.	PAF_C.I.
Oropharynx_Broad	0.00E+00	2.3835 (2.3521, 2.4154)	0.5805 (0.5748, 0.586)	0.5661 (0.5604, 0.5716)
Cervix	0.00E+00	2.2796 (2.2761, 2.2831)	0.5613 (0.5607, 0.562)	0.3597 (0.359, 0.3603)
Oesophagus	0.00E+00	2.0569 (2.052, 2.0619)	0.5138 (0.5127, 0.515)	0.3233 (0.3223, 0.3244)
Kidney	0.00E+00	1.9874 (1.9845, 1.9903)	0.4968 (0.4961, 0.4976)	0.3086 (0.308, 0.3092)
Stomach	0.00E+00	1.9201 (1.9175, 1.9227)	0.4792 (0.4785, 0.4799)	0.2938 (0.2932, 0.2944)
Prostate	0.00E+00	1.8283 (1.8273, 1.8292)	0.453 (0.4528, 0.4533)	0.2659 (0.2656, 0.2661)
All Cancers	0.00E+00	1.7738 (1.7724, 1.7751)	0.4362 (0.4358, 0.4367)	0.3784 (0.378, 0.3788)
Pancreas	0.00E+00	1.7714 (1.7687, 1.7741)	0.4355 (0.4346, 0.4363)	0.2586 (0.2579, 0.2592)
Ovary	0.00E+00	1.6969 (1.6945, 1.6992)	0.4107 (0.4099, 0.4115)	0.2342 (0.2336, 0.2348)
Leukaemia	0.00E+00	1.6844 (1.6817, 1.6871)	0.4063 (0.4054, 0.4073)	0.2225 (0.2218, 0.2232)
Lung	0.00E+00	1.5522 (1.551, 1.5534)	0.3558 (0.3553, 0.3563)	0.1908 (0.1905, 0.1911)
Corpus Uteri	0.00E+00	1.5396 (1.5378, 1.5414)	0.3505 (0.3497, 0.3512)	0.196 (0.1955, 0.1965)
Anus	0.00E+00	1.459 (1.4516, 1.4665)	0.3146 (0.3111, 0.3181)	0.1711 (0.1688, 0.1734)
All Cancers nNMSC	0.00E+00	1.4424 (1.442, 1.4428)	0.3067 (0.3065, 0.3069)	0.1475 (0.1474, 0.1476)
Vulva	0.00E+00	1.405 (1.3867, 1.4236)	0.2883 (0.2788, 0.2976)	0.2181 (0.2103, 0.2259)
Oropharynx	0.00E+00	1.323 (1.3126, 1.3335)	0.2441 (0.2382, 0.2501)	0.1844 (0.1795, 0.1892)
Melanoma	0.00E+00	1.3058 (1.304, 1.3076)	0.2342 (0.2331, 0.2353)	0.119 (0.1184, 0.1196)
Breast	0.00E+00	1.2329 (1.2322, 1.2335)	0.1889 (0.1884, 0.1893)	0.0902 (0.09, 0.0905)
Vagina	2.19E-61	1.2639 (1.2291, 1.2997)	0.2088 (0.1864, 0.2306)	0.1537 (0.1361, 0.1709)
Testis	0.00E+00	1.2072 (1.205, 1.2095)	0.1717 (0.1701, 0.1732)	0.0827 (0.0819, 0.0835)
Leukaemia - Lymphoid	0.00E+00	1.1926 (1.1835, 1.2017)	0.1615 (0.155, 0.1679)	0.1339 (0.1284, 0.1394)
Leukaemia - Myeloid	3.85E-222	1.1931 (1.1801, 1.2061)	0.1618 (0.1526, 0.1709)	0.1339 (0.1261, 0.1417)
Vulva & Vagina	0.00E+00	1.1573 (1.1519, 1.1628)	0.1359 (0.1318, 0.14)	0.0581 (0.0562, 0.06)
Non-Hodgkins lymphoma	0.00E+00	1.1288 (1.1271, 1.1305)	0.1141 (0.1128, 0.1154)	0.0492 (0.0486, 0.0498)
Larynx	0.00E+00	1.0946 (1.092, 1.0971)	0.0864 (0.0842, 0.0885)	0.0401 (0.0391, 0.0412)

Colorectum	0.00E+00	1.0812 (1.0806, 1.0819)	0.0751 (0.0746, 0.0757)	0.0344 (0.0341, 0.0347)
Myeloma	0.00E+00	1.08 (1.0772, 1.0828)	0.0741 (0.0717, 0.0765)	0.0346 (0.0334, 0.0357)
Penis	1.82E-70	1.0479 (1.0425, 1.0533)	0.0457 (0.0408, 0.0506)	0.0195 (0.0173, 0.0216)
Brain	0.00E+00	1.0375 (1.0355, 1.0394)	0.0361 (0.0343, 0.0379)	0.0162 (0.0154, 0.017)
Hodgkins	0.00E+00	0.8922 (0.8897, 0.8947)	-0.1209 (-0.124, -0.1178)	-0.0522 (-0.0535, -0.0509)
Hepatocellular	7.63E-08	0.8429 (0.7907, 0.8985)	-0.1864 (-0.2646, -0.113)	-0.0311 (-0.0422, -0.0202)
Liver	0.00E+00	0.8287 (0.8271, 0.8304)	-0.2067 (-0.209, -0.2043)	-0.0807 (-0.0815, -0.0799)
Thyroid	0.00E+00	0.8127 (0.8113, 0.8142)	-0.2304 (-0.2325, -0.2282)	-0.0916 (-0.0924, -0.0909)
Gallbladder & Biliary	0.00E+00	0.7984 (0.7957, 0.8011)	-0.2525 (-0.2568, -0.2483)	-0.0901 (-0.0914, -0.0888)
Bladder	0.00E+00	0.7632 (0.7623, 0.7642)	-0.3102 (-0.3119, -0.3086)	-0.1199 (-0.1204, -0.1193)

Supplementary Table S21: Ranking of Nations for

Cannabis Consumption

Rank	Country	Mean Last Month Cannabis Use	Mean Daily Cannabis Use (%)	Mean THC Content Cannabis Herb	Mean THC Content Cannabis Resin
1	Belgium	0.0460	0.0132	0.1263	0.1513
2	Norway	0.0089	0.0045	0.1200	0.2500
3	Hungary	0.0152	0.0017	0.1139	0.2207
4	Germany	0.0397	0.0027	0.1060	0.0396
5	Estonia	0.0265	-	0.0975	0.0482
6	Romania	0.0032	-	0.0777	0.0408
7	France	0.0470	0.0150	0.0746	0.0881
8	Austria	-	-	0.0697	0.0668
9	Poland	0.0521	0.0040	0.0648	-
10	Sweden	0.0099	-	0.0604	0.1228
11	Netherlands	0.0433	0.0077	0.0590	0.2695
12	Ireland	0.0417	-	0.0570	0.0237
13	Italy	-	-	0.0538	0.0246
14	Czechia	0.0451	-	0.0448	0.0541
15	Finland	0.0174	0.0032	0.0402	-
16	Bulgaria	-	-	0.0336	0.0090
17	Portugal	-	-	0.0271	0.0368
18	Spain	0.0773	0.0285	0.0038	0.0080
19	Denmark	0.0270	0.0047	-	0.2663
20	Iceland	-	-	-	-
21	Latvia	0.0175	0.0019	-	-
22	Lithuania	-	-	-	-
23	Luxembourg	-	-	-	-
24	United Kingdom	-	-	-	-

Supplementary Table 22.: Numbers of Cumulative Exposed and Non-Exposed People in High and Low Alcohol Consuming Countries Respectively and Applicable E-Values
See also Table 7

**Cancer**	**Mean ASRw High Nations**	**Mean ASRw Low Nations**	**Case Numbers in High Countries**	**Case Numbers in Low Countries**	**No.'s Non-Cases in High Countries**	**No.'s Non-Cases in Low Countries**	**E-Value Estimate**	**95% Lower Bound of E-Value**
Kaposi	0.286	0.138	2728	2780	9.54E+08	2.02E+09	3.58	3.36
Liver	6.529	3.704	4121842	252491	6.31E+10	6.82E+09	2.92	2.91
Thyroid	8.022	4.741	5189225	323166	6.47E+10	6.82E+09	2.77	2.76
Stomach	13.198	7.834	8604632	534040	6.52E+10	6.82E+09	2.76	2.75
Oropharynx_Broad	14.490	8.942	746046	117813	5.15E+09	1.32E+09	2.62	2.60
Larynx	4.142	2.604	2700747	144700	6.52E+10	5.56E+09	2.56	2.54
Breast	83.392	55.972	53495071	3342222	6.41E+10	5.97E+09	2.34	2.34
All Cancers	490.514	348.942	44112136	8703057	8.95E+09	2.49E+09	2.16	2.16
Hodgkins	2.883	2.220	1910067	151357	6.62E+10	6.82E+09	1.92	1.91
Bladder	15.023	11.649	9794262	794095	6.52E+10	6.82E+09	1.90	1.89
Kidney	11.542	8.968	7524752	611333	6.52E+10	6.82E+09	1.89	1.89
Pancreas	9.816	7.634	6399767	520382	6.52E+10	6.82E+09	1.89	1.88
Prostate	95.394	74.878	60850273	5104252	6.37E+10	6.81E+09	1.86	1.86
Lung	38.430	30.249	24513914	1680712	6.38E+10	5.55E+09	1.86	1.85
Leukaemia	9.514	7.628	5799676	516775	6.10E+10	6.77E+09	1.80	1.79
Colorectum	60.859	49.941	38968716	3493087	6.40E+10	6.99E+09	1.73	1.73
All Cancers nNMSC	348.385	287.415	193251354	17203216	5.53E+10	5.97E+09	1.72	1.72
Gallbladder & Biliary	2.311	2.084	1364315	116646	5.90E+10	5.60E+09	1.46	1.44
Myeloma	3.295	2.976	2129158	202843	6.46E+10	6.82E+09	1.45	1.44
Leukaemia - Myeloid	2.043	1.872	168148	47209	8.23E+09	2.52E+09	1.41	1.38

Leukaemia - Lymphoid	4.210	3.896	343629	96614	8.16E+09	2.48E+09	1.38	1.35
Corpus Uteri	15.895	14.915	10261549	1392771	6.45E+10	9.34E+09	1.33	1.32
Cervix	10.756	10.186	6831324	694366	6.35E+10	6.82E+09	1.30	1.29
Testis	6.304	6.069	3971392	413711	6.30E+10	6.82E+09	1.24	1.23
Ovary	11.694	11.414	7426766	778055	6.35E+10	6.82E+09	1.18	1.17
Anus	0.810	0.789	520516	53702	6.43E+10	6.80E+09	1.19	1.15
Non-Hodgkins lymphoma	10.275	10.505	6077238	716083	5.91E+10	6.82E+09	1.17	NA
Melanoma	11.013	11.506	7031931	784353	6.38E+10	6.82E+09	1.26	NA
Hepatocellular	1.316	1.440	5848	611	4.44E+08	4.25E+07	1.41	NA
Oesophagus	3.962	4.420	2582943	301328	6.52E+10	6.82E+09	1.48	NA
Brain	6.202	7.873	3938697	536653	6.35E+10	6.82E+09	1.85	NA
Penis	0.831	1.084	498381	73294	6.00E+10	6.76E+09	1.94	NA
Vulva	1.568	2.183	68164	48326	4.35E+09	2.21E+09	2.13	NA
Vagina	0.338	0.477	14683	10494	4.35E+09	2.20E+09	2.17	NA
Vulva & Vagina	1.032	1.481	582143	86830	5.64E+10	5.86E+09	2.23	NA
Oropharynx	2.682	4.262	184664	111456	6.89E+09	2.61E+09	2.56	NA

Supplementary Table S23.: Complete Output from Additive Mixed Effects Regression

Cancer	Term	β-estimate	Std. Error	t-statistic	P-Value	Sigma	logLik	eValPtL	eValLoL
All Cancers nNMSC	LM.Cannabis	15.369	2.052	7.491	1.52E-11	0.048	220.981	3.60E+126	3.86E+93
All Cancers nNMSC	THC.Herb	8.959	0.901	9.942	3.50E-17	0.048	220.981	7.94E+73	2.62E+59
Liver	LM.Cannabis	20.615	2.734	7.539	3.18E-12	0.158	68.146	6.20E+51	2.71E+38
Myeloma	LM.Cannabis	9.410	1.646	5.718	4.40E-08	0.127	154.791	3.47E+29	3.45E+19
Leukaemia - Lymphoid	LM.Cannabis	14.507	3.986	3.640	8.98E-04	0.141	37.529	7.07E+40	1.14E+19
Hodgkins	LM.Cannabis	10.659	1.719	6.199	3.21E-09	0.190	97.378	2.68E+22	2.79E+15
Pancreas	THC.Herb	4.735	0.692	6.841	1.19E-10	0.093	170.289	3.17E+20	5.31E+14
Kidney	LM.Cannabis	7.878	1.985	3.968	1.05E-04	0.129	119.188	3.11E+24	3.69E+12
Kidney	THC.Herb	5.497	0.858	6.407	1.26E-09	0.129	119.188	1.51E+17	1.06E+12
Oesophagus	LM.Cannabis	8.592	2.271	3.784	2.10E-04	0.153	82.451	3.11E+22	1.05E+11
Leukaemia - Myeloid	THC.Herb	11.054	3.433	3.220	2.82E-03	0.163	17.291	1.45E+27	6.95E+10
Melanoma	LM.Cannabis	8.008	2.183	3.668	3.22E-04	0.153	88.398	1.12E+21	9.64E+09
Cervix	THC.Herb	4.984	0.898	5.550	1.03E-07	0.143	103.542	1.26E+14	1.73E+09
Pancreas	LM.Cannabis	5.298	1.704	3.109	2.18E-03	0.093	170.289	8.00E+22	4.84E+08
Colorectum	LM.Cannabis	6.280	1.949	3.222	1.51E-03	0.126	124.025	1.16E+20	1.16E+08
Prostate	THC.Herb	4.050	0.889	4.557	9.66E-06	0.128	109.867	6.66E+12	2.81E+07
Thyroid	LM.Cannabis	9.862	3.297	2.991	3.17E-03	0.199	28.161	7.90E+19	1.21E+07
All Cancers	THC.Herb	2.680	0.846	3.170	2.53E-03	0.065	77.214	4.25E+16	3.58E+06
Stomach	THC.Herb	3.233	0.792	4.081	6.73E-05	0.107	138.190	1.71E+12	3.26E+06
Larynx	THC.Herb	4.407	1.100	4.008	8.95E-05	0.175	55.624	1.71E+10	2.43E+05
Ovary	THC.Herb	2.308	0.641	3.603	4.09E-04	0.101	184.626	2.00E+09	2.60E+04
Breast	THC.Herb	1.921	0.538	3.568	4.62E-04	0.085	225.891	1.53E+09	2.06E+04
Colorectum	THC.Herb	2.908	0.838	3.471	6.49E-04	0.126	124.025	2.83E+09	1.97E+04
Melanoma	THC.Herb	3.405	0.963	3.535	5.18E-04	0.153	88.398	1.33E+09	1.75E+04
Testis	THC.Herb	2.995	0.979	3.060	2.55E-03	0.137	124.374	8.18E+08	2.56E+03
Myeloma	THC.Herb	2.522	0.824	3.062	2.54E-03	0.127	154.791	1.37E+08	1.35E+03

Non-Hodgkins lymphoma	THC.Herb	1.829	0.637	2.873	4.57E-03	0.089	190.181	2.78E+08	7.93E+02
Prostate	LM.Cannabis	4.499	2.104	2.139	3.38E-02	0.128	109.867	1.63E+14	3.04E+01
Prostate	Income	0.453	0.048	9.382	3.14E-17	0.128	109.867	5.00E+01	2.52E+01
Leukaemia - Myeloid	Income	0.823	0.202	4.070	2.65E-04	0.163	17.291	2.00E+02	2.14E+01
Liver	THC.Herb	3.167	1.412	2.243	2.63E-02	0.158	68.146	1.63E+08	2.00E+01
Bladder	THC.Herb	2.105	0.902	2.333	2.07E-02	0.135	107.200	3.00E+06	1.94E+01
Oesophagus	THC.Herb	2.206	0.994	2.220	2.77E-02	0.153	82.451	9.99E+05	9.00E+00
Thyroid	Income	0.381	0.079	4.851	2.64E-06	0.199	28.161	1.09E+01	5.11E+00
Breast	Income	0.136	0.028	4.834	2.89E-06	0.085	225.891	7.99E+00	4.17E+00
Gallbladder & Biliary	Alcohol	0.191	0.053	3.580	4.57E-04	0.125	111.944	7.48E+00	3.16E+00
Myeloma	Income	0.162	0.040	4.072	6.98E-05	0.127	154.791	5.85E+00	3.06E+00
Oropharynx_Broad	Income	0.126	0.052	2.427	2.12E-02	0.040	59.858	3.37E+01	2.86E+00
Lung	Alcohol	0.039	0.009	4.221	3.88E-05	0.046	281.596	3.78E+00	2.41E+00
Leukaemia - Myeloid	Tobacco	0.120	0.025	4.724	3.90E-05	0.163	17.291	3.33E+00	2.33E+00
Colorectum	Alcohol	0.093	0.021	4.507	1.18E-05	0.126	124.025	3.34E+00	2.29E+00
Oesophagus	Income	0.166	0.052	3.167	1.81E-03	0.153	82.451	4.81E+00	2.28E+00
Hodgkins	THC.Resin	0.595	0.270	2.209	2.83E-02	0.190	97.378	3.39E+01	2.12E+00
All Cancers nNMSC	Tobacco	0.021	0.004	5.696	9.55E-08	0.048	220.981	2.32E+00	1.91E+00
Anus	Income	0.136	0.052	2.608	9.88E-03	0.144	111.232	4.14E+00	1.78E+00
Hodgkins	Income	0.117	0.047	2.506	1.30E-02	0.190	97.378	2.90E+00	1.52E+00
Myeloma	Tobacco	0.022	0.005	4.509	1.17E-05	0.127	154.791	1.62E+00	1.41E+00
Prostate	Tobacco	0.025	0.006	3.773	2.20E-04	0.128	109.867	1.67E+00	1.40E+00
Larynx	Tobacco	0.029	0.007	3.885	1.44E-04	0.175	55.624	1.60E+00	1.37E+00
Hodgkins	Tobacco	0.020	0.005	4.038	7.70E-05	0.190	97.378	1.43E+00	1.28E+00
Oesophagus	Tobacco	0.021	0.007	3.069	2.48E-03	0.153	82.451	1.52E+00	1.27E+00
Breast	Tobacco	0.011	0.004	3.023	2.87E-03	0.085	225.891	1.50E+00	1.25E+00
Non-Hodgkins lymphoma	Tobacco	0.012	0.005	2.417	1.67E-02	0.089	190.181	1.50E+00	1.18E+00
Ovary	Tobacco	0.009	0.004	2.114	3.59E-02	0.101	184.626	1.39E+00	1.09E+00
Oropharynx	Alcohol	0.011	0.125	0.085	9.32E-01	0.390	-16.719	1.19E+00	1.00E+00
Stomach	Tobacco	0.004	0.006	0.666	5.06E-01	0.107	138.190	1.22E+00	1.00E+00

Colorectum	Tobacco	0.006	0.006	1.016	3.11E-01	0.126	124.025	1.26E+00	1.00E+00
Corpus Uteri	Alcohol	0.021	0.049	0.429	6.68E-01	0.354	-103.266	1.30E+00	1.00E+00
Kidney	Tobacco	0.009	0.006	1.532	1.27E-01	0.129	119.188	1.34E+00	1.00E+00
Testis	Income	0.010	0.048	0.215	8.30E-01	0.137	124.374	1.35E+00	1.00E+00
Liver	Tobacco	0.012	0.008	1.434	1.54E-01	0.158	68.146	1.35E+00	1.00E+00
Liver	Income	0.020	0.064	0.313	7.54E-01	0.158	68.146	1.49E+00	1.00E+00
Oropharynx	Tobacco	0.052	0.051	1.019	3.15E-01	0.390	-16.719	1.51E+00	1.00E+00
Prostate	Alcohol	0.018	0.022	0.836	4.04E-01	0.128	109.867	1.54E+00	1.00E+00
Kidney	Income	0.019	0.046	0.419	6.76E-01	0.129	119.188	1.55E+00	1.00E+00
Lung	Income	0.008	0.024	0.331	7.41E-01	0.046	281.596	1.61E+00	1.00E+00
Brain	Income	0.024	0.050	0.478	6.34E-01	0.137	99.167	1.62E+00	1.00E+00
Colorectum	Income	0.025	0.045	0.554	5.80E-01	0.126	124.025	1.68E+00	1.00E+00
Stomach	Alcohol	0.035	0.020	1.802	7.33E-02	0.107	138.190	2.04E+00	1.00E+00
Melanoma	Income	0.069	0.051	1.366	1.74E-01	0.153	88.398	2.39E+00	1.00E+00
Non-Hodgkins lymphoma	Income	0.041	0.036	1.139	2.56E-01	0.089	190.181	2.40E+00	1.00E+00
Leukaemia - Myeloid	Alcohol	0.081	0.136	0.591	5.58E-01	0.163	17.291	2.51E+00	1.00E+00
Gallbladder & Biliary	Income	0.116	0.075	1.544	1.25E-01	0.125	111.944	4.07E+00	1.00E+00
Liver	THC.Resin	0.173	0.611	0.284	7.77E-01	0.158	68.146	4.86E+00	1.00E+00
All Cancers	Income	0.075	0.054	1.394	1.69E-01	0.065	77.214	5.21E+00	1.00E+00
Oropharynx	Income	0.495	0.413	1.198	2.38E-01	0.390	-16.719	5.80E+00	1.00E+00
Myeloma	THC.Resin	0.213	0.306	0.698	4.86E-01	0.127	154.791	8.68E+00	1.00E+00
Thyroid	THC.Resin	0.392	0.681	0.575	5.66E-01	0.199	28.161	1.15E+01	1.00E+00
Anus	THC.Resin	0.554	0.439	1.261	2.09E-01	0.144	111.232	6.53E+01	1.00E+00
Corpus Uteri	THC.Herb	1.497	2.039	0.734	4.64E-01	0.354	-103.266	9.37E+01	1.00E+00
Hodgkins	THC.Herb	1.316	0.849	1.549	1.23E-01	0.190	97.378	1.08E+03	1.00E+00
Thyroid	THC.Herb	1.393	1.538	0.906	3.66E-01	0.199	28.161	1.17E+03	1.00E+00
All Cancers	LM.Cannabis	0.538	1.992	0.270	7.88E-01	0.065	77.214	3.81E+03	1.00E+00
Leukaemia - Myeloid	THC.Resin	1.512	1.291	1.172	2.50E-01	0.163	17.291	9.47E+03	1.00E+00
Brain	THC.Herb	1.279	0.928	1.378	1.70E-01	0.137	99.167	9.81E+03	1.00E+00
Larynx	LM.Cannabis	2.344	2.475	0.947	3.45E-01	0.175	55.624	3.83E+05	1.00E+00

Anus	LM.Cannabis	2.090	2.197	0.951	3.43E-01	0.144	111.232	1.06E+06	1.00E+00
Testis	LM.Cannabis	2.621	1.998	1.311	1.91E-01	0.137	124.374	6.87E+07	1.00E+00
Oropharynx	THC.Herb	8.718	5.014	1.739	9.00E-02	0.390	-16.719	1.35E+09	1.00E+00
Non-Hodgkins lymphoma	LM.Cannabis	2.262	1.547	1.462	1.46E-01	0.089	190.181	2.35E+10	1.00E+00
Breast	LM.Cannabis	2.253	1.215	1.854	6.55E-02	0.085	225.891	5.20E+10	1.00E+00
Bladder	Tobacco	-0.001	0.006	-0.230	8.19E-01	0.135	107.200	1.11E+00	-
Leukaemia - Lymphoid	Alcohol	-0.002	0.038	-0.048	9.62E-01	0.141	37.529	1.12E+00	-
Pancreas	Tobacco	-0.001	0.005	-0.239	8.11E-01	0.093	170.289	1.13E+00	-
Corpus Uteri	Tobacco	-0.006	0.013	-0.449	6.54E-01	0.354	-103.266	1.14E+00	-
All Cancers	Tobacco	-0.001	0.007	-0.196	8.45E-01	0.065	77.214	1.16E+00	-
Ovary	Alcohol	-0.003	0.016	-0.176	8.61E-01	0.101	184.626	1.19E+00	-
Thyroid	Tobacco	-0.007	0.010	-0.680	4.97E-01	0.199	28.161	1.21E+00	-
Brain	Tobacco	-0.006	0.007	-0.935	3.51E-01	0.137	99.167	1.25E+00	-
Larynx	Alcohol	-0.009	0.027	-0.338	7.36E-01	0.175	55.624	1.27E+00	-
Anus	Tobacco	-0.009	0.007	-1.312	1.91E-01	0.144	111.232	1.30E+00	-
Brain	Alcohol	-0.009	0.023	-0.375	7.08E-01	0.137	99.167	1.31E+00	-
Hodgkins	Alcohol	-0.015	0.021	-0.695	4.88E-01	0.190	97.378	1.35E+00	-
Melanoma	Tobacco	-0.012	0.007	-1.881	6.16E-02	0.153	88.398	1.36E+00	-
Testis	Tobacco	-0.018	0.006	-2.971	3.39E-03	0.137	124.374	1.50E+00	-
Thyroid	Alcohol	-0.032	0.037	-0.890	3.75E-01	0.199	28.161	1.59E+00	-
Gallbladder & Biliary	Tobacco	-0.024	0.010	-2.529	1.24E-02	0.125	111.944	1.67E+00	-
Cervix	Tobacco	-0.029	0.006	-4.774	3.77E-06	0.143	103.542	1.70E+00	-
Lung	Tobacco	-0.010	0.003	-3.092	2.31E-03	0.046	281.596	1.73E+00	-
Myeloma	Alcohol	-0.034	0.019	-1.735	8.45E-02	0.127	154.791	1.86E+00	-
Bladder	Alcohol	-0.037	0.022	-1.657	9.93E-02	0.135	107.200	1.89E+00	-
Corpus Uteri	Income	-0.101	0.106	-0.951	3.43E-01	0.354	-103.266	1.91E+00	-
Bladder	Income	-0.041	0.048	-0.844	4.00E-01	0.135	107.200	1.96E+00	-
Leukaemia - Lymphoid	Tobacco	-0.050	0.012	-3.994	3.30E-04	0.141	37.529	2.10E+00	-
Larynx	Income	-0.064	0.057	-1.119	2.65E-01	0.175	55.624	2.14E+00	-
All Cancers	Alcohol	-0.026	0.027	-0.965	3.39E-01	0.065	77.214	2.23E+00	-

Testis	Alcohol	-0.056	0.023	-2.405	1.72E-02	0.137	124.374	2.25E+00	-
Cervix	Alcohol	-0.058	0.022	-2.663	8.46E-03	0.143	103.542	2.26E+00	-
Kidney	Alcohol	-0.053	0.021	-2.518	1.27E-02	0.129	119.188	2.28E+00	-
Oropharynx_Broad	Tobacco	-0.018	0.022	-0.815	4.22E-01	0.040	59.858	2.37E+00	-
Anus	Alcohol	-0.068	0.025	-2.740	6.75E-03	0.144	111.232	2.45E+00	-
Pancreas	Income	-0.046	0.039	-1.163	2.46E-01	0.093	170.289	2.51E+00	-
Oesophagus	Alcohol	-0.085	0.024	-3.488	6.11E-04	0.153	82.451	2.71E+00	-
Breast	Alcohol	-0.058	0.013	-4.411	1.79E-05	0.085	225.891	3.12E+00	-
Melanoma	Alcohol	-0.118	0.024	-4.998	1.37E-06	0.153	88.398	3.45E+00	-
Oropharynx	THC.Resin	-0.310	2.419	-0.128	8.99E-01	0.390	-16.719	3.54E+00	-
Oesophagus	THC.Resin	-0.124	0.413	-0.301	7.64E-01	0.153	82.451	3.60E+00	-
Liver	Alcohol	-0.146	0.030	-4.825	3.21E-06	0.158	68.146	4.05E+00	-
Pancreas	Alcohol	-0.102	0.017	-5.965	1.27E-08	0.093	170.289	4.91E+00	-
Cervix	LM.Cannabis	-0.179	2.026	-0.088	9.30E-01	0.143	103.542	5.69E+00	-
Oropharynx_Broad	Alcohol	-0.051	0.084	-0.602	5.52E-01	0.040	59.858	5.71E+00	-
Non-Hodgkins lymphoma	Alcohol	-0.124	0.016	-7.829	4.33E-13	0.089	190.181	6.57E+00	-
Ovary	Income	-0.148	0.034	-4.403	1.84E-05	0.101	184.626	6.99E+00	-
Anus	THC.Herb	-0.247	1.059	-0.233	8.16E-01	0.144	111.232	8.94E+00	-
Stomach	Income	-0.221	0.045	-4.951	1.69E-06	0.107	138.190	1.25E+01	-
Leukaemia - Lymphoid	Income	-0.303	0.103	-2.928	6.05E-03	0.141	37.529	1.35E+01	-
Non-Hodgkins lymphoma	THC.Resin	-0.204	0.296	-0.691	4.91E-01	0.089	190.181	1.57E+01	-
All Cancers nNMSC	Income	-0.119	0.026	-4.649	8.95E-06	0.048	220.981	1.84E+01	-
Cervix	Income	-0.363	0.047	-7.741	7.26E-13	0.143	103.542	1.98E+01	-
All Cancers nNMSC	Alcohol	-0.146	0.022	-6.720	7.33E-10	0.048	220.981	3.09E+01	-
Melanoma	THC.Resin	-0.498	0.391	-1.276	2.04E-01	0.153	88.398	3.86E+01	-
Breast	THC.Resin	-0.302	0.217	-1.389	1.67E-01	0.085	225.891	4.92E+01	-
Corpus Uteri	THC.Resin	-1.424	0.766	-1.859	6.47E-02	0.354	-103.266	7.77E+01	-
Lung	THC.Herb	-0.219	0.384	-0.568	5.70E-01	0.046	281.596	1.57E+02	-
Bladder	LM.Cannabis	-0.670	2.093	-0.320	7.49E-01	0.135	107.200	1.85E+02	-
Larynx	THC.Resin	-1.132	0.441	-2.565	1.11E-02	0.175	55.624	7.11E+02	-

Brain	THC.Resin	-0.924	0.403	-2.295	2.29E-02	0.137	99.167	9.30E+02	-
Bladder	THC.Resin	-0.930	0.386	-2.406	1.72E-02	0.135	107.200	1.07E+03	-
Gallbladder & Biliary	THC.Resin	-1.037	0.464	-2.238	2.66E-02	0.125	111.944	3.79E+03	-
Colorectum	THC.Resin	-1.149	0.360	-3.193	1.66E-03	0.126	124.025	8.24E+03	-
Kidney	THC.Resin	-1.408	0.366	-3.850	1.64E-04	0.129	119.188	4.21E+04	-
Prostate	THC.Resin	-1.436	0.396	-3.628	3.73E-04	0.128	109.867	5.53E+04	-
Testis	THC.Resin	-1.711	0.395	-4.335	2.45E-05	0.137	124.374	1.66E+05	-
Brain	LM.Cannabis	-1.710	2.168	-0.789	4.31E-01	0.137	99.167	1.72E+05	-
Pancreas	THC.Resin	-1.183	0.332	-3.563	4.69E-04	0.093	170.289	2.22E+05	-
Leukaemia - Lymphoid	THC.Resin	-1.944	0.613	-3.169	3.23E-03	0.141	37.529	5.44E+05	-
Ovary	THC.Resin	-1.466	0.259	-5.656	6.12E-08	0.101	184.626	1.05E+06	-
Lung	THC.Resin	-0.709	0.208	-3.417	7.86E-04	0.046	281.596	2.88E+06	-
Leukaemia - Lymphoid	THC.Herb	-2.261	1.694	-1.335	1.91E-01	0.141	37.529	4.18E+06	-
Ovary	LM.Cannabis	-1.851	1.448	-1.279	2.03E-01	0.101	184.626	3.32E+07	-
Cervix	THC.Resin	-2.636	0.362	-7.285	1.02E-11	0.143	103.542	3.97E+07	-
Stomach	THC.Resin	-1.980	0.376	-5.266	3.95E-07	0.107	138.190	4.06E+07	-
Corpus Uteri	LM.Cannabis	-6.698	4.488	-1.492	1.37E-01	0.354	-103.266	6.14E+07	-
All Cancers	THC.Resin	-1.302	0.390	-3.334	1.57E-03	0.065	77.214	1.70E+08	-
All Cancers nNMSC	THC.Resin	-1.088	0.208	-5.230	7.70E-07	0.048	220.981	1.75E+09	-
Oropharynx_Broad	THC.Resin	-1.825	0.634	-2.879	7.17E-03	0.040	59.858	1.59E+18	-
Gallbladder & Biliary	LM.Cannabis	-5.709	4.876	-1.171	2.43E-01	0.125	111.944	2.18E+18	-
Oropharynx_Broad	THC.Herb	-2.015	3.877	-0.520	6.07E-01	0.040	59.858	1.15E+20	-
Gallbladder & Biliary	THC.Herb	-7.062	2.911	-2.426	1.64E-02	0.125	111.944	4.07E+22	-
Lung	LM.Cannabis	-2.684	1.013	-2.651	8.75E-03	0.046	281.596	4.00E+23	-
Oropharynx	LM.Cannabis	-28.081	17.876	-1.571	1.24E-01	0.390	-16.719	5.54E+28	-
Stomach	LM.Cannabis	-10.925	1.939	-5.634	6.67E-08	0.107	138.190	4.20E+40	-
Leukaemia - Myeloid	LM.Cannabis	-37.633	26.246	-1.434	1.61E-01	0.163	17.291	5.57E+91	-
Oropharynx_Broad	LM.Cannabis	-17.440	8.264	-2.110	4.30E-02	0.040	59.858	Inf	-

Supplementary Table S24.: Positive and Significant Terms from Additive Mixed Effects Regression

Cancer	Term	ß-Estimate	Std. Error	t-Statistic	AIC	P_Value	Adj.P.FDR	Adj.P.Holm	E-Value Estimate	E-Value 95% Lower Bound
All Cancers nNMSC	LM.Cannabis	15.369	2.052	7.491	-423.961	1.52E-11	2.06E-10	7.76E-10	3.60E+126	3.86E+93
All Cancers nNMSC	THC.Herb	8.959	0.901	9.942	-423.961	3.50E-17	9.46E-16	1.86E-15	7.94E+73	2.62E+59
Liver	LM.Cannabis	20.615	2.734	7.539	-118.291	3.18E-12	5.73E-11	1.65E-10	6.20E+51	2.71E+38
Myeloma	LM.Cannabis	9.410	1.646	5.718	-291.582	4.40E-08	2.97E-07	2.07E-06	3.47E+29	3.45E+19
Leukaemia - Lymphoid	LM.Cannabis	14.507	3.986	3.640	-57.057	8.98E-04	1.47E-03	1.97E-02	7.07E+40	1.14E+19
Hodgkins	LM.Cannabis	10.659	1.719	6.199	-176.757	3.21E-09	2.48E-08	1.54E-07	2.68E+22	2.79E+15
Pancreas	THC.Herb	4.735	0.692	6.841	-322.579	1.19E-10	1.28E-09	5.95E-09	3.17E+20	5.31E+14
Kidney	LM.Cannabis	7.878	1.985	3.968	-220.375	1.05E-04	2.57E-04	3.45E-03	3.11E+24	3.69E+12
Kidney	THC.Herb	5.497	0.858	6.407	-220.375	1.26E-09	1.13E-08	6.18E-08	1.51E+17	1.06E+12
Oesophagus	LM.Cannabis	8.592	2.271	3.784	-146.903	2.10E-04	4.73E-04	6.51E-03	3.11E+22	1.05E+11
Leukaemia - Myeloid	THC.Herb	11.054	3.433	3.220	-16.581	2.82E-03	3.69E-03	4.47E-02	1.45E+27	6.95E+10
Melanoma	LM.Cannabis	8.008	2.183	3.668	-158.797	3.22E-04	6.44E-04	9.02E-03	1.12E+21	9.64E+09
Cervix	THC.Herb	4.984	0.898	5.550	-189.083	1.03E-07	5.55E-07	4.62E-06	1.26E+14	1.73E+09
Pancreas	LM.Cannabis	5.298	1.704	3.109	-322.579	2.18E-03	3.27E-03	4.14E-02	8.00E+22	4.84E+08
Colorectum	LM.Cannabis	6.280	1.949	3.222	-230.050	1.51E-03	2.40E-03	3.17E-02	1.16E+20	1.16E+08
Prostate	THC.Herb	4.050	0.889	4.557	-201.735	9.66E-06	4.01E-05	4.06E-04	6.66E+12	2.81E+07
Thyroid	LM.Cannabis	9.862	3.297	2.991	-38.321	3.17E-03	3.98E-03	4.47E-02	7.90E+19	1.21E+07
All Cancers	THC.Herb	2.680	0.846	3.170	-136.427	2.53E-03	3.45E-03	4.47E-02	4.25E+16	3.58E+06

Stomach	THC.Herb	3.233	0.792	4.081	-258.380	6.73E-05	1.98E-04	2.49E-03	1.71E+12	3.26E+06
Larynx	THC.Herb	4.407	1.100	4.008	-93.247	8.95E-05	2.30E-04	3.04E-03	1.71E+10	2.43E+05
Ovary	THC.Herb	2.308	0.641	3.603	-351.253	4.09E-04	7.89E-04	1.10E-02	2.00E+09	2.60E+04
Breast	THC.Herb	1.921	0.538	3.568	-433.783	4.62E-04	8.32E-04	1.19E-02	1.53E+09	2.06E+04
Colorectum	THC.Herb	2.908	0.838	3.471	-230.050	6.49E-04	1.09E-03	1.49E-02	2.83E+09	1.97E+04
Melanoma	THC.Herb	3.405	0.963	3.535	-158.797	5.18E-04	9.02E-04	1.24E-02	1.33E+09	1.75E+04
Testis	THC.Herb	2.995	0.979	3.060	-230.748	2.55E-03	3.45E-03	4.47E-02	8.18E+08	2.56E+03
Myeloma	THC.Herb	2.522	0.824	3.062	-291.582	2.54E-03	3.45E-03	4.47E-02	1.37E+08	1.35E+03
Non-Hodgkins lymphoma	THC.Herb	1.829	0.637	2.873	-362.362	4.57E-03	5.61E-03	5.03E-02	2.78E+08	7.93E+02
Prostate	LM.Cannabis	4.499	2.104	2.139	-201.735	3.38E-02	3.45E-02	1.45E-01	1.63E+14	3.04E+01
Prostate	Income	0.453	0.048	9.382	-201.735	3.14E-17	9.46E-16	1.70E-15	5.00E+01	2.52E+01
Leukaemia - Myeloid	Income	0.823	0.202	4.070	-16.581	2.65E-04	5.50E-04	7.68E-03	2.00E+02	2.14E+01
Liver	THC.Herb	3.167	1.412	2.243	-118.291	2.63E-02	2.84E-02	1.45E-01	1.63E+08	2.00E+01
Bladder	THC.Herb	2.105	0.902	2.333	-196.399	2.07E-02	2.33E-02	1.45E-01	3.00E+06	1.94E+01
Oesophagus	THC.Herb	2.206	0.994	2.220	-146.903	2.77E-02	2.93E-02	1.45E-01	9.99E+05	9.00E+00
Thyroid	Income	0.381	0.079	4.851	-38.321	2.64E-06	1.30E-05	1.16E-04	1.09E+01	5.11E+00
Breast	Income	0.136	0.028	4.834	-433.783	2.89E-06	1.30E-05	1.24E-04	7.99E+00	4.17E+00
Gallbladder & Biliary	Alcohol	0.191	0.053	3.580	-205.889	4.57E-04	8.32E-04	1.19E-02	7.48E+00	3.16E+00
Myeloma	Income	0.162	0.040	4.072	-291.582	6.98E-05	1.98E-04	2.51E-03	5.85E+00	3.06E+00
Oropharynx_Broad	Income	0.126	0.052	2.427	-101.716	2.12E-02	2.34E-02	1.45E-01	3.37E+01	2.86E+00
Lung	Alcohol	0.039	0.009	4.221	-545.192	3.88E-05	1.24E-04	1.51E-03	3.78E+00	2.41E+00
Leukaemia - Myeloid	Tobacco	0.120	0.025	4.724	-16.581	3.90E-05	1.24E-04	1.51E-03	3.33E+00	2.33E+00
Colorectum	Alcohol	0.093	0.021	4.507	-230.050	1.18E-05	4.24E-05	4.80E-04	3.34E+00	2.29E+00
Oesophagus	Income	0.166	0.052	3.167	-146.903	1.81E-03	2.79E-03	3.62E-02	4.81E+00	2.28E+00
Hodgkins	THC.Resin	0.595	0.270	2.209	-176.757	2.83E-02	2.94E-02	1.45E-01	3.39E+01	2.12E+00
All Cancers nNMSC	Tobacco	0.021	0.004	5.696	-423.961	9.55E-08	5.55E-07	4.39E-06	2.32E+00	1.91E+00
Anus	Income	0.136	0.052	2.608	-204.465	9.88E-03	1.19E-02	9.88E-02	4.14E+00	1.78E+00
Hodgkins	Income	0.117	0.047	2.506	-176.757	1.30E-02	1.53E-02	1.17E-01	2.90E+00	1.52E+00
Myeloma	Tobacco	0.022	0.005	4.509	-291.582	1.17E-05	4.24E-05	4.80E-04	1.62E+00	1.41E+00

Prostate	Tobacco	0.025	0.006	3.773	-201.735	2.20E-04	4.74E-04	6.59E-03	1.67E+00	1.40E+00
Larynx	Tobacco	0.029	0.007	3.885	-93.247	1.44E-04	3.37E-04	4.60E-03	1.60E+00	1.37E+00
Hodgkins	Tobacco	0.020	0.005	4.038	-176.757	7.70E-05	2.08E-04	2.70E-03	1.43E+00	1.28E+00
Oesophagus	Tobacco	0.021	0.007	3.069	-146.903	2.48E-03	3.45E-03	4.47E-02	1.52E+00	1.27E+00
Breast	Tobacco	0.011	0.004	3.023	-433.783	2.87E-03	3.69E-03	4.47E-02	1.50E+00	1.25E+00
Non-Hodgkins lymphoma	Tobacco	0.012	0.005	2.417	-362.362	1.67E-02	1.91E-02	1.33E-01	1.50E+00	1.18E+00
Ovary	Tobacco	0.009	0.004	2.114	-351.253	3.59E-02	3.59E-02	1.45E-01	1.39E+00	1.09E+00

Supplementary Table S25.: Summary Table for Significant Positive Terms from Mixed Effects Additive Regression

Term	Count	Negative Total of P-Value Exponents	Mean of the Negative P-Value Exponents	Median of the Negative P-Value Exponents	Total of the Lower E-Value Exponents	Mean of the Lower E-Value Exponents	Median of the Lower E-Value Exponents
Last Month Cannabis	19	189	9.95	8	341	17.95	17
Herb.THC	21	551	26.24	18	165	7.86	7
Resin.THC	5	13	2.60	2	5	1.00	0
Income	7	29	4.14	5	1	0.14	0
Alcohol	4	17	4.25	2	0	0	0
Tobacco	14	55	3.93	2.5	0	0	0

Supplementary Table S26.: Complete Output from Additive Mixed Effects Regression

Cancer	Term	β-Estimate	Std. Error	P-Value	E-Value Estimate	95% Lower Bound of the E-Value
All Cancers nNMSC	LM.Cannabis	28.793	2.305	7.96E-26	3.50E+60	1.29E+51
Myeloma	LM.Cannabis	18.834	2.110	1.64E-16	1.79E+43	6.91E+33
Lung	LM.Cannabis	27.039	2.293	1.32E-25	7.26E+39	2.00E+33
Kidney	LM.Cannabis	34.251	2.951	4.67E-25	1.70E+39	4.68E+32
Pancreas	LM.Cannabis	29.502	2.956	7.13E-20	5.97E+33	1.65E+27
Leukaemia - Lymphoid	LM.Cannabis	9.998	2.584	3.66E-04	1.09E+45	2.64E+22
All Cancers nNMSC	THC.Herb	10.642	0.521	1.15E-47	3.69E+22	2.73E+20
Non-Hodgkins lymphoma	LM.Cannabis	26.819	3.429	1.75E-13	3.42E+26	9.39E+19
Colorectum	LM.Cannabis	18.142	2.303	1.13E-13	5.83E+25	2.76E+19
Prostate	LM.Cannabis	22.525	3.112	6.20E-12	3.77E+24	1.04E+18
All Cancers	LM.Cannabis	17.153	4.312	1.51E-04	4.43E+34	5.71E+17
Pancreas	THC.Herb	15.302	0.584	1.27E-72	4.61E+17	2.33E+16
Hodgkins	LM.Cannabis	13.231	2.507	2.91E-07	3.05E+25	1.41E+16
Stomach	LM.Cannabis	20.239	3.032	1.67E-10	4.86E+22	1.34E+16
Stomach	THC.Herb	15.302	0.599	1.20E-70	1.68E+17	8.51E+15
Prostate	THC.Herb	15.119	0.616	2.58E-67	3.94E+16	1.98E+15
Breast	LM.Cannabis	13.958	2.225	1.66E-09	2.18E+21	5.99E+14
Kidney	THC.Herb	13.038	0.583	8.04E-61	1.32E+15	6.66E+13
Lung	THC.Herb	9.751	0.453	8.43E-58	3.69E+14	1.86E+13
All Cancers	THC.Herb	9.102	1.851	4.52E-06	3.37E+18	1.89E+11
Breast	THC.Herb	7.398	0.440	2.23E-42	2.83E+11	1.43E+10
Melanoma	LM.Cannabis	19.128	4.101	5.13E-06	8.80E+15	2.42E+09
Non-Hodgkins lymphoma	THC.Herb	10.009	0.679	2.68E-35	1.24E+10	6.20E+08
Oropharynx	THC.Herb	17.962	5.844	3.42E-03	4.75E+19	2.22E+07

Corpus Uteri	THC.Herb	10.573	0.871	7.86E-27	2.06E+08	1.06E+07
Cervix	THC.Herb	7.048	0.692	1.79E-20	1.15E+07	5.77E+05
Oropharynx	THC.Resin	7.056	1.081	3.30E-08	8.17E+07	4.28E+05
Colorectum	THC.Herb	4.636	0.467	1.03E-19	6.43E+06	3.36E+05
Myeloma	THC.Herb	4.293	1.067	7.80E-05	1.24E+10	2.16E+05
Bladder	LM.Cannabis	12.723	3.734	7.68E-04	5.35E+11	1.47E+05
Larynx	LM.Cannabis	21.062	6.232	8.46E-04	4.31E+11	1.19E+05
Oesophagus	LM.Cannabis	21.862	6.703	1.27E-03	1.73E+11	4.78E+04
Ovary	THC.Herb	6.596	0.867	6.26E-13	2.24E+05	1.13E+04
Larynx	THC.Herb	8.671	1.231	1.95E-11	9.27E+04	4.69E+03
Liver	THC.Herb	6.224	1.927	1.44E-03	1.64E+08	2.64E+03
Melanoma	THC.Herb	5.221	0.810	6.37E-10	3.72E+04	1.88E+03
Oropharynx	Income	2.004	0.345	4.46E-07	2.90E+02	5.37E+01
Bladder	THC.Herb	2.976	0.738	7.35E-05	9.41E+02	4.71E+01
Liver	LM.Cannabis	8.543	3.824	2.66E-02	1.45E+11	4.45E+01
Oesophagus	THC.Herb	5.217	1.324	1.07E-04	8.14E+02	4.07E+01
Testis	Income	0.568	0.090	1.72E-09	1.90E+01	9.07E+00
Brain	THC.Herb	3.806	1.278	3.20E-03	1.89E+02	9.00E+00
Gallbladder & Biliary	Income	0.600	0.123	2.23E-06	2.35E+01	8.33E+00
Thyroid	THC.Resin	1.567	0.533	3.62E-03	6.10E+01	5.75E+00
Anus	Income	0.347	0.069	1.09E-06	1.18E+01	5.52E+00
Myeloma	THC.Resin	0.632	0.222	4.79E-03	5.48E+01	5.11E+00
Gallbladder & Biliary	Alcohol	0.296	0.037	1.09E-13	6.27E+00	4.48E+00
Ovary	LM.Cannabis	9.010	4.381	4.08E-02	1.58E+07	3.76E+00
Myeloma	Income	0.272	0.069	1.06E-04	7.82E+00	3.53E+00
Oropharynx	Tobacco	0.298	0.037	1.99E-10	3.61E+00	2.89E+00
All Cancers nNMSC	THC.Resin	0.643	0.290	2.78E-02	4.38E+01	2.24E+00
Leukaemia - Myeloid	Alcohol	0.164	0.062	1.15E-02	5.24E+00	1.96E+00
Testis	Alcohol	0.126	0.035	3.89E-04	2.70E+00	1.83E+00
Hodgkins	THC.Resin	0.564	0.263	3.29E-02	2.32E+01	1.80E+00

Prostate	Tobacco	0.089	0.011	2.47E-13	1.80E+00	1.64E+00
Corpus Uteri	Tobacco	0.085	0.016	1.05E-07	1.59E+00	1.43E+00
Breast	Income	0.145	0.060	1.72E-02	2.69E+00	1.42E+00
Myeloma	Tobacco	0.029	0.006	2.86E-06	1.60E+00	1.42E+00
Anus	Alcohol	0.066	0.027	1.40E-02	2.18E+00	1.36E+00
Breast	Tobacco	0.037	0.008	1.21E-05	1.53E+00	1.36E+00
Hodgkins	Income	0.176	0.082	3.25E-02	3.75E+00	1.35E+00
Kidney	Tobacco	0.045	0.011	4.32E-05	1.50E+00	1.32E+00
Lung	Tobacco	0.032	0.008	1.76E-04	1.47E+00	1.29E+00
Hodgkins	Tobacco	0.023	0.007	1.13E-03	1.45E+00	1.25E+00
Non-Hodgkins lymphoma	Tobacco	0.041	0.013	1.63E-03	1.42E+00	1.23E+00
Pancreas	Tobacco	0.031	0.011	3.93E-03	1.39E+00	1.19E+00
Colorectum	Tobacco	0.025	0.009	4.11E-03	1.39E+00	1.19E+00
All Cancers	Tobacco	0.039	0.017	2.56E-02	1.68E+00	1.19E+00
Stomach	Tobacco	0.030	0.011	7.66E-03	1.37E+00	1.17E+00
Ovary	Tobacco	0.033	0.016	4.18E-02	1.31E+00	1.05E+00
Thyroid	Tobacco	0.002	0.014	8.68E-01	1.08E+00	1.00E+00
Melanoma	Tobacco	0.010	0.015	5.08E-01	1.16E+00	1.00E+00
Brain	Income	0.021	0.175	9.06E-01	1.19E+00	1.00E+00
Oesophagus	Tobacco	0.022	0.024	3.72E-01	1.19E+00	1.00E+00
Bladder	Tobacco	0.013	0.014	3.49E-01	1.19E+00	1.00E+00
Brain	Tobacco	0.029	0.024	2.30E-01	1.22E+00	1.00E+00
Larynx	Tobacco	0.033	0.023	1.46E-01	1.25E+00	1.00E+00
Liver	Tobacco	0.017	0.011	1.39E-01	1.28E+00	1.00E+00
All Cancers nNMSC	Tobacco	0.015	0.008	6.12E-02	1.36E+00	1.00E+00
Bladder	Income	0.040	0.101	6.93E-01	1.39E+00	1.00E+00
Oropharynx_Broad	Tobacco	0.077	0.098	4.38E-01	1.41E+00	1.00E+00
Lung	THC.Resin	0.064	0.340	8.50E-01	1.79E+00	1.00E+00
Thyroid	Alcohol	0.119	0.064	6.56E-02	1.92E+00	1.00E+00
Melanoma	Income	0.176	0.111	1.16E-01	2.13E+00	1.00E+00

Thyroid	Income	0.161	0.166	3.31E-01	2.20E+00	1.00E+00
Oropharynx_Broad	Income	0.337	1.068	7.54E-01	2.32E+00	1.00E+00
Bladder	THC.Resin	0.191	0.553	7.30E-01	2.33E+00	1.00E+00
Corpus Uteri	THC.Resin	0.271	0.655	6.80E-01	2.59E+00	1.00E+00
Leukaemia - Lymphoid	Alcohol	0.069	0.039	8.60E-02	3.48E+00	1.00E+00
Oropharynx_Broad	THC.Resin	0.740	5.689	8.97E-01	4.15E+00	1.00E+00
Oesophagus	THC.Resin	0.898	0.993	3.67E-01	5.07E+00	1.00E+00
Melanoma	THC.Resin	0.765	0.608	2.09E-01	7.91E+00	1.00E+00
Non-Hodgkins lymphoma	THC.Resin	0.769	0.510	1.33E-01	1.08E+01	1.00E+00
Kidney	THC.Resin	0.826	0.437	6.02E-02	1.68E+01	1.00E+00
Testis	THC.Herb	1.009	1.400	4.72E-01	1.14E+02	1.00E+00
Thyroid	THC.Herb	1.920	2.562	4.55E-01	1.32E+02	1.00E+00
Brain	LM.Cannabis	3.517	6.460	5.87E-01	1.34E+02	1.00E+00
Cervix	LM.Cannabis	1.909	3.498	5.86E-01	1.35E+02	1.00E+00
Anus	LM.Cannabis	0.927	2.123	6.63E-01	2.58E+02	1.00E+00
Hodgkins	THC.Herb	1.270	1.270	3.18E-01	5.22E+02	1.00E+00
Anus	THC.Herb	1.926	1.069	7.28E-02	4.85E+04	1.00E+00
Corpus Uteri	LM.Cannabis	6.268	4.329	1.49E-01	1.13E+05	1.00E+00
Oropharynx_Broad	THC.Herb	12.626	13.390	3.50E-01	4.65E+06	1.00E+00
Thyroid	LM.Cannabis	7.763	5.069	1.27E-01	4.69E+07	1.00E+00
Testis	LM.Cannabis	4.673	2.771	9.31E-02	2.75E+08	1.00E+00
Oropharynx_Broad	LM.Cannabis	24.051	51.031	6.39E-01	2.68E+12	1.00E+00
Testis	Tobacco	-0.001	0.008	8.54E-01	1.08E+00	-
Prostate	Income	-0.005	0.084	9.49E-01	1.13E+00	-
Colorectum	Alcohol	-0.005	0.022	8.18E-01	1.14E+00	-
Cervix	Tobacco	-0.022	0.013	8.22E-02	1.28E+00	-
Anus	Tobacco	-0.012	0.006	4.36E-02	1.33E+00	-
Gallbladder & Biliary	Tobacco	-0.018	0.008	2.36E-02	1.37E+00	-
Hodgkins	Alcohol	-0.021	0.032	5.00E-01	1.43E+00	-
Oesophagus	Income	-0.082	0.182	6.52E-01	1.43E+00	-

Oesophagus	Alcohol	-0.083	0.071	2.49E-01	1.43E+00	-
Corpus Uteri	Alcohol	-0.062	0.047	1.87E-01	1.47E+00	-
Liver	Alcohol	-0.038	0.049	4.36E-01	1.48E+00	-
Myeloma	Alcohol	-0.021	0.027	4.26E-01	1.48E+00	-
Larynx	THC.Resin	-0.107	0.923	9.08E-01	1.54E+00	-
Ovary	Alcohol	-0.080	0.047	8.86E-02	1.57E+00	-
Larynx	Alcohol	-0.117	0.066	8.00E-02	1.58E+00	-
Bladder	Alcohol	-0.084	0.040	3.57E-02	1.66E+00	-
Brain	Alcohol	-0.148	0.069	3.24E-02	1.67E+00	-
Oropharynx_Broad	Alcohol	-0.169	0.310	5.89E-01	1.73E+00	-
Non-Hodgkins lymphoma	Income	-0.113	0.093	2.27E-01	1.90E+00	-
Melanoma	Alcohol	-0.147	0.044	9.04E-04	1.97E+00	-
Cervix	Alcohol	-0.132	0.037	4.74E-04	2.01E+00	-
Breast	Alcohol	-0.085	0.024	3.91E-04	2.02E+00	-
Oropharynx	Alcohol	-0.120	0.072	1.03E-01	2.03E+00	-
Corpus Uteri	Income	-0.201	0.118	8.98E-02	2.20E+00	-
Breast	THC.Resin	-0.109	0.330	7.41E-01	2.28E+00	-
Non-Hodgkins lymphoma	Alcohol	-0.187	0.037	6.54E-07	2.42E+00	-
Ovary	Income	-0.268	0.119	2.48E-02	2.59E+00	-
Kidney	Alcohol	-0.181	0.031	2.46E-08	2.59E+00	-
Pancreas	THC.Resin	-0.186	0.438	6.72E-01	2.63E+00	-
Stomach	Alcohol	-0.193	0.032	8.76E-09	2.65E+00	-
Prostate	Alcohol	-0.209	0.033	1.40E-09	2.75E+00	-
Liver	Income	-0.178	0.125	1.57E-01	2.75E+00	-
All Cancers nNMSC	Alcohol	-0.116	0.024	3.18E-06	2.88E+00	-
Pancreas	Alcohol	-0.232	0.031	2.80E-12	3.07E+00	-
Colorectum	Income	-0.188	0.063	3.09E-03	3.07E+00	-
Leukaemia - Lymphoid	Tobacco	-0.064	0.012	1.82E-06	3.28E+00	-
Lung	Alcohol	-0.205	0.024	4.27E-15	3.40E+00	-
Brain	THC.Resin	-0.587	0.959	5.41E-01	3.45E+00	-

Prostate	THC.Resin	-0.327	0.462	4.79E-01	3.94E+00	-
Larynx	Income	-0.685	0.169	6.69E-05	4.10E+00	-
Testis	THC.Resin	-0.235	0.291	4.21E-01	4.56E+00	-
Leukaemia - Myeloid	Tobacco	-0.170	0.018	1.05E-11	5.46E+00	-
All Cancers	Alcohol	-0.245	0.042	1.03E-07	5.63E+00	-
Anus	THC.Resin	-0.246	0.223	2.70E-01	6.72E+00	-
Colorectum	THC.Resin	-0.485	0.298	1.05E-01	9.05E+00	-
Kidney	Income	-0.652	0.080	1.83E-14	1.05E+01	-
Pancreas	Income	-0.714	0.080	1.19E-16	1.24E+01	-
Cervix	Income	-0.862	0.095	4.22E-17	1.29E+01	-
Stomach	Income	-0.774	0.082	3.58E-18	1.38E+01	-
Ovary	THC.Resin	-1.133	0.650	8.28E-02	1.42E+01	-
Lung	Income	-0.595	0.062	1.40E-18	1.43E+01	-
Leukaemia - Lymphoid	Income	-0.197	0.147	1.88E-01	1.46E+01	-
Gallbladder & Biliary	THC.Resin	-0.516	0.309	9.64E-02	1.65E+01	-
All Cancers	Income	-0.466	0.195	1.92E-02	1.66E+01	-
All Cancers nNMSC	Income	-0.490	0.060	4.78E-14	2.07E+01	-
Liver	THC.Resin	-1.291	0.404	1.60E-03	8.71E+01	-
All Cancers	THC.Resin	-0.925	0.516	7.67E-02	1.42E+02	-
Cervix	THC.Resin	-2.040	0.519	1.13E-04	1.80E+02	-
Stomach	THC.Resin	-1.985	0.449	1.50E-05	3.13E+02	-
Leukaemia - Myeloid	Income	-1.042	0.233	5.77E-05	1.71E+03	-
Leukaemia - Myeloid	LM.Cannabis	-1.220	4.108	7.68E-01	5.44E+03	-
Leukaemia - Lymphoid	THC.Resin	-1.416	0.528	1.04E-02	4.35E+06	-
Oropharynx	LM.Cannabis	-7.738	6.586	2.46E-01	4.44E+08	-
Gallbladder & Biliary	LM.Cannabis	-5.497	3.949	1.65E-01	1.57E+10	-
Leukaemia - Myeloid	THC.Resin	-3.689	0.840	7.16E-05	4.86E+10	-
Leukaemia - Lymphoid	THC.Herb	-5.101	2.309	3.25E-02	1.34E+23	-
Gallbladder & Biliary	THC.Herb	-13.766	1.840	2.40E-12	1.20E+25	-
Leukaemia - Myeloid	THC.Herb	-10.232	3.671	7.87E-03	1.27E+29	-

Supplementary Table S27.: Complete Model Output from Interactive Panel Regression

Cancer	Term	ß-Estimate	Std. Error	P-Value	E-Value Estimate	95% Lower Bound of the E-Value
Colorectum	THC.Herb	55.387	5.081	1.17E-26	8.51E+30	2.72E+25
Breast	THC.Herb	37.005	3.771	4.69E-22	7.65E+27	2.43E+22
Gallbladder & Biliary	THC.Herb	20.744	2.892	1.41E-12	1.84E+26	1.53E+19
Oropharynx_Broad	THC.Herb	23.856	4.274	4.15E-08	1.31E+26	1.17E+17
All Cancers	THC.Herb	12.034	2.019	4.45E-09	1.41E+23	4.48E+15
Thyroid	THC.Herb	20.386	3.101	7.61E-11	2.72E+21	1.40E+15
Anus	THC.Herb	13.789	2.229	8.74E-10	1.74E+20	8.63E+13
Testis	THC.Herb	33.843	6.241	7.22E-08	5.43E+17	2.80E+11
Stomach	THC.Herb	24.735	4.252	7.31E-09	4.63E+16	1.46E+11
Oropharynx	THC.Resin	7.349	0.625	4.34E-22	1.92E+11	2.86E+09
Corpus Uteri	THC.Herb	25.436	5.293	1.70E-06	6.37E+13	2.03E+08
Prostate	THC.Herb	24.791	5.498	7.03E-06	9.35E+12	2.98E+07
Oesophagus	THC.Herb	13.982	3.228	1.58E-05	3.05E+12	9.59E+06
Leukaemia - Lymphoid	LM.Cannabis:THC.Herb	7.397	3.030	1.57E-02	2.60E+23	7.91E+04
Melanoma	THC.Herb	10.965	3.151	5.17E-04	1.26E+10	3.90E+04
Cervix	THC.Herb	13.081	5.359	1.48E-02	1.47E+07	4.57E+01
Oesophagus	THC.Resin	1.763	0.155	1.11E-28	6.82E+01	3.68E+01
All Cancers nNMSC	Tobacco:THC.Herb	0.527	0.059	9.85E-19	4.86E+01	2.39E+01
Oropharynx	Income	1.024	0.191	3.81E-07	6.72E+01	1.82E+01
Stomach	LM.Cannabis	1.283	0.096	3.16E-38	1.36E+01	1.01E+01
Kidney	THC.Herb	6.010	2.758	2.95E-02	2.69E+06	7.94E+00
Colorectum	LM.Cannabis	1.323	0.115	2.41E-29	1.03E+01	7.56E+00
Myeloma	THC.Resin	0.526	0.090	6.60E-09	1.43E+01	7.06E+00

Ovary	THC.Herb	9.381	4.345	3.10E-02	2.37E+06	6.91E+00
Larynx	THC.Resin	0.796	0.145	4.56E-08	1.06E+01	5.48E+00
Larynx	LM.Cannabis	0.612	0.068	8.34E-19	6.93E+00	5.05E+00
Leukaemia - Lymphoid	Alcohol	0.164	0.021	4.81E-13	5.96E+00	4.29E+00
Breast	LM.Cannabis	0.628	0.086	3.61E-13	5.32E+00	3.83E+00
Thyroid	LM.Cannabis	0.433	0.065	4.51E-11	5.07E+00	3.57E+00
Pancreas	THC.Herb	5.546	2.709	4.09E-02	1.14E+06	3.00E+00
Hodgkins	LM.Cannabis	0.253	0.044	1.54E-08	4.28E+00	2.98E+00
Gallbladder & Biliary	Tobacco:LM.Cannabis:THC.Herb	0.266	0.034	7.85E-15	3.72E+00	2.95E+00
Leukaemia - Myeloid	LM.Cannabis:THC.Herb	10.783	5.449	4.96E-02	1.08E+19	2.67E+00
Oropharynx	Tobacco:THC.Herb	0.509	0.187	7.31E-03	1.10E+01	2.66E+00
Leukaemia - Myeloid	Alcohol	0.195	0.037	6.00E-07	3.78E+00	2.63E+00
Corpus Uteri	LM.Cannabis	0.611	0.120	3.94E-07	3.64E+00	2.55E+00
Gallbladder & Biliary	LM.Cannabis	0.252	0.055	5.50E-06	3.55E+00	2.40E+00
Colorectum	Tobacco:LM.Cannabis:THC.Herb	0.453	0.069	5.71E-11	2.96E+00	2.37E+00
Myeloma	Income	0.137	0.023	6.68E-09	2.77E+00	2.19E+00
Testis	Income	0.440	0.076	7.35E-09	2.76E+00	2.18E+00
Prostate	LM.Cannabis	0.512	0.125	4.20E-05	3.05E+00	2.08E+00
Prostate	Income	0.372	0.054	9.01E-12	2.47E+00	2.08E+00
Stomach	Tobacco:LM.Cannabis:THC.Herb	0.317	0.058	4.27E-08	2.63E+00	2.07E+00
Thyroid	Tobacco:LM.Cannabis:THC.Herb	0.202	0.038	9.80E-08	2.62E+00	2.06E+00
All Cancers	Alcohol	0.092	0.013	5.34E-13	2.36E+00	2.03E+00
Oropharynx	Tobacco	0.148	0.033	1.44E-05	2.71E+00	2.00E+00
Breast	Tobacco:LM.Cannabis:THC.Herb	0.267	0.051	1.98E-07	2.54E+00	2.00E+00
Anus	Tobacco:LM.Cannabis:THC.Herb	0.138	0.027	4.91E-07	2.55E+00	1.98E+00
Breast	Income	0.216	0.037	8.22E-09	2.25E+00	1.87E+00
Non-Hodgkins lymphoma	Tobacco:THC.Herb	0.314	0.103	2.26E-03	3.15E+00	1.82E+00
Lung	Tobacco:THC.Herb	0.186	0.061	2.51E-03	3.11E+00	1.80E+00
Brain	Income	0.136	0.027	7.04E-07	2.09E+00	1.72E+00
Gallbladder & Biliary	Tobacco	0.072	0.005	1.16E-41	1.76E+00	1.68E+00

Larynx	Alcohol	0.097	0.010	8.57E-22	1.76E+00	1.64E+00
Colorectum	THC.Resin	0.604	0.242	1.28E-02	3.74E+00	1.64E+00
Prostate	Tobacco:LM.Cannabis:THC.Herb	0.253	0.074	6.94E-04	2.03E+00	1.53E+00
Kidney	Tobacco:LM.Cannabis:THC.Herb	0.127	0.037	7.28E-04	2.03E+00	1.52E+00
Colorectum	Tobacco	0.104	0.007	1.16E-46	1.54E+00	1.49E+00
Breast	Tobacco	0.074	0.005	5.07E-43	1.53E+00	1.47E+00
Oesophagus	Tobacco:LM.Cannabis:THC.Herb	0.136	0.044	1.86E-03	1.96E+00	1.45E+00
Stomach	Tobacco	0.076	0.006	3.92E-37	1.50E+00	1.44E+00
Colorectum	Alcohol	0.103	0.017	7.58E-10	1.54E+00	1.41E+00
Pancreas	Tobacco:LM.Cannabis:THC.Herb	0.108	0.037	3.44E-03	1.91E+00	1.40E+00
Ovary	LM.Cannabis	0.249	0.099	1.16E-02	2.26E+00	1.40E+00
Corpus Uteri	Tobacco	0.077	0.007	1.75E-25	1.43E+00	1.37E+00
Oropharynx	Tobacco:LM.Cannabis	0.060	0.020	2.75E-03	1.76E+00	1.37E+00
All Cancers nNMSC	Tobacco:LM.Cannabis	0.014	0.001	1.10E-28	1.41E+00	1.36E+00
Ovary	Tobacco	0.056	0.006	3.11E-20	1.39E+00	1.34E+00
Prostate	Tobacco	0.068	0.008	7.41E-19	1.38E+00	1.33E+00
Corpus Uteri	Tobacco:LM.Cannabis:THC.Herb	0.188	0.072	8.51E-03	1.83E+00	1.32E+00
Hodgkins	Tobacco:LM.Cannabis:THC.Herb	0.067	0.026	9.15E-03	1.84E+00	1.31E+00
Bladder	THC.Resin	0.266	0.125	3.30E-02	3.30E+00	1.30E+00
Testis	Tobacco	0.063	0.009	2.70E-11	1.37E+00	1.29E+00
Prostate	Alcohol	0.075	0.018	3.02E-05	1.41E+00	1.27E+00
Oesophagus	Alcohol	0.041	0.011	1.26E-04	1.39E+00	1.25E+00
Stomach	Alcohol	0.053	0.014	1.41E-04	1.39E+00	1.25E+00
Oropharynx_Broad	Tobacco	0.037	0.012	1.73E-03	1.42E+00	1.23E+00
Larynx	Tobacco	0.024	0.004	9.70E-09	1.29E+00	1.22E+00
Melanoma	Tobacco:LM.Cannabis	0.020	0.002	4.91E-18	1.25E+00	1.22E+00
Liver	Tobacco:LM.Cannabis	0.018	0.002	3.69E-16	1.25E+00	1.21E+00
Cervix	Alcohol	0.059	0.018	8.14E-04	1.36E+00	1.21E+00
Breast	Alcohol	0.041	0.012	1.06E-03	1.35E+00	1.20E+00
Lung	Tobacco:LM.Cannabis	0.011	0.001	5.55E-15	1.23E+00	1.20E+00

Melanoma	Income	0.073	0.031	1.83E-02	1.60E+00	1.19E+00
Bladder	LM.Cannabis	0.126	0.059	3.32E-02	2.08E+00	1.19E+00
Pancreas	Tobacco:LM.Cannabis	0.014	0.002	1.62E-12	1.22E+00	1.18E+00
Melanoma	Tobacco:LM.Cannabis:THC.Herb	0.095	0.043	2.66E-02	1.73E+00	1.18E+00
Oesophagus	Tobacco:LM.Cannabis	0.015	0.002	7.62E-11	1.21E+00	1.17E+00
Kidney	Tobacco:LM.Cannabis	0.013	0.002	2.33E-10	1.21E+00	1.17E+00
Brain	Tobacco:LM.Cannabis:THC.Herb	0.081	0.038	3.08E-02	1.71E+00	1.15E+00
Thyroid	Alcohol	0.027	0.011	1.56E-02	1.33E+00	1.12E+00
Anus	Tobacco:LM.Cannabis	0.006	0.001	6.92E-05	1.16E+00	1.11E+00
Non-Hodgkins lymphoma	Tobacco:LM.Cannabis	0.009	0.002	6.70E-05	1.16E+00	1.11E+00
Testis	Tobacco:LM.Cannabis:THC.Herb	0.154	0.076	4.22E-02	1.69E+00	1.09E+00
All Cancers	Tobacco:LM.Cannabis	0.013	0.006	3.07E-02	1.31E+00	1.08E+00
Cervix	Tobacco	0.017	0.007	1.92E-02	1.17E+00	1.06E+00
Cervix	Tobacco:LM.Cannabis	0.001	0.004	8.96E-01	1.03E+00	1.00E+00
Corpus Uteri	Income	0.002	0.052	9.73E-01	1.05E+00	1.00E+00
Testis	Alcohol	0.002	0.022	9.11E-01	1.06E+00	1.00E+00
Hodgkins	Alcohol	0.004	0.007	5.80E-01	1.14E+00	1.00E+00
Bladder	Tobacco	0.006	0.004	8.73E-02	1.14E+00	1.00E+00
Corpus Uteri	Alcohol	0.013	0.017	4.69E-01	1.14E+00	1.00E+00
Brain	Tobacco	0.007	0.004	6.51E-02	1.15E+00	1.00E+00
All Cancers nNMSC	Tobacco:LM.Cannabis:THC.Herb	0.010	0.020	5.95E-01	1.33E+00	1.00E+00
Leukaemia - Myeloid	Tobacco:LM.Cannabis	0.023	0.017	1.92E-01	1.42E+00	1.00E+00
Cervix	Tobacco:LM.Cannabis:THC.Herb	0.078	0.073	2.82E-01	1.43E+00	1.00E+00
Colorectum	Income	0.079	0.050	1.13E-01	1.45E+00	1.00E+00
Lung	Tobacco:LM.Cannabis:THC.Herb	0.031	0.026	2.33E-01	1.46E+00	1.00E+00
Larynx	Tobacco:LM.Cannabis:THC.Herb	0.050	0.041	2.21E-01	1.47E+00	1.00E+00
Bladder	Tobacco:LM.Cannabis:THC.Herb	0.045	0.035	1.99E-01	1.49E+00	1.00E+00
Kidney	Tobacco:THC.Herb	0.050	0.087	5.66E-01	1.50E+00	1.00E+00
Myeloma	Tobacco:LM.Cannabis:THC.Herb	0.033	0.024	1.64E-01	1.52E+00	1.00E+00
Leukaemia - Lymphoid	Tobacco:LM.Cannabis	0.017	0.010	7.03E-02	1.52E+00	1.00E+00

Bladder	Tobacco:THC.Herb	0.052	0.082	5.29E-01	1.53E+00	1.00E+00
Hodgkins	Income	0.037	0.026	1.50E-01	1.53E+00	1.00E+00
Ovary	Tobacco:LM.Cannabis:THC.Herb	0.087	0.059	1.40E-01	1.54E+00	1.00E+00
Oropharynx_Broad	Income	0.055	0.048	2.57E-01	1.56E+00	1.00E+00
Liver	Tobacco:LM.Cannabis:THC.Herb	0.061	0.040	1.28E-01	1.56E+00	1.00E+00
Non-Hodgkins lymphoma	Tobacco:LM.Cannabis:THC.Herb	0.072	0.045	1.08E-01	1.58E+00	1.00E+00
All Cancers	Tobacco:LM.Cannabis:THC.Herb	0.038	0.062	5.37E-01	1.64E+00	1.00E+00
Liver	Tobacco:THC.Herb	0.076	0.107	4.80E-01	1.66E+00	1.00E+00
Brain	LM.Cannabis	0.084	0.063	1.85E-01	1.73E+00	1.00E+00
Brain	Tobacco:THC.Herb	0.084	0.088	3.35E-01	1.73E+00	1.00E+00
Pancreas	Tobacco:THC.Herb	0.094	0.085	2.70E-01	1.82E+00	1.00E+00
Myeloma	Tobacco:THC.Herb	0.069	0.062	2.67E-01	1.93E+00	1.00E+00
Myeloma	LM.Cannabis	0.070	0.041	8.42E-02	1.94E+00	1.00E+00
Testis	LM.Cannabis	0.237	0.131	7.07E-02	1.98E+00	1.00E+00
Lung	THC.Resin	0.091	0.093	3.28E-01	2.04E+00	1.00E+00
Hodgkins	THC.Resin	0.114	0.098	2.43E-01	2.35E+00	1.00E+00
Non-Hodgkins lymphoma	THC.Resin	0.236	0.159	1.38E-01	2.59E+00	1.00E+00
Oropharynx_Broad	Tobacco:LM.Cannabis:THC.Herb	0.261	0.192	1.74E-01	3.24E+00	1.00E+00
Myeloma	THC.Herb	0.221	1.940	9.09E-01	4.08E+00	1.00E+00
Oropharynx_Broad	LM.Cannabis	0.546	0.682	4.24E-01	7.26E+00	1.00E+00
All Cancers	LM.Cannabis:THC.Herb	0.547	2.023	7.87E-01	2.13E+01	1.00E+00
Liver	THC.Herb	1.938	3.383	5.67E-01	1.59E+02	1.00E+00
Bladder	THC.Herb	2.276	2.597	3.81E-01	5.84E+02	1.00E+00
Leukaemia - Lymphoid	THC.Herb	1.115	3.638	7.60E-01	6.09E+03	1.00E+00
Hodgkins	THC.Herb	2.749	2.111	1.93E-01	3.07E+04	1.00E+00
Larynx	THC.Herb	5.154	3.009	8.69E-02	1.32E+05	1.00E+00
Oropharynx	LM.Cannabis:THC.Herb	6.114	5.998	3.10E-01	2.74E+09	1.00E+00
Kaposi	Tobacco	6.014	25.670	8.16E-01	1.67E+12	1.00E+00
Leukaemia - Myeloid	THC.Herb	9.240	6.543	1.60E-01	2.25E+16	1.00E+00
Kaposi	Tobacco:LM.Cannabis:THC.Herb	21.495	27.173	4.32E-01	8.17E+42	1.00E+00

Kaposi	LM.Cannabis	75.351	116.035	5.18E-01	4.77E+149	1.00E+00
Kaposi	LM.Cannabis:THC.Herb	551.469	1246.298	6.60E-01	Inf	1.00E+00
Myeloma	Tobacco:LM.Cannabis	-0.001	0.001	5.45E-01	1.06E+00	-
Bladder	Tobacco:LM.Cannabis	-0.003	0.002	1.52E-01	1.09E+00	-
Brain	Tobacco:LM.Cannabis	-0.003	0.002	9.85E-02	1.10E+00	-
Thyroid	Income	-0.004	0.038	9.14E-01	1.11E+00	-
Testis	Tobacco:LM.Cannabis	-0.009	0.004	2.78E-02	1.12E+00	-
Hodgkins	Tobacco	-0.004	0.003	2.60E-01	1.13E+00	-
Ovary	Tobacco:LM.Cannabis	-0.009	0.003	6.14E-03	1.13E+00	-
Prostate	Tobacco:LM.Cannabis	-0.016	0.004	7.03E-05	1.16E+00	-
Corpus Uteri	Tobacco:LM.Cannabis	-0.019	0.004	6.98E-07	1.18E+00	-
Hodgkins	Tobacco:LM.Cannabis	-0.007	0.001	3.26E-07	1.19E+00	-
Thyroid	Tobacco:LM.Cannabis	-0.013	0.002	9.72E-10	1.21E+00	-
Thyroid	Tobacco	-0.013	0.005	4.15E-03	1.21E+00	-
Larynx	Tobacco:LM.Cannabis	-0.015	0.002	1.82E-12	1.22E+00	-
Breast	Tobacco:LM.Cannabis	-0.019	0.003	1.62E-12	1.22E+00	-
Gallbladder & Biliary	Tobacco:LM.Cannabis	-0.012	0.002	2.18E-11	1.22E+00	-
Oropharynx_Broad	Tobacco:LM.Cannabis	-0.015	0.021	4.70E-01	1.24E+00	-
Ovary	Income	-0.026	0.043	5.38E-01	1.24E+00	-
Ovary	Alcohol	-0.027	0.014	5.94E-02	1.25E+00	-
Kidney	Tobacco	-0.018	0.004	1.78E-06	1.26E+00	-
All Cancers	Tobacco	-0.010	0.003	4.35E-04	1.27E+00	-
Anus	Tobacco	-0.014	0.003	2.93E-05	1.27E+00	-
Oesophagus	Tobacco	-0.024	0.004	5.91E-08	1.28E+00	-
Lung	Tobacco	-0.015	0.003	5.65E-08	1.28E+00	-
Colorectum	Tobacco:LM.Cannabis	-0.039	0.004	1.08E-25	1.28E+00	-
Myeloma	Alcohol	-0.013	0.007	5.53E-02	1.28E+00	-
Pancreas	Tobacco	-0.022	0.004	5.76E-09	1.29E+00	-
Stomach	Tobacco:LM.Cannabis	-0.038	0.003	2.14E-34	1.31E+00	-
Melanoma	Tobacco	-0.030	0.004	3.36E-12	1.33E+00	-

Oropharynx_Broad	Alcohol	-0.027	0.045	5.52E-01	1.34E+00	-
Myeloma	Tobacco	-0.018	0.003	1.43E-09	1.34E+00	-
Brain	Alcohol	-0.031	0.009	7.42E-04	1.36E+00	-
Non-Hodgkins lymphoma	Tobacco	-0.048	0.004	1.87E-25	1.43E+00	-
Liver	Tobacco	-0.049	0.005	1.39E-22	1.48E+00	-
Cervix	LM.Cannabis	-0.105	0.121	3.89E-01	1.53E+00	-
Anus	Alcohol	-0.040	0.008	4.55E-07	1.55E+00	-
Oesophagus	Income	-0.079	0.032	1.25E-02	1.62E+00	-
Ovary	Tobacco:THC.Herb	-0.109	0.137	4.28E-01	1.63E+00	-
Non-Hodgkins lymphoma	Alcohol	-0.083	0.011	2.30E-14	1.64E+00	-
Gallbladder & Biliary	Income	-0.063	0.033	5.28E-02	1.69E+00	-
Anus	Income	-0.057	0.027	3.51E-02	1.71E+00	-
Lung	Alcohol	-0.057	0.006	7.00E-19	1.71E+00	-
Leukaemia - Myeloid	Tobacco	-0.049	0.026	6.30E-02	1.73E+00	-
Bladder	Income	-0.088	0.026	5.83E-04	1.80E+00	-
Kidney	Alcohol	-0.100	0.009	5.42E-27	1.84E+00	-
Larynx	Tobacco:THC.Herb	-0.111	0.095	2.40E-01	1.86E+00	-
Leukaemia - Lymphoid	Tobacco	-0.034	0.014	2.18E-02	1.86E+00	-
Melanoma	Alcohol	-0.119	0.010	4.98E-29	1.87E+00	-
All Cancers nNMSC	Tobacco	-0.044	0.004	1.73E-31	1.93E+00	-
Larynx	Income	-0.123	0.030	3.24E-05	1.93E+00	-
Cervix	Income	-0.230	0.053	1.35E-05	1.97E+00	-
Bladder	Alcohol	-0.121	0.009	6.11E-43	2.05E+00	-
Cervix	Tobacco:THC.Herb	-0.253	0.169	1.34E-01	2.06E+00	-
Pancreas	Alcohol	-0.131	0.009	1.15E-45	2.08E+00	-
Leukaemia - Lymphoid	THC.Resin	-0.044	0.397	9.12E-01	2.09E+00	-
Hodgkins	Tobacco:THC.Herb	-0.091	0.068	1.80E-01	2.09E+00	-
Liver	Alcohol	-0.158	0.012	3.50E-39	2.22E+00	-
Non-Hodgkins lymphoma	Income	-0.204	0.032	3.24E-10	2.37E+00	-
Gallbladder & Biliary	Alcohol	-0.143	0.011	7.26E-39	2.39E+00	-

Liver	Income	-0.205	0.040	3.08E-07	2.56E+00	-
Melanoma	Tobacco:THC.Herb	-0.238	0.099	1.67E-02	2.65E+00	-
Breast	THC.Resin	-0.314	0.180	8.23E-02	2.82E+00	-
All Cancers nNMSC	Alcohol	-0.092	0.005	5.85E-81	2.89E+00	-
Oropharynx	Alcohol	-0.167	0.036	1.01E-05	2.95E+00	-
Kaposi	Income	-0.126	0.855	8.83E-01	2.95E+00	-
Stomach	Income	-0.381	0.042	2.75E-19	2.97E+00	-
All Cancers	Income	-0.133	0.024	4.77E-08	2.97E+00	-
Non-Hodgkins lymphoma	LM.Cannabis	-0.304	0.074	4.52E-05	3.08E+00	-
Prostate	Tobacco:THC.Herb	-0.550	0.173	1.54E-03	3.23E+00	-
All Cancers nNMSC	LM.Cannabis:THC.Herb	-0.112	0.626	8.58E-01	3.37E+00	-
Pancreas	Income	-0.293	0.027	4.74E-27	3.44E+00	-
Melanoma	THC.Resin	-0.348	0.152	2.25E-02	3.51E+00	-
Lung	Income	-0.218	0.019	8.37E-29	3.55E+00	-
Oesophagus	Tobacco:THC.Herb	-0.369	0.102	2.97E-04	3.61E+00	-
Anus	LM.Cannabis	-0.222	0.047	2.63E-06	3.61E+00	-
Kidney	Income	-0.317	0.027	3.13E-30	3.63E+00	-
Corpus Uteri	Tobacco:THC.Herb	-0.616	0.167	2.33E-04	3.67E+00	-
Stomach	Tobacco:THC.Herb	-0.494	0.134	2.32E-04	3.67E+00	-
Leukaemia - Lymphoid	Income	-0.111	0.093	2.33E-01	3.88E+00	-
Leukaemia - Myeloid	Income	-0.208	0.167	2.14E-01	4.03E+00	-
Leukaemia - Myeloid	Tobacco:LM.Cannabis:THC.Herb	-0.231	0.159	1.49E-01	4.48E+00	-
Oesophagus	LM.Cannabis	-0.512	0.073	4.08E-12	5.03E+00	-
Kidney	LM.Cannabis	-0.475	0.063	5.14E-14	5.56E+00	-
Anus	Tobacco:THC.Herb	-0.352	0.071	9.03E-07	5.90E+00	-
Pancreas	LM.Cannabis	-0.490	0.061	2.95E-15	5.91E+00	-
Liver	LM.Cannabis	-0.537	0.069	1.66E-14	6.19E+00	-
Testis	Tobacco:THC.Herb	-1.028	0.200	3.15E-07	6.22E+00	-
Lung	LM.Cannabis	-0.371	0.044	1.02E-16	6.31E+00	-
Leukaemia - Lymphoid	Tobacco:LM.Cannabis:THC.Herb	-0.182	0.089	4.12E-02	6.88E+00	-

Leukaemia - Lymphoid	Tobacco:THC.Herb	-0.184	0.095	5.43E-02	6.96E+00	-
All Cancers	Tobacco:THC.Herb	-0.312	0.068	5.62E-06	7.29E+00	-
All Cancers nNMSC	Income	-0.240	0.014	1.39E-57	8.06E+00	-
Melanoma	LM.Cannabis	-0.751	0.072	7.40E-25	8.86E+00	-
Leukaemia - Myeloid	Tobacco:THC.Herb	-0.391	0.170	2.30E-02	9.04E+00	-
Thyroid	Tobacco:THC.Herb	-0.656	0.099	6.03E-11	9.04E+00	-
Oropharynx_Broad	Tobacco:THC.Herb	-0.632	0.141	8.88E-06	9.14E+00	-
Oropharynx	Tobacco:LM.Cannabis:THC.Herb	-0.463	0.174	8.89E-03	9.30E+00	-
Testis	THC.Resin	-1.403	0.289	1.40E-06	1.00E+01	-
Liver	LM.Cannabis:THC.Herb	-0.752	1.283	5.58E-01	1.04E+01	-
Stomach	THC.Resin	-1.228	0.205	2.43E-09	1.25E+01	-
Breast	Tobacco:THC.Herb	-1.091	0.119	1.57E-19	1.25E+01	-
Colorectum	Tobacco:THC.Herb	-1.679	0.160	7.81E-25	1.64E+01	-
Oropharynx_Broad	THC.Resin	-0.866	0.490	7.79E-02	1.68E+01	-
Corpus Uteri	THC.Resin	-1.807	0.253	1.35E-12	1.77E+01	-
Cervix	LM.Cannabis:THC.Herb	-1.859	2.312	4.21E-01	1.84E+01	-
Brain	THC.Resin	-1.029	0.134	2.63E-14	2.15E+01	-
Brain	THC.Herb	-1.029	2.780	7.11E-01	2.15E+01	-
Gallbladder & Biliary	Tobacco:THC.Herb	-0.852	0.094	5.93E-19	2.28E+01	-
Lung	LM.Cannabis:THC.Herb	-0.835	0.837	3.18E-01	3.15E+01	-
All Cancers	LM.Cannabis	-0.637	0.204	1.90E-03	3.19E+01	-
Prostate	THC.Resin	-2.389	0.262	2.71E-19	3.28E+01	-
All Cancers nNMSC	LM.Cannabis	-0.479	0.041	4.31E-30	3.61E+01	-
Ovary	THC.Resin	-2.029	0.209	1.20E-21	4.07E+01	-
Lung	THC.Herb	-0.983	1.949	6.14E-01	5.18E+01	-
Oropharynx	LM.Cannabis	-1.004	0.688	1.47E-01	6.28E+01	-
Cervix	THC.Resin	-2.905	0.258	2.64E-28	6.64E+01	-
Leukaemia - Myeloid	LM.Cannabis	-1.025	0.601	9.02E-02	1.20E+02	-
All Cancers	THC.Resin	-0.956	0.111	7.52E-17	1.30E+02	-
Ovary	LM.Cannabis:THC.Herb	-2.828	1.874	1.32E-01	1.35E+02	-

Non-Hodgkins lymphoma	LM.Cannabis:THC.Herb	-2.199	1.420	1.22E-01	1.62E+02	-
Melanoma	LM.Cannabis:THC.Herb	-2.220	1.365	1.04E-01	1.92E+02	-
Kidney	THC.Resin	-1.965	0.133	2.28E-46	2.01E+02	-
Anus	THC.Resin	-1.395	0.105	1.29E-37	2.08E+02	-
All Cancers nNMSC	THC.Resin	-0.788	0.072	5.17E-27	2.38E+02	-
Leukaemia - Lymphoid	LM.Cannabis	-0.699	0.334	3.82E-02	3.05E+02	-
Myeloma	LM.Cannabis:THC.Herb	-1.345	0.750	7.34E-02	3.38E+02	-
Testis	LM.Cannabis:THC.Herb	-4.364	2.413	7.08E-02	3.54E+02	-
Pancreas	THC.Resin	-2.207	0.130	6.68E-59	3.91E+02	-
Larynx	LM.Cannabis:THC.Herb	-2.452	1.298	5.91E-02	3.91E+02	-
Brain	LM.Cannabis:THC.Herb	-2.508	1.199	3.66E-02	6.90E+02	-
Leukaemia - Myeloid	THC.Resin	-1.504	0.714	3.67E-02	8.20E+02	-
Thyroid	THC.Resin	-2.765	0.144	3.01E-71	1.47E+03	-
Hodgkins	LM.Cannabis:THC.Herb	-1.914	0.816	1.93E-02	1.64E+03	-
Bladder	LM.Cannabis:THC.Herb	-2.900	1.120	9.73E-03	2.77E+03	-
Gallbladder & Biliary	THC.Resin	-2.528	0.151	5.67E-56	2.92E+03	-
Pancreas	LM.Cannabis:THC.Herb	-3.157	1.169	6.98E-03	3.79E+03	-
Corpus Uteri	LM.Cannabis:THC.Herb	-6.231	2.274	6.22E-03	4.06E+03	-
Kidney	LM.Cannabis:THC.Herb	-3.292	1.190	5.74E-03	4.55E+03	-
Oesophagus	LM.Cannabis:THC.Herb	-4.163	1.393	2.84E-03	8.48E+03	-
Prostate	LM.Cannabis:THC.Herb	-7.847	2.362	9.16E-04	2.05E+04	-
Thyroid	LM.Cannabis:THC.Herb	-4.614	1.199	1.26E-04	1.21E+05	-
Liver	THC.Resin	-4.908	0.155	6.74E-156	1.31E+05	-
Non-Hodgkins lymphoma	THC.Herb	-6.009	3.250	6.46E-02	3.33E+05	-
Anus	LM.Cannabis:THC.Herb	-3.630	0.876	3.68E-05	3.55E+05	-
Breast	LM.Cannabis:THC.Herb	-8.263	1.624	4.08E-07	2.88E+06	-
Stomach	LM.Cannabis:THC.Herb	-11.388	1.834	6.93E-10	6.84E+07	-
Colorectum	LM.Cannabis:THC.Herb	-15.361	2.183	3.01E-12	6.25E+08	-
Oropharynx_Broad	LM.Cannabis:THC.Herb	-8.620	6.233	1.67E-01	4.27E+09	-
Gallbladder & Biliary	LM.Cannabis:THC.Herb	-7.970	1.063	1.40E-13	1.89E+10	-

Kaposi	Tobacco:LM.Cannabis	-7.744	12.776	5.47E-01	4.49E+15	-
Oropharynx	THC.Herb	-11.954	7.420	1.10E-01	1.47E+18	-
All Cancers nNMSC	THC.Herb	-11.441	1.865	1.14E-09	2.88E+30	-
Kaposi	THC.Herb	-2685.723	5511.062	6.28E-01	Inf	-
Kaposi	THC.Resin	-447.119	537.032	4.08E-01	Inf	-

Supplementary Table S28.: Complete Model Output from

Interactive Panel Regression at Two Temporal Lags

Cancer	Term	ß-Estimate	Std. Error	P-Value	P.Adj.Holm	E-Value Estimate	95% Lower Bound of the E-Value
Colorectum	Herb.THC	71.633	6.427	1.57E-27	4.30E-25	1.51E+38	3.40E+31
Breast	Herb.THC	46.987	4.896	4.60E-21	1.21E-18	9.34E+32	2.05E+26
Gallbladder & Biliary	Herb.THC	23.007	2.984	3.78E-14	9.34E-12	1.44E+32	1.19E+24
Anus	Herb.THC	20.215	2.753	4.83E-13	1.16E-10	4.88E+28	1.34E+21
Thyroid	Herb.THC	24.546	3.529	6.92E-12	1.59E-09	1.56E+27	4.27E+19
All Cancers	Herb.THC	13.291	2.291	1.17E-08	2.42E-06	1.44E+26	2.75E+17
Oropharynx	Resin.THC	9.983	1.206	4.86E-13	1.16E-10	5.94E+21	4.99E+16
Oropharynx_Broad	Herb.THC	27.243	6.396	2.56E-05	0.0045042	1.58E+29	8.46E+15
Testis	Herb.THC	39.967	8.119	1.02E-06	1.98E-04	2.14E+19	5.87E+11
Oesophagus	Herb.THC	20.767	3.903	1.23E-07	2.46E-05	2.47E+18	5.52E+11
Stomach	Herb.THC	25.533	5.249	1.30E-06	2.45E-04	6.92E+16	1.55E+10
Myeloma	Herb.THC	7.103	2.279	1.89E-03	0.2677484	2.25E+12	6.17E+04
Melanoma	Herb.THC	12.545	3.908	1.36E-03	0.1974942	1.72E+11	3.74E+04
Prostate	Herb.THC	21.614	7.110	2.42E-03	0.3337366	4.28E+10	9.64E+03
Corpus Uteri	Herb.THC	19.252	6.845	4.99E-03	0.6491038	7.24E+09	1.63E+03
Oropharynx	LM.Cannabis:Herb.THC	18.029	8.556	3.75E-02	1	1.20E+39	1.21E+03
Liver	Herb.THC	9.628	3.773	1.09E-02	1	3.27E+10	4.84E+02
Oropharynx	Income	1.334	0.259	1.21E-06	2.30E-04	1.48E+03	1.20E+02
Oropharynx	Tobacco:Herb.THC	1.171	0.183	4.80E-09	1.01E-06	6.59E+02	1.11E+02
Kidney	Herb.THC	7.611	3.189	1.71E-02	1	2.61E+08	5.79E+01
Oesophagus	Resin.THC	2.014	0.174	2.48E-29	6.84E-27	1.13E+02	5.68E+01

Hodgkins	Herb.THC	5.964	2.585	2.13E-02	1	1.66E+09	4.50E+01
All Cancers nNMSC	Tobacco:Herb.THC	0.550	0.072	3.83E-14	9.42E-12	6.84E+01	2.74E+01
Myeloma	Resin.THC	0.701	0.100	5.36E-12	1.24E-09	3.04E+01	1.39E+01
Colorectum	LM.Cannabis	1.849	0.144	1.17E-35	3.34E-33	1.85E+01	1.30E+01
Stomach	LM.Cannabis	1.415	0.117	1.01E-31	2.83E-29	1.60E+01	1.12E+01
Larynx	Resin.THC	1.146	0.165	5.97E-12	1.38E-09	2.22E+01	1.09E+01
Bladder	Herb.THC	6.672	3.094	3.12E-02	1	4.30E+07	9.07E+00
Thyroid	LM.Cannabis	0.650	0.074	9.63E-18	2.46E-15	9.78E+00	6.61E+00
Larynx	LM.Cannabis	0.756	0.083	2.19E-19	5.72E-17	9.40E+00	6.51E+00
Breast	LM.Cannabis	0.834	0.110	5.40E-14	1.31E-11	7.07E+00	4.84E+00
Hodgkins	LM.Cannabis	0.335	0.054	1.12E-09	2.41E-07	5.78E+00	3.81E+00
Leukaemia - Lymphoid	Alcohol	0.138	0.026	6.53E-07	1.27E-04	6.29E+00	3.73E+00
Colorectum	Resin.THC	1.177	0.285	3.88E-05	0.0066822	7.85E+00	3.67E+00
Leukaemia - Myeloid	Alcohol	0.262	0.053	2.23E-06	4.13E-04	5.85E+00	3.45E+00
Colorectum	Tobacco:LM.Cannabis:Herb.THC	0.726	0.088	3.53E-16	8.87E-14	4.27E+00	3.34E+00
Bladder	Resin.THC	0.515	0.138	2.03E-04	0.0328647	6.83E+00	3.12E+00
Gallbladder & Biliary	Tobacco:LM.Cannabis:Herb.THC	0.265	0.037	1.35E-12	3.17E-10	4.09E+00	3.10E+00
Thyroid	Tobacco:LM.Cannabis:Herb.THC	0.319	0.044	8.30E-13	1.96E-10	3.90E+00	3.00E+00
Anus	Tobacco:LM.Cannabis:Herb.THC	0.213	0.034	8.03E-10	1.75E-07	3.39E+00	2.59E+00
Corpus Uteri	LM.Cannabis	0.666	0.153	1.43E-05	0.00254	3.71E+00	2.41E+00
Hodgkins	Resin.THC	0.343	0.114	2.61E-03	0.3503521	5.98E+00	2.40E+00
Breast	Tobacco:LM.Cannabis:Herb.THC	0.381	0.067	1.96E-08	4.02E-06	3.08E+00	2.34E+00
Lung	Resin.THC	0.301	0.100	2.59E-03	0.3494918	5.15E+00	2.25E+00
Testis	Income	0.522	0.107	1.21E-06	2.30E-04	2.94E+00	2.17E+00
All Cancers	Tobacco:LM.Cannabis:Herb.THC	0.215	0.072	2.89E-03	0.3837699	4.69E+00	2.14E+00
Stomach	Tobacco:LM.Cannabis:Herb.THC	0.362	0.072	5.39E-07	1.06E-04	2.82E+00	2.13E+00
Oropharynx	Tobacco	0.111	0.027	6.00E-05	0.0101373	2.86E+00	2.02E+00
Prostate	LM.Cannabis	0.554	0.159	5.01E-04	0.0760975	3.08E+00	1.94E+00
Gallbladder & Biliary	Tobacco	0.093	0.006	1.26E-45	3.66E-43	2.02E+00	1.91E+00
Gallbladder & Biliary	LM.Cannabis	0.200	0.062	1.30E-03	0.1894048	3.19E+00	1.89E+00

All Cancers	Alcohol	0.072	0.011	6.55E-10	1.45E-07	2.11E+00	1.81E+00
Testis	LM.Cannabis	0.533	0.171	1.84E-03	0.2633628	2.99E+00	1.80E+00
Hodgkins	Tobacco:LM.Cannabis:Herb.THC	0.125	0.032	1.04E-04	0.0172133	2.45E+00	1.79E+00
Larynx	Alcohol	0.121	0.012	1.24E-23	3.33E-21	1.91E+00	1.76E+00
Kidney	Tobacco:LM.Cannabis:Herb.THC	0.162	0.044	2.13E-04	0.0340732	2.34E+00	1.71E+00
Brain	Income	0.148	0.033	1.09E-05	0.0019595	2.15E+00	1.70E+00
Myeloma	Tobacco:LM.Cannabis:Herb.THC	0.103	0.028	2.89E-04	0.0450391	2.36E+00	1.70E+00
Oesophagus	Tobacco:LM.Cannabis:Herb.THC	0.195	0.053	2.68E-04	0.0424001	2.32E+00	1.69E+00
Breast	Income	0.200	0.048	2.98E-05	0.0051635	2.10E+00	1.66E+00
Liver	Tobacco:LM.Cannabis:Herb.THC	0.157	0.046	6.92E-04	0.1038684	2.29E+00	1.63E+00
Non-Hodgkins lymphoma	Tobacco:Herb.THC	0.344	0.132	9.62E-03	1	3.27E+00	1.63E+00
Oropharynx	Tobacco:LM.Cannabis	0.086	0.028	2.24E-03	0.3107643	2.44E+00	1.63E+00
Myeloma	LM.Cannabis	0.135	0.048	5.05E-03	0.6516111	2.77E+00	1.62E+00
Prostate	Income	0.279	0.069	5.83E-05	0.0099148	2.06E+00	1.62E+00
Colorectum	Tobacco	0.119	0.009	2.97E-37	8.49E-35	1.58E+00	1.52E+00
Breast	Tobacco	0.088	0.007	3.68E-35	1.04E-32	1.57E+00	1.50E+00
Prostate	Tobacco:LM.Cannabis:Herb.THC	0.281	0.097	3.90E-03	0.5114543	2.06E+00	1.45E+00
Stomach	Tobacco	0.081	0.007	5.69E-27	1.55E-24	1.51E+00	1.45E+00
Colorectum	Alcohol	0.120	0.020	5.07E-09	1.06E-06	1.58E+00	1.44E+00
All Cancers nNMSC	Tobacco:LM.Cannabis	0.017	0.002	6.89E-24	1.86E-21	1.47E+00	1.40E+00
Testis	Tobacco:LM.Cannabis:Herb.THC	0.264	0.101	9.25E-03	1	2.00E+00	1.36E+00
Pancreas	Tobacco:LM.Cannabis:Herb.THC	0.112	0.043	9.81E-03	1	1.97E+00	1.34E+00
Corpus Uteri	Tobacco	0.073	0.010	4.44E-14	1.09E-11	1.40E+00	1.33E+00
Oesophagus	Alcohol	0.055	0.012	1.12E-05	0.0020028	1.48E+00	1.32E+00
Testis	Tobacco	0.075	0.012	3.13E-09	6.64E-07	1.39E+00	1.30E+00
Prostate	Tobacco	0.069	0.010	8.15E-12	1.87E-09	1.37E+00	1.30E+00
Stomach	Alcohol	0.067	0.017	7.62E-05	0.0128029	1.44E+00	1.28E+00
Ovary	Tobacco	0.052	0.008	1.06E-10	2.36E-08	1.36E+00	1.28E+00
Liver	Tobacco:LM.Cannabis	0.016	0.002	2.54E-11	5.74E-09	1.25E+00	1.20E+00
Breast	Alcohol	0.048	0.016	2.14E-03	0.2999347	1.37E+00	1.20E+00

Melanoma	Tobacco:LM.Cannabis	0.019	0.003	4.89E-11	1.10E-08	1.24E+00	1.19E+00
Lung	Tobacco:LM.Cannabis	0.011	0.002	4.76E-11	1.07E-08	1.24E+00	1.19E+00
Bladder	LM.Cannabis	0.145	0.069	3.63E-02	1	2.24E+00	1.18E+00
Pancreas	Tobacco:LM.Cannabis	0.014	0.002	9.55E-10	2.07E-07	1.23E+00	1.18E+00
Larynx	Tobacco	0.019	0.005	2.06E-04	0.0331831	1.25E+00	1.16E+00
Cervix	Alcohol	0.060	0.022	6.45E-03	0.8197789	1.35E+00	1.16E+00
Kidney	Tobacco:LM.Cannabis	0.012	0.002	5.20E-07	1.02E-04	1.20E+00	1.15E+00
Prostate	Alcohol	0.058	0.023	9.90E-03	1	1.33E+00	1.14E+00
Oesophagus	Tobacco:LM.Cannabis	0.013	0.003	7.85E-06	0.0014205	1.19E+00	1.13E+00
Non-Hodgkins lymphoma	Resin.THC	0.374	0.188	4.71E-02	1	3.50E+00	1.12E+00
Bladder	Tobacco	0.012	0.004	6.51E-03	0.8204035	1.21E+00	1.10E+00
Non-Hodgkins lymphoma	Tobacco:LM.Cannabis	0.010	0.003	7.32E-04	0.1091011	1.16E+00	1.10E+00
Thyroid	Alcohol	0.027	0.013	3.21E-02	1	1.34E+00	1.08E+00
Brain	Tobacco	0.011	0.005	2.49E-02	1	1.19E+00	1.06E+00
Anus	Tobacco:LM.Cannabis	0.004	0.002	2.71E-02	1	1.13E+00	1.04E+00
Brain	Tobacco:LM.Cannabis	0.000	0.002	9.17E-01	1	1.02E+00	1.00E+00
Cervix	Tobacco:LM.Cannabis	0.003	0.005	5.88E-01	1	1.06E+00	1.00E+00
Corpus Uteri	Alcohol	0.007	0.022	7.58E-01	1	1.10E+00	1.00E+00
Cervix	Tobacco	0.011	0.010	2.79E-01	1	1.12E+00	1.00E+00
Testis	Alcohol	0.024	0.029	4.01E-01	1	1.19E+00	1.00E+00
Hodgkins	Alcohol	0.010	0.009	2.95E-01	1	1.22E+00	1.00E+00
Melanoma	Income	0.024	0.038	5.27E-01	1	1.28E+00	1.00E+00
Ovary	Tobacco:LM.Cannabis:Herb.THC	0.064	0.077	4.07E-01	1	1.41E+00	1.00E+00
Ovary	Tobacco:Herb.THC	0.081	0.181	6.52E-01	1	1.49E+00	1.00E+00
Brain	Tobacco:LM.Cannabis:Herb.THC	0.055	0.047	2.40E-01	1	1.52E+00	1.00E+00
Cervix	Tobacco:Herb.THC	0.113	0.222	6.09E-01	1	1.53E+00	1.00E+00
Pancreas	Tobacco:Herb.THC	0.054	0.102	5.99E-01	1	1.54E+00	1.00E+00
Oropharynx_Broad	Income	0.058	0.057	3.07E-01	1	1.57E+00	1.00E+00
Non-Hodgkins lymphoma	Tobacco:LM.Cannabis:Herb.THC	0.081	0.058	1.64E-01	1	1.61E+00	1.00E+00
Oropharynx_Broad	Tobacco	0.064	0.033	5.49E-02	1	1.61E+00	1.00E+00

Bladder	Tobacco:LM.Cannabis:Herb.THC	0.062	0.042	1.46E-01	1	1.61E+00	1.00E+00
Lung	Tobacco:LM.Cannabis:Herb.THC	0.046	0.031	1.33E-01	1	1.63E+00	1.00E+00
Larynx	Tobacco:LM.Cannabis:Herb.THC	0.086	0.050	8.89E-02	1	1.69E+00	1.00E+00
Corpus Uteri	Tobacco:LM.Cannabis:Herb.THC	0.182	0.093	5.16E-02	1	1.77E+00	1.00E+00
Melanoma	Tobacco:LM.Cannabis:Herb.THC	0.104	0.054	5.43E-02	1	1.77E+00	1.00E+00
Myeloma	Income	0.054	0.030	7.06E-02	1	1.78E+00	1.00E+00
Brain	Tobacco:Herb.THC	0.132	0.110	2.33E-01	1	2.04E+00	1.00E+00
Lung	Tobacco:Herb.THC	0.089	0.072	2.17E-01	1	2.07E+00	1.00E+00
Ovary	LM.Cannabis	0.229	0.126	6.91E-02	1	2.09E+00	1.00E+00
Leukaemia - Myeloid	Tobacco:LM.Cannabis:Herb.THC	0.076	0.233	7.46E-01	1	2.14E+00	1.00E+00
Kaposi	Income	0.132	0.765	8.64E-01	1	3.41E+00	1.00E+00
Cervix	Herb.THC	0.972	6.916	8.88E-01	1	5.46E+00	1.00E+00
Leukaemia - Lymphoid	Tobacco:LM.Cannabis:Herb.THC	0.144	0.116	2.17E-01	1	6.67E+00	1.00E+00
Leukaemia - Myeloid	LM.Cannabis	0.348	0.885	6.95E-01	1	8.85E+00	1.00E+00
Oropharynx_Broad	Tobacco:LM.Cannabis:Herb.THC	0.671	0.520	1.97E-01	1	9.78E+00	1.00E+00
Cervix	LM.Cannabis:Herb.THC	1.628	2.958	5.82E-01	1	1.21E+01	1.00E+00
All Cancers nNMSC	LM.Cannabis:Herb.THC	0.723	0.785	3.57E-01	1	2.10E+02	1.00E+00
Oropharynx_Broad	LM.Cannabis	2.141	1.888	2.57E-01	1	3.73E+02	1.00E+00
Ovary	Herb.THC	3.972	5.634	4.81E-01	1	4.99E+02	1.00E+00
Leukaemia - Lymphoid	LM.Cannabis	0.681	0.442	1.25E-01	1	8.56E+02	1.00E+00
Lung	Herb.THC	2.054	2.246	3.61E-01	1	2.57E+03	1.00E+00
Larynx	Herb.THC	6.218	3.692	9.25E-02	1	1.06E+06	1.00E+00
Pancreas	Herb.THC	6.037	3.177	5.77E-02	1	5.77E+06	1.00E+00
Kaposi	Tobacco:LM.Cannabis	5.973	23.657	8.02E-01	1	8.03E+13	1.00E+00
Leukaemia - Lymphoid	Herb.THC	4.180	3.411	2.23E-01	1	2.78E+16	1.00E+00
Leukaemia - Myeloid	Herb.THC	9.537	6.836	1.65E-01	1	4.82E+18	1.00E+00
Kaposi	Herb.THC	3112.129	9432.047	7.43E-01	1	Inf	1.00E+00
Thyroid	Tobacco	-0.001	0.005	8.94E-01	1	1.04E+00	-
Bladder	Tobacco:LM.Cannabis	-0.003	0.002	1.51E-01	1	1.10E+00	-
Myeloma	Tobacco:LM.Cannabis	-0.002	0.002	1.14E-01	1	1.11E+00	-

Ovary	Tobacco:LM.Cannabis	-0.008	0.004	4.53E-02	1	1.12E+00	-
Hodgkins	Tobacco	-0.004	0.004	2.63E-01	1	1.14E+00	-
All Cancers	Tobacco:LM.Cannabis	-0.004	0.008	6.41E-01	1	1.15E+00	-
Anus	Tobacco	-0.005	0.004	2.23E-01	1	1.15E+00	-
Prostate	Tobacco:LM.Cannabis	-0.017	0.005	7.87E-04	0.1164532	1.16E+00	-
Testis	Tobacco:LM.Cannabis	-0.018	0.005	7.97E-04	0.1171623	1.16E+00	-
Corpus Uteri	Tobacco:LM.Cannabis	-0.021	0.005	2.72E-05	0.0047296	1.18E+00	-
Corpus Uteri	Income	-0.023	0.067	7.25E-01	1	1.19E+00	-
Kidney	Tobacco	-0.012	0.004	6.67E-03	0.8333041	1.21E+00	-
Hodgkins	Tobacco:LM.Cannabis	-0.009	0.002	8.55E-08	1.72E-05	1.22E+00	-
All Cancers	Tobacco	-0.007	0.003	3.83E-02	1	1.22E+00	-
Gallbladder & Biliary	Tobacco:LM.Cannabis	-0.012	0.002	1.76E-09	3.77E-07	1.24E+00	-
Oesophagus	Tobacco	-0.019	0.005	4.87E-04	0.0745743	1.24E+00	-
Cervix	Tobacco:LM.Cannabis:Herb.THC	-0.034	0.095	7.16E-01	1	1.24E+00	-
Larynx	Tobacco:LM.Cannabis	-0.019	0.003	5.24E-13	1.24E-10	1.25E+00	-
Breast	Tobacco:LM.Cannabis	-0.026	0.004	4.10E-13	9.85E-11	1.25E+00	-
Colorectum	Income	-0.034	0.062	5.81E-01	1	1.25E+00	-
Lung	Tobacco	-0.012	0.003	1.06E-04	0.0174307	1.26E+00	-
Pancreas	Tobacco	-0.018	0.004	7.91E-05	0.0132035	1.26E+00	-
Ovary	Alcohol	-0.032	0.018	7.28E-02	1	1.27E+00	-
Myeloma	Alcohol	-0.012	0.008	1.52E-01	1	1.27E+00	-
Ovary	Income	-0.034	0.055	5.36E-01	1	1.27E+00	-
Melanoma	Tobacco	-0.024	0.005	1.45E-05	0.0025677	1.28E+00	-
Thyroid	Tobacco:LM.Cannabis	-0.020	0.002	2.44E-16	6.19E-14	1.28E+00	-
Kidney	Tobacco:Herb.THC	-0.021	0.102	8.41E-01	1	1.29E+00	-
Myeloma	Tobacco	-0.015	0.003	2.68E-05	0.0046903	1.31E+00	-
Stomach	Tobacco:LM.Cannabis	-0.043	0.004	1.27E-28	3.49E-26	1.33E+00	-
Colorectum	Tobacco:LM.Cannabis	-0.054	0.005	2.99E-30	8.30E-28	1.34E+00	-
All Cancers nNMSC	Tobacco:LM.Cannabis:Herb.THC	-0.011	0.025	6.63E-01	1	1.35E+00	-
Leukaemia - Myeloid	Tobacco:LM.Cannabis	-0.017	0.026	5.04E-01	1	1.37E+00	-

Hodgkins	Income	-0.025	0.034	4.53E-01	1	1.41E+00	-
Leukaemia - Myeloid	Tobacco	-0.021	0.025	4.02E-01	1	1.43E+00	-
Non-Hodgkins lymphoma	Tobacco	-0.051	0.006	4.97E-18	1.28E-15	1.44E+00	-
All Cancers	LM.Cannabis	-0.024	0.260	9.27E-01	1	1.47E+00	-
Liver	Tobacco	-0.045	0.006	6.10E-15	1.52E-12	1.48E+00	-
Brain	Alcohol	-0.053	0.011	1.85E-06	3.46E-04	1.51E+00	-
Brain	LM.Cannabis	-0.054	0.077	4.82E-01	1	1.52E+00	-
Oropharynx_Broad	Alcohol	-0.051	0.071	4.76E-01	1	1.52E+00	-
Non-Hodgkins lymphoma	Alcohol	-0.076	0.013	9.75E-09	2.03E-06	1.58E+00	-
Thyroid	Income	-0.059	0.046	2.02E-01	1	1.59E+00	-
Gallbladder & Biliary	Income	-0.049	0.038	1.95E-01	1	1.62E+00	-
Oropharynx_Broad	Tobacco:LM.Cannabis	-0.065	0.058	2.67E-01	1	1.62E+00	-
Lung	Alcohol	-0.046	0.007	1.07E-10	2.38E-08	1.63E+00	-
Anus	Alcohol	-0.052	0.010	1.58E-07	3.15E-05	1.65E+00	-
Oropharynx	Alcohol	-0.034	0.057	5.58E-01	1	1.65E+00	-
Leukaemia - Lymphoid	Tobacco:LM.Cannabis	-0.022	0.013	9.04E-02	1	1.73E+00	-
Leukaemia - Lymphoid	Tobacco	-0.022	0.012	7.77E-02	1	1.73E+00	-
Melanoma	Alcohol	-0.103	0.012	3.32E-16	8.41E-14	1.76E+00	-
Cervix	LM.Cannabis	-0.190	0.155	2.20E-01	1	1.78E+00	-
Bladder	Tobacco:Herb.THC	-0.091	0.099	3.61E-01	1	1.83E+00	-
Kidney	Alcohol	-0.103	0.010	6.26E-23	1.67E-20	1.89E+00	-
Larynx	Tobacco:Herb.THC	-0.120	0.118	3.10E-01	1	1.90E+00	-
Cervix	Income	-0.240	0.067	3.76E-04	0.0582714	1.95E+00	-
All Cancers nNMSC	Tobacco	-0.043	0.004	1.17E-23	3.15E-21	1.97E+00	-
Bladder	Alcohol	-0.115	0.010	6.49E-30	1.80E-27	2.01E+00	-
Pancreas	Alcohol	-0.125	0.010	4.90E-33	1.38E-30	2.06E+00	-
Larynx	Income	-0.175	0.036	1.14E-06	2.17E-04	2.26E+00	-
Oesophagus	Income	-0.186	0.038	1.10E-06	2.12E-04	2.26E+00	-
Liver	Tobacco:Herb.THC	-0.161	0.121	1.82E-01	1	2.33E+00	-
Liver	Alcohol	-0.162	0.013	6.78E-33	1.90E-30	2.33E+00	-

Melanoma	Resin.THC	-0.205	0.176	2.44E-01	1	2.38E+00	-
Breast	Resin.THC	-0.259	0.218	2.36E-01	1	2.39E+00	-
Anus	Income	-0.132	0.036	2.77E-04	0.0434285	2.44E+00	-
Corpus Uteri	Tobacco:Herb.THC	-0.392	0.219	7.40E-02	1	2.51E+00	-
Prostate	Tobacco:Herb.THC	-0.431	0.228	5.89E-02	1	2.59E+00	-
Leukaemia - Myeloid	Income	-0.111	0.232	6.33E-01	1	2.66E+00	-
Myeloma	Tobacco:Herb.THC	-0.128	0.074	8.32E-02	1	2.68E+00	-
Gallbladder & Biliary	Alcohol	-0.163	0.012	2.60E-39	7.47E-37	2.75E+00	-
Bladder	Income	-0.206	0.030	1.19E-11	2.71E-09	2.76E+00	-
Anus	LM.Cannabis	-0.162	0.058	5.28E-03	0.6757845	2.76E+00	-
Liver	Income	-0.219	0.048	5.72E-06	0.0010408	2.81E+00	-
Non-Hodgkins lymphoma	Income	-0.307	0.040	5.31E-14	1.30E-11	3.00E+00	-
Hodgkins	Tobacco:Herb.THC	-0.174	0.083	3.78E-02	1	3.04E+00	-
All Cancers nNMSC	Alcohol	-0.094	0.005	3.77E-60	1.11E-57	3.07E+00	-
Non-Hodgkins lymphoma	LM.Cannabis	-0.324	0.093	5.14E-04	0.077616	3.12E+00	-
Melanoma	Tobacco:Herb.THC	-0.336	0.125	7.41E-03	0.9188925	3.34E+00	-
Stomach	Income	-0.491	0.051	3.43E-21	9.08E-19	3.58E+00	-
Stomach	Tobacco:Herb.THC	-0.511	0.168	2.42E-03	0.3337366	3.71E+00	-
Oesophagus	LM.Cannabis	-0.410	0.087	2.83E-06	5.20E-04	3.98E+00	-
All Cancers	Income	-0.203	0.027	1.05E-13	2.53E-11	4.40E+00	-
Kidney	LM.Cannabis	-0.441	0.071	8.00E-10	1.75E-07	5.36E+00	-
Pancreas	Income	-0.454	0.031	4.04E-45	1.17E-42	5.57E+00	-
Liver	LM.Cannabis	-0.464	0.077	2.58E-09	5.49E-07	5.66E+00	-
Oesophagus	Tobacco:Herb.THC	-0.571	0.125	5.55E-06	0.001016	5.74E+00	-
Leukaemia - Myeloid	LM.Cannabis:Herb.THC	-0.265	7.868	9.73E-01	1	5.94E+00	-
Lung	LM.Cannabis	-0.351	0.050	4.33E-12	1.01E-09	6.25E+00	-
Pancreas	LM.Cannabis	-0.498	0.071	3.82E-12	8.94E-10	6.28E+00	-
Lung	Income	-0.356	0.022	3.05E-54	8.91E-52	6.37E+00	-
Kidney	Income	-0.507	0.031	1.54E-54	4.53E-52	6.40E+00	-
Testis	Tobacco:Herb.THC	-1.253	0.262	2.04E-06	3.80E-04	7.36E+00	-

All Cancers	Tobacco:Herb.THC	-0.320	0.078	4.39E-05	0.0075147	7.85E+00	-
Melanoma	LM.Cannabis	-0.725	0.088	3.33E-16	8.41E-14	8.04E+00	-
Testis	Resin.THC	-1.334	0.357	2.01E-04	0.0327814	8.10E+00	-
Leukaemia - Myeloid	Tobacco:Herb.THC	-0.379	0.187	4.48E-02	1	1.02E+01	-
Leukaemia - Myeloid	Resin.THC	-0.390	0.963	6.86E-01	1	1.08E+01	-
Stomach	Resin.THC	-1.164	0.235	7.95E-07	1.54E-04	1.08E+01	-
Anus	Tobacco:Herb.THC	-0.553	0.089	7.57E-10	1.67E-07	1.14E+01	-
Leukaemia - Lymphoid	Tobacco:Herb.THC	-0.203	0.093	3.14E-02	1	1.17E+01	-
Oropharynx_Broad	Tobacco:Herb.THC	-0.811	0.261	2.05E-03	0.2897444	1.40E+01	-
Leukaemia - Lymphoid	Income	-0.238	0.116	4.14E-02	1	1.62E+01	-
Thyroid	Tobacco:Herb.THC	-0.844	0.114	3.00E-13	7.22E-11	1.63E+01	-
Corpus Uteri	Resin.THC	-1.864	0.304	1.13E-09	2.43E-07	1.63E+01	-
Breast	Tobacco:Herb.THC	-1.405	0.157	1.36E-18	3.55E-16	1.85E+01	-
All Cancers nNMSC	Income	-0.367	0.017	1.37E-86	4.07E-84	2.07E+01	-
Colorectum	Tobacco:Herb.THC	-2.145	0.206	2.33E-24	6.33E-22	2.67E+01	-
Prostate	Resin.THC	-2.458	0.315	1.43E-14	3.54E-12	2.94E+01	-
Brain	Resin.THC	-1.302	0.154	7.79E-17	1.99E-14	3.80E+01	-
Ovary	Resin.THC	-2.215	0.252	4.58E-18	1.18E-15	4.30E+01	-
Gallbladder & Biliary	Tobacco:Herb.THC	-0.995	0.100	5.55E-22	1.48E-19	4.72E+01	-
Ovary	LM.Cannabis:Herb.THC	-2.359	2.409	3.28E-01	1	5.26E+01	-
Cervix	Resin.THC	-2.911	0.309	2.21E-20	5.82E-18	5.35E+01	-
Brain	LM.Cannabis:Herb.THC	-1.479	1.473	3.16E-01	1	5.72E+01	-
All Cancers nNMSC	LM.Cannabis	-0.556	0.053	7.68E-25	2.09E-22	7.09E+01	-
Melanoma	LM.Cannabis:Herb.THC	-1.865	1.681	2.68E-01	1	8.39E+01	-
Oropharynx_Broad	Resin.THC	-1.594	1.046	1.28E-01	1	9.76E+01	-
Oropharynx	Tobacco:LM.Cannabis:Herb.THC	-0.792	0.256	2.52E-03	0.3429089	1.00E+02	-
Lung	LM.Cannabis:Herb.THC	-1.200	0.959	2.11E-01	1	1.30E+02	-
Non-Hodgkins lymphoma	LM.Cannabis:Herb.THC	-2.287	1.801	2.04E-01	1	1.58E+02	-
Kidney	Resin.THC	-1.820	0.142	3.75E-35	1.06E-32	1.74E+02	-
All Cancers nNMSC	Resin.THC	-0.707	0.087	1.68E-15	4.20E-13	1.88E+02	-

All Cancers	Resin.THC	-1.041	0.110	8.80E-20	2.31E-17	2.12E+02	-
Anus	Resin.THC	-1.447	0.121	1.52E-30	4.23E-28	2.15E+02	-
Pancreas	Resin.THC	-2.022	0.142	1.10E-42	3.17E-40	2.92E+02	-
Leukaemia - Lymphoid	Resin.THC	-0.650	0.480	1.79E-01	1	6.45E+02	-
Thyroid	Resin.THC	-2.501	0.155	3.14E-51	9.14E-49	1.10E+03	-
Brain	Herb.THC	-3.023	3.445	3.80E-01	1	1.92E+03	-
Corpus Uteri	LM.Cannabis:Herb.THC	-6.351	2.921	2.99E-02	1	2.85E+03	-
Pancreas	LM.Cannabis:Herb.THC	-3.114	1.359	2.21E-02	1	4.30E+03	-
Larynx	LM.Cannabis:Herb.THC	-3.793	1.579	1.65E-02	1	6.22E+03	-
Testis	LM.Cannabis:Herb.THC	-7.897	3.197	1.37E-02	1	1.15E+04	-
Bladder	LM.Cannabis:Herb.THC	-3.528	1.323	7.77E-03	0.9560667	1.51E+04	-
Gallbladder & Biliary	Resin.THC	-2.889	0.169	3.31E-56	9.73E-54	2.00E+04	-
Liver	LM.Cannabis:Herb.THC	-3.856	1.460	8.40E-03	1	2.46E+04	-
Prostate	LM.Cannabis:Herb.THC	-8.929	3.035	3.32E-03	0.4381633	3.71E+04	-
Oropharynx	LM.Cannabis	-2.078	0.944	2.99E-02	1	5.88E+04	-
Kidney	LM.Cannabis:Herb.THC	-4.294	1.364	1.68E-03	0.2415374	7.60E+04	-
Liver	Resin.THC	-4.608	0.165	2.33E-122	6.93E-120	1.55E+05	-
Oesophagus	LM.Cannabis:Herb.THC	-5.896	1.669	4.27E-04	0.0658085	2.74E+05	-
Hodgkins	LM.Cannabis:Herb.THC	-3.753	1.018	2.41E-04	0.0383064	8.19E+05	-
Non-Hodgkins lymphoma	Herb.THC	-6.856	4.122	9.65E-02	1	9.85E+05	-
Myeloma	LM.Cannabis:Herb.THC	-3.506	0.897	1.00E-04	0.0166784	1.77E+06	-
Breast	LM.Cannabis:Herb.THC	-11.728	2.099	2.87E-08	5.86E-06	2.86E+08	-
Anus	LM.Cannabis:Herb.THC	-5.910	1.084	6.50E-08	1.31E-05	3.98E+08	-
Stomach	LM.Cannabis:Herb.THC	-12.980	2.245	9.39E-09	1.96E-06	5.12E+08	-
Thyroid	LM.Cannabis:Herb.THC	-8.030	1.389	1.04E-08	2.16E-06	1.25E+09	-
Gallbladder & Biliary	LM.Cannabis:Herb.THC	-7.950	1.156	1.23E-11	2.78E-09	2.03E+11	-
All Cancers	LM.Cannabis:Herb.THC	-5.790	2.342	1.38E-02	1	3.67E+11	-
Colorectum	LM.Cannabis:Herb.THC	-24.428	2.743	1.91E-18	4.95E-16	1.65E+13	-
Leukaemia - Lymphoid	LM.Cannabis:Herb.THC	-4.021	3.926	3.08E-01	1	6.78E+15	-
Oropharynx_Broad	LM.Cannabis:Herb.THC	-21.443	16.589	1.97E-01	1	1.11E+23	-

Kaposi	Tobacco:LM.Cannabis:Herb.THC	-13.532	52.173	7.96E-01	1	1.32E+31	-
Kaposi	Tobacco	-14.942	45.896	7.46E-01	1	2.14E+34	-
All Cancers nNMSC	Herb.THC	-12.551	2.267	3.88E-08	7.88E-06	2.37E+35	-
Oropharynx	Herb.THC	-39.139	7.250	4.33E-07	8.58E-05	3.03E+84	-
Kaposi	LM.Cannabis	-44.889	201.931	8.25E-01	1	3.40E+102	-
Kaposi	LM.Cannabis:Herb.THC	-587.141	2205.823	7.91E-01	1	Inf	-

Supplementary Table S29.: Significant Positive Terms from

Interactive Panel Regression at Two Temporal Lags

Cancer	Term	Estimate	Std.Error	P_Value	P.Adj.Holm	eValPt	eValLo
Colorectum	Herb.THC	71.633	6.427	1.57E-27	4.30E-25	1.51E+38	3.40E+31
Breast	Herb.THC	46.987	4.896	4.60E-21	1.21E-18	9.34E+32	2.05E+26
Gallbladder & Biliary	Herb.THC	23.007	2.984	3.78E-14	9.34E-12	1.44E+32	1.19E+24
Anus	Herb.THC	20.215	2.753	4.83E-13	1.16E-10	4.88E+28	1.34E+21
Thyroid	Herb.THC	24.546	3.529	6.92E-12	1.59E-09	1.56E+27	4.27E+19
All Cancers	Herb.THC	13.291	2.291	1.17E-08	2.42E-06	1.44E+26	2.75E+17
Oropharynx	Resin.THC	9.983	1.206	4.86E-13	1.16E-10	5.94E+21	4.99E+16
Oropharynx_Broad	Herb.THC	27.243	6.396	2.56E-05	4.50E-03	1.58E+29	8.46E+15
Testis	Herb.THC	39.967	8.119	1.02E-06	1.98E-04	2.14E+19	5.87E+11
Oesophagus	Herb.THC	20.767	3.903	1.23E-07	2.46E-05	2.47E+18	5.52E+11
Stomach	Herb.THC	25.533	5.249	1.30E-06	2.45E-04	6.92E+16	1.55E+10
Myeloma	Herb.THC	7.103	2.279	1.89E-03	2.68E-01	2.25E+12	6.17E+04
Melanoma	Herb.THC	12.545	3.908	1.36E-03	1.97E-01	1.72E+11	3.74E+04
Prostate	Herb.THC	21.614	7.110	2.42E-03	3.34E-01	4.28E+10	9.64E+03
Corpus Uteri	Herb.THC	19.252	6.845	4.99E-03	6.49E-01	7.24E+09	1.63E+03
Oropharynx	LM.Cannabis:Herb.THC	18.029	8.556	3.75E-02	1.00E+00	1.20E+39	1.21E+03
Liver	Herb.THC	9.628	3.773	1.09E-02	1.00E+00	3.27E+10	4.84E+02
Oropharynx	Income	1.334	0.259	1.21E-06	2.30E-04	1.48E+03	1.20E+02
Oropharynx	Tobacco:Herb.THC	1.171	0.183	4.80E-09	1.01E-06	6.59E+02	1.11E+02
Kidney	Herb.THC	7.611	3.189	1.71E-02	1.00E+00	2.61E+08	5.79E+01
Oesophagus	Resin.THC	2.014	0.174	2.48E-29	6.84E-27	1.13E+02	5.68E+01
Hodgkins	Herb.THC	5.964	2.585	2.13E-02	1.00E+00	1.66E+09	4.50E+01
All Cancers nNMSC	Tobacco:Herb.THC	0.550	0.072	3.83E-14	9.42E-12	6.84E+01	2.74E+01
Myeloma	Resin.THC	0.701	0.100	5.36E-12	1.24E-09	3.04E+01	1.39E+01

Colorectum	LM.Cannabis	1.849	0.144	1.17E-35	3.34E-33	1.85E+01	1.30E+01
Stomach	LM.Cannabis	1.415	0.117	1.01E-31	2.83E-29	1.60E+01	1.12E+01
Larynx	Resin.THC	1.146	0.165	5.97E-12	1.38E-09	2.22E+01	1.09E+01
Bladder	Herb.THC	6.672	3.094	3.12E-02	1.00E+00	4.30E+07	9.07E+00
Thyroid	LM.Cannabis	0.650	0.074	9.63E-18	2.46E-15	9.78E+00	6.61E+00
Larynx	LM.Cannabis	0.756	0.083	2.19E-19	5.72E-17	9.40E+00	6.51E+00
Breast	LM.Cannabis	0.834	0.110	5.40E-14	1.31E-11	7.07E+00	4.84E+00
Hodgkins	LM.Cannabis	0.335	0.054	1.12E-09	2.41E-07	5.78E+00	3.81E+00
Leukaemia - Lymphoid	Alcohol	0.138	0.026	6.53E-07	1.27E-04	6.29E+00	3.73E+00
Colorectum	Resin.THC	1.177	0.285	3.88E-05	6.68E-03	7.85E+00	3.67E+00
Leukaemia - Myeloid	Alcohol	0.262	0.053	2.23E-06	4.13E-04	5.85E+00	3.45E+00
Colorectum	Tobacco:LM.Cannabis:Herb.THC	0.726	0.088	3.53E-16	8.87E-14	4.27E+00	3.34E+00
Bladder	Resin.THC	0.515	0.138	2.03E-04	3.29E-02	6.83E+00	3.12E+00
Gallbladder & Biliary	Tobacco:LM.Cannabis:Herb.THC	0.265	0.037	1.35E-12	3.17E-10	4.09E+00	3.10E+00
Thyroid	Tobacco:LM.Cannabis:Herb.THC	0.319	0.044	8.30E-13	1.96E-10	3.90E+00	3.00E+00
Anus	Tobacco:LM.Cannabis:Herb.THC	0.213	0.034	8.03E-10	1.75E-07	3.39E+00	2.59E+00
Corpus Uteri	LM.Cannabis	0.666	0.153	1.43E-05	2.54E-03	3.71E+00	2.41E+00
Hodgkins	Resin.THC	0.343	0.114	2.61E-03	3.50E-01	5.98E+00	2.40E+00
Breast	Tobacco:LM.Cannabis:Herb.THC	0.381	0.067	1.96E-08	4.02E-06	3.08E+00	2.34E+00
Lung	Resin.THC	0.301	0.100	2.59E-03	3.49E-01	5.15E+00	2.25E+00
Testis	Income	0.522	0.107	1.21E-06	2.30E-04	2.94E+00	2.17E+00
All Cancers	Tobacco:LM.Cannabis:Herb.THC	0.215	0.072	2.89E-03	3.84E-01	4.69E+00	2.14E+00
Stomach	Tobacco:LM.Cannabis:Herb.THC	0.362	0.072	5.39E-07	1.06E-04	2.82E+00	2.13E+00
Oropharynx	Tobacco	0.111	0.027	6.00E-05	1.01E-02	2.86E+00	2.02E+00
Prostate	LM.Cannabis	0.554	0.159	5.01E-04	7.61E-02	3.08E+00	1.94E+00
Gallbladder & Biliary	Tobacco	0.093	0.006	1.26E-45	3.66E-43	2.02E+00	1.91E+00
Gallbladder & Biliary	LM.Cannabis	0.200	0.062	1.30E-03	1.89E-01	3.19E+00	1.89E+00
All Cancers	Alcohol	0.072	0.011	6.55E-10	1.45E-07	2.11E+00	1.81E+00
Testis	LM.Cannabis	0.533	0.171	1.84E-03	2.63E-01	2.99E+00	1.80E+00
Hodgkins	Tobacco:LM.Cannabis:Herb.THC	0.125	0.032	1.04E-04	1.72E-02	2.45E+00	1.79E+00

Larynx	Alcohol	0.121	0.012	1.24E-23	3.33E-21	1.91E+00	1.76E+00
Kidney	Tobacco:LM.Cannabis:Herb.THC	0.162	0.044	2.13E-04	3.41E-02	2.34E+00	1.71E+00
Brain	Income	0.148	0.033	1.09E-05	1.96E-03	2.15E+00	1.70E+00
Myeloma	Tobacco:LM.Cannabis:Herb.THC	0.103	0.028	2.89E-04	4.50E-02	2.36E+00	1.70E+00
Oesophagus	Tobacco:LM.Cannabis:Herb.THC	0.195	0.053	2.68E-04	4.24E-02	2.32E+00	1.69E+00
Breast	Income	0.200	0.048	2.98E-05	5.16E-03	2.10E+00	1.66E+00
Liver	Tobacco:LM.Cannabis:Herb.THC	0.157	0.046	6.92E-04	1.04E-01	2.29E+00	1.63E+00
Non-Hodgkins lymphoma	Tobacco:Herb.THC	0.344	0.132	9.62E-03	1.00E+00	3.27E+00	1.63E+00
Oropharynx	Tobacco:LM.Cannabis	0.086	0.028	2.24E-03	3.11E-01	2.44E+00	1.63E+00
Myeloma	LM.Cannabis	0.135	0.048	5.05E-03	6.52E-01	2.77E+00	1.62E+00
Prostate	Income	0.279	0.069	5.83E-05	9.91E-03	2.06E+00	1.62E+00
Colorectum	Tobacco	0.119	0.009	2.97E-37	8.49E-35	1.58E+00	1.52E+00
Breast	Tobacco	0.088	0.007	3.68E-35	1.04E-32	1.57E+00	1.50E+00
Prostate	Tobacco:LM.Cannabis:Herb.THC	0.281	0.097	3.90E-03	5.11E-01	2.06E+00	1.45E+00
Stomach	Tobacco	0.081	0.007	5.69E-27	1.55E-24	1.51E+00	1.45E+00
Colorectum	Alcohol	0.120	0.020	5.07E-09	1.06E-06	1.58E+00	1.44E+00
All Cancers nNMSC	Tobacco:LM.Cannabis	0.017	0.002	6.89E-24	1.86E-21	1.47E+00	1.40E+00
Testis	Tobacco:LM.Cannabis:Herb.THC	0.264	0.101	9.25E-03	1.00E+00	2.00E+00	1.36E+00
Pancreas	Tobacco:LM.Cannabis:Herb.THC	0.112	0.043	9.81E-03	1.00E+00	1.97E+00	1.34E+00
Corpus Uteri	Tobacco	0.073	0.010	4.44E-14	1.09E-11	1.40E+00	1.33E+00
Oesophagus	Alcohol	0.055	0.012	1.12E-05	2.00E-03	1.48E+00	1.32E+00
Testis	Tobacco	0.075	0.012	3.13E-09	6.64E-07	1.39E+00	1.30E+00
Prostate	Tobacco	0.069	0.010	8.15E-12	1.87E-09	1.37E+00	1.30E+00
Stomach	Alcohol	0.067	0.017	7.62E-05	1.28E-02	1.44E+00	1.28E+00
Ovary	Tobacco	0.052	0.008	1.06E-10	2.36E-08	1.36E+00	1.28E+00
Liver	Tobacco:LM.Cannabis	0.016	0.002	2.54E-11	5.74E-09	1.25E+00	1.20E+00
Breast	Alcohol	0.048	0.016	2.14E-03	3.00E-01	1.37E+00	1.20E+00
Melanoma	Tobacco:LM.Cannabis	0.019	0.003	4.89E-11	1.10E-08	1.24E+00	1.19E+00
Lung	Tobacco:LM.Cannabis	0.011	0.002	4.76E-11	1.07E-08	1.24E+00	1.19E+00

Bladder	LM.Cannabis	0.145	0.069	3.63E-02	1.00E+00	2.24E+00	1.18E+00
Pancreas	Tobacco:LM.Cannabis	0.014	0.002	9.55E-10	2.07E-07	1.23E+00	1.18E+00
Larynx	Tobacco	0.019	0.005	2.06E-04	3.32E-02	1.25E+00	1.16E+00
Cervix	Alcohol	0.060	0.022	6.45E-03	8.20E-01	1.35E+00	1.16E+00
Kidney	Tobacco:LM.Cannabis	0.012	0.002	5.20E-07	1.02E-04	1.20E+00	1.15E+00
Prostate	Alcohol	0.058	0.023	9.90E-03	1.00E+00	1.33E+00	1.14E+00
Oesophagus	Tobacco:LM.Cannabis	0.013	0.003	7.85E-06	1.42E-03	1.19E+00	1.13E+00
Non-Hodgkins lymphoma	Resin.THC	0.374	0.188	4.71E-02	1.00E+00	3.50E+00	1.12E+00
Bladder	Tobacco	0.012	0.004	6.51E-03	8.20E-01	1.21E+00	1.10E+00
Non-Hodgkins lymphoma	Tobacco:LM.Cannabis	0.010	0.003	7.32E-04	1.09E-01	1.16E+00	1.10E+00
Thyroid	Alcohol	0.027	0.013	3.21E-02	1.00E+00	1.34E+00	1.08E+00
Brain	Tobacco	0.011	0.005	2.49E-02	1.00E+00	1.19E+00	1.06E+00
Anus	Tobacco:LM.Cannabis	0.004	0.002	2.71E-02	1.00E+00	1.13E+00	1.04E+00

Supplementary Table S30.: Summary by Terms of Significant Positive

Terms from Interactive Panel Regression at Two Temporal Lags

Term	Count	Negative Total of P-Value Exponents	Mean of the Negative P-Value Exponents	Median of the Negative P-Value Exponents	Total of the Lower E-Value Exponents	Mean of the Lower E-Value Exponents	Median of the Lower E-Value Exponents
Herb.THC	18	121	6.72	4.5	176	9.78	10.5
Resin.THC	9	74	8.22	4	19	2.11	0
LM.Cannabis : Herb.THC	1	1	1	1	3	3.00	3
Tobacco : Herb.THC	3	23	7.67	8	3	1.00	1
Last.Month.Cannabis	12	135	11.25	6	2	0.17	0
Income	5	22	4.4	4	2	0.4	0
Alcohol	11	65	5.91	4	0	0	0
Tobacco	12	194	16.17	10	0	0	0
Tobacco : LM.Cannabis	10	79	7.90	7.5	0	0	0
Tobacco:LM.Cannabis:Herb.THC	15	83	5.53	3	0	0	0

Supplementary Table S31.: Complete Model Output from

Interactive Panel Regression at Four Temporal Lags

Cancer	Term	ß-Estimate	Std. Error	P-Value	P.Adj.Holm	E-Value Estimate	95% Lower Bound of the E-Value
Oropharynx	LM.Cannabis:Herb.THC	34.320	7.372	1.12E-05	0.0021349	2.11E+95	2.49E+55
Colorectum	Herb.THC	92.213	8.721	8.28E-25	2.31E-22	3.79E+46	1.04E+38
Gallbladder & Biliary	Herb.THC	29.693	3.555	4.60E-16	1.19E-13	1.61E+42	2.47E+32
Thyroid	Herb.THC	33.741	4.103	1.03E-15	2.63E-13	3.50E+39	1.62E+30
Breast	Herb.THC	51.744	6.784	5.76E-14	1.43E-11	5.52E+33	1.47E+25
Anus	Herb.THC	24.891	3.505	3.19E-12	7.62E-10	1.56E+34	7.22E+24
Oropharynx	Resin.THC	9.947	1.105	3.68E-14	9.19E-12	6.95E+27	7.23E+21
Oesophagus	Herb.THC	31.178	5.101	1.42E-09	3.25E-07	1.12E+27	3.09E+18
Myeloma	Herb.THC	15.860	2.788	1.93E-08	4.18E-06	2.76E+27	1.28E+18
All Cancers	Herb.THC	13.310	2.871	4.70E-06	9.20E-04	3.99E+26	3.19E+15
Melanoma	Herb.THC	24.578	5.069	1.45E-06	2.91E-04	3.61E+21	9.59E+12
Liver	Herb.THC	19.895	4.541	1.38E-05	0.0025785	9.79E+21	2.02E+12
Hodgkins	Herb.THC	13.432	3.190	2.89E-05	0.0052919	2.48E+20	1.15E+11
Testis	Herb.THC	45.669	10.959	3.49E-05	0.0062793	1.53E+20	7.12E+10
Oropharynx_Broad	Herb.THC	21.554	7.876	6.52E-03	0.827903	1.32E+23	6.45E+06
Bladder	Herb.THC	13.187	4.024	1.09E-03	0.1682717	4.41E+14	1.21E+06
Oropharynx	Tobacco:Herb.THC	1.120	0.180	1.69E-08	3.69E-06	2.52E+03	2.65E+02
Stomach	Herb.THC	17.035	7.068	1.61E-02	1	7.08E+10	1.94E+02
Oropharynx_Broad	LM.Cannabis	6.254	2.287	6.55E-03	0.827903	8.37E+06	1.53E+02
Oesophagus	Resin.THC	1.932	0.206	5.48E-20	1.47E-17	9.03E+01	4.04E+01
Colorectum	LM.Cannabis	2.338	0.184	2.13E-34	6.15E-32	2.93E+01	1.92E+01
Thyroid	LM.Cannabis	0.980	0.087	3.18E-27	9.00E-25	2.71E+01	1.70E+01

Myeloma	Resin.THC	0.719	0.116	9.36E-10	2.16E-07	3.35E+01	1.34E+01
Larynx	Resin.THC	1.162	0.197	5.11E-09	1.14E-06	2.16E+01	9.45E+00
Stomach	LM.Cannabis	1.329	0.149	2.34E-18	6.22E-16	1.28E+01	8.25E+00
Hodgkins	LM.Cannabis	0.553	0.067	1.24E-15	3.16E-13	1.29E+01	7.99E+00
Larynx	LM.Cannabis	0.868	0.103	1.04E-16	2.70E-14	1.15E+01	7.42E+00
Cervix	LM.Cannabis:Herb.THC	8.526	3.781	2.44E-02	1	1.75E+04	6.12E+00
Breast	LM.Cannabis	1.063	0.144	2.98E-13	7.31E-11	9.20E+00	5.86E+00
Thyroid	Tobacco:LM.Cannabis:Herb.THC	0.505	0.050	9.13E-23	2.50E-20	7.19E+00	5.41E+00
Colorectum	Tobacco:LM.Cannabis:Herb.THC	1.066	0.113	3.06E-20	8.30E-18	6.31E+00	4.75E+00
Gallbladder & Biliary	Tobacco:LM.Cannabis:Herb.THC	0.312	0.044	2.95E-12	7.07E-10	4.95E+00	3.59E+00
Myeloma	LM.Cannabis	0.297	0.059	6.19E-07	1.26E-04	5.89E+00	3.50E+00
Oropharynx	Tobacco:LM.Cannabis	0.157	0.024	6.02E-09	1.34E-06	4.89E+00	3.43E+00
Hodgkins	Resin.THC	0.459	0.132	5.58E-04	0.0898609	9.20E+00	3.40E+00
Leukaemia - Myeloid	Alcohol	0.269	0.066	8.04E-05	0.0139935	6.31E+00	3.21E+00
Hodgkins	Tobacco:LM.Cannabis:Herb.THC	0.236	0.038	1.52E-09	3.45E-07	3.93E+00	2.87E+00
Leukaemia - Lymphoid	Alcohol	0.119	0.032	2.61E-04	0.0435782	5.67E+00	2.84E+00
Anus	Tobacco:LM.Cannabis:Herb.THC	0.247	0.042	7.88E-09	1.73E-06	3.77E+00	2.74E+00
Colorectum	Resin.THC	1.124	0.352	1.47E-03	0.2226953	6.79E+00	2.69E+00
Myeloma	Tobacco:LM.Cannabis:Herb.THC	0.190	0.034	2.58E-08	5.56E-06	3.64E+00	2.64E+00
Breast	Tobacco:LM.Cannabis:Herb.THC	0.494	0.089	3.60E-08	7.71E-06	3.59E+00	2.60E+00
Lung	Resin.THC	0.350	0.116	2.49E-03	0.3617381	6.33E+00	2.47E+00
All Cancers	Tobacco:LM.Cannabis:Herb.THC	0.253	0.081	1.92E-03	0.2848745	5.77E+00	2.44E+00
Testis	LM.Cannabis	0.809	0.232	5.12E-04	0.0829502	3.93E+00	2.21E+00
Gallbladder & Biliary	LM.Cannabis	0.261	0.079	9.97E-04	0.156563	4.09E+00	2.18E+00
Liver	Tobacco:LM.Cannabis:Herb.THC	0.230	0.054	2.17E-05	0.0039876	2.96E+00	2.08E+00
Oesophagus	Tobacco:LM.Cannabis:Herb.THC	0.274	0.066	3.76E-05	0.0066981	2.83E+00	1.99E+00
Gallbladder & Biliary	Tobacco	0.102	0.008	4.70E-36	1.36E-33	2.13E+00	1.99E+00
Brain	Tobacco:Herb.THC	0.419	0.148	4.73E-03	0.6338577	4.43E+00	1.99E+00
Testis	Income	0.520	0.133	1.05E-04	0.0181315	2.76E+00	1.92E+00
Corpus Uteri	LM.Cannabis	0.587	0.198	3.14E-03	0.4399844	3.16E+00	1.78E+00

Larynx	Alcohol	0.128	0.015	1.22E-17	3.22E-15	1.93E+00	1.76E+00
Cervix	Tobacco:Herb.THC	0.785	0.304	9.85E-03	1	4.04E+00	1.75E+00
Brain	Income	0.161	0.041	8.51E-05	0.0147164	2.19E+00	1.67E+00
All Cancers	Alcohol	0.061	0.013	5.80E-06	0.0011256	1.96E+00	1.62E+00
Breast	Income	0.218	0.060	2.97E-04	0.0489266	2.11E+00	1.59E+00
Stomach	Tobacco:LM.Cannabis:Herb.THC	0.283	0.092	2.06E-03	0.3030811	2.36E+00	1.59E+00
Colorectum	Tobacco	0.147	0.012	8.63E-31	2.49E-28	1.65E+00	1.57E+00
Testis	Tobacco:LM.Cannabis:Herb.THC	0.396	0.132	2.84E-03	0.4051248	2.34E+00	1.56E+00
Breast	Tobacco	0.097	0.010	4.73E-23	1.30E-20	1.58E+00	1.50E+00
Melanoma	Tobacco:LM.Cannabis:Herb.THC	0.185	0.066	5.49E-03	0.718574	2.25E+00	1.47E+00
Stomach	Tobacco	0.078	0.010	1.61E-14	4.04E-12	1.48E+00	1.39E+00
Oropharynx_Broad	Tobacco	0.098	0.036	5.89E-03	0.7595198	1.86E+00	1.35E+00
Colorectum	Alcohol	0.109	0.026	3.19E-05	0.0058109	1.53E+00	1.34E+00
Prostate	Income	0.232	0.087	7.59E-03	0.9333969	1.85E+00	1.33E+00
Testis	Tobacco	0.087	0.018	9.88E-07	1.99E-04	1.41E+00	1.29E+00
Myeloma	Income	0.079	0.034	1.98E-02	1	2.07E+00	1.29E+00
Hodgkins	Alcohol	0.035	0.011	1.02E-03	0.1593218	1.51E+00	1.28E+00
All Cancers nNMSC	Tobacco:LM.Cannabis	0.012	0.002	7.07E-07	1.43E-04	1.37E+00	1.27E+00
Corpus Uteri	Tobacco	0.069	0.013	3.00E-07	6.21E-05	1.36E+00	1.27E+00
Larynx	Tobacco:LM.Cannabis:Herb.THC	0.146	0.063	2.14E-02	1	2.04E+00	1.27E+00
Thyroid	Alcohol	0.042	0.014	2.57E-03	0.3699626	1.48E+00	1.24E+00
Stomach	Alcohol	0.068	0.021	1.39E-03	0.2109464	1.44E+00	1.24E+00
Prostate	Tobacco	0.061	0.014	1.14E-05	0.0021569	1.33E+00	1.23E+00
Ovary	Tobacco	0.047	0.011	2.03E-05	0.0037506	1.32E+00	1.22E+00
Pancreas	Tobacco:LM.Cannabis	0.016	0.003	1.32E-09	3.01E-07	1.26E+00	1.20E+00
Lung	Tobacco:LM.Cannabis	0.011	0.002	6.83E-09	1.51E-06	1.25E+00	1.19E+00
Oesophagus	Alcohol	0.043	0.015	4.75E-03	0.6338577	1.40E+00	1.19E+00
All Cancers nNMSC	Tobacco:Herb.THC	0.213	0.107	4.65E-02	1	7.37E+00	1.19E+00
Kidney	Tobacco:LM.Cannabis	0.014	0.003	2.20E-07	4.60E-05	1.23E+00	1.18E+00
Breast	Alcohol	0.055	0.020	7.36E-03	0.9130061	1.39E+00	1.17E+00

Bladder	Tobacco	0.020	0.006	6.23E-04	0.0997405	1.28E+00	1.17E+00
Melanoma	Tobacco:LM.Cannabis	0.016	0.003	5.53E-06	0.0010786	1.21E+00	1.15E+00
Liver	Tobacco:LM.Cannabis	0.012	0.003	3.71E-05	0.0066328	1.21E+00	1.15E+00
Larynx	Tobacco	0.020	0.007	3.22E-03	0.4470402	1.25E+00	1.13E+00
Thyroid	Tobacco	0.018	0.007	7.95E-03	0.9622921	1.27E+00	1.13E+00
Oesophagus	Tobacco:LM.Cannabis	0.011	0.003	1.81E-03	0.2719305	1.17E+00	1.10E+00
Non-Hodgkins lymphoma	Tobacco:LM.Cannabis	0.012	0.004	2.83E-03	0.4051248	1.17E+00	1.09E+00
Kidney	Tobacco:LM.Cannabis:Herb.THC	0.102	0.052	4.97E-02	1	1.91E+00	1.04E+00
Corpus Uteri	Alcohol	0.002	0.028	9.41E-01	1	1.05E+00	1.00E+00
Brain	Tobacco	0.002	0.007	7.27E-01	1	1.08E+00	1.00E+00
Anus	Tobacco:LM.Cannabis	0.003	0.002	1.45E-01	1	1.12E+00	1.00E+00
Brain	Tobacco:LM.Cannabis	0.005	0.003	1.16E-01	1	1.12E+00	1.00E+00
Anus	Tobacco	0.003	0.006	5.38E-01	1	1.12E+00	1.00E+00
Cervix	Tobacco:LM.Cannabis	0.010	0.006	1.13E-01	1	1.12E+00	1.00E+00
Hodgkins	Tobacco	0.005	0.005	3.44E-01	1	1.15E+00	1.00E+00
Prostate	Tobacco:Herb.THC	0.018	0.316	9.55E-01	1	1.16E+00	1.00E+00
Myeloma	Alcohol	0.005	0.009	5.98E-01	1	1.16E+00	1.00E+00
Testis	Alcohol	0.035	0.037	3.39E-01	1	1.23E+00	1.00E+00
Prostate	Alcohol	0.045	0.030	1.30E-01	1	1.27E+00	1.00E+00
Cervix	Alcohol	0.052	0.028	6.94E-02	1	1.30E+00	1.00E+00
Lung	Tobacco:LM.Cannabis:Herb.THC	0.019	0.037	6.01E-01	1	1.35E+00	1.00E+00
Kidney	Tobacco:Herb.THC	0.029	0.128	8.19E-01	1	1.36E+00	1.00E+00
Ovary	LM.Cannabis	0.067	0.162	6.77E-01	1	1.41E+00	1.00E+00
Leukaemia - Myeloid	Tobacco:LM.Cannabis:Herb.THC	0.022	0.236	9.27E-01	1	1.45E+00	1.00E+00
Corpus Uteri	Tobacco:LM.Cannabis:Herb.THC	0.098	0.122	4.21E-01	1	1.46E+00	1.00E+00
Oropharynx	Alcohol	0.017	0.059	7.76E-01	1	1.47E+00	1.00E+00
Hodgkins	Income	0.031	0.039	4.17E-01	1	1.47E+00	1.00E+00
Prostate	Tobacco:LM.Cannabis:Herb.THC	0.120	0.128	3.47E-01	1	1.52E+00	1.00E+00
Non-Hodgkins lymphoma	Tobacco:LM.Cannabis:Herb.THC	0.073	0.075	3.27E-01	1	1.55E+00	1.00E+00
Pancreas	Tobacco:LM.Cannabis:Herb.THC	0.053	0.051	2.99E-01	1	1.55E+00	1.00E+00

Oropharynx_Broad	Income	0.075	0.063	2.39E-01	1	1.69E+00	1.00E+00
Bladder	Tobacco:LM.Cannabis:Herb.THC	0.080	0.052	1.27E-01	1	1.74E+00	1.00E+00
Pancreas	Tobacco:Herb.THC	0.082	0.125	5.15E-01	1	1.77E+00	1.00E+00
All Cancers nNMSC	Tobacco:LM.Cannabis:Herb.THC	0.038	0.038	3.27E-01	1	1.87E+00	1.00E+00
Bladder	LM.Cannabis	0.131	0.085	1.22E-01	1	2.12E+00	1.00E+00
Prostate	LM.Cannabis	0.325	0.207	1.17E-01	1	2.14E+00	1.00E+00
Lung	Tobacco:Herb.THC	0.120	0.092	1.90E-01	1	2.42E+00	1.00E+00
Leukaemia - Myeloid	Income	0.097	0.306	7.52E-01	1	2.49E+00	1.00E+00
Ovary	Tobacco:Herb.THC	0.369	0.246	1.34E-01	1	2.63E+00	1.00E+00
Non-Hodgkins lymphoma	Tobacco:Herb.THC	0.286	0.179	1.10E-01	1	2.76E+00	1.00E+00
Non-Hodgkins lymphoma	Resin.THC	0.292	0.231	2.06E-01	1	2.80E+00	1.00E+00
All Cancers	LM.Cannabis	0.123	0.291	6.72E-01	1	2.90E+00	1.00E+00
Oropharynx_Broad	Alcohol	0.231	0.139	9.67E-02	1	2.91E+00	1.00E+00
Bladder	Resin.THC	0.317	0.163	5.21E-02	1	3.85E+00	1.00E+00
Leukaemia - Myeloid	LM.Cannabis	0.297	0.937	7.52E-01	1	7.28E+00	1.00E+00
Oropharynx_Broad	Tobacco:LM.Cannabis:Herb.THC	0.579	0.546	2.90E-01	1	7.66E+00	1.00E+00
Ovary	LM.Cannabis:Herb.THC	1.264	3.066	6.80E-01	1	9.99E+00	1.00E+00
Leukaemia - Lymphoid	LM.Cannabis	0.235	0.450	6.03E-01	1	1.83E+01	1.00E+00
Oropharynx	Income	0.376	0.248	1.32E-01	1	2.15E+01	1.00E+00
Lung	Herb.THC	1.039	2.860	7.16E-01	1	7.74E+01	1.00E+00
Brain	LM.Cannabis:Herb.THC	1.981	1.844	2.83E-01	1	1.51E+02	1.00E+00
Leukaemia - Myeloid	LM.Cannabis:Herb.THC	1.351	8.047	8.67E-01	1	9.80E+02	1.00E+00
Prostate	Herb.THC	7.192	9.839	4.65E-01	1	3.16E+03	1.00E+00
Corpus Uteri	Herb.THC	8.034	9.413	3.94E-01	1	1.09E+04	1.00E+00
Pancreas	Herb.THC	4.554	3.906	2.44E-01	1	2.54E+05	1.00E+00
Leukaemia - Lymphoid	LM.Cannabis:Herb.THC	1.347	3.864	7.28E-01	1	7.79E+05	1.00E+00
Kidney	Herb.THC	5.315	3.984	1.83E-01	1	1.38E+06	1.00E+00
Larynx	Herb.THC	6.741	4.871	1.67E-01	1	2.28E+06	1.00E+00
Leukaemia - Myeloid	Herb.THC	3.274	8.166	6.89E-01	1	6.66E+06	1.00E+00
Leukaemia - Lymphoid	Herb.THC	3.064	3.921	4.36E-01	1	1.03E+13	1.00E+00

Kaposi	Tobacco	62.282	102.530	5.46E-01	1	1.02E+125	1.00E+00
Kaposi	Tobacco:LM.Cannabis:Herb.THC	64.795	91.953	4.85E-01	1	1.10E+130	1.00E+00
Kaposi	LM.Cannabis	846.442	1327.503	5.27E-01	1	Inf	1.00E+00
Kaposi	LM.Cannabis:Herb.THC	2757.893	4512.526	5.44E-01	1	Inf	1.00E+00
Ovary	Tobacco:LM.Cannabis	-0.004	0.005	4.83E-01	1	1.07E+00	-
Bladder	Tobacco:LM.Cannabis	-0.003	0.003	3.60E-01	1	1.09E+00	-
Cervix	Tobacco	-0.008	0.013	5.67E-01	1	1.10E+00	-
Melanoma	Tobacco	-0.005	0.007	5.25E-01	1	1.11E+00	-
Oesophagus	Tobacco	-0.005	0.007	5.12E-01	1	1.11E+00	-
Prostate	Tobacco:LM.Cannabis	-0.011	0.007	1.05E-01	1	1.12E+00	-
Kidney	Tobacco	-0.006	0.006	3.06E-01	1	1.14E+00	-
All Cancers	Tobacco	-0.004	0.004	3.84E-01	1	1.15E+00	-
Myeloma	Tobacco	-0.005	0.004	2.75E-01	1	1.16E+00	-
Corpus Uteri	Tobacco:LM.Cannabis	-0.019	0.006	3.04E-03	0.4292627	1.17E+00	-
Testis	Tobacco:LM.Cannabis	-0.027	0.007	3.35E-04	0.0550099	1.19E+00	-
Myeloma	Tobacco:LM.Cannabis	-0.007	0.002	1.13E-04	0.0192927	1.20E+00	-
Pancreas	Tobacco	-0.012	0.006	2.81E-02	1	1.21E+00	-
Gallbladder & Biliary	Income	-0.010	0.043	8.12E-01	1	1.22E+00	-
All Cancers	Tobacco:LM.Cannabis	-0.008	0.009	3.86E-01	1	1.23E+00	-
Corpus Uteri	Income	-0.036	0.083	6.61E-01	1	1.24E+00	-
Lung	Tobacco	-0.011	0.004	5.59E-03	0.7262569	1.25E+00	-
Leukaemia - Lymphoid	Tobacco	-0.004	0.012	7.32E-01	1	1.25E+00	-
Gallbladder & Biliary	Tobacco:LM.Cannabis	-0.013	0.002	3.93E-08	8.37E-06	1.26E+00	-
Larynx	Tobacco:LM.Cannabis	-0.023	0.003	9.73E-12	2.30E-09	1.27E+00	-
Corpus Uteri	Tobacco:Herb.THC	-0.045	0.302	8.81E-01	1	1.28E+00	-
Breast	Tobacco:LM.Cannabis	-0.033	0.005	2.67E-12	6.49E-10	1.28E+00	-
Ovary	Alcohol	-0.039	0.023	8.86E-02	1	1.29E+00	-
Hodgkins	Tobacco:LM.Cannabis	-0.016	0.002	2.86E-13	7.04E-11	1.30E+00	-
Stomach	Tobacco:LM.Cannabis	-0.041	0.005	6.99E-17	1.82E-14	1.31E+00	-
Oropharynx_Broad	Resin.THC	-0.025	1.194	9.83E-01	1	1.32E+00	-

Ovary	Tobacco:LM.Cannabis:Herb.THC	-0.049	0.100	6.21E-01	1	1.33E+00	-
Leukaemia - Myeloid	Tobacco:LM.Cannabis	-0.015	0.027	5.95E-01	1	1.34E+00	-
Ovary	Income	-0.054	0.068	4.30E-01	1	1.35E+00	-
Leukaemia - Lymphoid	Tobacco:LM.Cannabis	-0.008	0.013	5.62E-01	1	1.36E+00	-
Oropharynx	Tobacco	-0.012	0.021	5.68E-01	1	1.37E+00	-
Liver	Tobacco	-0.031	0.007	1.88E-05	0.0035006	1.37E+00	-
Colorectum	Tobacco:LM.Cannabis	-0.069	0.006	4.15E-29	1.18E-26	1.38E+00	-
Thyroid	Tobacco:LM.Cannabis	-0.030	0.003	4.91E-25	1.37E-22	1.38E+00	-
Non-Hodgkins lymphoma	Tobacco	-0.049	0.008	7.59E-10	1.76E-07	1.41E+00	-
All Cancers nNMSC	Tobacco	-0.017	0.005	2.13E-03	0.3105389	1.47E+00	-
Brain	Tobacco:LM.Cannabis:Herb.THC	-0.053	0.060	3.80E-01	1	1.49E+00	-
Leukaemia - Myeloid	Tobacco	-0.025	0.025	3.26E-01	1	1.49E+00	-
Brain	Alcohol	-0.056	0.014	5.37E-05	0.009444	1.52E+00	-
Non-Hodgkins lymphoma	Alcohol	-0.074	0.017	9.77E-06	0.0018667	1.55E+00	-
Lung	Alcohol	-0.040	0.009	3.39E-06	6.70E-04	1.57E+00	-
Colorectum	Income	-0.151	0.077	5.00E-02	1	1.67E+00	-
Anus	Alcohol	-0.056	0.012	2.49E-06	4.95E-04	1.67E+00	-
Thyroid	Income	-0.087	0.050	8.20E-02	1	1.84E+00	-
Melanoma	Alcohol	-0.119	0.015	1.51E-14	3.81E-12	1.85E+00	-
Cervix	Income	-0.236	0.084	4.88E-03	0.6436394	1.89E+00	-
Melanoma	Income	-0.132	0.045	3.28E-03	0.4527736	1.93E+00	-
Larynx	Tobacco:Herb.THC	-0.128	0.156	4.14E-01	1	1.93E+00	-
Cervix	Tobacco:LM.Cannabis:Herb.THC	-0.260	0.123	3.47E-02	1	1.97E+00	-
Kidney	Alcohol	-0.109	0.012	3.84E-19	1.03E-16	1.97E+00	-
Stomach	Tobacco:Herb.THC	-0.220	0.227	3.32E-01	1	2.08E+00	-
Bladder	Alcohol	-0.128	0.012	1.02E-24	2.84E-22	2.10E+00	-
Leukaemia - Lymphoid	Tobacco:LM.Cannabis:Herb.THC	-0.034	0.113	7.65E-01	1	2.11E+00	-
Pancreas	Alcohol	-0.126	0.012	2.15E-25	6.03E-23	2.11E+00	-
Liver	Alcohol	-0.135	0.015	4.10E-19	1.10E-16	2.16E+00	-
Larynx	Income	-0.201	0.043	3.38E-06	6.70E-04	2.40E+00	-

Anus	LM.Cannabis	-0.135	0.074	6.93E-02	1	2.42E+00	-
Kaposi	Income	-0.093	0.899	9.18E-01	1	2.44E+00	-
Breast	Resin.THC	-0.302	0.276	2.75E-01	1	2.51E+00	-
Liver	Income	-0.182	0.053	6.73E-04	0.1069288	2.54E+00	-
Oropharynx_Broad	Tobacco:LM.Cannabis	-0.194	0.071	6.61E-03	0.827903	2.59E+00	-
Cervix	LM.Cannabis	-0.445	0.200	2.59E-02	1	2.59E+00	-
Brain	LM.Cannabis	-0.221	0.097	2.32E-02	1	2.62E+00	-
Gallbladder & Biliary	Alcohol	-0.156	0.014	3.38E-27	9.52E-25	2.70E+00	-
Anus	Income	-0.162	0.043	1.51E-04	0.0257445	2.72E+00	-
Melanoma	Resin.THC	-0.264	0.207	2.02E-01	1	2.77E+00	-
All Cancers nNMSC	Alcohol	-0.086	0.007	9.05E-30	2.58E-27	2.87E+00	-
Oesophagus	Income	-0.313	0.045	7.26E-12	1.73E-09	3.11E+00	-
Bladder	Tobacco:Herb.THC	-0.252	0.129	5.08E-02	1	3.17E+00	-
Non-Hodgkins lymphoma	LM.Cannabis	-0.372	0.119	1.83E-03	0.2732197	3.36E+00	-
Oesophagus	LM.Cannabis	-0.345	0.108	1.37E-03	0.2098952	3.37E+00	-
Stomach	Income	-0.512	0.062	7.08E-16	1.81E-13	3.57E+00	-
Non-Hodgkins lymphoma	Income	-0.407	0.049	6.78E-16	1.74E-13	3.62E+00	-
Leukaemia - Myeloid	Tobacco:Herb.THC	-0.167	0.236	4.81E-01	1	3.73E+00	-
Bladder	Income	-0.309	0.036	1.30E-17	3.41E-15	3.76E+00	-
Liver	LM.Cannabis	-0.319	0.093	6.84E-04	0.1081087	3.88E+00	-
Leukaemia - Lymphoid	Tobacco:Herb.THC	-0.102	0.113	3.70E-01	1	4.74E+00	-
Testis	Resin.THC	-1.128	0.455	1.34E-02	1	5.65E+00	-
All Cancers	Income	-0.253	0.029	1.90E-17	4.97E-15	5.77E+00	-
Lung	LM.Cannabis:Herb.THC	-0.341	1.142	7.65E-01	1	6.10E+00	-
Melanoma	LM.Cannabis	-0.622	0.107	9.68E-09	2.12E-06	6.35E+00	-
Lung	LM.Cannabis	-0.370	0.060	1.18E-09	2.72E-07	6.83E+00	-
Kidney	LM.Cannabis	-0.529	0.084	4.58E-10	1.07E-07	7.09E+00	-
Liver	Tobacco:Herb.THC	-0.547	0.149	2.74E-04	0.0454271	7.35E+00	-
All Cancers	Tobacco:Herb.THC	-0.308	0.095	1.22E-03	0.1881044	7.59E+00	-
Melanoma	Tobacco:Herb.THC	-0.713	0.163	1.33E-05	0.0024916	7.73E+00	-

Testis	Tobacco:Herb.THC	-1.450	0.364	7.55E-05	0.0132093	8.03E+00	-
Pancreas	LM.Cannabis	-0.569	0.082	8.91E-12	2.11E-09	8.15E+00	-
Hodgkins	Tobacco:Herb.THC	-0.443	0.106	3.36E-05	0.0060775	8.65E+00	-
Lung	Income	-0.444	0.025	2.75E-60	8.03E-58	9.03E+00	-
Pancreas	Income	-0.626	0.034	1.47E-63	4.31E-61	9.52E+00	-
Myeloma	Tobacco:Herb.THC	-0.420	0.093	6.87E-06	0.0013251	9.94E+00	-
Kidney	Income	-0.669	0.035	7.43E-69	2.18E-66	1.03E+01	-
Oesophagus	Tobacco:Herb.THC	-0.877	0.164	1.05E-07	2.22E-05	1.08E+01	-
Stomach	Resin.THC	-1.464	0.286	3.72E-07	7.67E-05	1.56E+01	-
Corpus Uteri	Resin.THC	-2.050	0.380	8.88E-08	1.88E-05	1.74E+01	-
Anus	Tobacco:Herb.THC	-0.704	0.116	2.42E-09	5.48E-07	1.77E+01	-
Breast	Tobacco:Herb.THC	-1.543	0.218	2.74E-12	6.61E-10	1.93E+01	-
All Cancers nNMSC	LM.Cannabis	-0.393	0.078	5.64E-07	1.16E-04	2.47E+01	-
Leukaemia - Lymphoid	Income	-0.266	0.147	7.31E-02	1	2.48E+01	-
Prostate	Resin.THC	-2.625	0.398	6.66E-11	1.57E-08	2.89E+01	-
Oropharynx_Broad	Tobacco:Herb.THC	-1.154	0.306	1.94E-04	0.0325134	3.29E+01	-
Brain	Resin.THC	-1.375	0.187	3.94E-13	9.61E-11	3.97E+01	-
All Cancers nNMSC	Income	-0.472	0.022	1.94E-84	5.75E-82	4.16E+01	-
Ovary	Resin.THC	-2.332	0.311	1.36E-13	3.36E-11	4.22E+01	-
Cervix	Resin.THC	-2.927	0.383	5.10E-14	1.27E-11	4.46E+01	-
Leukaemia - Myeloid	Resin.THC	-0.680	1.055	5.20E-01	1	4.48E+01	-
Colorectum	Tobacco:Herb.THC	-2.734	0.280	1.41E-21	3.85E-19	4.66E+01	-
Thyroid	Tobacco:Herb.THC	-1.194	0.136	1.58E-17	4.15E-15	4.85E+01	-
Pancreas	LM.Cannabis:Herb.THC	-1.303	1.560	4.04E-01	1	5.71E+01	-
All Cancers nNMSC	Resin.THC	-0.522	0.116	7.07E-06	0.0013569	5.77E+01	-
Non-Hodgkins lymphoma	LM.Cannabis:Herb.THC	-2.020	2.276	3.75E-01	1	7.93E+01	-
Prostate	LM.Cannabis:Herb.THC	-3.765	3.930	3.38E-01	1	9.41E+01	-
Corpus Uteri	LM.Cannabis:Herb.THC	-3.619	3.760	3.36E-01	1	9.58E+01	-
Gallbladder & Biliary	Tobacco:Herb.THC	-1.209	0.123	2.75E-21	7.48E-19	1.01E+02	-
Kidney	Resin.THC	-1.673	0.161	5.27E-24	1.46E-21	1.37E+02	-

Anus	Resin.THC	-1.383	0.145	3.66E-20	9.89E-18	1.52E+02	-
Pancreas	Resin.THC	-1.864	0.158	4.14E-30	1.18E-27	2.45E+02	-
All Cancers	Resin.THC	-1.104	0.136	4.26E-15	1.08E-12	3.03E+02	-
Thyroid	Resin.THC	-2.048	0.170	2.93E-30	8.42E-28	4.81E+02	-
Kidney	LM.Cannabis:Herb.THC	-2.346	1.591	1.41E-01	1	7.55E+02	-
All Cancers nNMSC	LM.Cannabis:Herb.THC	-0.929	1.215	4.45E-01	1	8.01E+02	-
Leukaemia - Lymphoid	Resin.THC	-0.689	0.507	1.76E-01	1	1.45E+03	-
Ovary	Herb.THC	-5.348	7.675	4.86E-01	1	2.24E+03	-
Oropharynx	Tobacco:LM.Cannabis:Herb.THC	-1.181	0.220	6.24E-07	1.27E-04	3.72E+03	-
Gallbladder & Biliary	Resin.THC	-2.662	0.193	7.75E-38	2.26E-35	1.14E+04	-
Non-Hodgkins lymphoma	Herb.THC	-4.993	5.556	3.69E-01	1	1.81E+04	-
Melanoma	LM.Cannabis:Herb.THC	-4.662	2.039	2.24E-02	1	2.15E+04	-
Liver	Resin.THC	-4.048	0.189	1.37E-77	4.04E-75	5.18E+04	-
Bladder	LM.Cannabis:Herb.THC	-4.604	1.607	4.27E-03	0.5765123	2.03E+05	-
Larynx	LM.Cannabis:Herb.THC	-5.687	1.946	3.55E-03	0.4827118	2.58E+05	-
Testis	LM.Cannabis:Herb.THC	-12.035	4.088	3.35E-03	0.4590646	3.48E+05	-
All Cancers nNMSC	Herb.THC	-1.880	3.436	5.84E-01	1	3.72E+05	-
Liver	LM.Cannabis:Herb.THC	-5.932	1.670	4.09E-04	0.0666225	5.87E+06	-
Stomach	LM.Cannabis:Herb.THC	-10.569	2.823	1.92E-04	0.0324108	7.02E+06	-
Oesophagus	LM.Cannabis:Herb.THC	-8.375	2.038	4.29E-05	0.0075983	3.06E+07	-
Cervix	Herb.THC	-20.218	9.466	3.29E-02	1	4.46E+09	-
Anus	LM.Cannabis:Herb.THC	-6.886	1.308	1.88E-07	3.95E-05	4.76E+09	-
Breast	LM.Cannabis:Herb.THC	-15.227	2.728	3.10E-08	6.66E-06	1.39E+10	-
Myeloma	LM.Cannabis:Herb.THC	-6.157	1.040	5.19E-09	1.16E-06	6.86E+10	-
Hodgkins	LM.Cannabis:Herb.THC	-7.089	1.190	4.14E-09	9.33E-07	8.04E+10	-
Brain	Herb.THC	-12.314	4.616	7.77E-03	0.9474173	9.48E+11	-
Oropharynx	LM.Cannabis	-4.690	0.836	2.23E-07	4.63E-05	1.93E+13	-
Gallbladder & Biliary	LM.Cannabis:Herb.THC	-9.674	1.355	2.69E-12	6.50E-10	9.00E+13	-
All Cancers	LM.Cannabis:Herb.THC	-7.077	2.573	6.19E-03	0.7924609	1.92E+14	-
Thyroid	LM.Cannabis:Herb.THC	-13.551	1.530	7.57E-18	2.01E-15	1.15E+16	-

Oropharynx_Broad	LM.Cannabis:Herb.THC	-15.619	17.713	3.78E-01	1	6.87E+16	-
Colorectum	LM.Cannabis:Herb.THC	-35.451	3.483	3.48E-23	9.61E-21	1.24E+18	-
Kaposi	Tobacco:LM.Cannabis	-29.221	45.471	5.24E-01	1	6.49E+58	-
Oropharynx	Herb.THC	-48.615	6.665	1.19E-10	2.79E-08	7.94E+134	-

Supplementary Table S32.: Significant Positive Terms from

Interactive Panel Regression at Four Temporal Lags

Cancer	Term	ß-Estimate	Std. Error	P_Value	P.Adj.Holm	Adj.P.FDR	E-Value Estimate	E-Value 95% Lower Bound
Oropharynx	LM.Cannabis:Herb.THC	34.320	7.372	1.12E-05	2.13E-03	1.12E-05	2.11E+95	2.49E+55
Colorectum	Herb.THC	92.213	8.721	8.28E-25	2.31E-22	8.28E-25	3.79E+46	1.04E+38
Gallbladder & Biliary	Herb.THC	29.693	3.555	4.60E-16	1.19E-13	4.60E-16	1.61E+42	2.47E+32
Thyroid	Herb.THC	33.741	4.103	1.03E-15	2.63E-13	1.03E-15	3.50E+39	1.62E+30
Breast	Herb.THC	51.744	6.784	5.76E-14	1.43E-11	5.76E-14	5.52E+33	1.47E+25
Anus	Herb.THC	24.891	3.505	3.19E-12	7.62E-10	3.19E-12	1.56E+34	7.22E+24
Oropharynx	Resin.THC	9.947	1.105	3.68E-14	9.19E-12	3.68E-14	6.95E+27	7.23E+21
Oesophagus	Herb.THC	31.178	5.101	1.42E-09	3.25E-07	1.42E-09	1.12E+27	3.09E+18
Myeloma	Herb.THC	15.860	2.788	1.93E-08	4.18E-06	1.93E-08	2.76E+27	1.28E+18
All Cancers	Herb.THC	13.310	2.871	4.70E-06	9.20E-04	4.70E-06	3.99E+26	3.19E+15
Melanoma	Herb.THC	24.578	5.069	1.45E-06	2.91E-04	1.45E-06	3.61E+21	9.59E+12
Liver	Herb.THC	19.895	4.541	1.38E-05	2.58E-03	1.38E-05	9.79E+21	2.02E+12
Hodgkins	Herb.THC	13.432	3.190	2.89E-05	5.29E-03	2.89E-05	2.48E+20	1.15E+11
Testis	Herb.THC	45.669	10.959	3.49E-05	6.28E-03	3.49E-05	1.53E+20	7.12E+10
Oropharynx_Broad	Herb.THC	21.554	7.876	6.52E-03	8.28E-01	6.52E-03	1.32E+23	6.45E+06
Bladder	Herb.THC	13.187	4.024	1.09E-03	1.68E-01	1.09E-03	4.41E+14	1.21E+06
Oropharynx	Tobacco:Herb.THC	1.120	0.180	1.69E-08	3.69E-06	1.69E-08	2.52E+03	2.65E+02
Stomach	Herb.THC	17.035	7.068	1.61E-02	1.00E+00	1.61E-02	7.08E+10	1.94E+02
Oropharynx_Broad	LM.Cannabis	6.254	2.287	6.55E-03	8.28E-01	6.55E-03	8.37E+06	1.53E+02
Oesophagus	Resin.THC	1.932	0.206	5.48E-20	1.47E-17	5.48E-20	9.03E+01	4.04E+01
Colorectum	LM.Cannabis	2.338	0.184	2.13E-34	6.15E-32	2.13E-34	2.93E+01	1.92E+01

Thyroid	LM.Cannabis	0.980	0.087	3.18E-27	9.00E-25	3.18E-27	2.71E+01	1.70E+01
Myeloma	Resin.THC	0.719	0.116	9.36E-10	2.16E-07	9.36E-10	3.35E+01	1.34E+01
Larynx	Resin.THC	1.162	0.197	5.11E-09	1.14E-06	5.11E-09	2.16E+01	9.45E+00
Stomach	LM.Cannabis	1.329	0.149	2.34E-18	6.22E-16	2.34E-18	1.28E+01	8.25E+00
Hodgkins	LM.Cannabis	0.553	0.067	1.24E-15	3.16E-13	1.24E-15	1.29E+01	7.99E+00
Larynx	LM.Cannabis	0.868	0.103	1.04E-16	2.70E-14	1.04E-16	1.15E+01	7.42E+00
Cervix	LM.Cannabis:Herb.THC	8.526	3.781	2.44E-02	1.00E+00	2.44E-02	1.75E+04	6.12E+00
Breast	LM.Cannabis	1.063	0.144	2.98E-13	7.31E-11	2.98E-13	9.20E+00	5.86E+00
Thyroid	Tobacco:LM.Cannabis:Herb.THC	0.505	0.050	9.13E-23	2.50E-20	9.13E-23	7.19E+00	5.41E+00
Colorectum	Tobacco:LM.Cannabis:Herb.THC	1.066	0.113	3.06E-20	8.30E-18	3.06E-20	6.31E+00	4.75E+00
Gallbladder & Biliary	Tobacco:LM.Cannabis:Herb.THC	0.312	0.044	2.95E-12	7.07E-10	2.95E-12	4.95E+00	3.59E+00
Myeloma	LM.Cannabis	0.297	0.059	6.19E-07	1.26E-04	6.19E-07	5.89E+00	3.50E+00
Oropharynx	Tobacco:LM.Cannabis	0.157	0.024	6.02E-09	1.34E-06	6.02E-09	4.89E+00	3.43E+00
Hodgkins	Resin.THC	0.459	0.132	5.58E-04	8.99E-02	5.58E-04	9.20E+00	3.40E+00
Leukaemia - Myeloid	Alcohol	0.269	0.066	8.04E-05	1.40E-02	8.04E-05	6.31E+00	3.21E+00
Hodgkins	Tobacco:LM.Cannabis:Herb.THC	0.236	0.038	1.52E-09	3.45E-07	1.52E-09	3.93E+00	2.87E+00
Leukaemia - Lymphoid	Alcohol	0.119	0.032	2.61E-04	4.36E-02	2.61E-04	5.67E+00	2.84E+00
Anus	Tobacco:LM.Cannabis:Herb.THC	0.247	0.042	7.88E-09	1.73E-06	7.88E-09	3.77E+00	2.74E+00
Colorectum	Resin.THC	1.124	0.352	1.47E-03	2.23E-01	1.47E-03	6.79E+00	2.69E+00
Myeloma	Tobacco:LM.Cannabis:Herb.THC	0.190	0.034	2.58E-08	5.56E-06	2.58E-08	3.64E+00	2.64E+00
Breast	Tobacco:LM.Cannabis:Herb.THC	0.494	0.089	3.60E-08	7.71E-06	3.60E-08	3.59E+00	2.60E+00
Lung	Resin.THC	0.350	0.116	2.49E-03	3.62E-01	2.49E-03	6.33E+00	2.47E+00
All Cancers	Tobacco:LM.Cannabis:Herb.THC	0.253	0.081	1.92E-03	2.85E-01	1.92E-03	5.77E+00	2.44E+00
Testis	LM.Cannabis	0.809	0.232	5.12E-04	8.30E-02	5.12E-04	3.93E+00	2.21E+00
Gallbladder & Biliary	LM.Cannabis	0.261	0.079	9.97E-04	1.57E-01	9.97E-04	4.09E+00	2.18E+00
Liver	Tobacco:LM.Cannabis:Herb.THC	0.230	0.054	2.17E-05	3.99E-03	2.17E-05	2.96E+00	2.08E+00
Oesophagus	Tobacco:LM.Cannabis:Herb.THC	0.274	0.066	3.76E-05	6.70E-03	3.76E-05	2.83E+00	1.99E+00
Gallbladder & Biliary	Tobacco	0.102	0.008	4.70E-36	1.36E-33	4.70E-36	2.13E+00	1.99E+00
Brain	Tobacco:Herb.THC	0.419	0.148	4.73E-03	6.34E-01	4.73E-03	4.43E+00	1.99E+00

Testis	Income	0.520	0.133	1.05E-04	1.81E-02	1.05E-04	2.76E+00	1.92E+00
Corpus Uteri	LM.Cannabis	0.587	0.198	3.14E-03	4.40E-01	3.14E-03	3.16E+00	1.78E+00
Larynx	Alcohol	0.128	0.015	1.22E-17	3.22E-15	1.22E-17	1.93E+00	1.76E+00
Cervix	Tobacco:Herb.THC	0.785	0.304	9.85E-03	1.00E+00	9.85E-03	4.04E+00	1.75E+00
Brain	Income	0.161	0.041	8.51E-05	1.47E-02	8.51E-05	2.19E+00	1.67E+00
All Cancers	Alcohol	0.061	0.013	5.80E-06	1.13E-03	5.80E-06	1.96E+00	1.62E+00
Breast	Income	0.218	0.060	2.97E-04	4.89E-02	2.97E-04	2.11E+00	1.59E+00
Stomach	Tobacco:LM.Cannabis:Herb.THC	0.283	0.092	2.06E-03	3.03E-01	2.06E-03	2.36E+00	1.59E+00
Colorectum	Tobacco	0.147	0.012	8.63E-31	2.49E-28	8.63E-31	1.65E+00	1.57E+00
Testis	Tobacco:LM.Cannabis:Herb.THC	0.396	0.132	2.84E-03	4.05E-01	2.84E-03	2.34E+00	1.56E+00
Breast	Tobacco	0.097	0.010	4.73E-23	1.30E-20	4.73E-23	1.58E+00	1.50E+00
Melanoma	Tobacco:LM.Cannabis:Herb.THC	0.185	0.066	5.49E-03	7.19E-01	5.49E-03	2.25E+00	1.47E+00
Stomach	Tobacco	0.078	0.010	1.61E-14	4.04E-12	1.61E-14	1.48E+00	1.39E+00
Oropharynx_Broad	Tobacco	0.098	0.036	5.89E-03	7.60E-01	5.89E-03	1.86E+00	1.35E+00
Colorectum	Alcohol	0.109	0.026	3.19E-05	5.81E-03	3.19E-05	1.53E+00	1.34E+00
Prostate	Income	0.232	0.087	7.59E-03	9.33E-01	7.59E-03	1.85E+00	1.33E+00
Testis	Tobacco	0.087	0.018	9.88E-07	1.99E-04	9.88E-07	1.41E+00	1.29E+00
Myeloma	Income	0.079	0.034	1.98E-02	1.00E+00	1.98E-02	2.07E+00	1.29E+00
Hodgkins	Alcohol	0.035	0.011	1.02E-03	1.59E-01	1.02E-03	1.51E+00	1.28E+00
All Cancers nNMSC	Tobacco:LM.Cannabis	0.012	0.002	7.07E-07	1.43E-04	7.07E-07	1.37E+00	1.27E+00
Corpus Uteri	Tobacco	0.069	0.013	3.00E-07	6.21E-05	3.00E-07	1.36E+00	1.27E+00
Larynx	Tobacco:LM.Cannabis:Herb.THC	0.146	0.063	2.14E-02	1.00E+00	2.14E-02	2.04E+00	1.27E+00
Thyroid	Alcohol	0.042	0.014	2.57E-03	3.70E-01	2.57E-03	1.48E+00	1.24E+00
Stomach	Alcohol	0.068	0.021	1.39E-03	2.11E-01	1.39E-03	1.44E+00	1.24E+00
Prostate	Tobacco	0.061	0.014	1.14E-05	2.16E-03	1.14E-05	1.33E+00	1.23E+00
Ovary	Tobacco	0.047	0.011	2.03E-05	3.75E-03	2.03E-05	1.32E+00	1.22E+00
Pancreas	Tobacco:LM.Cannabis	0.016	0.003	1.32E-09	3.01E-07	1.32E-09	1.26E+00	1.20E+00
Lung	Tobacco:LM.Cannabis	0.011	0.002	6.83E-09	1.51E-06	6.83E-09	1.25E+00	1.19E+00
Oesophagus	Alcohol	0.043	0.015	4.75E-03	6.34E-01	4.75E-03	1.40E+00	1.19E+00

All Cancers nNMSC	Tobacco:Herb.THC	0.213	0.107	4.65E-02	1.00E+00	4.65E-02	7.37E+00	1.19E+00
Kidney	Tobacco:LM.Cannabis	0.014	0.003	2.20E-07	4.60E-05	2.20E-07	1.23E+00	1.18E+00
Breast	Alcohol	0.055	0.020	7.36E-03	9.13E-01	7.36E-03	1.39E+00	1.17E+00
Bladder	Tobacco	0.020	0.006	6.23E-04	9.97E-02	6.23E-04	1.28E+00	1.17E+00
Melanoma	Tobacco:LM.Cannabis	0.016	0.003	5.53E-06	1.08E-03	5.53E-06	1.21E+00	1.15E+00
Liver	Tobacco:LM.Cannabis	0.012	0.003	3.71E-05	6.63E-03	3.71E-05	1.21E+00	1.15E+00
Larynx	Tobacco	0.020	0.007	3.22E-03	4.47E-01	3.22E-03	1.25E+00	1.13E+00
Thyroid	Tobacco	0.018	0.007	7.95E-03	9.62E-01	7.95E-03	1.27E+00	1.13E+00
Oesophagus	Tobacco:LM.Cannabis	0.011	0.003	1.81E-03	2.72E-01	1.81E-03	1.17E+00	1.10E+00
Non-Hodgkins lymphoma	Tobacco:LM.Cannabis	0.012	0.004	2.83E-03	4.05E-01	2.83E-03	1.17E+00	1.09E+00
Kidney	Tobacco:LM.Cannabis:Herb.THC	0.102	0.052	4.97E-02	1.00E+00	4.97E-02	1.91E+00	1.04E+00

Supplementary Table S33.: Summary of Significant Positive Terms from

Interactive Panel Regression at Four Temporal Lags

Term	Count	Negative Total of P-Value Exponents	Mean of the Negative P-Value Exponents	Median of the Negative P-Value Exponents	Total of the Lower E-Value Exponents	Mean of the Lower E-Value Exponents	Median of the Lower E-Value Exponents
Herb.THC	15	119	7.93	5	259	17.27	15
LM.Cannabis : Herb.THC	2	5	2.50	2.5	55	27.50	27.5
Resin.THC	7	56	8	8.00	23	3.29	0
Last.Month.Cannabis	11	133	12.09	12	4	0.36	0
Tobacco:Herb.THC	4	12	3	2	2	0.5	0
Alcohol	10	42	4.2	2.5	0	0	0
Income	5	13	2.60	3	0	0	0
Tobacco	12	130	10.83	5	0	0	0
Tobacco:LM.Cannabis	9	49	5.44	6	0	0	0
Tobacco:LM.Cannabis:Herb.THC	15	100	6.67	4	0	0	0

Supplementary Table S34.: Complete Model Output from

Interactive Panel Regression at Six Temporal Lags

Cancer	Term	ß-Estimate	Std. Error	P-Value	E-Value Estimate	95% Lower Bound of the E-Value
Thyroid	Herb.THC	42.032	5.452	7.28E-14	3.50E+48	2.01E+36
Anus	Herb.THC	33.645	4.821	9.88E-12	9.40E+43	5.41E+31
Colorectum	Herb.THC	89.627	11.695	5.57E-14	2.28E+42	4.21E+31
Oropharynx	LM.Cannabis:Herb.THC	26.474	9.156	5.01E-03	2.87E+74	1.97E+24
Melanoma	Herb.THC	35.988	6.202	9.69E-09	1.67E+32	2.90E+21
Gallbladder & Biliary	Herb.THC	25.697	5.031	4.92E-07	1.31E+34	1.47E+21
Breast	Herb.THC	49.072	9.167	1.15E-07	5.63E+29	9.77E+18
Oropharynx	Resin.THC	8.136	1.419	1.95E-07	1.23E+23	2.07E+15
All Cancers nNMSC	Herb.THC	12.447	4.357	4.42E-03	8.78E+39	6.30E+12
Oropharynx_Broad	Herb.THC	28.761	9.112	1.75E-03	3.89E+32	3.81E+12
Oropharynx_Broad	LM.Cannabis	15.402	3.351	6.26E-06	3.91E+17	1.71E+10
Testis	Herb.THC	52.101	15.980	1.19E-03	5.05E+20	2.91E+08
Oesophagus	Herb.THC	21.875	6.577	9.24E-04	3.58E+18	6.60E+07
Myeloma	Herb.THC	10.996	3.763	3.64E-03	3.85E+18	2.22E+06
Cervix	LM.Cannabis:Herb.THC	17.937	4.974	3.32E-04	1.51E+08	8.04E+03
Hodgkins	Herb.THC	10.998	4.384	1.24E-02	9.99E+15	5.75E+03
Bladder	Herb.THC	13.125	5.195	1.17E-02	1.46E+14	2.70E+03
Oropharynx	Tobacco:Herb.THC	1.255	0.222	2.66E-07	6.55E+03	3.98E+02
Brain	LM.Cannabis:Herb.THC	6.950	2.367	3.43E-03	5.19E+06	2.76E+02
Thyroid	LM.Cannabis	1.312	0.121	1.25E-24	6.36E+01	3.38E+01
Oesophagus	Resin.THC	1.944	0.250	2.30E-14	8.32E+01	3.23E+01

Colorectum	LM.Cannabis	2.454	0.250	1.85E-21	2.78E+01	1.62E+01
Myeloma	Resin.THC	0.779	0.146	1.55E-07	3.90E+01	1.27E+01
Larynx	LM.Cannabis	1.046	0.137	5.66E-14	1.53E+01	8.82E+00
Thyroid	Tobacco:LM.Cannabis:Herb.THC	0.668	0.066	6.12E-22	1.12E+01	7.78E+00
Larynx	Resin.THC	1.139	0.242	3.07E-06	1.86E+01	6.92E+00
Pancreas	LM.Cannabis:Herb.THC	4.265	1.953	2.93E-02	1.18E+05	5.73E+00
Breast	LM.Cannabis	1.157	0.197	6.93E-09	9.36E+00	5.25E+00
Hodgkins	LM.Cannabis	0.514	0.097	1.97E-07	1.03E+01	5.24E+00
Colorectum	Tobacco:LM.Cannabis:Herb.THC	1.150	0.155	3.59E-13	6.39E+00	4.43E+00
Cervix	Tobacco:Herb.THC	1.692	0.407	3.64E-05	1.05E+01	4.38E+00
Ovary	LM.Cannabis:Herb.THC	8.697	4.082	3.34E-02	9.04E+04	4.25E+00
Stomach	LM.Cannabis	0.980	0.196	6.90E-07	7.20E+00	3.99E+00
Anus	Tobacco:LM.Cannabis:Herb.THC	0.348	0.058	4.81E-09	5.11E+00	3.44E+00
Brain	Tobacco:Herb.THC	0.708	0.194	2.76E-04	8.48E+00	3.44E+00
Lung	Resin.THC	0.445	0.135	1.06E-03	9.15E+00	3.20E+00
Ovary	Tobacco:Herb.THC	1.123	0.334	8.13E-04	7.45E+00	2.97E+00
Oropharynx_Broad	Tobacco:LM.Cannabis:Herb.THC	1.495	0.655	2.30E-02	9.49E+01	2.88E+00
Lung	Tobacco:Herb.THC	0.381	0.116	1.09E-03	7.18E+00	2.84E+00
All Cancers nNMSC	Tobacco:LM.Cannabis:Herb.THC	0.182	0.055	1.02E-03	7.06E+00	2.84E+00
Hodgkins	Resin.THC	0.498	0.170	3.67E-03	9.74E+00	2.82E+00
Oropharynx_Broad	Alcohol	0.499	0.149	9.17E-04	6.72E+00	2.81E+00
Oropharynx	Tobacco:LM.Cannabis	0.133	0.031	6.84E-05	4.14E+00	2.54E+00
Myeloma	LM.Cannabis	0.280	0.084	8.81E-04	5.29E+00	2.50E+00
Leukaemia - Lymphoid	Alcohol	0.125	0.041	2.71E-03	6.20E+00	2.49E+00
Leukaemia - Myeloid	Alcohol	0.262	0.086	2.86E-03	6.15E+00	2.46E+00
Kidney	Tobacco:Herb.THC	0.490	0.164	2.89E-03	6.30E+00	2.43E+00
Breast	Tobacco:LM.Cannabis:Herb.THC	0.538	0.124	1.51E-05	3.63E+00	2.38E+00
Hodgkins	Tobacco:LM.Cannabis:Herb.THC	0.228	0.053	2.19E-05	3.65E+00	2.37E+00
Pancreas	Tobacco:Herb.THC	0.456	0.160	4.50E-03	5.92E+00	2.25E+00
Myeloma	Tobacco:LM.Cannabis:Herb.THC	0.185	0.046	6.03E-05	3.47E+00	2.24E+00

Gallbladder & Biliary	Tobacco:LM.Cannabis:Herb.THC	0.238	0.065	2.67E-04	3.53E+00	2.15E+00
Testis	LM.Cannabis	1.066	0.355	2.81E-03	4.67E+00	2.14E+00
Gallbladder & Biliary	Tobacco	0.098	0.011	1.14E-17	2.03E+00	1.84E+00
Larynx	Alcohol	0.148	0.019	7.76E-15	2.01E+00	1.80E+00
Testis	Income	0.597	0.183	1.16E-03	2.82E+00	1.79E+00
Stomach	Tobacco:Herb.THC	0.737	0.298	1.37E-02	4.98E+00	1.78E+00
Oropharynx_Broad	Tobacco	0.166	0.044	2.23E-04	2.44E+00	1.75E+00
All Cancers	Tobacco:LM.Cannabis:Herb.THC	0.253	0.107	1.85E-02	5.92E+00	1.75E+00
Colorectum	Resin.THC	1.055	0.444	1.77E-02	5.70E+00	1.75E+00
Larynx	Tobacco:LM.Cannabis:Herb.THC	0.257	0.085	2.55E-03	2.71E+00	1.68E+00
Melanoma	Tobacco:LM.Cannabis:Herb.THC	0.233	0.084	5.53E-03	2.60E+00	1.57E+00
Colorectum	Tobacco	0.157	0.017	1.40E-18	1.65E+00	1.54E+00
Oesophagus	Tobacco:LM.Cannabis:Herb.THC	0.236	0.087	7.05E-03	2.52E+00	1.52E+00
Testis	Tobacco:LM.Cannabis:Herb.THC	0.504	0.194	9.60E-03	2.53E+00	1.48E+00
Breast	Income	0.225	0.075	2.64E-03	2.07E+00	1.47E+00
Breast	Tobacco	0.102	0.014	1.83E-13	1.57E+00	1.46E+00
Thyroid	Alcohol	0.074	0.018	3.88E-05	1.73E+00	1.46E+00
Oropharynx_Broad	Income	0.184	0.074	1.34E-02	2.60E+00	1.45E+00
All Cancers	Alcohol	0.048	0.015	2.12E-03	1.80E+00	1.39E+00
Bladder	Resin.THC	0.414	0.197	3.61E-02	4.92E+00	1.35E+00
All Cancers nNMSC	Tobacco	0.021	0.006	9.66E-04	1.60E+00	1.33E+00
Thyroid	Tobacco	0.036	0.009	9.34E-05	1.43E+00	1.27E+00
Testis	Tobacco	0.104	0.027	1.12E-04	1.43E+00	1.27E+00
Pancreas	Tobacco:LM.Cannabis	0.025	0.003	2.58E-12	1.33E+00	1.27E+00
Colorectum	Alcohol	0.102	0.034	2.74E-03	1.48E+00	1.24E+00
Kidney	Tobacco:LM.Cannabis	0.021	0.004	2.92E-09	1.30E+00	1.23E+00
Lung	Tobacco:LM.Cannabis	0.015	0.003	4.98E-09	1.29E+00	1.23E+00
Stomach	Tobacco	0.050	0.014	2.47E-04	1.35E+00	1.22E+00
Bladder	Tobacco	0.028	0.008	2.71E-04	1.35E+00	1.22E+00
Hodgkins	Alcohol	0.037	0.014	1.01E-02	1.51E+00	1.21E+00

Liver	Tobacco:LM.Cannabis:Herb.THC	0.159	0.075	3.46E-02	2.28E+00	1.20E+00
Liver	Tobacco:LM.Cannabis	0.018	0.004	5.02E-05	1.26E+00	1.17E+00
Melanoma	Tobacco	0.028	0.009	2.12E-03	1.31E+00	1.17E+00
Prostate	Tobacco:Herb.THC	0.858	0.430	4.61E-02	3.98E+00	1.15E+00
Larynx	Tobacco	0.027	0.009	4.25E-03	1.30E+00	1.15E+00
Anus	Tobacco	0.020	0.008	1.14E-02	1.32E+00	1.13E+00
Corpus Uteri	Tobacco	0.050	0.019	8.37E-03	1.28E+00	1.13E+00
Brain	Tobacco:LM.Cannabis	0.014	0.004	1.02E-03	1.20E+00	1.12E+00
Melanoma	Tobacco:LM.Cannabis	0.014	0.004	1.60E-03	1.20E+00	1.12E+00
Brain	Income	0.099	0.048	3.99E-02	1.77E+00	1.11E+00
Oesophagus	Tobacco:LM.Cannabis	0.014	0.005	2.96E-03	1.19E+00	1.11E+00
Non-Hodgkins lymphoma	Tobacco:LM.Cannabis	0.015	0.005	3.83E-03	1.19E+00	1.10E+00
Cervix	Tobacco:LM.Cannabis	0.022	0.009	1.08E-02	1.18E+00	1.08E+00
Stomach	Alcohol	0.055	0.027	4.06E-02	1.37E+00	1.06E+00
Prostate	Tobacco	0.040	0.020	4.05E-02	1.24E+00	1.04E+00
Prostate	Tobacco:LM.Cannabis	0.002	0.009	8.33E-01	1.05E+00	1.00E+00
Prostate	Alcohol	0.003	0.038	9.28E-01	1.06E+00	1.00E+00
Ovary	Tobacco:LM.Cannabis	0.007	0.007	3.50E-01	1.10E+00	1.00E+00
Hodgkins	Tobacco	0.003	0.007	6.99E-01	1.11E+00	1.00E+00
Stomach	Tobacco:LM.Cannabis:Herb.THC	0.010	0.122	9.32E-01	1.14E+00	1.00E+00
Cervix	Alcohol	0.020	0.036	5.83E-01	1.16E+00	1.00E+00
Ovary	Tobacco	0.025	0.015	1.02E-01	1.21E+00	1.00E+00
Non-Hodgkins lymphoma	Tobacco:LM.Cannabis:Herb.THC	0.019	0.104	8.54E-01	1.22E+00	1.00E+00
Myeloma	Alcohol	0.010	0.012	4.09E-01	1.24E+00	1.00E+00
Myeloma	Income	0.012	0.043	7.83E-01	1.27E+00	1.00E+00
Bladder	Tobacco:LM.Cannabis:Herb.THC	0.020	0.069	7.72E-01	1.28E+00	1.00E+00
Testis	Alcohol	0.059	0.052	2.62E-01	1.29E+00	1.00E+00
Oesophagus	Alcohol	0.028	0.019	1.48E-01	1.30E+00	1.00E+00
Breast	Alcohol	0.050	0.027	6.29E-02	1.35E+00	1.00E+00
Anus	LM.Cannabis	0.035	0.107	7.44E-01	1.46E+00	1.00E+00

Prostate	Income	0.111	0.107	3.00E-01	1.47E+00	1.00E+00
Bladder	LM.Cannabis	0.059	0.111	5.93E-01	1.58E+00	1.00E+00
All Cancers nNMSC	LM.Cannabis	0.027	0.108	8.05E-01	1.73E+00	1.00E+00
Corpus Uteri	LM.Cannabis	0.257	0.271	3.43E-01	1.91E+00	1.00E+00
Leukaemia - Lymphoid	Tobacco:Herb.THC	0.040	0.136	7.67E-01	2.32E+00	1.00E+00
Leukaemia - Myeloid	Tobacco:LM.Cannabis:Herb.THC	0.090	0.280	7.50E-01	2.39E+00	1.00E+00
Leukaemia - Lymphoid	LM.Cannabis	0.047	0.550	9.32E-01	2.54E+00	1.00E+00
Non-Hodgkins lymphoma	Resin.THC	0.317	0.289	2.73E-01	2.85E+00	1.00E+00
Gallbladder & Biliary	LM.Cannabis	0.188	0.121	1.21E-01	2.94E+00	1.00E+00
Non-Hodgkins lymphoma	Tobacco:Herb.THC	0.390	0.242	1.08E-01	3.33E+00	1.00E+00
Corpus Uteri	Tobacco:Herb.THC	0.702	0.413	8.94E-02	3.44E+00	1.00E+00
Leukaemia - Myeloid	Income	0.303	0.382	4.29E-01	7.56E+00	1.00E+00
All Cancers nNMSC	Resin.THC	0.210	0.153	1.72E-01	8.79E+00	1.00E+00
All Cancers	LM.Cannabis	0.365	0.389	3.48E-01	1.04E+01	1.00E+00
Leukaemia - Myeloid	LM.Cannabis	0.800	1.165	4.94E-01	7.91E+01	1.00E+00
Corpus Uteri	LM.Cannabis:Herb.THC	3.697	5.041	4.64E-01	7.95E+01	1.00E+00
Prostate	LM.Cannabis:Herb.THC	4.989	5.248	3.42E-01	2.38E+02	1.00E+00
Oropharynx_Broad	Resin.THC	2.208	1.269	8.29E-02	6.02E+02	1.00E+00
Kidney	LM.Cannabis:Herb.THC	2.510	2.004	2.11E-01	1.09E+03	1.00E+00
Lung	LM.Cannabis:Herb.THC	1.929	1.421	1.75E-01	1.85E+03	1.00E+00
Leukaemia - Myeloid	Herb.THC	2.045	9.817	8.35E-01	2.47E+04	1.00E+00
Kaposi	Tobacco:LM.Cannabis	3.268	101.245	9.74E-01	3.80E+07	1.00E+00
Larynx	Herb.THC	11.854	6.383	6.37E-02	3.11E+10	1.00E+00
All Cancers	Herb.THC	5.948	3.590	9.84E-02	1.97E+12	1.00E+00
Liver	Herb.THC	11.759	6.311	6.30E-02	3.00E+12	1.00E+00
Leukaemia - Lymphoid	LM.Cannabis:Herb.THC	4.085	4.542	3.71E-01	3.98E+17	1.00E+00
Bladder	Tobacco:LM.Cannabis	0.000	0.004	9.04E-01	1.03E+00	-
All Cancers nNMSC	Tobacco:LM.Cannabis	-0.001	0.003	8.73E-01	1.07E+00	-
Anus	Tobacco:LM.Cannabis	-0.002	0.003	5.81E-01	1.08E+00	-
Brain	Tobacco	-0.004	0.009	6.77E-01	1.10E+00	-

Corpus Uteri	Tobacco:LM.Cannabis	-0.009	0.009	2.93E-01	1.11E+00	-
Leukaemia - Lymphoid	Tobacco:LM.Cannabis	-0.002	0.016	9.20E-01	1.14E+00	-
Hodgkins	Income	-0.006	0.050	9.04E-01	1.16E+00	-
Leukaemia - Lymphoid	Tobacco	-0.002	0.013	8.72E-01	1.17E+00	-
Oesophagus	Tobacco	-0.013	0.010	1.88E-01	1.19E+00	-
Myeloma	Tobacco:LM.Cannabis	-0.007	0.003	1.05E-02	1.19E+00	-
Corpus Uteri	Alcohol	-0.027	0.037	4.64E-01	1.19E+00	-
All Cancers	Tobacco	-0.006	0.006	2.57E-01	1.21E+00	-
Testis	Tobacco:LM.Cannabis	-0.035	0.011	2.34E-03	1.21E+00	-
Gallbladder & Biliary	Tobacco:LM.Cannabis	-0.011	0.004	3.71E-03	1.22E+00	-
Leukaemia - Myeloid	Tobacco	-0.008	0.028	7.83E-01	1.23E+00	-
Cervix	Tobacco	-0.035	0.019	5.92E-02	1.23E+00	-
Kidney	Tobacco	-0.015	0.007	5.09E-02	1.23E+00	-
Thyroid	Income	-0.014	0.062	8.22E-01	1.24E+00	-
Pancreas	Tobacco	-0.015	0.007	4.29E-02	1.24E+00	-
Myeloma	Tobacco	-0.010	0.006	1.10E-01	1.24E+00	-
Stomach	Tobacco:LM.Cannabis	-0.031	0.006	2.11E-06	1.26E+00	-
Hodgkins	Tobacco:LM.Cannabis	-0.015	0.003	2.99E-06	1.28E+00	-
Breast	Tobacco:LM.Cannabis	-0.036	0.006	2.69E-08	1.28E+00	-
Gallbladder & Biliary	Income	-0.018	0.057	7.54E-01	1.30E+00	-
Larynx	Tobacco:LM.Cannabis	-0.029	0.004	1.89E-10	1.31E+00	-
Lung	Tobacco	-0.017	0.005	1.42E-03	1.32E+00	-
All Cancers	Tobacco:LM.Cannabis	-0.014	0.012	2.34E-01	1.34E+00	-
Colorectum	Tobacco:LM.Cannabis	-0.073	0.008	3.47E-18	1.38E+00	-
Ovary	Alcohol	-0.068	0.030	2.31E-02	1.40E+00	-
Non-Hodgkins lymphoma	Tobacco	-0.050	0.011	7.23E-06	1.40E+00	-
Liver	Tobacco	-0.039	0.010	1.67E-04	1.42E+00	-
Prostate	LM.Cannabis	-0.104	0.282	7.13E-01	1.44E+00	-
Corpus Uteri	Income	-0.104	0.103	3.10E-01	1.46E+00	-
Thyroid	Tobacco:LM.Cannabis	-0.040	0.004	9.06E-23	1.47E+00	-

Leukaemia - Myeloid	Tobacco:LM.Cannabis	-0.028	0.034	4.10E-01	1.54E+00	-
Anus	Alcohol	-0.044	0.016	5.65E-03	1.54E+00	-
Non-Hodgkins lymphoma	Alcohol	-0.081	0.022	2.08E-04	1.56E+00	-
Corpus Uteri	Tobacco:LM.Cannabis:Herb.THC	-0.144	0.168	3.93E-01	1.58E+00	-
Kidney	Tobacco:LM.Cannabis:Herb.THC	-0.057	0.067	3.93E-01	1.58E+00	-
Prostate	Tobacco:LM.Cannabis:Herb.THC	-0.170	0.175	3.33E-01	1.63E+00	-
Lung	Alcohol	-0.047	0.010	7.81E-06	1.64E+00	-
Brain	Alcohol	-0.083	0.017	1.98E-06	1.67E+00	-
Ovary	Income	-0.147	0.083	7.79E-02	1.69E+00	-
Lung	Tobacco:LM.Cannabis:Herb.THC	-0.051	0.047	2.79E-01	1.69E+00	-
Colorectum	Income	-0.177	0.095	6.34E-02	1.71E+00	-
Leukaemia - Myeloid	Tobacco:Herb.THC	-0.049	0.287	8.65E-01	1.82E+00	-
Oropharynx	Tobacco	-0.036	0.021	8.90E-02	1.84E+00	-
Melanoma	Alcohol	-0.141	0.018	1.91E-14	2.00E+00	-
Kidney	Alcohol	-0.125	0.015	9.01E-17	2.08E+00	-
Melanoma	Resin.THC	-0.155	0.237	5.14E-01	2.09E+00	-
Bladder	Alcohol	-0.131	0.015	2.63E-17	2.10E+00	-
Anus	Income	-0.107	0.055	5.29E-02	2.10E+00	-
Cervix	Income	-0.323	0.102	1.55E-03	2.12E+00	-
Pancreas	Tobacco:LM.Cannabis:Herb.THC	-0.129	0.065	4.77E-02	2.14E+00	-
Ovary	LM.Cannabis	-0.274	0.219	2.12E-01	2.15E+00	-
All Cancers	Tobacco:Herb.THC	-0.073	0.120	5.43E-01	2.16E+00	-
Non-Hodgkins lymphoma	LM.Cannabis:Herb.THC	-0.204	3.069	9.47E-01	2.19E+00	-
Liver	Alcohol	-0.151	0.020	1.82E-13	2.22E+00	-
Ovary	Tobacco:LM.Cannabis:Herb.THC	-0.296	0.136	2.99E-02	2.24E+00	-
Pancreas	Alcohol	-0.147	0.014	2.37E-23	2.28E+00	-
Larynx	Income	-0.195	0.052	1.81E-04	2.30E+00	-
Oropharynx	Alcohol	-0.069	0.081	3.92E-01	2.51E+00	-
All Cancers nNMSC	Alcohol	-0.061	0.010	2.28E-09	2.51E+00	-
Brain	Tobacco:LM.Cannabis:Herb.THC	-0.214	0.079	7.02E-03	2.52E+00	-

Gallbladder & Biliary	Alcohol	-0.155	0.020	6.45E-14	2.57E+00	-
Melanoma	Income	-0.272	0.050	9.96E-08	2.88E+00	-
Cervix	Tobacco:LM.Cannabis:Herb.THC	-0.573	0.166	5.87E-04	2.97E+00	-
Larynx	Tobacco:Herb.THC	-0.300	0.208	1.50E-01	3.02E+00	-
Breast	Resin.THC	-0.434	0.351	2.16E-01	3.05E+00	-
Liver	Tobacco:Herb.THC	-0.264	0.216	2.23E-01	3.16E+00	-
Liver	Income	-0.276	0.069	8.05E-05	3.27E+00	-
Bladder	Tobacco:Herb.THC	-0.274	0.169	1.06E-01	3.31E+00	-
Oesophagus	Income	-0.389	0.053	8.74E-13	3.64E+00	-
Stomach	Income	-0.577	0.074	2.80E-14	3.86E+00	-
Oesophagus	LM.Cannabis	-0.427	0.141	2.51E-03	3.97E+00	-
Cervix	LM.Cannabis	-0.843	0.267	1.68E-03	4.12E+00	-
Non-Hodgkins lymphoma	Income	-0.495	0.060	1.28E-15	4.12E+00	-
Non-Hodgkins lymphoma	LM.Cannabis	-0.496	0.162	2.30E-03	4.14E+00	-
Testis	Resin.THC	-0.988	0.621	1.12E-01	4.31E+00	-
Bladder	Income	-0.386	0.042	5.57E-19	4.55E+00	-
Myeloma	Tobacco:Herb.THC	-0.258	0.131	4.91E-02	4.80E+00	-
Oesophagus	Tobacco:Herb.THC	-0.545	0.214	1.13E-02	5.14E+00	-
Brain	LM.Cannabis	-0.507	0.127	7.34E-05	5.32E+00	-
Oropharynx	Income	-0.177	0.300	5.56E-01	5.73E+00	-
Liver	LM.Cannabis	-0.485	0.136	4.21E-04	5.81E+00	-
Melanoma	LM.Cannabis	-0.567	0.134	2.44E-05	5.82E+00	-
Hodgkins	Tobacco:Herb.THC	-0.360	0.152	1.82E-02	5.99E+00	-
Oropharynx_Broad	Tobacco:LM.Cannabis	-0.470	0.103	6.87E-06	6.20E+00	-
Leukaemia - Lymphoid	Tobacco:LM.Cannabis:Herb.THC	-0.130	0.132	3.30E-01	6.54E+00	-
All Cancers	Income	-0.315	0.031	3.15E-21	8.09E+00	-
Testis	Tobacco:Herb.THC	-1.699	0.554	2.30E-03	8.72E+00	-
All Cancers nNMSC	Tobacco:Herb.THC	-0.224	0.135	9.80E-02	9.80E+00	-
Leukaemia - Myeloid	Resin.THC	-0.363	1.347	7.88E-01	1.01E+01	-
Lung	LM.Cannabis	-0.475	0.076	8.21E-10	1.02E+01	-

Kidney	LM.Cannabis	-0.747	0.108	8.61E-12	1.25E+01	-
Lung	Income	-0.532	0.029	2.31E-62	1.26E+01	-
Leukaemia - Lymphoid	Income	-0.197	0.180	2.78E-01	1.31E+01	-
Breast	Tobacco:Herb.THC	-1.448	0.299	1.57E-06	1.43E+01	-
Stomach	Resin.THC	-1.453	0.347	3.21E-05	1.44E+01	-
Kidney	Income	-0.799	0.041	4.21E-69	1.44E+01	-
Pancreas	Income	-0.782	0.040	1.63E-69	1.45E+01	-
Pancreas	LM.Cannabis	-0.824	0.105	1.40E-14	1.62E+01	-
Corpus Uteri	Resin.THC	-2.146	0.481	9.27E-06	1.65E+01	-
Kaposi	Income	-0.454	1.090	6.80E-01	2.00E+01	-
Melanoma	Tobacco:Herb.THC	-1.144	0.202	2.25E-08	2.02E+01	-
Prostate	Resin.THC	-2.775	0.500	4.08E-08	2.81E+01	-
Ovary	Resin.THC	-2.242	0.389	1.21E-08	3.12E+01	-
Colorectum	Tobacco:Herb.THC	-2.629	0.381	1.12E-11	3.37E+01	-
Brain	Resin.THC	-1.344	0.226	3.98E-09	3.43E+01	-
Cervix	Resin.THC	-2.831	0.474	3.69E-09	3.45E+01	-
Anus	Tobacco:Herb.THC	-1.023	0.167	1.97E-09	4.20E+01	-
Stomach	LM.Cannabis:Herb.THC	-2.308	3.642	5.26E-01	4.80E+01	-
Gallbladder & Biliary	Tobacco:Herb.THC	-1.066	0.181	7.86E-09	5.00E+01	-
Kidney	Resin.THC	-1.464	0.191	5.61E-14	7.84E+01	-
Thyroid	Tobacco:Herb.THC	-1.520	0.189	7.00E-15	1.11E+02	-
Leukaemia - Lymphoid	Herb.THC	-0.418	4.638	9.28E-01	1.17E+02	-
Pancreas	Resin.THC	-1.625	0.186	1.72E-17	1.31E+02	-
Anus	Resin.THC	-1.421	0.187	1.81E-13	1.39E+02	-
Thyroid	Resin.THC	-1.693	0.212	1.05E-14	1.75E+02	-
All Cancers nNMSC	Income	-0.620	0.024	9.26E-101	1.88E+02	-
All Cancers	Resin.THC	-1.041	0.159	1.98E-10	2.51E+02	-
Oropharynx_Broad	Tobacco:Herb.THC	-1.969	0.435	8.51E-06	3.24E+02	-
Leukaemia - Lymphoid	Resin.THC	-0.606	0.636	3.44E-01	7.33E+02	-
Oropharynx	Tobacco:LM.Cannabis:Herb.THC	-0.923	0.273	1.16E-03	7.71E+02	-

Kaposi	Tobacco:LM.Cannabis:Herb.THC	-1.333	213.296	9.95E-01	1.86E+03	-
Gallbladder & Biliary	Resin.THC	-2.298	0.262	4.16E-17	2.12E+03	-
Bladder	LM.Cannabis:Herb.THC	-2.877	2.068	1.65E-01	2.19E+03	-
Leukaemia - Myeloid	LM.Cannabis:Herb.THC	-1.725	9.614	8.58E-01	5.65E+03	-
Liver	LM.Cannabis:Herb.THC	-3.762	2.265	9.74E-02	1.57E+04	-
Liver	Resin.THC	-3.849	0.246	1.31E-44	1.93E+04	-
Melanoma	LM.Cannabis:Herb.THC	-5.839	2.495	1.95E-02	3.02E+05	-
Oesophagus	LM.Cannabis:Herb.THC	-7.129	2.618	6.63E-03	1.78E+06	-
Testis	LM.Cannabis:Herb.THC	-15.273	5.810	8.84E-03	1.91E+06	-
Non-Hodgkins lymphoma	Herb.THC	-8.532	7.400	2.49E-01	4.86E+06	-
Corpus Uteri	Herb.THC	-14.875	12.662	2.40E-01	5.59E+06	-
Stomach	Herb.THC	-11.937	9.148	1.92E-01	2.90E+07	-
Larynx	LM.Cannabis:Herb.THC	-8.956	2.541	4.50E-04	1.00E+08	-
Prostate	Herb.THC	-18.537	13.183	1.60E-01	1.04E+08	-
Pancreas	Herb.THC	-7.851	4.906	1.10E-01	1.21E+09	-
Hodgkins	LM.Cannabis:Herb.THC	-6.746	1.594	2.77E-05	8.52E+09	-
Myeloma	LM.Cannabis:Herb.THC	-5.829	1.368	2.45E-05	9.87E+09	-
Breast	LM.Cannabis:Herb.THC	-16.472	3.687	9.17E-06	1.54E+10	-
Kidney	Herb.THC	-9.332	5.034	6.42E-02	2.98E+10	-
Gallbladder & Biliary	LM.Cannabis:Herb.THC	-7.797	1.949	7.45E-05	3.64E+10	-
Lung	Herb.THC	-7.030	3.569	4.92E-02	1.29E+11	-
Oropharynx	LM.Cannabis	-3.919	1.073	4.76E-04	1.90E+11	-
Anus	LM.Cannabis:Herb.THC	-9.820	1.753	3.55E-08	1.12E+13	-
All Cancers	LM.Cannabis:Herb.THC	-7.438	3.329	2.60E-02	1.99E+15	-
Ovary	Herb.THC	-28.432	10.253	5.69E-03	3.31E+15	-
Colorectum	LM.Cannabis:Herb.THC	-37.926	4.656	1.56E-15	1.25E+18	-
All Cancers nNMSC	LM.Cannabis:Herb.THC	-5.658	1.725	1.09E-03	2.09E+18	-
Brain	Herb.THC	-21.677	5.946	2.85E-04	2.03E+20	-
Thyroid	LM.Cannabis:Herb.THC	-18.302	1.982	8.31E-19	2.03E+21	-
Cervix	Herb.THC	-48.198	12.495	1.24E-04	2.94E+21	-

Kaposi	Tobacco	-13.206	228.346	9.54E-01	5.21E+29	-
Oropharynx_Broad	LM.Cannabis:Herb.THC	-44.739	20.963	3.36E-02	3.36E+50	-
Oropharynx	Herb.THC	-49.846	7.923	1.91E-08	8.41E+139	-
Kaposi	LM.Cannabis	-118.638	2885.207	9.67E-01	Inf	-
Kaposi	LM.Cannabis:Herb.THC	-493.820	9732.971	9.60E-01	Inf	-

Supplementary Table S35.: Summary of Significant Positive Terms from

Interactive Panel Regression at Six Temporal Lags

Cancer	Term	ß-Estimate	Std. Error	P_Value	Adj.P.FDR	Adj.P.Holm	E-Value Estimate	E-Value 95% Lower Bound
Thyroid	Herb.THC	42.032	5.452	7.28E-14	7.28E-14	7.28E-14	3.50E+48	2.01E+36
Anus	Herb.THC	33.645	4.821	9.88E-12	9.88E-12	9.88E-12	9.40E+43	5.41E+31
Colorectum	Herb.THC	89.627	11.695	5.57E-14	5.57E-14	5.57E-14	2.28E+42	4.21E+31
Oropharynx	LM.Cannabis:Herb.THC	26.474	9.156	5.01E-03	5.01E-03	5.01E-03	2.87E+74	1.97E+24
Melanoma	Herb.THC	35.988	6.202	9.69E-09	9.69E-09	9.69E-09	1.67E+32	2.90E+21
Gallbladder & Biliary	Herb.THC	25.697	5.031	4.92E-07	4.92E-07	4.92E-07	1.31E+34	1.47E+21
Breast	Herb.THC	49.072	9.167	1.15E-07	1.15E-07	1.15E-07	5.63E+29	9.77E+18
Oropharynx	Resin.THC	8.136	1.419	1.95E-07	1.95E-07	1.95E-07	1.23E+23	2.07E+15
All Cancers nNMSC	Herb.THC	12.447	4.357	4.42E-03	4.42E-03	4.42E-03	8.78E+39	6.30E+12
Oropharynx_Broad	Herb.THC	28.761	9.112	1.75E-03	1.75E-03	1.75E-03	3.89E+32	3.81E+12
Oropharynx_Broad	LM.Cannabis	15.402	3.351	6.26E-06	6.26E-06	6.26E-06	3.91E+17	1.71E+10
Testis	Herb.THC	52.101	15.980	1.19E-03	1.19E-03	1.19E-03	5.05E+20	2.91E+08
Oesophagus	Herb.THC	21.875	6.577	9.24E-04	9.24E-04	9.24E-04	3.58E+18	6.60E+07
Myeloma	Herb.THC	10.996	3.763	3.64E-03	3.64E-03	3.64E-03	3.85E+18	2.22E+06
Cervix	LM.Cannabis:Herb.THC	17.937	4.974	3.32E-04	3.32E-04	3.32E-04	1.51E+08	8.04E+03
Hodgkins	Herb.THC	10.998	4.384	1.24E-02	1.24E-02	1.24E-02	9.99E+15	5.75E+03
Bladder	Herb.THC	13.125	5.195	1.17E-02	1.17E-02	1.17E-02	1.46E+14	2.70E+03
Oropharynx	Tobacco:Herb.THC	1.255	0.222	2.66E-07	2.66E-07	2.66E-07	6.55E+03	3.98E+02
Brain	LM.Cannabis:Herb.THC	6.950	2.367	3.43E-03	3.43E-03	3.43E-03	5.19E+06	2.76E+02
Thyroid	LM.Cannabis	1.312	0.121	1.25E-24	1.25E-24	1.25E-24	6.36E+01	3.38E+01
Oesophagus	Resin.THC	1.944	0.250	2.30E-14	2.30E-14	2.30E-14	8.32E+01	3.23E+01
Colorectum	LM.Cannabis	2.454	0.250	1.85E-21	1.85E-21	1.85E-21	2.78E+01	1.62E+01

Myeloma	Resin.THC	0.779	0.146	1.55E-07	1.55E-07	1.55E-07	3.90E+01	1.27E+01
Larynx	LM.Cannabis	1.046	0.137	5.66E-14	5.66E-14	5.66E-14	1.53E+01	8.82E+00
Thyroid	Tobacco:LM.Cannabis:Herb.THC	0.668	0.066	6.12E-22	6.12E-22	6.12E-22	1.12E+01	7.78E+00
Larynx	Resin.THC	1.139	0.242	3.07E-06	3.07E-06	3.07E-06	1.86E+01	6.92E+00
Pancreas	LM.Cannabis:Herb.THC	4.265	1.953	2.93E-02	2.93E-02	2.93E-02	1.18E+05	5.73E+00
Breast	LM.Cannabis	1.157	0.197	6.93E-09	6.93E-09	6.93E-09	9.36E+00	5.25E+00
Hodgkins	LM.Cannabis	0.514	0.097	1.97E-07	1.97E-07	1.97E-07	1.03E+01	5.24E+00
Colorectum	Tobacco:LM.Cannabis:Herb.THC	1.150	0.155	3.59E-13	3.59E-13	3.59E-13	6.39E+00	4.43E+00
Cervix	Tobacco:Herb.THC	1.692	0.407	3.64E-05	3.64E-05	3.64E-05	1.05E+01	4.38E+00
Ovary	LM.Cannabis:Herb.THC	8.697	4.082	3.34E-02	3.34E-02	3.34E-02	9.04E+04	4.25E+00
Stomach	LM.Cannabis	0.980	0.196	6.90E-07	6.90E-07	6.90E-07	7.20E+00	3.99E+00
Anus	Tobacco:LM.Cannabis:Herb.THC	0.348	0.058	4.81E-09	4.81E-09	4.81E-09	5.11E+00	3.44E+00
Brain	Tobacco:Herb.THC	0.708	0.194	2.76E-04	2.76E-04	2.76E-04	8.48E+00	3.44E+00
Lung	Resin.THC	0.445	0.135	1.06E-03	1.06E-03	1.06E-03	9.15E+00	3.20E+00
Ovary	Tobacco:Herb.THC	1.123	0.334	8.13E-04	8.13E-04	8.13E-04	7.45E+00	2.97E+00
Oropharynx_Broad	Tobacco:LM.Cannabis:Herb.THC	1.495	0.655	2.30E-02	2.30E-02	2.30E-02	9.49E+01	2.88E+00
Lung	Tobacco:Herb.THC	0.381	0.116	1.09E-03	1.09E-03	1.09E-03	7.18E+00	2.84E+00
All Cancers nNMSC	Tobacco:LM.Cannabis:Herb.THC	0.182	0.055	1.02E-03	1.02E-03	1.02E-03	7.06E+00	2.84E+00
Hodgkins	Resin.THC	0.498	0.170	3.67E-03	3.67E-03	3.67E-03	9.74E+00	2.82E+00
Oropharynx_Broad	Alcohol	0.499	0.149	9.17E-04	9.17E-04	9.17E-04	6.72E+00	2.81E+00
Oropharynx	Tobacco:LM.Cannabis	0.133	0.031	6.84E-05	6.84E-05	6.84E-05	4.14E+00	2.54E+00
Myeloma	LM.Cannabis	0.280	0.084	8.81E-04	8.81E-04	8.81E-04	5.29E+00	2.50E+00
Leukaemia – Lymphoid	Alcohol	0.125	0.041	2.71E-03	2.71E-03	2.71E-03	6.20E+00	2.49E+00
Leukaemia – Myeloid	Alcohol	0.262	0.086	2.86E-03	2.86E-03	2.86E-03	6.15E+00	2.46E+00
Kidney	Tobacco:Herb.THC	0.490	0.164	2.89E-03	2.89E-03	2.89E-03	6.30E+00	2.43E+00
Breast	Tobacco:LM.Cannabis:Herb.THC	0.538	0.124	1.51E-05	1.51E-05	1.51E-05	3.63E+00	2.38E+00
Hodgkins	Tobacco:LM.Cannabis:Herb.THC	0.228	0.053	2.19E-05	2.19E-05	2.19E-05	3.65E+00	2.37E+00
Pancreas	Tobacco:Herb.THC	0.456	0.160	4.50E-03	4.50E-03	4.50E-03	5.92E+00	2.25E+00
Myeloma	Tobacco:LM.Cannabis:Herb.THC	0.185	0.046	6.03E-05	6.03E-05	6.03E-05	3.47E+00	2.24E+00
Gallbladder & Biliary	Tobacco:LM.Cannabis:Herb.THC	0.238	0.065	2.67E-04	2.67E-04	2.67E-04	3.53E+00	2.15E+00

Testis	LM.Cannabis	1.066	0.355	2.81E-03	2.81E-03	2.81E-03	4.67E+00	2.14E+00
Gallbladder & Biliary	Tobacco	0.098	0.011	1.14E-17	1.14E-17	1.14E-17	2.03E+00	1.84E+00
Larynx	Alcohol	0.148	0.019	7.76E-15	7.76E-15	7.76E-15	2.01E+00	1.80E+00
Testis	Income	0.597	0.183	1.16E-03	1.16E-03	1.16E-03	2.82E+00	1.79E+00
Stomach	Tobacco:Herb.THC	0.737	0.298	1.37E-02	1.37E-02	1.37E-02	4.98E+00	1.78E+00
Oropharynx_Broad	Tobacco	0.166	0.044	2.23E-04	2.23E-04	2.23E-04	2.44E+00	1.75E+00
All Cancers	Tobacco:LM.Cannabis:Herb.THC	0.253	0.107	1.85E-02	1.85E-02	1.85E-02	5.92E+00	1.75E+00
Colorectum	Resin.THC	1.055	0.444	1.77E-02	1.77E-02	1.77E-02	5.70E+00	1.75E+00
Larynx	Tobacco:LM.Cannabis:Herb.THC	0.257	0.085	2.55E-03	2.55E-03	2.55E-03	2.71E+00	1.68E+00
Melanoma	Tobacco:LM.Cannabis:Herb.THC	0.233	0.084	5.53E-03	5.53E-03	5.53E-03	2.60E+00	1.57E+00
Colorectum	Tobacco	0.157	0.017	1.40E-18	1.40E-18	1.40E-18	1.65E+00	1.54E+00
Oesophagus	Tobacco:LM.Cannabis:Herb.THC	0.236	0.087	7.05E-03	7.05E-03	7.05E-03	2.52E+00	1.52E+00
Testis	Tobacco:LM.Cannabis:Herb.THC	0.504	0.194	9.60E-03	9.60E-03	9.60E-03	2.53E+00	1.48E+00
Breast	Income	0.225	0.075	2.64E-03	2.64E-03	2.64E-03	2.07E+00	1.47E+00
Breast	Tobacco	0.102	0.014	1.83E-13	1.83E-13	1.83E-13	1.57E+00	1.46E+00
Thyroid	Alcohol	0.074	0.018	3.88E-05	3.88E-05	3.88E-05	1.73E+00	1.46E+00
Oropharynx_Broad	Income	0.184	0.074	1.34E-02	1.34E-02	1.34E-02	2.60E+00	1.45E+00
All Cancers	Alcohol	0.048	0.015	2.12E-03	2.12E-03	2.12E-03	1.80E+00	1.39E+00
Bladder	Resin.THC	0.414	0.197	3.61E-02	3.61E-02	3.61E-02	4.92E+00	1.35E+00
All Cancers nNMSC	Tobacco	0.021	0.006	9.66E-04	9.66E-04	9.66E-04	1.60E+00	1.33E+00
Thyroid	Tobacco	0.036	0.009	9.34E-05	9.34E-05	9.34E-05	1.43E+00	1.27E+00
Testis	Tobacco	0.104	0.027	1.12E-04	1.12E-04	1.12E-04	1.43E+00	1.27E+00
Pancreas	Tobacco:LM.Cannabis	0.025	0.003	2.58E-12	2.58E-12	2.58E-12	1.33E+00	1.27E+00
Colorectum	Alcohol	0.102	0.034	2.74E-03	2.74E-03	2.74E-03	1.48E+00	1.24E+00
Kidney	Tobacco:LM.Cannabis	0.021	0.004	2.92E-09	2.92E-09	2.92E-09	1.30E+00	1.23E+00
Lung	Tobacco:LM.Cannabis	0.015	0.003	4.98E-09	4.98E-09	4.98E-09	1.29E+00	1.23E+00
Stomach	Tobacco	0.050	0.014	2.47E-04	2.47E-04	2.47E-04	1.35E+00	1.22E+00
Bladder	Tobacco	0.028	0.008	2.71E-04	2.71E-04	2.71E-04	1.35E+00	1.22E+00
Hodgkins	Alcohol	0.037	0.014	1.01E-02	1.01E-02	1.01E-02	1.51E+00	1.21E+00
Liver	Tobacco:LM.Cannabis:Herb.THC	0.159	0.075	3.46E-02	3.46E-02	3.46E-02	2.28E+00	1.20E+00

Liver	Tobacco:LM.Cannabis	0.018	0.004	5.02E-05	5.02E-05	5.02E-05	1.26E+00	1.17E+00
Melanoma	Tobacco	0.028	0.009	2.12E-03	2.12E-03	2.12E-03	1.31E+00	1.17E+00
Prostate	Tobacco:Herb.THC	0.858	0.430	4.61E-02	4.61E-02	4.61E-02	3.98E+00	1.15E+00
Larynx	Tobacco	0.027	0.009	4.25E-03	4.25E-03	4.25E-03	1.30E+00	1.15E+00
Anus	Tobacco	0.020	0.008	1.14E-02	1.14E-02	1.14E-02	1.32E+00	1.13E+00
Corpus Uteri	Tobacco	0.050	0.019	8.37E-03	8.37E-03	8.37E-03	1.28E+00	1.13E+00
Brain	Tobacco:LM.Cannabis	0.014	0.004	1.02E-03	1.02E-03	1.02E-03	1.20E+00	1.12E+00
Melanoma	Tobacco:LM.Cannabis	0.014	0.004	1.60E-03	1.60E-03	1.60E-03	1.20E+00	1.12E+00
Brain	Income	0.099	0.048	3.99E-02	3.99E-02	3.99E-02	1.77E+00	1.11E+00
Oesophagus	Tobacco:LM.Cannabis	0.014	0.005	2.96E-03	2.96E-03	2.96E-03	1.19E+00	1.11E+00
Non-Hodgkins lymphoma	Tobacco:LM.Cannabis	0.015	0.005	3.83E-03	3.83E-03	3.83E-03	1.19E+00	1.10E+00
Cervix	Tobacco:LM.Cannabis	0.022	0.009	1.08E-02	1.08E-02	1.08E-02	1.18E+00	1.08E+00
Stomach	Alcohol	0.055	0.027	4.06E-02	4.06E-02	4.06E-02	1.37E+00	1.06E+00
Prostate	Tobacco	0.040	0.020	4.05E-02	4.05E-02	4.05E-02	1.24E+00	1.04E+00

Table Key: As in Tables 8 and 12.

Supplementary Table S36.: Summary of Significant Positive Terms from

Interactive Panel Regression at Six Temporal Lags

Term	Count	Negative Total of P-Value Exponents	Mean of the Negative P-Value Exponents	Median of the Negative P-Value Exponents	Total of the Lower E-Value Exponents	Mean of the Lower E-Value Exponents	Median of the Lower E-Value Exponents
Herb.THC	13	70	5.38	3	209	16.08	12
LM.Cannabis : Herb.THC	5	9	1.80	2	29	5.80	2
Resin.THC	8	36	4.50	3.5	17	2.13	0
LM.Cannabis	9	86	9.56	6	12	1.33	0
Tobacco: Herb.THC	9	24	2.67	2	2	0.22	0
Alcohol	9	31	3.44	2	0	0	0
Income	4	6	1.50	1.5	0	0	0
Tobacco	14	72	5.14	3	0	0	0
Tobacco:LM.Cannabis	10	44	4.40	3	0	0	0
Tobacco:LM.Cannabis:Herb.THC	15	69	4.6	2	0	0	0

Table Key: ":" – Interaction; LM.Cannabis – Last Month cannabis use; Herb.THC – THC concentration of cannabis herb.

SUPPLEMENTARY FIGURES

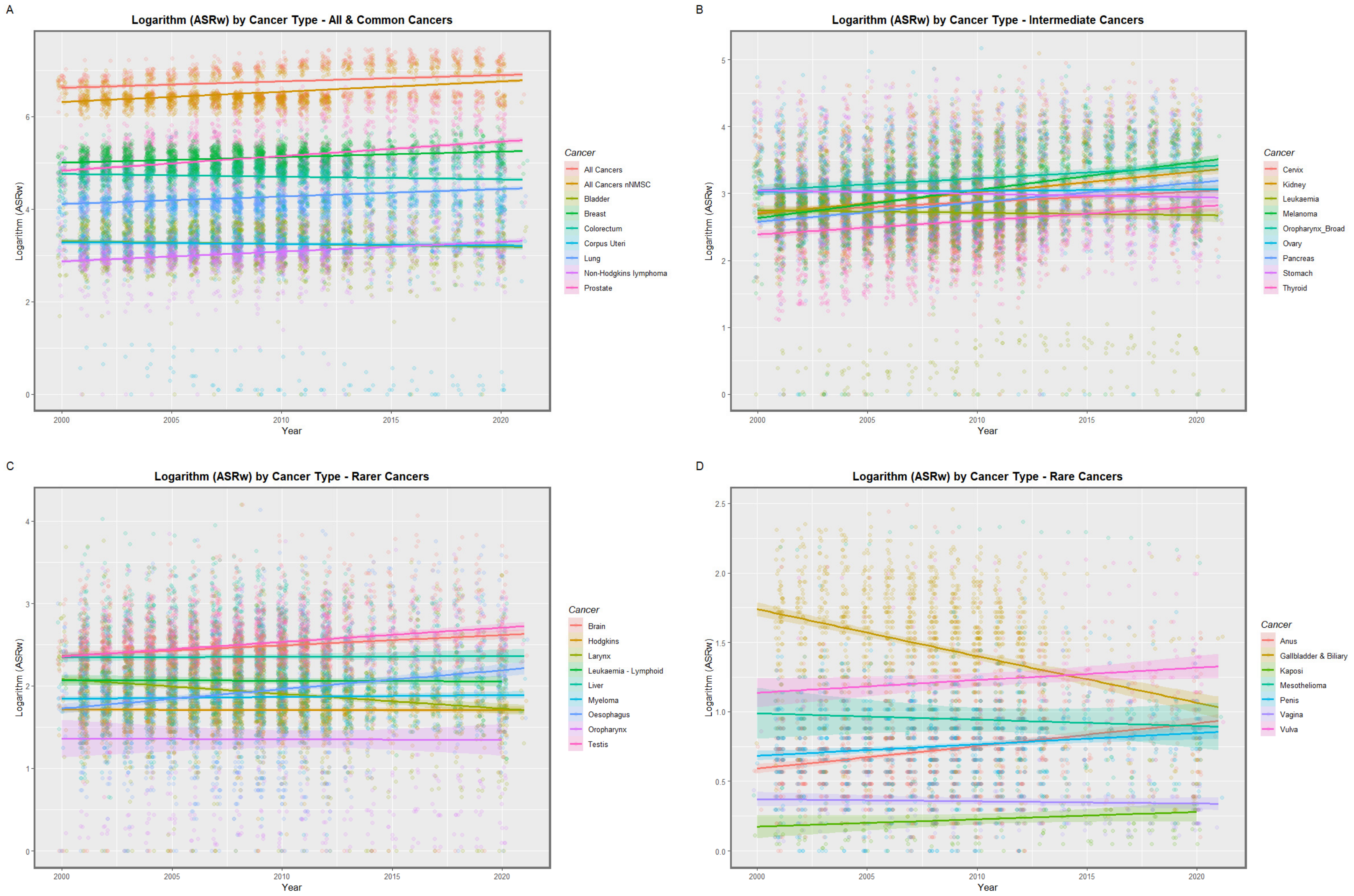
A
Logarithm (ASRw) by Cancer Type - All & Common Cancers
Logarithm (ASRw)
Year
Cancer
All Cancers
All Cancers nNMSC
Bladder
Breast
Colorectum
Corpus Uteri
Lung
Non-Hodgkins lymphoma
Prostate
B
Logarithm (ASRw) by Cancer Type - Intermediate Cancers
Logarithm (ASRw)
Year
Cancer
Cervix
Kidney
Leukaemia
Melanoma
Oropharynx_Broad
Ovary
Pancreas
Stomach
Thyroid
C
Logarithm (ASRw) by Cancer Type - Rarer Cancers
Logarithm (ASRw)
Year
Cancer
Brain
Hodgkins
Larynx
Leukaemia - Lymphoid
Liver
Myeloma
Oesophagus
Oropharynx
Testis
D
Logarithm (ASRw) by Cancer Type - Rare Cancers
Logarithm (ASRw)
Year
Cancer
Anus
Gallbladder & Biliary
Kaposi
Mesothelioma
Penis
Vagina
Vulva

Supplementary Figure S1.: Cancer trends across time for (A) common, (B) intermediate, (C) rarer and (D) rare cancer types.

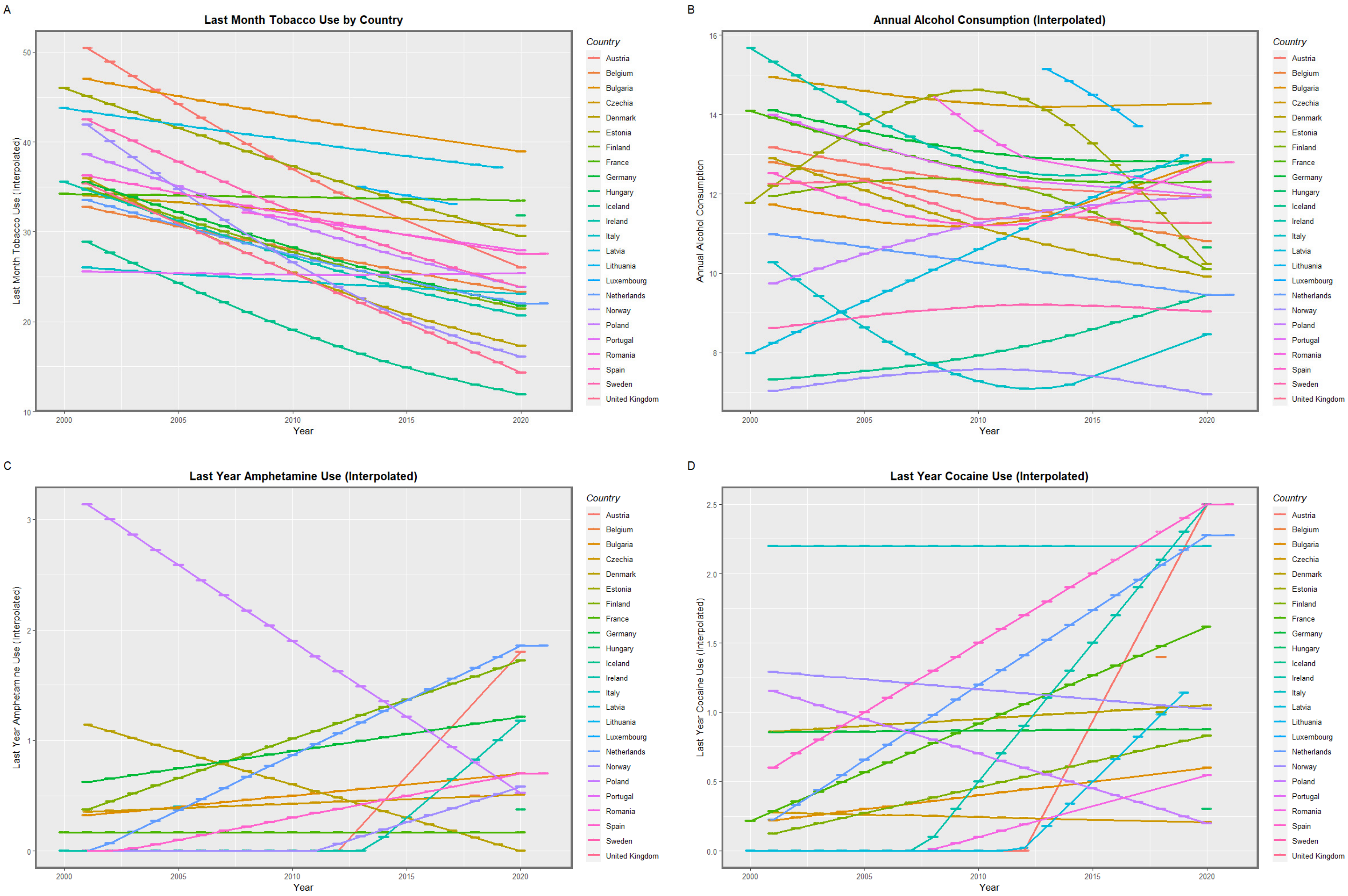
A
Last Month Tobacco Use by Country
Last Month Tobacco Use (Interpolated)
Year
B
Annual Alcohol Consumption (Interpolated)
Annual Alcohol Consumption
Year
C
Last Year Amphetamine Use (Interpolated)
Last Year Amphetamine Use (Interpolated)
Year
D
Last Year Cocaine Use (Interpolated)
Last Year Cocaine Use (Interpolated)
Year
Country
Austria
Belgium
Bulgaria
Czechia
Denmark
Estonia
Finland
France
Germany
Hungary
Iceland
Ireland
Italy
Latvia
Lithuania
Luxembourg
Netherlands
Norway
Poland
Portugal
Romania
Spain
Sweden
United Kingdom

Supplementary Figure S2.: Trends in substance use across time interpolated for (A) last month tobacco use, (B) annual alcohol consumption, (C) last year amphetamine use and (D) last year cocaine use.

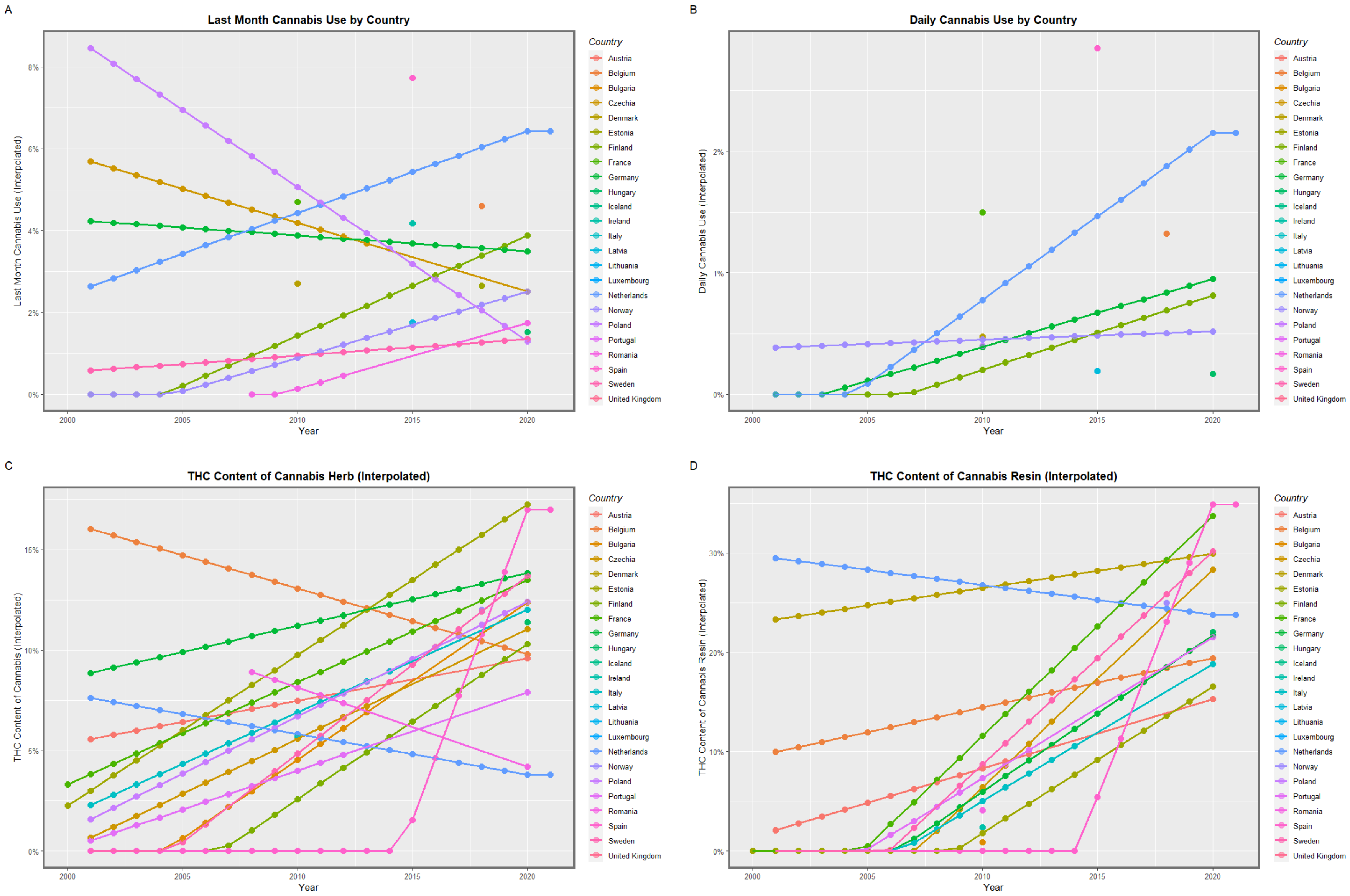
A
Last Month Cannabis Use by Country
Last Month Cannabis Use (Interpolated)
0%
2%
4%
6%
8%
2000
2005
2010
2015
2020
Year
B
Daily Cannabis Use by Country
Daily Cannabis Use (Interpolated)
0%
1%
2%
2000
2005
2010
2015
2020
Year
C
THC Content of Cannabis Herb (Interpolated)
THC Content of Cannabis (Interpolated)
0%
5%
10%
15%
2000
2005
2010
2015
2020
Year
D
THC Content of Cannabis Resin (Interpolated)
THC Content of Cannabis Resin (Interpolated)
0%
10%
20%
30%
2000
2005
2010
2015
2020
Year
Country
Austria
Belgium
Bulgaria
Czechia
Denmark
Estonia
Finland
France
Germany
Hungary
Iceland
Ireland
Italy
Latvia
Lithuania
Luxembourg
Netherlands
Norway
Poland
Portugal
Romania
Spain
Sweden
United Kingdom

Supplementary Figure S3.: Trends across time for selected cannabis metrics. (A) Last month cannabis use, (B) daily cannabis use, (C) THC concentration of cannabis herb and (D) THC concentration of cannabis resin.

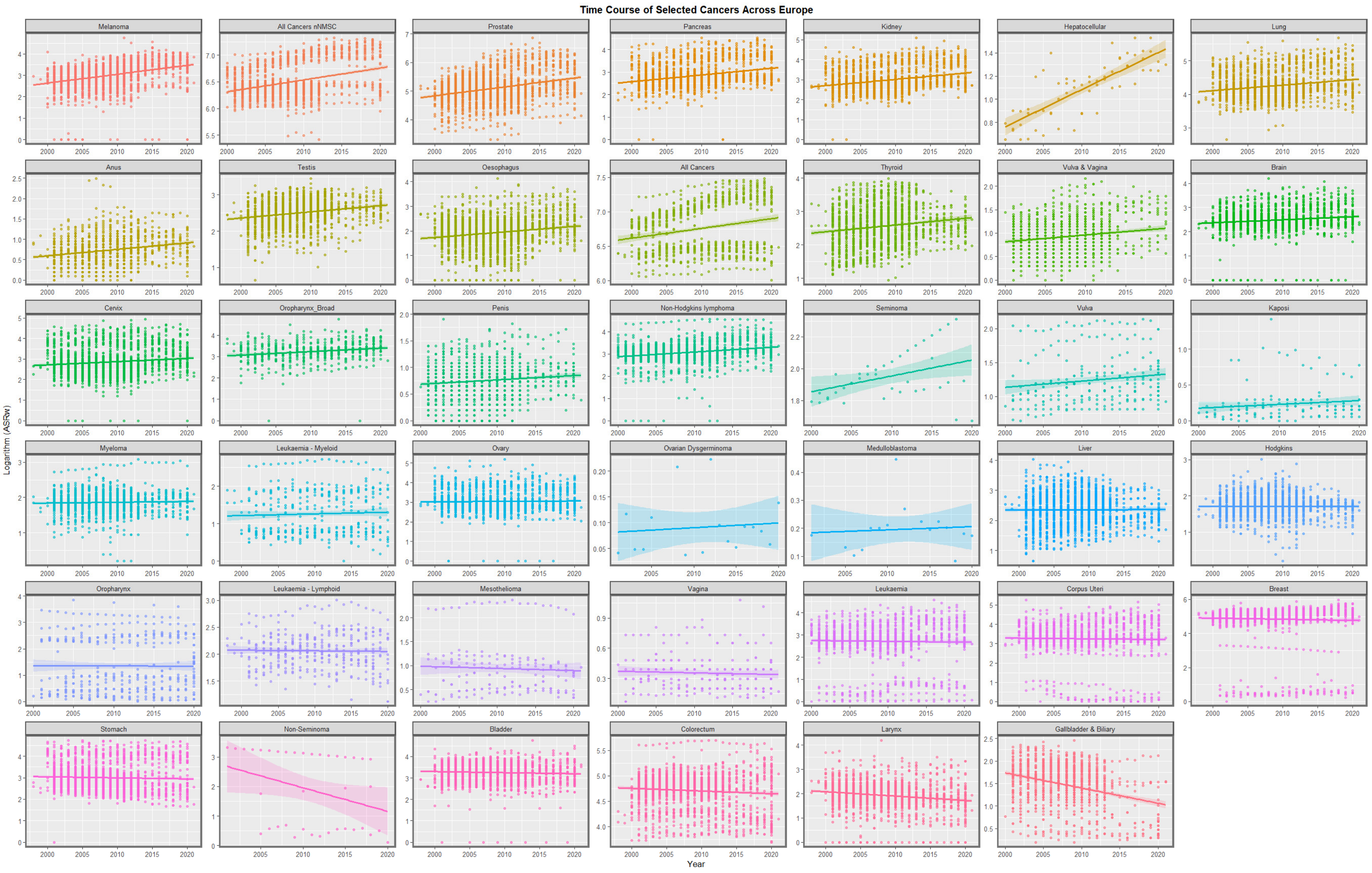
Time Course of Selected Cancers Across Europe
Melanoma
All Cancers nNMSC
Prostate
Pancreas
Kidney
Hepatocellular
Lung
Anus
Testis
Oesophagus
All Cancers
Thyroid
Vulva & Vagina
Brain
Cervix
Oropharynx_Broad
Penis
Non-Hodgkins lymphoma
Seminoma
Vulva
Kaposi
Myeloma
Leukaemia - Myeloid
Ovary
Ovarian Dysgerminoma
Medulloblastoma
Liver
Hodgkins
Oropharynx
Leukaemia - Lymphoid
Mesothelioma
Vagina
Leukaemia
Corpus Uteri
Breast
Stomach
Non-Seminoma
Bladder
Colorectum
Larynx
Gallbladder & Biliary
Logarithm (ASRw)
Year

Supplementary Figure S4.: Trends across time for selected cancer types. The log of the age standardized rate standardized to the world population 1976 is plotted on the ordinate axis.

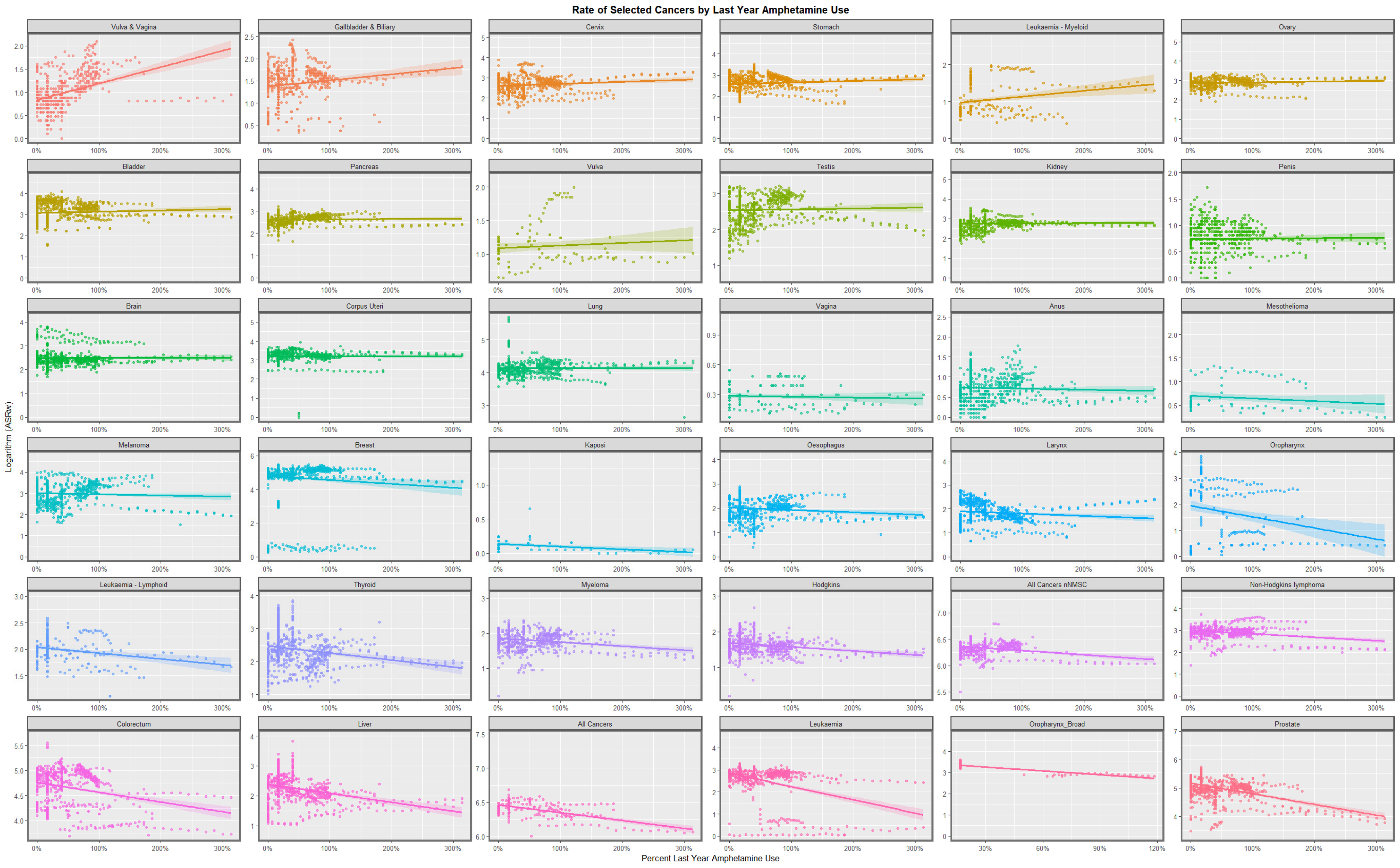
Rate of Selected Cancers by Last Year Amphetamine Use
Vulva & Vagina
Gallbladder & Biliary
Cervix
Stomach
Leukaemia - Myeloid
Ovary
Bladder
Pancreas
Vulva
Testis
Kidney
Penis
Brain
Corpus Uteri
Lung
Vagina
Anus
Mesothelioma
Melanoma
Breast
Kaposi
Oesophagus
Larynx
Oropharynx
Leukaemia - Lymphoid
Thyroid
Myeloma
Hodgkins
All Cancers nNMSC
Non-Hodgkins lymphoma
Colorectum
Liver
All Cancers
Leukaemia
Oropharynx_Broad
Prostate
Logarithm (ASRw)
Percent Last Year Amphetamine Use

Supplementary Figure S5.: The log ASRw of selected cancers against last year amphetamine use.

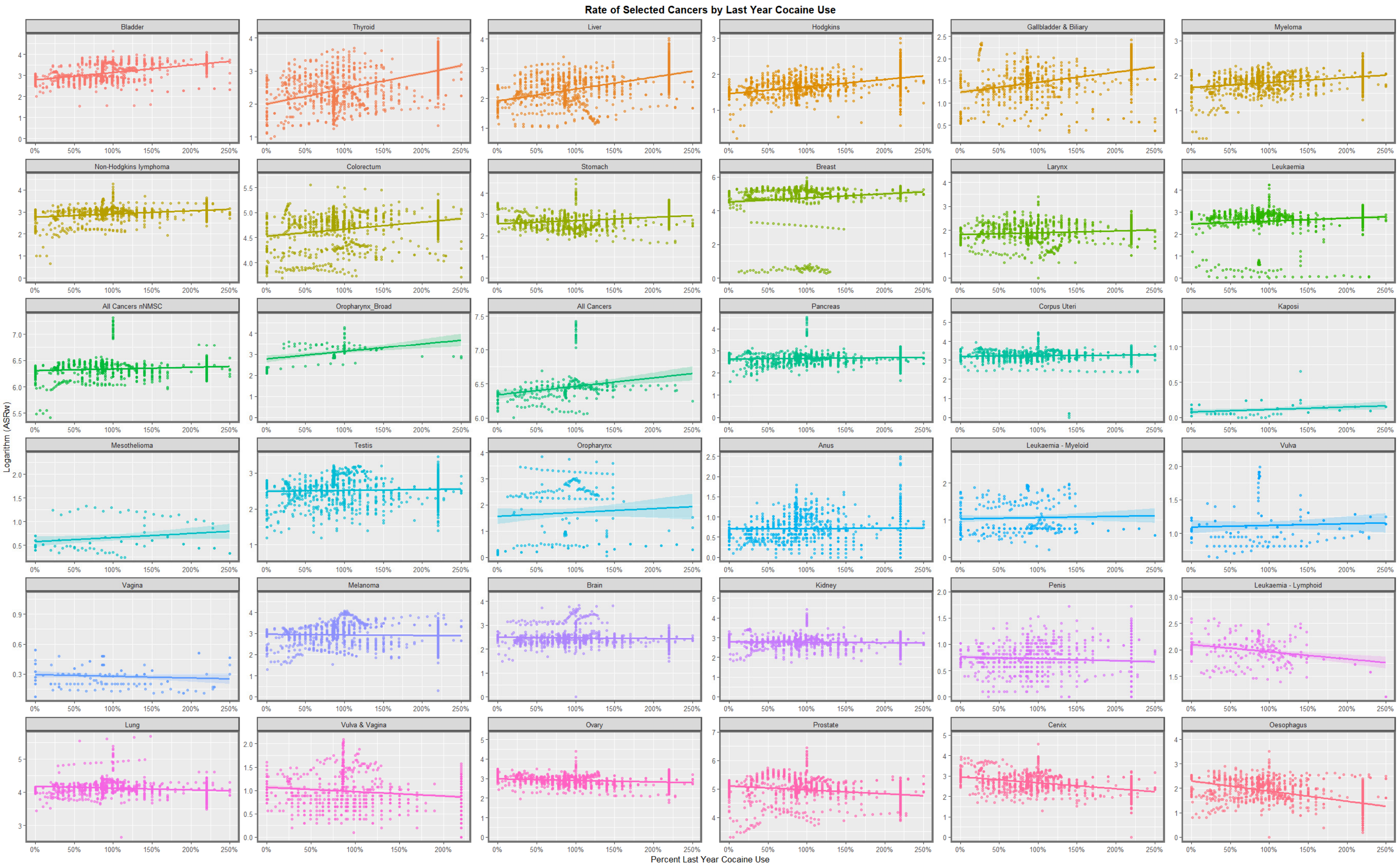

Rate of Selected Cancers by Last Year Cocaine Use
Bladder
Thyroid
Liver
Hodgkins
Gallbladder & Biliary
Myeloma
Non-Hodgkins lymphoma
Colorectum
Stomach
Breast
Larynx
Leukaemia
All Cancers nNMSC
Oropharynx_Broad
All Cancers
Pancreas
Corpus Uteri
Kaposi
Mesothelioma
Testis
Oropharynx
Anus
Leukaemia - Myeloid
Vulva
Vagina
Melanoma
Brain
Kidney
Penis
Leukaemia - Lymphoid
Lung
Vulva & Vagina
Ovary
Prostate
Cervix
Oesophagus
Logarithm (ASRw)
Percent Last Year Cocaine Use

Supplementary Figure S6.: The log ASRw of selected cancers against last year cocaine amphetamine use.

Correlation Plot - Correlation Plot - Substances and Common Cancer Rates

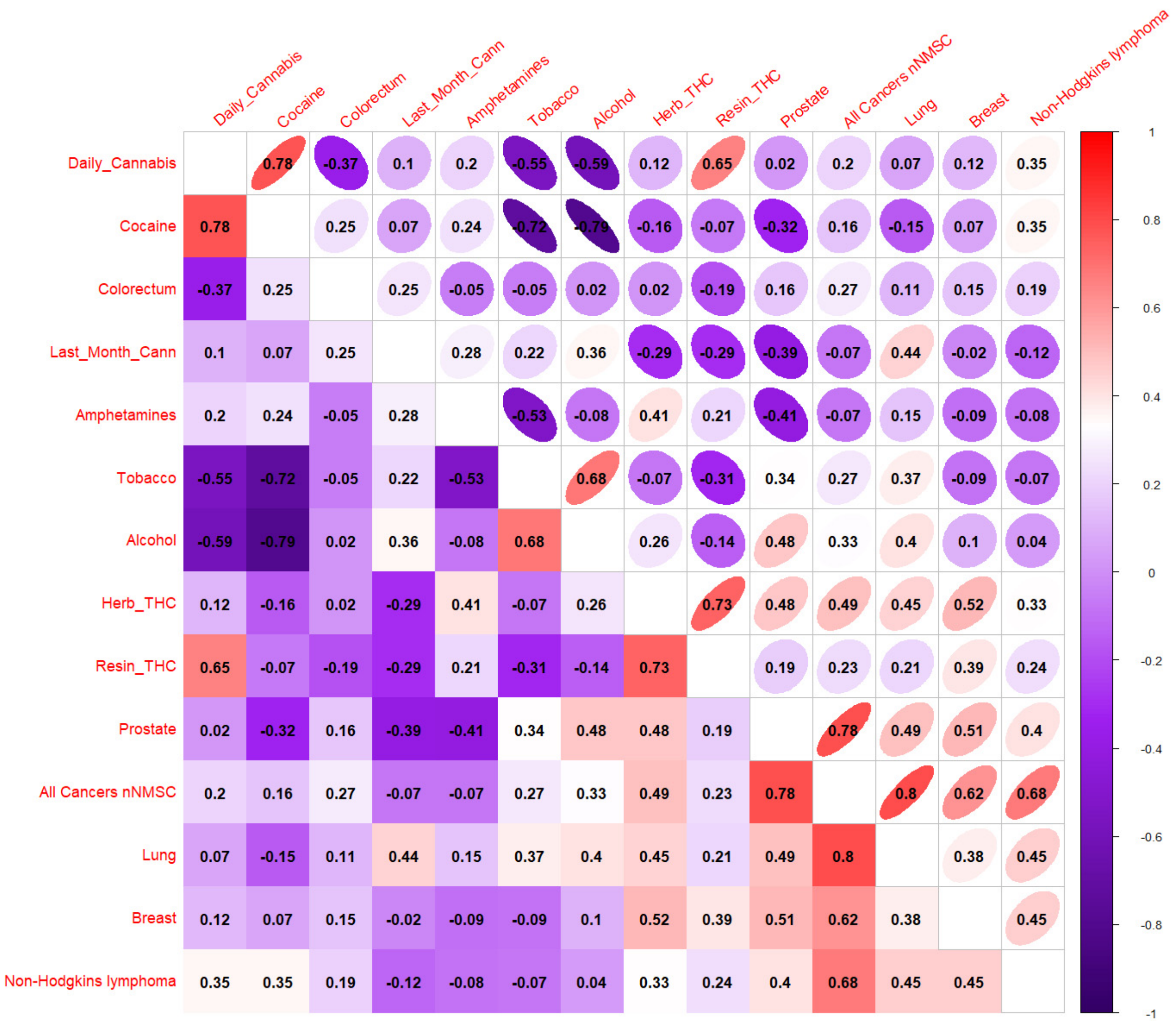

Supplementary Figure S7.: Correlogram as Correlation Matrix for Bivariate Relationships.

Significance Level Plot - Substances and Common Cancer Rates

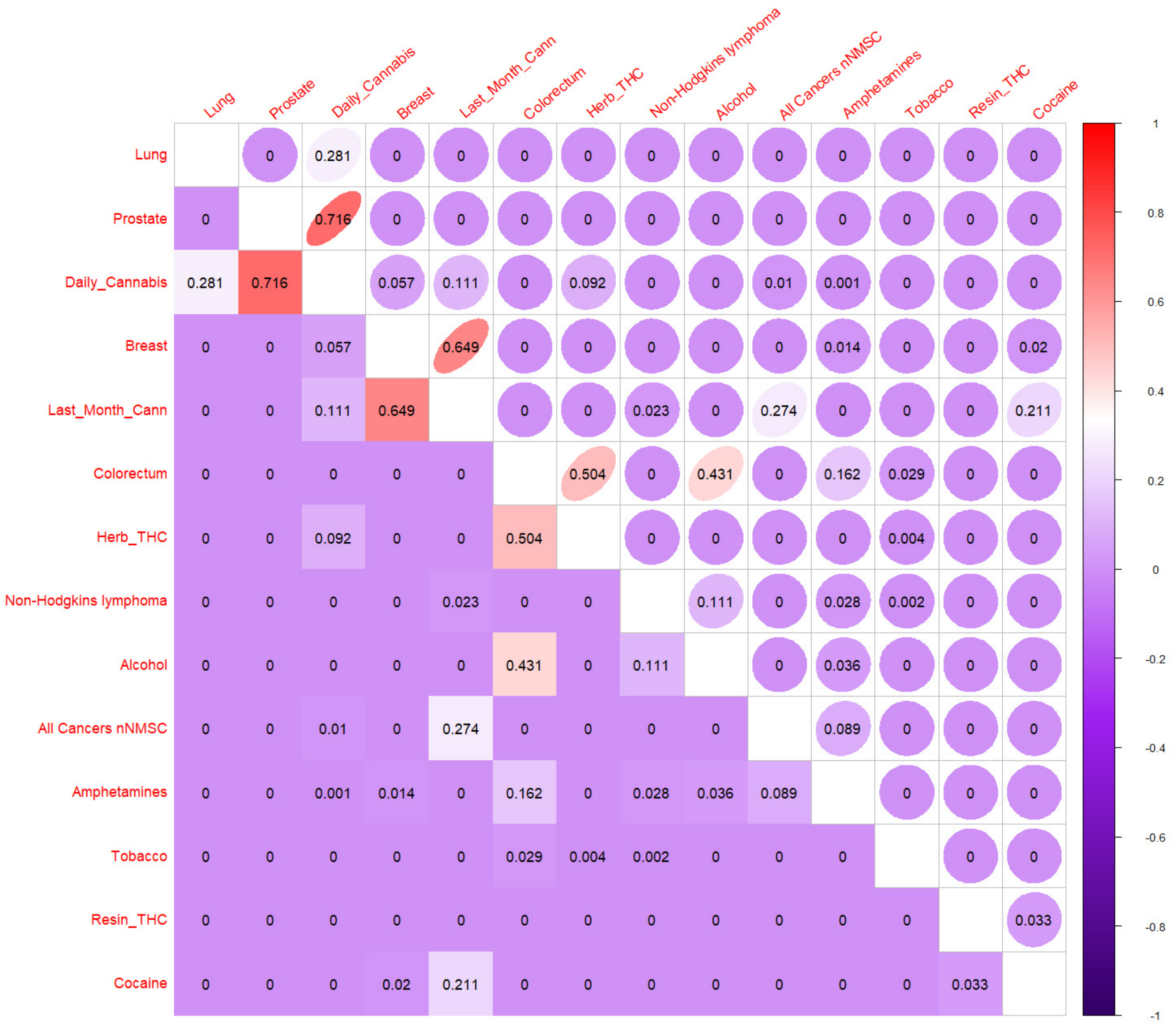

Supplementary Figure S8.: Significance of Correlation Matrix for Bivariate Relationships.

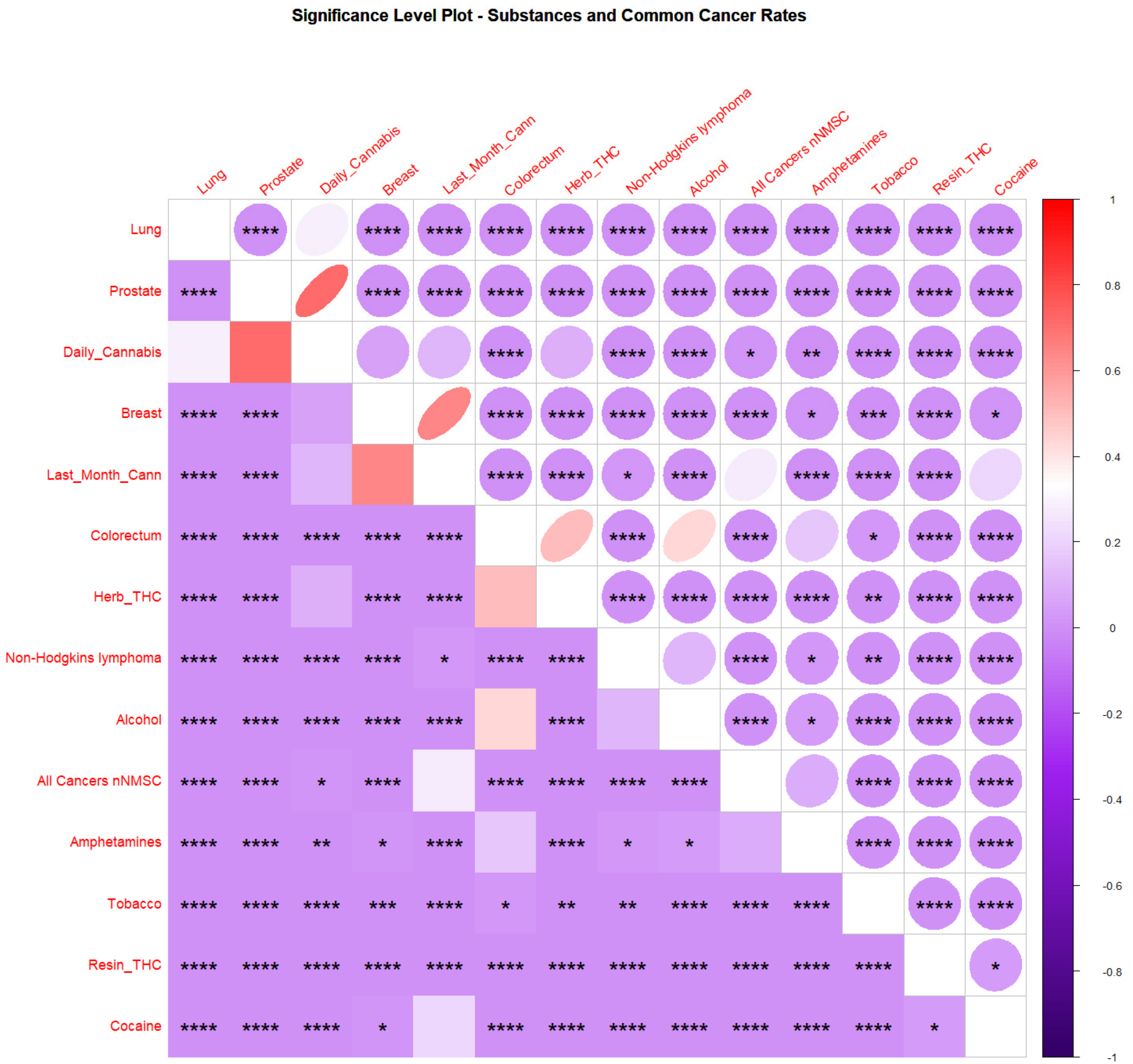

Significance Level Plot - Substances and Common Cancer Rates
Lung
Prostate
Daily_Cannabis
Breast
Last_Month_Cann
Colorectum
Herb_THC
Non-Hodgkins lymphoma
Alcohol
All Cancers nNMSC
Amphetamines
Tobacco
Resin_THC
Cocaine
1
0.8
0.6
0.4
0.2
0
-0.2
-0.4
-0.6
-0.8
-1

Supplementary Figure S9.: Semiquantitative significance testing results from the correlogram shown in Supplementary Figure S7 in the main text.

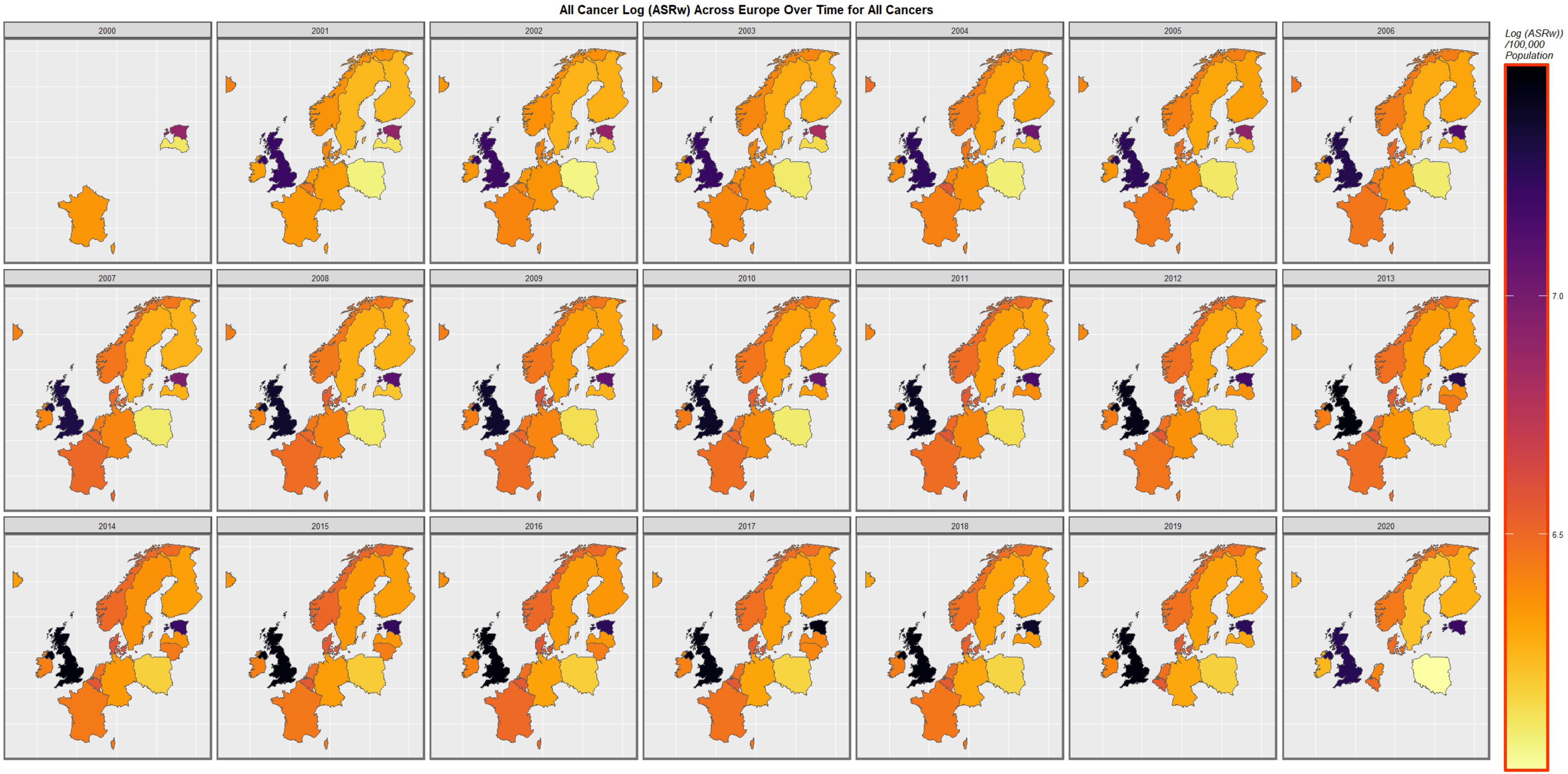
All Cancer Log (ASRw) Across Europe Over Time for All Cancers
2000
2001
2002
2003
2004
2005
2006
2007
2008
2009
2010
2011
2012
2013
2014
2015
2016
2017
2018
2019
2020
Log (ASRw))
/100,000
Population
7.0
6.5

Supplementary Figure S10.: Time trends of the Log ASRw across Europe 2000-2020.

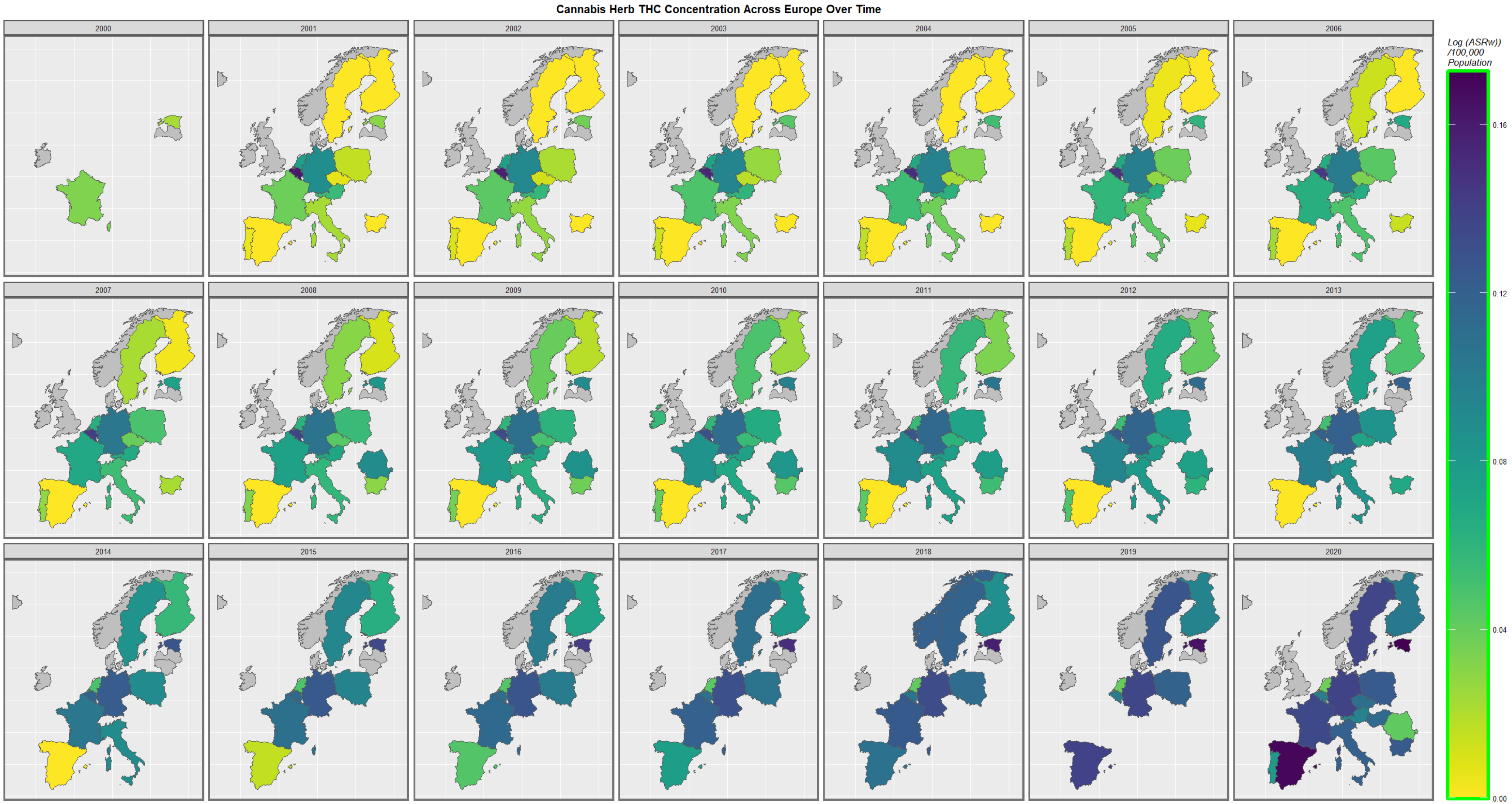
Cannabis Herb THC Concentration Across Europe Over Time
2000
2001
2002
2003
2004
2005
2006
2007
2008
2009
2010
2011
2012
2013
2014
2015
2016
2017
2018
2019
2020
Log (ASRw))
/100,000
Population
0.16
0.12
0.08
0.04
0.00

Supplementary Figure S11.: Log of All cancer Rate across Europe over time

All Cancer Log (ASRw) by Cannabis Herb THC Content Across Europe
Bivariate Choropleth Colorplane Map

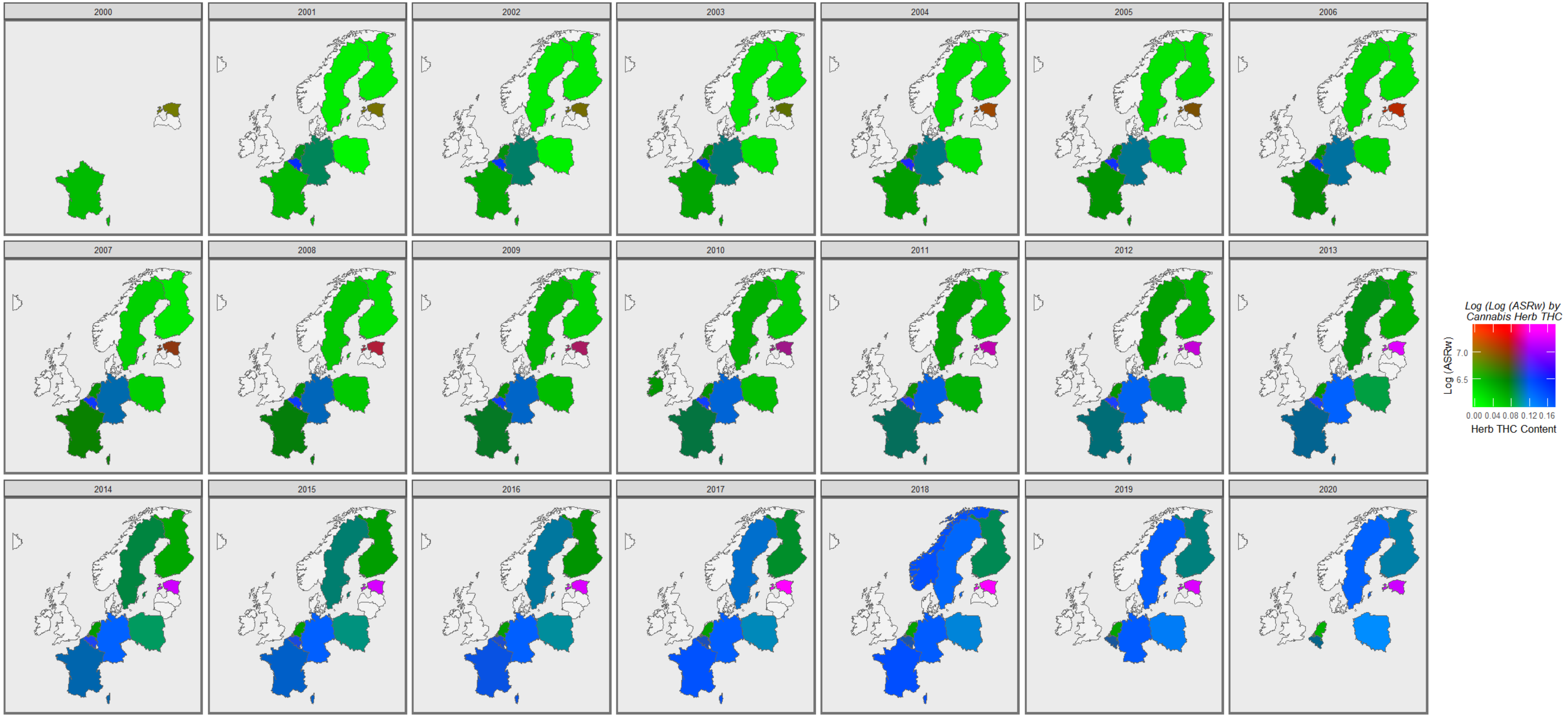

Supplementary Figure S12.: Bivariate map of All cancer rates by Log of Cannabis Herb THC Concentration Across Europe over time. Please refer to the colorplane for the interpretation of the various colour shadings. Green is where both covariates are low. Pink and purple indicates where they are both high etc.

Liver Cancer Log (ASRw) by Cannabis Herb THC Content Across Europe
Bivariate Choropleth Colorplane Map

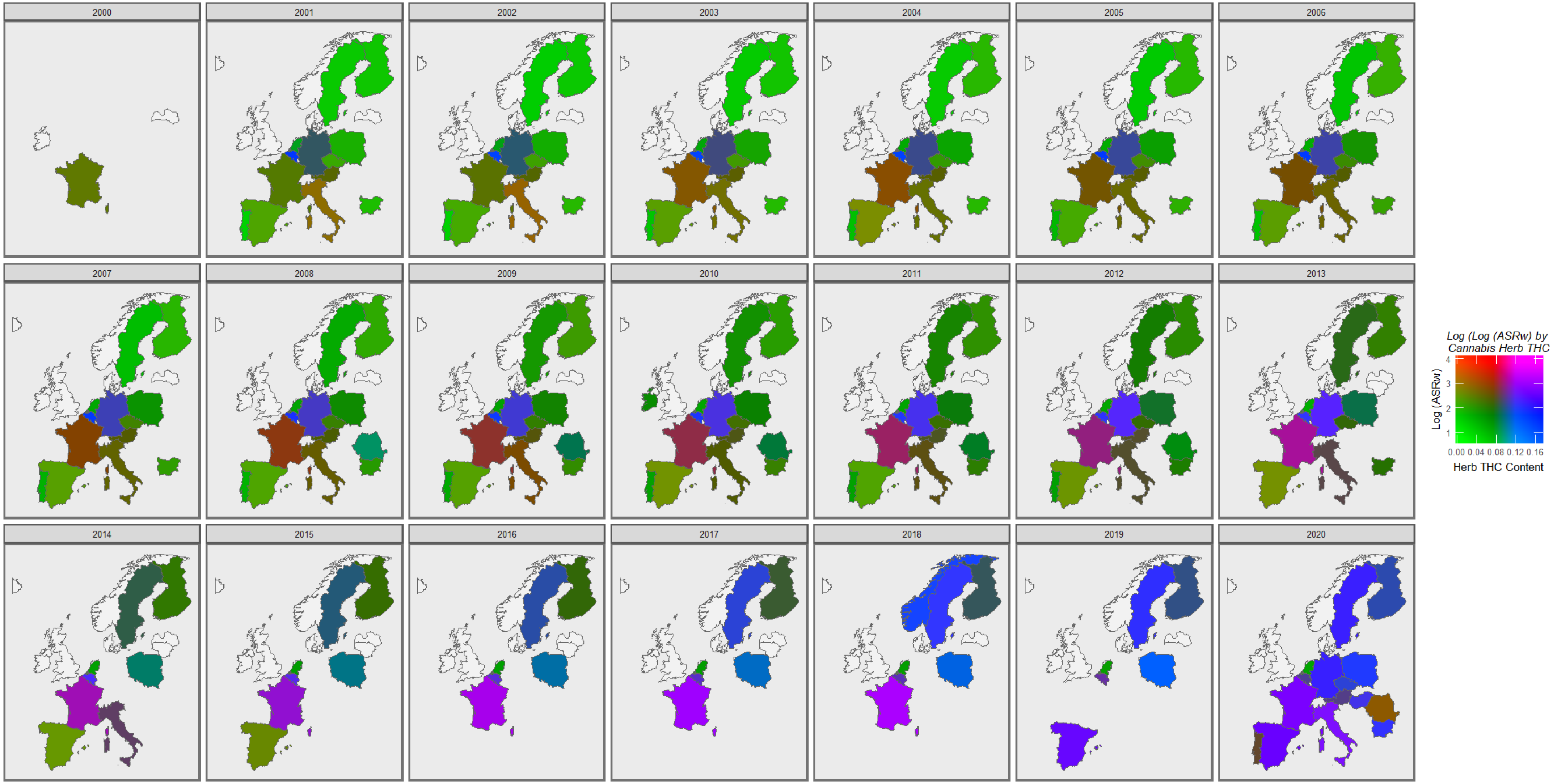

Supplementary Figure S13.: Bivariate map of Liver cancer rate by Log of Cannabis Herb THC Concentration Across Europe over time

Prostate Cancer Log (ASRw) by Cannabis Herb THC Content Across Europe
Bivariate Choropleth Colorplane Map

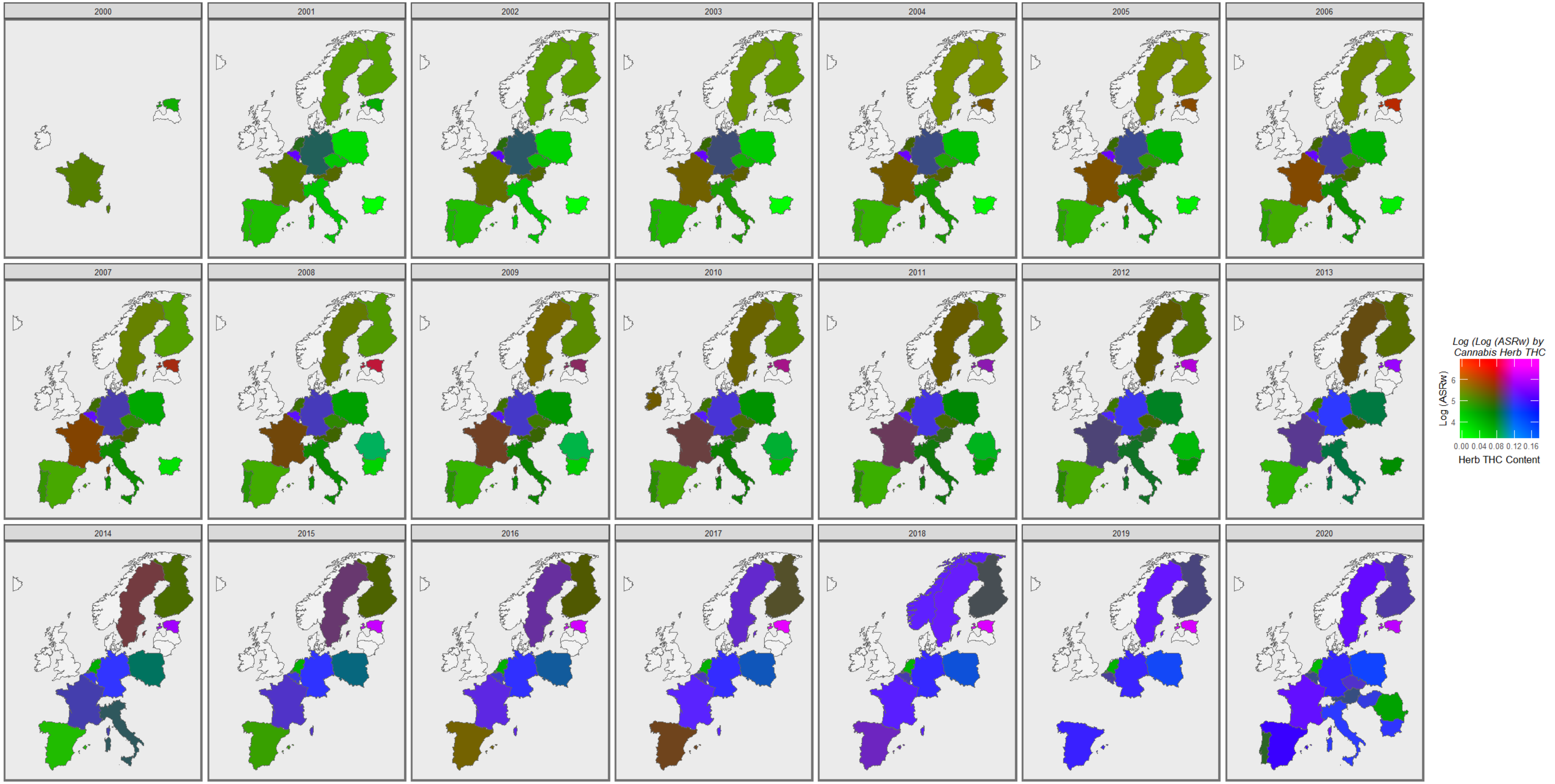

Supplementary Figure S14.: Bivariate map of Prostate cancer rate by Log of Cannabis Herb THC Concentration Across Europe over time

Colorectum Cancer Log (ASRw) by Cannabis Herb THC Content Across Europe
Bivariate Choropleth Colorplane Map

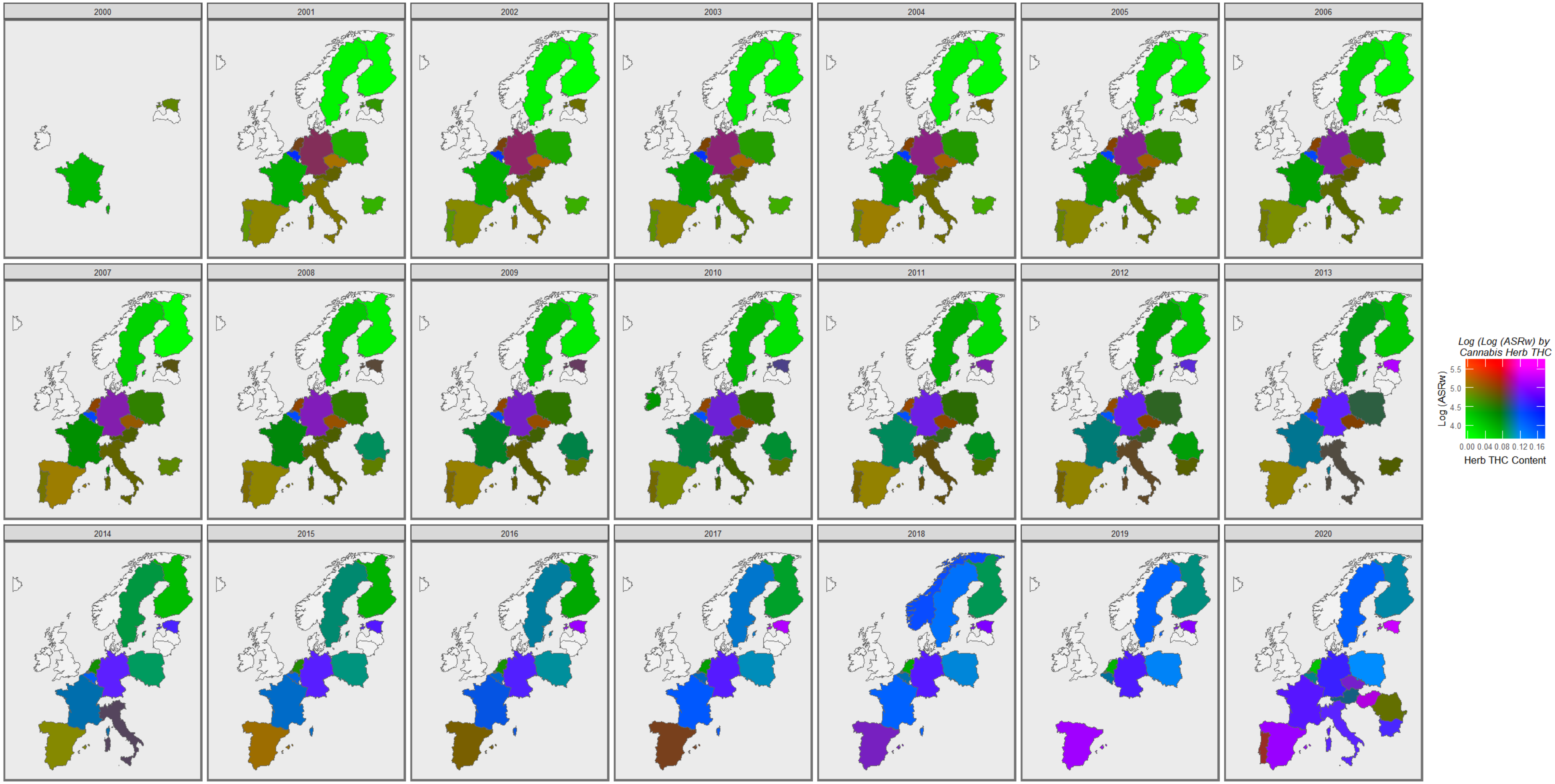

Supplementary Figure S15.: The bivariate relationship between cannabis herb THC content and colorectal cancers.

Lung Cancer Log (ASRw) by Cannabis Herb THC Content Across Europe
Bivariate Choropleth Colorplane Map

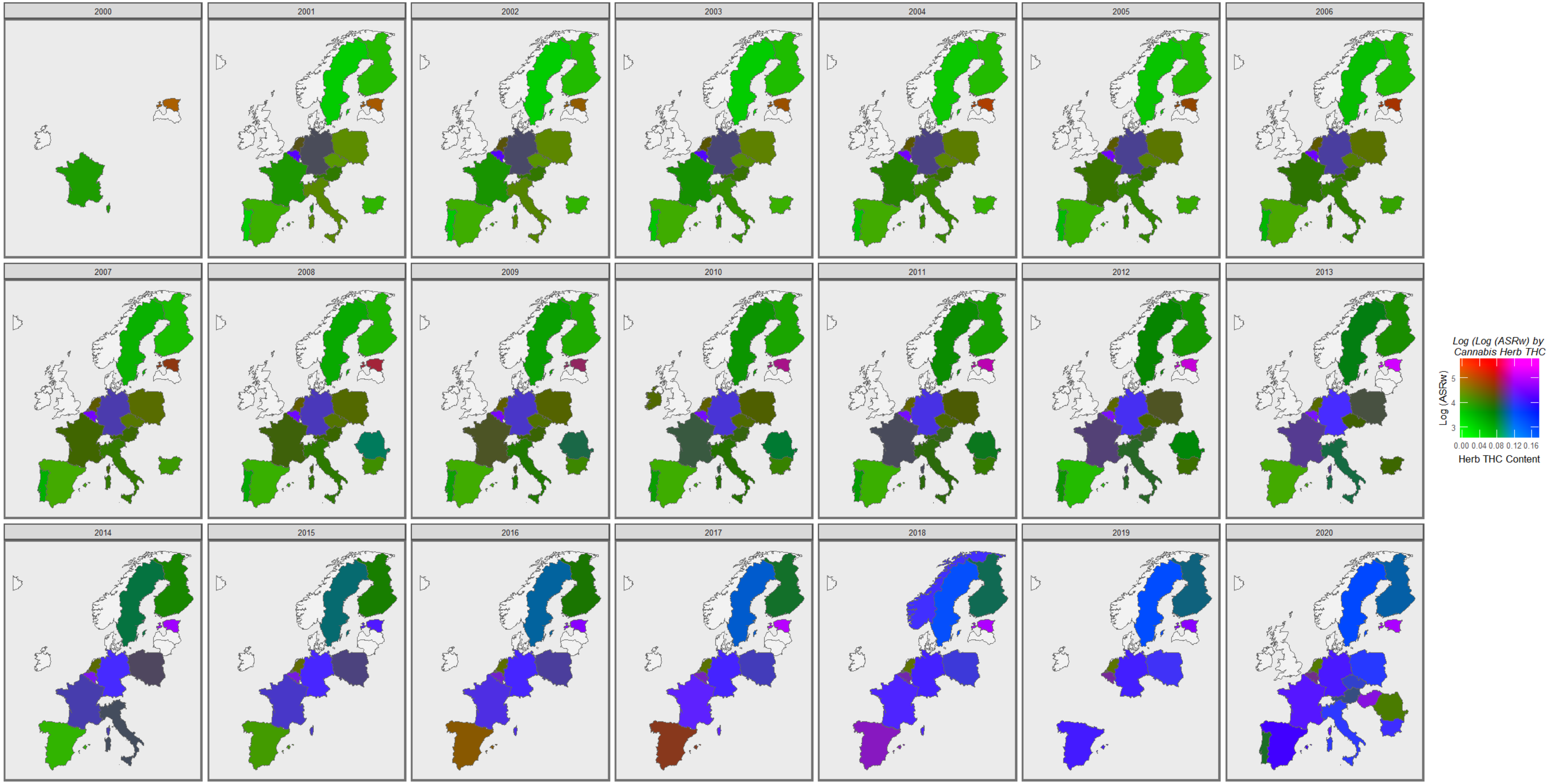

Supplementary Figure S16.: The bivariate relationship between cannabis herb THC content and lung cancers.

Non-Hodgkins Lymphoma Log (ASRw) by Cannabis Herb THC Content Across Europe
Bivariate Choropleth Colorplane Map

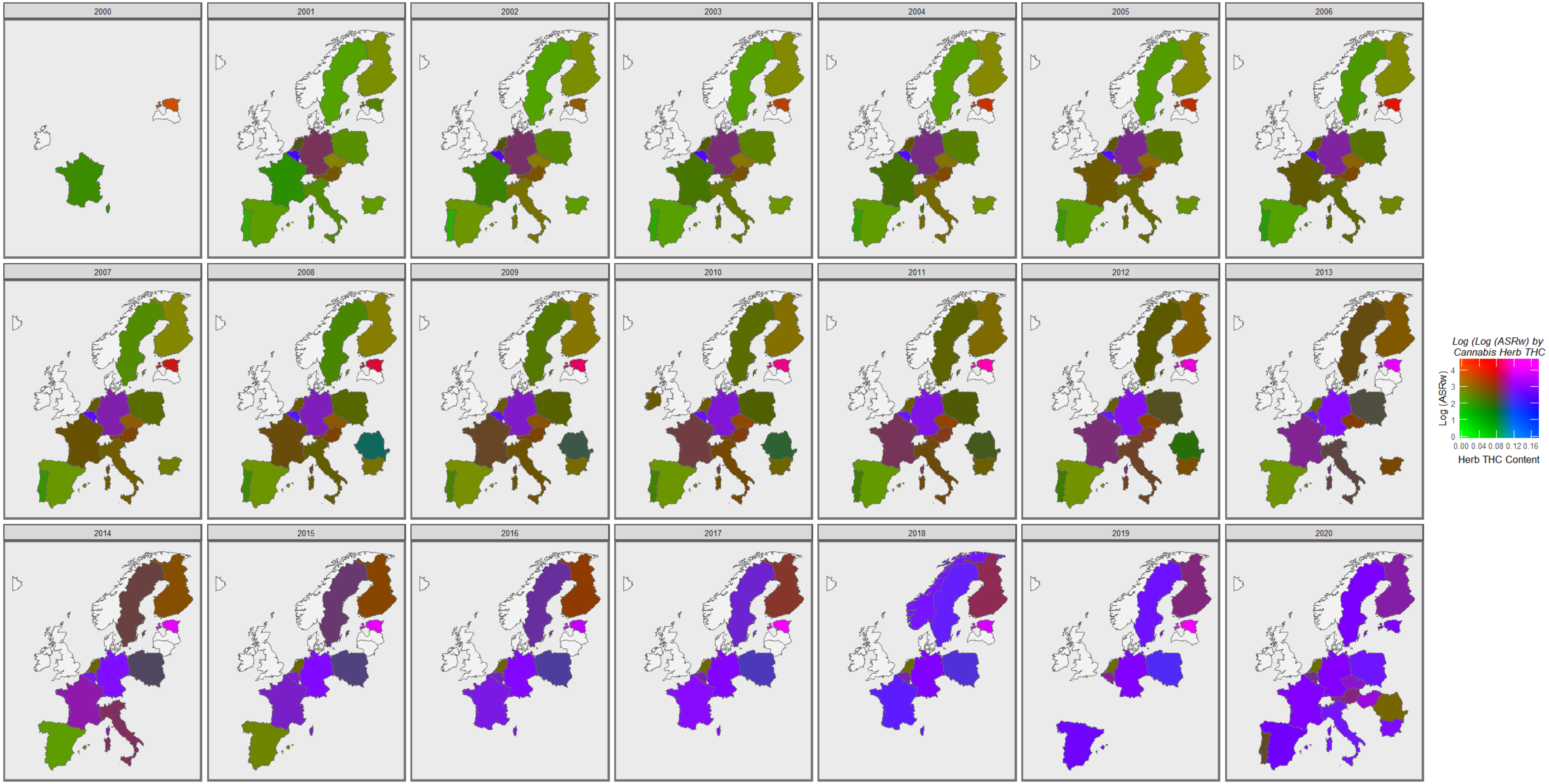

Supplementary Figure S17.: The bivariate relationship between cannabis herb THC content and rate of Non-Hodgkins Lymphoma.

Lympoid Leukaemia Log (ASRw) by Cannabis Herb THC Content Across Europe
Bivariate Choropleth Colorplane Map

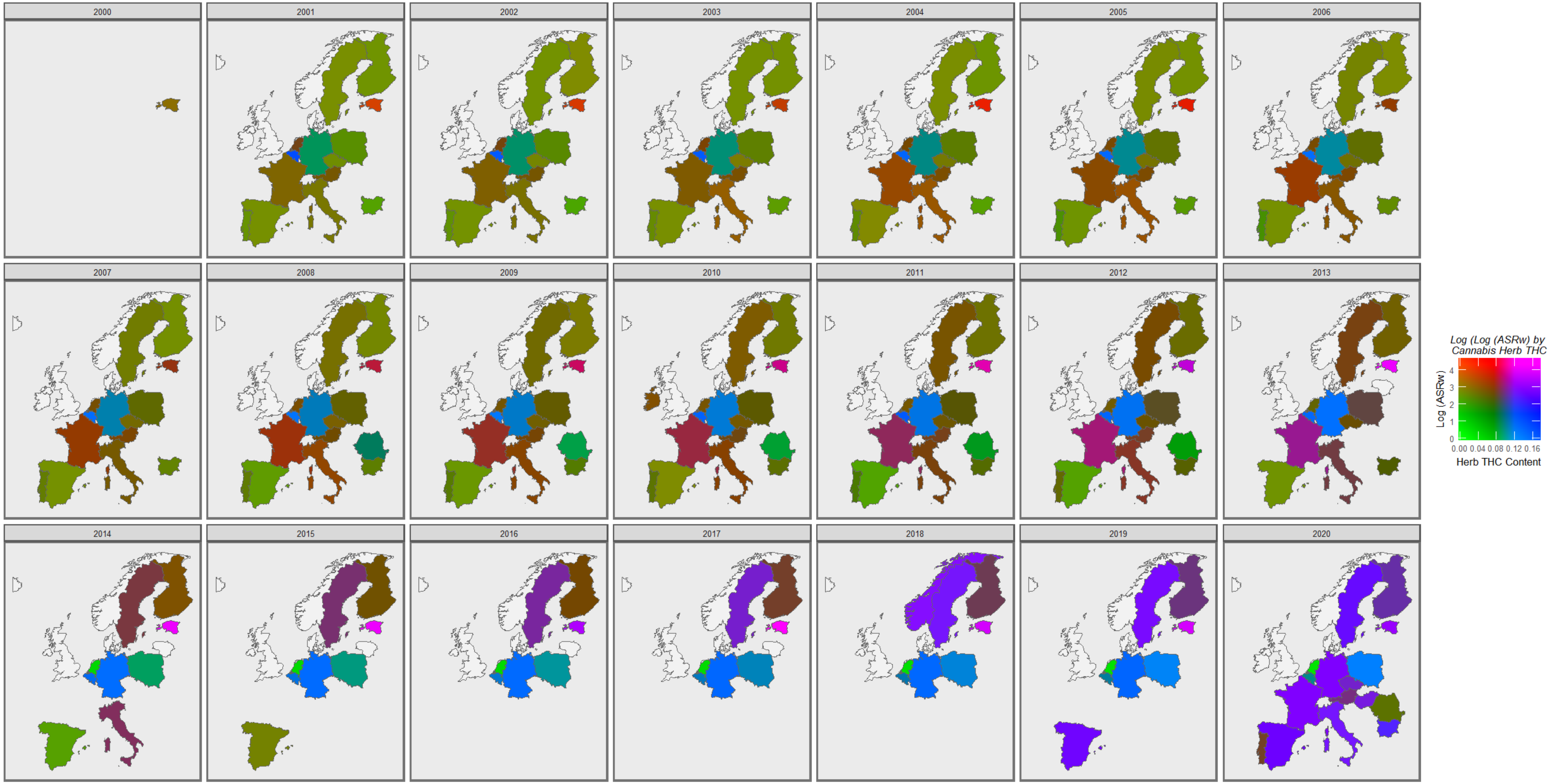

Supplementary Figure S18.: The bivariate relationship between cannabis herb THC content and lymphoid leukaemias.

Vulva Cancer Log (ASRw) by Cannabis Herb THC Content Across Europe
Bivariate Choropleth Colorplane Map

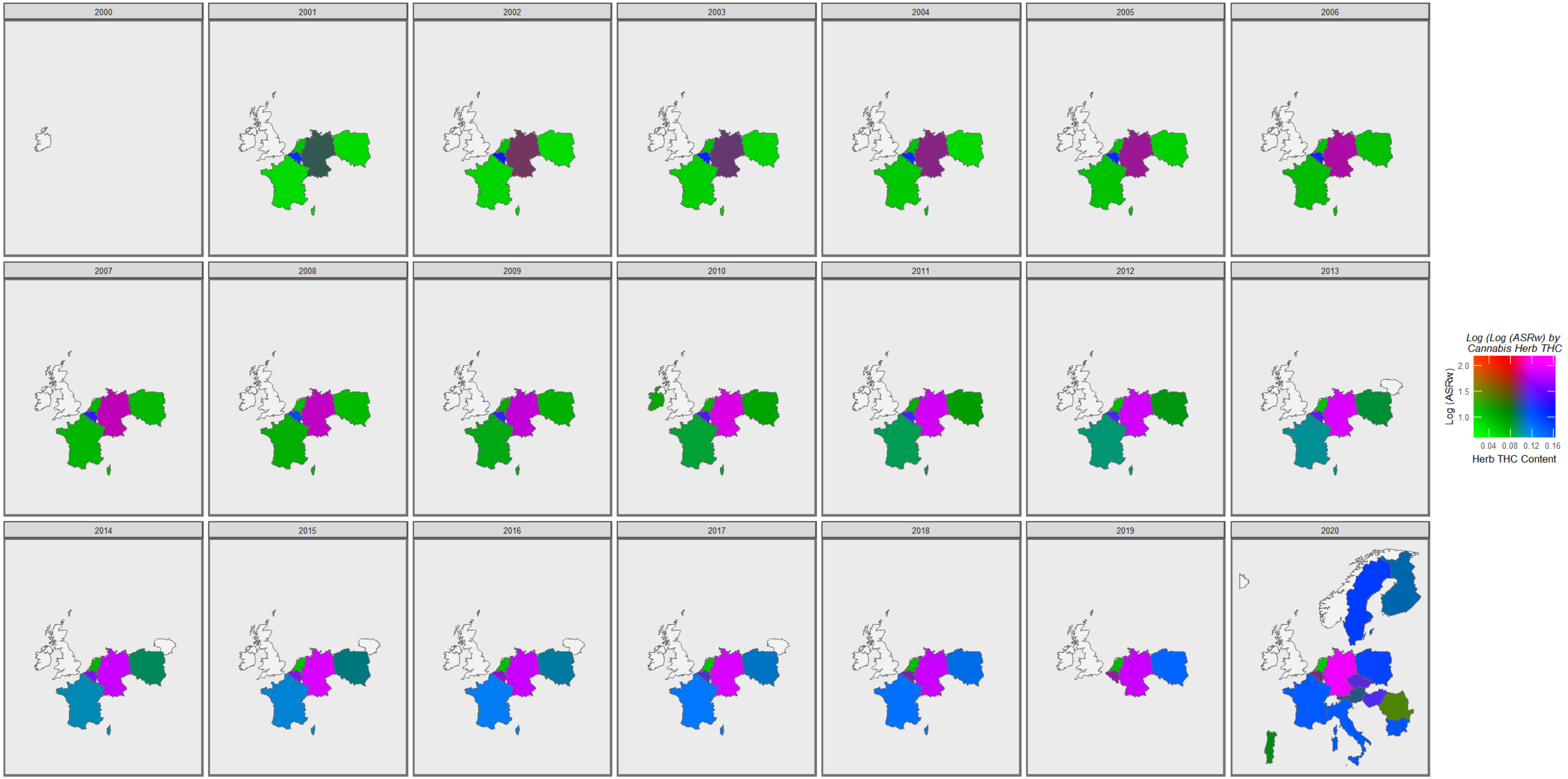

Supplementary Figure S19.: The bivariate relationship between cannabis herb THC content and vulva cancer.

Log (ASRw) by Cancer by Daily Cannabis Use Status,
- Combdf11DateRxSlp ~ HiLo

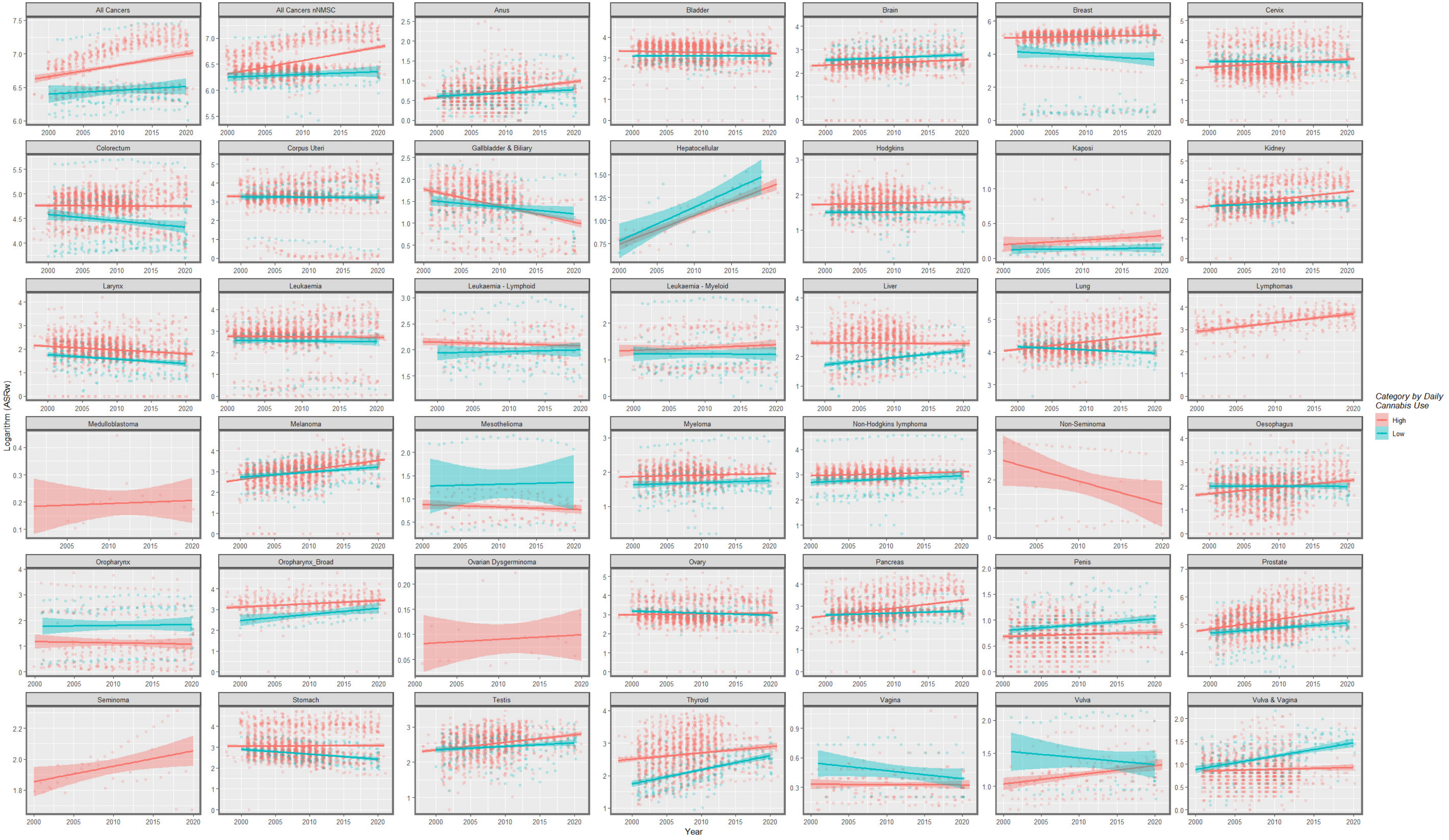

Supplementary Figure S20.: Log Rate of Selected Cancers over time by high and low cannabis use nations. (See text for details.)

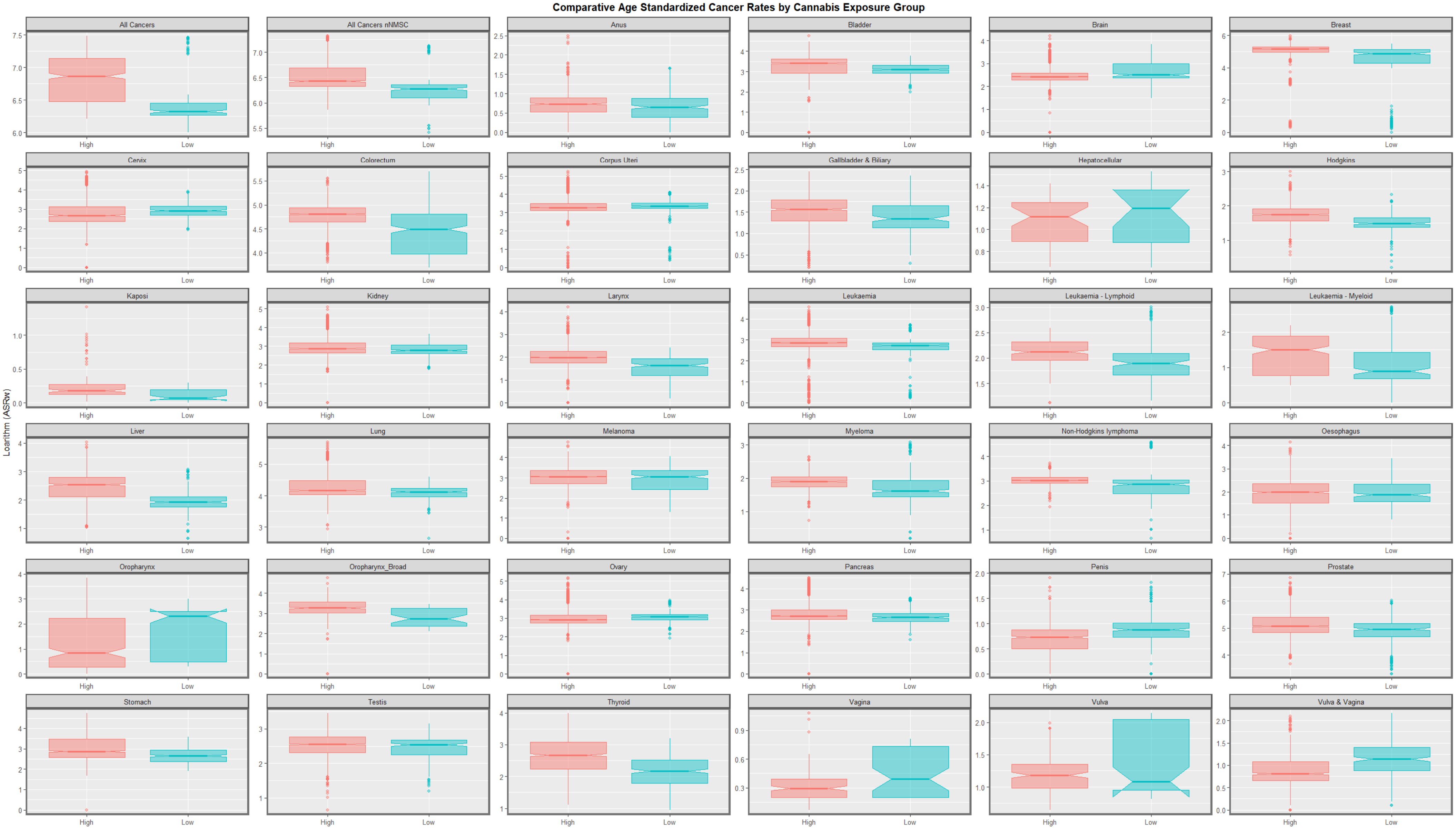

Comparative Age Standardized Cancer Rates by Cannabis Exposure Group
Logarithm (ASRw)
All Cancers
All Cancers nNMSC
Anus
Bladder
Brain
Breast
Cervix
Colorectum
Corpus Uteri
Gallbladder & Biliary
Hepatocellular
Hodgkins
Kaposi
Kidney
Larynx
Leukaemia
Leukaemia - Lymphoid
Leukaemia - Myeloid
Liver
Lung
Melanoma
Myeloma
Non-Hodgkins lymphoma
Oesophagus
Oropharynx
Oropharynx_Broad
Ovary
Pancreas
Penis
Prostate
Stomach
Testis
Thyroid
Vagina
Vulva
Vulva & Vagina
High
Low

Supplementary Figure S21.: Serial Boxplots Log Rate of Selected Cancers aggregated across time by high and low cannabis use nations. (See text for details.)

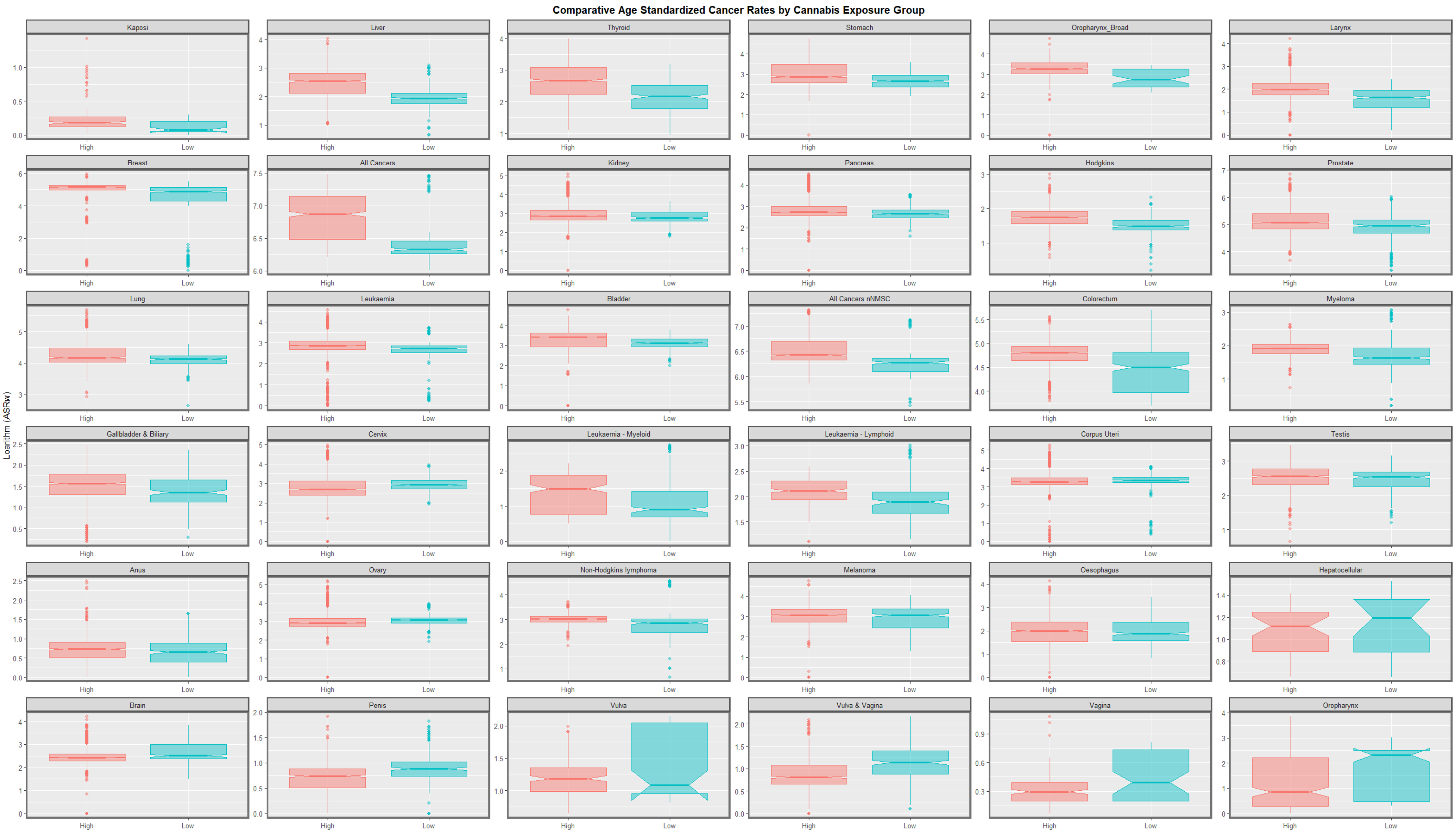

Comparative Age Standardized Cancer Rates by Cannabis Exposure Group
Logarithm (ASRw)
Kaposi
Liver
Thyroid
Stomach
Oropharynx_Broad
Larynx
Breast
All Cancers
Kidney
Pancreas
Hodgkins
Prostate
Lung
Leukaemia
Bladder
All Cancers nNMSC
Colorectum
Myeloma
Gallbladder & Biliary
Cervix
Leukaemia - Myeloid
Leukaemia - Lymphoid
Corpus Uteri
Testis
Anus
Ovary
Non-Hodgkins lymphoma
Melanoma
Oesophagus
Hepatocellular
Brain
Penis
Vulva
Vulva & Vagina
Vagina
Oropharynx
High
Low

Supplementary Figure S22.: Pannelled boxplot of the aggregated rates of various cancers ordered by the ratio between the rates in the high cannabis use group and that in the low cannabis use group.

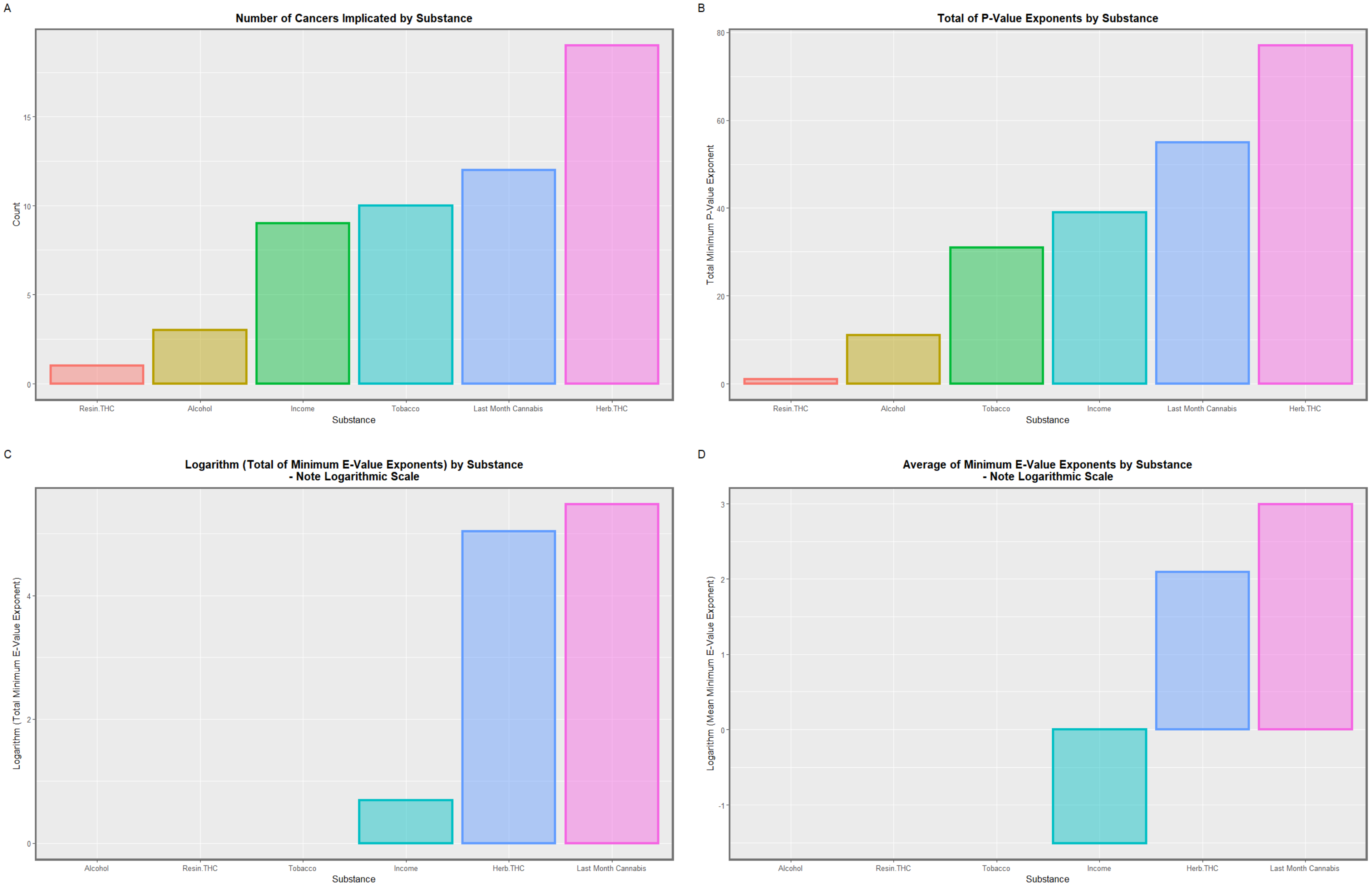
A
Number of Cancers Implicated by Substance
Count
0
5
10
15
Resin.THC
Alcohol
Income
Tobacco
Last Month Cannabis
Herb.THC
Substance
B
Total of P-Value Exponents by Substance
Total Minimum P-Value Exponent
0
20
40
60
80
Resin.THC
Alcohol
Tobacco
Income
Last Month Cannabis
Herb.THC
Substance
C
Logarithm (Total of Minimum E-Value Exponents) by Substance
- Note Logarithmic Scale
Logarithm (Total Minimum E-Value Exponent)
0
2
4
Alcohol
Resin.THC
Tobacco
Income
Herb.THC
Last Month Cannabis
Substance
D
Average of Minimum E-Value Exponents by Substance
- Note Logarithmic Scale
Logarithm (Mean Minimum E-Value Exponent)
-1
0
1
2
3
Alcohol
Resin.THC
Tobacco
Income
Herb.THC
Last Month Cannabis
Substance

Supplementary Figure S23.: Summary plots of the additive mixed effects regression. (A) number of cancer implicated by substance, (B) the total value of the negative exponents of the P-values, (C) the sum of the exponents of the minimum P-values and (D) the average of the exponents of the minimum P-values.

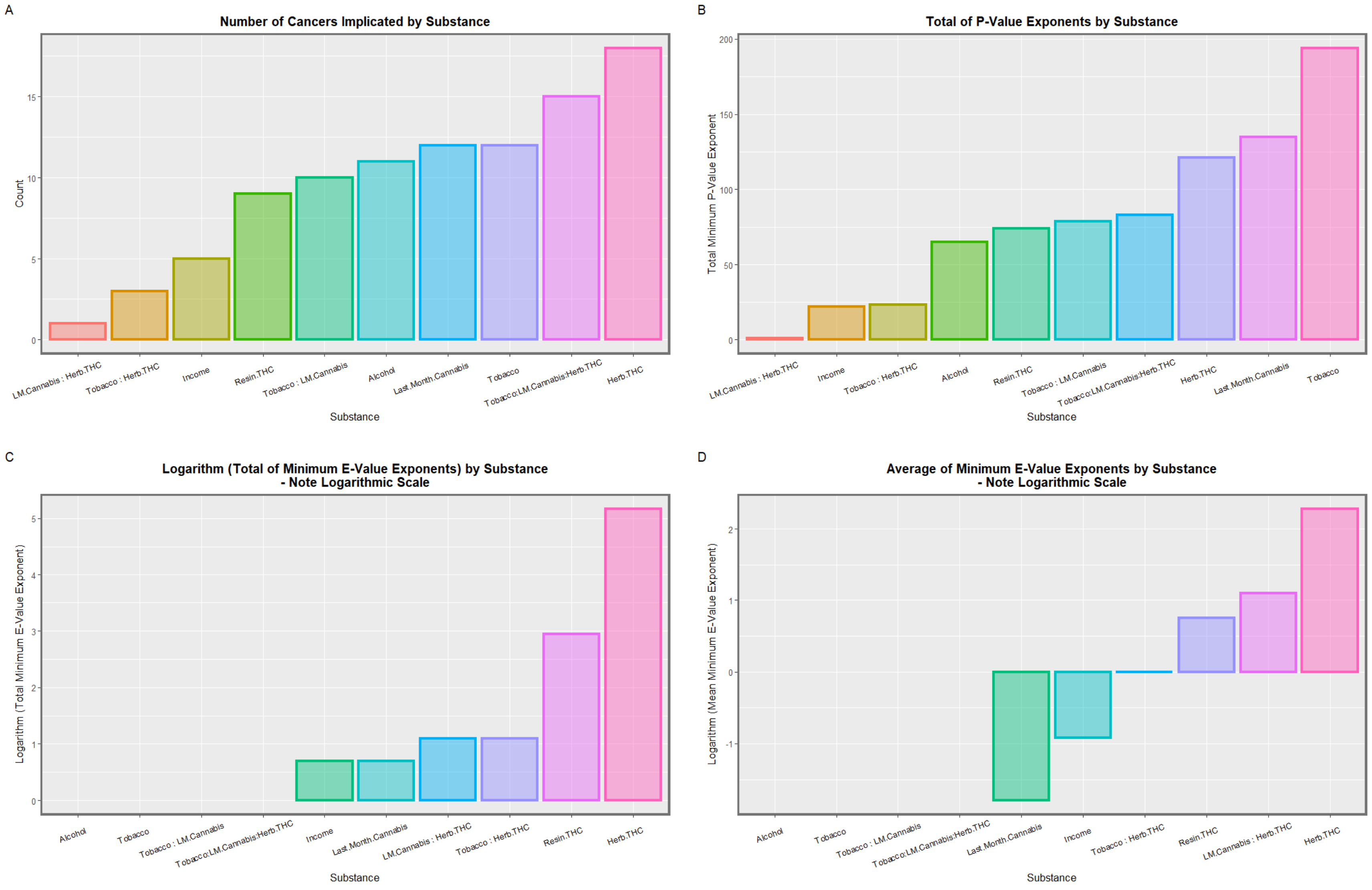
A
Number of Cancers Implicated by Substance
Count
Substance
LM.Cannabis : Herb.THC
Tobacco : Herb.THC
Income
Resin.THC
Tobacco : LM.Cannabis
Alcohol
Last.Month.Cannabis
Tobacco
Tobacco:LM.Cannabis:Herb.THC
Herb.THC
B
Total of P-Value Exponents by Substance
Total Minimum P-Value Exponent
Substance
LM.Cannabis : Herb.THC
Income
Tobacco : Herb.THC
Alcohol
Resin.THC
Tobacco : LM.Cannabis
Tobacco:LM.Cannabis:Herb.THC
Herb.THC
Last.Month.Cannabis
Tobacco
C
Logarithm (Total of Minimum E-Value Exponents) by Substance
- Note Logarithmic Scale
Logarithm (Total Minimum E-Value Exponent)
Substance
Alcohol
Tobacco
Tobacco : LM.Cannabis
Tobacco:LM.Cannabis:Herb.THC
Income
Last.Month.Cannabis
LM.Cannabis : Herb.THC
Tobacco : Herb.THC
Resin.THC
Herb.THC
D
Average of Minimum E-Value Exponents by Substance
- Note Logarithmic Scale
Logarithm (Mean Minimum E-Value Exponent)
Substance
Alcohol
Tobacco
Tobacco : LM.Cannabis
Tobacco:LM.Cannabis:Herb.THC
Last.Month.Cannabis
Income
Tobacco : Herb.THC
Resin.THC
LM.Cannabis : Herb.THC
Herb.THC

Supplementary Figure S24.: Graphical summary of interactive panel model at two years of lag.

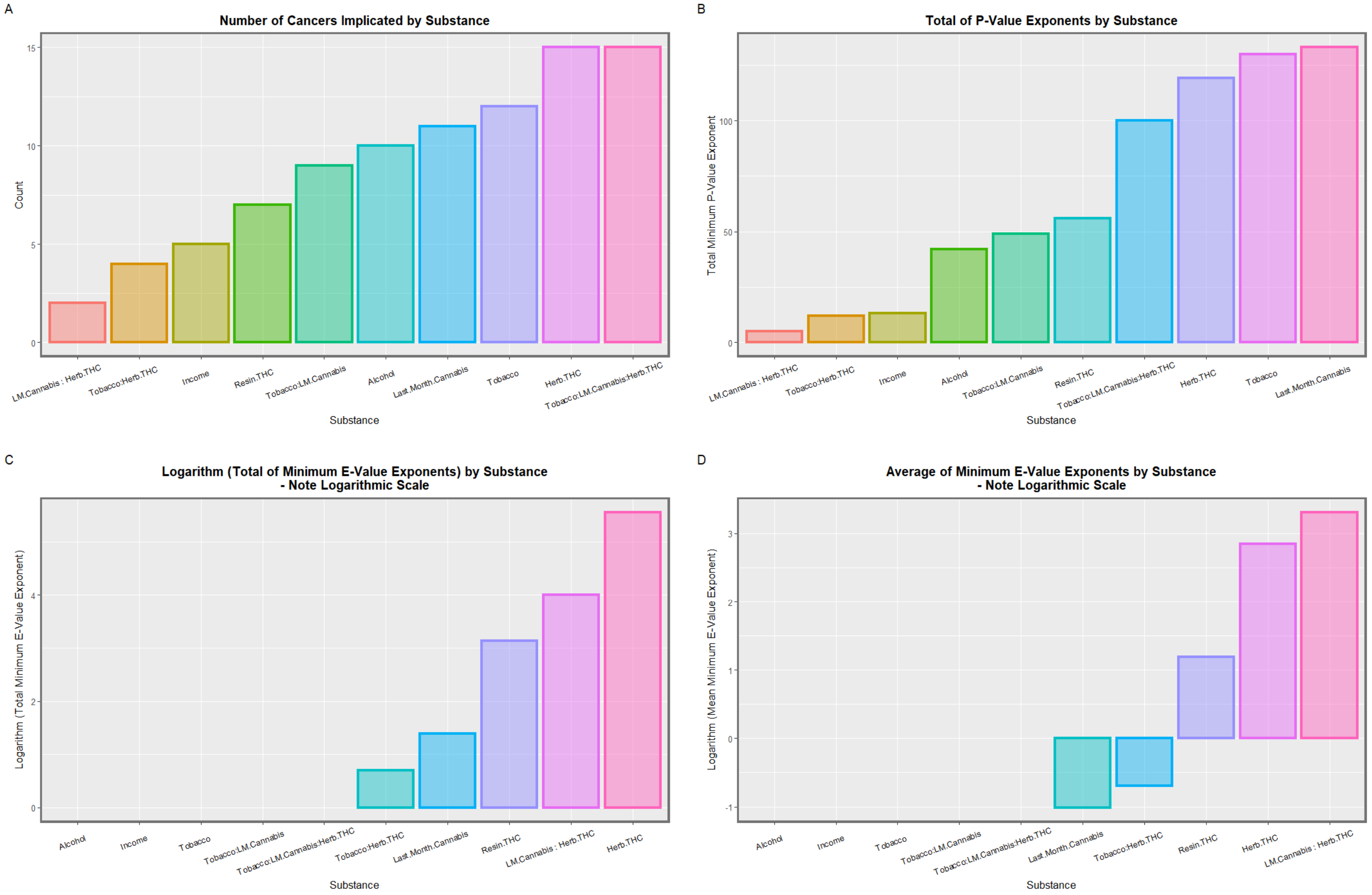
A
Number of Cancers Implicated by Substance
Count
0
5
10
15
LM.Cannabis : Herb.THC
Tobacco:Herb.THC
Income
Resin.THC
Tobacco:LM.Cannabis
Alcohol
Last.Month.Cannabis
Tobacco
Herb.THC
Tobacco:LM.Cannabis:Herb.THC
Substance
B
Total of P-Value Exponents by Substance
Total Minimum P-Value Exponent
0
50
100
LM.Cannabis : Herb.THC
Tobacco:Herb.THC
Income
Alcohol
Tobacco:LM.Cannabis
Resin.THC
Tobacco:LM.Cannabis:Herb.THC
Herb.THC
Tobacco
Last.Month.Cannabis
Substance
C
Logarithm (Total of Minimum E-Value Exponents) by Substance
- Note Logarithmic Scale
Logarithm (Total Minimum E-Value Exponent)
0
2
4
Alcohol
Income
Tobacco
Tobacco:LM.Cannabis
Tobacco:LM.Cannabis:Herb.THC
Tobacco:Herb.THC
Last.Month.Cannabis
Resin.THC
LM.Cannabis : Herb.THC
Herb.THC
Substance
D
Average of Minimum E-Value Exponents by Substance
- Note Logarithmic Scale
Logarithm (Mean Minimum E-Value Exponent)
-1
0
1
2
3
Alcohol
Income
Tobacco
Tobacco:LM.Cannabis
Tobacco:LM.Cannabis:Herb.THC
Last.Month.Cannabis
Tobacco:Herb.THC
Resin.THC
Herb.THC
LM.Cannabis : Herb.THC
Substance

Supplementary Figure S25.: Graphical summary of interactive panel model at four years of lag.

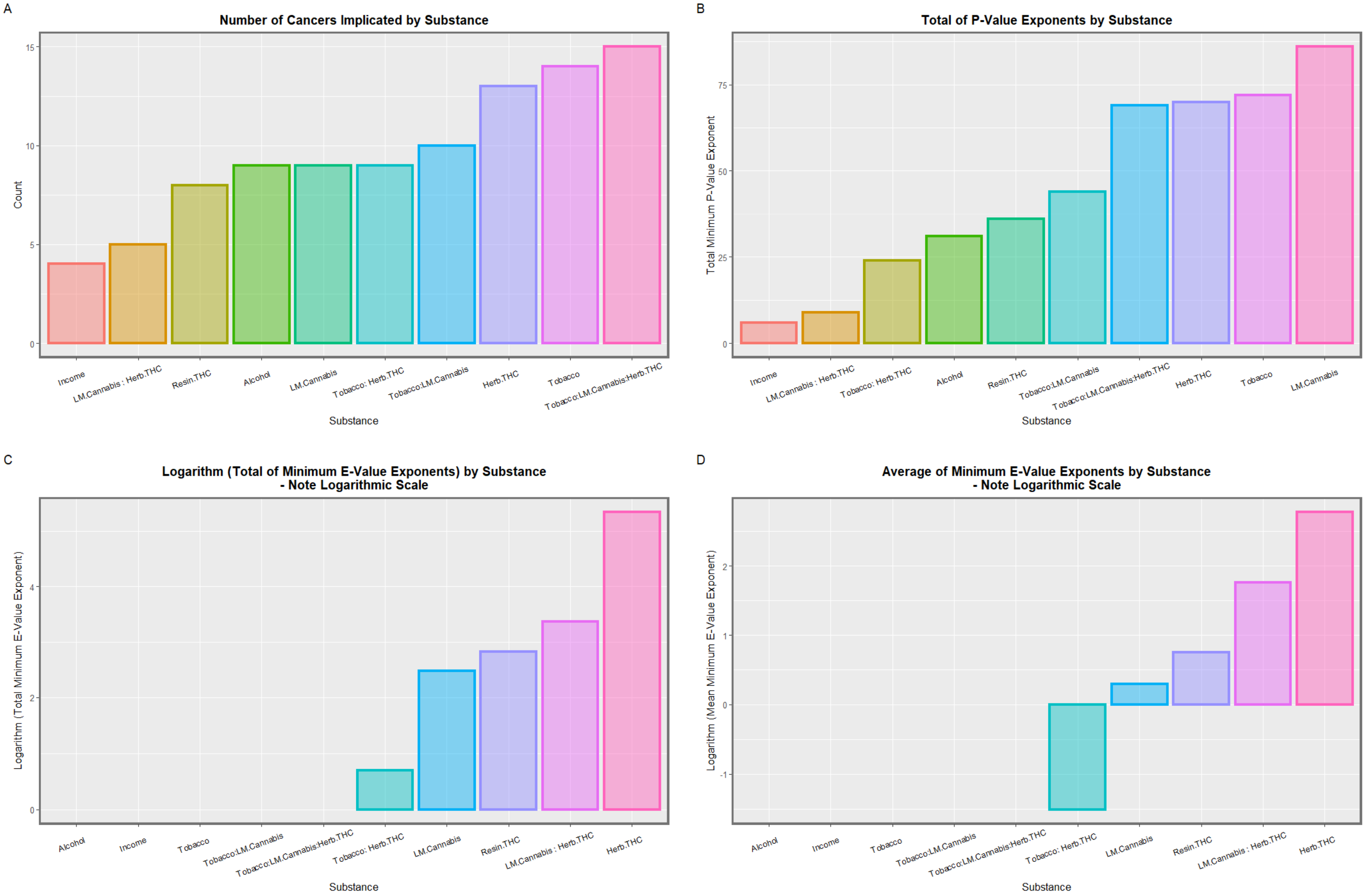
A
Number of Cancers Implicated by Substance
Count
0
5
10
15
Income
LM.Cannabis : Herb.THC
Resin.THC
Alcohol
LM.Cannabis
Tobacco: Herb.THC
Tobacco:LM.Cannabis
Herb.THC
Tobacco
Tobacco:LM.Cannabis:Herb.THC
Substance
B
Total of P-Value Exponents by Substance
Total Minimum P-Value Exponent
0
25
50
75
Income
LM.Cannabis : Herb.THC
Tobacco: Herb.THC
Alcohol
Resin.THC
Tobacco:LM.Cannabis
Tobacco:LM.Cannabis:Herb.THC
Herb.THC
Tobacco
LM.Cannabis
Substance
C
Logarithm (Total of Minimum E-Value Exponents) by Substance
- Note Logarithmic Scale
Logarithm (Total Minimum E-Value Exponent)
0
2
4
Alcohol
Income
Tobacco
Tobacco:LM.Cannabis
Tobacco:LM.Cannabis:Herb.THC
Tobacco: Herb.THC
LM.Cannabis
Resin.THC
LM.Cannabis : Herb.THC
Herb.THC
Substance
D
Average of Minimum E-Value Exponents by Substance
- Note Logarithmic Scale
Logarithm (Mean Minimum E-Value Exponent)
-1
0
1
2
Alcohol
Income
Tobacco
Tobacco:LM.Cannabis
Tobacco:LM.Cannabis:Herb.THC
Tobacco: Herb.THC
LM.Cannabis
Resin.THC
LM.Cannabis : Herb.THC
Herb.THC
Substance

Supplementary Figure S26.: Graphical summary of interactive panel model at six years of lag.
